# Impact of fermented foods consumption on gastrointestinal wellbeing in healthy adults: a systematic review and meta-analysis

**DOI:** 10.3389/fnut.2025.1668889

**Published:** 2025-10-10

**Authors:** Arghya Mukherjee, Dominic N. Farsi, Enriqueta Garcia-Gutierrez, Ecem Akan, Jose Angel Salas Millan, Ljupco Angelovski, Thomas Bintsis, Amaury Gérard, Ziba Güley, Sümeyye Kabakcı, Minna Kahala, Ryma Merabti, Foteini Pavli, Elisa Salvetti, Cem Karagözlü, Nurcan Bağlam, Bahtir Hyseni, Simona Bavaro, Konstantinos Papadimitriou, Eun-Hee Doo, Christophe Chassard, Smilja Praćer, Guy Vergères, Paul D. Cotter, Sandra Mojsova

**Affiliations:** ^1^Department of Food Biosciences, Teagasc, Fermoy, Cork, Ireland; ^2^APC Microbiome Ireland, Cork, Ireland; ^3^Human Nutrition Unit, INRAE, Université Clermont-Auvergne, Clermont-Ferrand, France; ^4^Department of Agronomic Engineering, Technical University of Cartagena, Murcia, Spain; ^5^Department of Dairy Technology, Faculty of Agriculture, Adnan Menderes University, Aydın, Türkiye; ^6^University College Cork, Cork, Ireland; ^7^Faculty of Veterinary Medicine, University Ss Cyril and Methodius, Skopje, North Macedonia; ^8^Faculty of Veterinary Medicine, Aristotle University of Thessaloniki, Thessaloniki, Greece; ^9^Brewing and Food Science Unit, LABIRIS, Anderlecht, Belgium; ^10^Department of Food Engineering, Alanya Alaaddin Keykubat University, Alanya, Türkiye; ^11^National Food Reference Laboratory, Ministry of Agriculture and Forestry, Ankara, Türkiye; ^12^Productions Systems, Food and Bioproducts, Natural Resources Institute Finland (Luke), Helsinki, Finland; ^13^Faculty of Natural and Life Sciences, Department of Cellular and Molecular Biology, Abbes Laghrour University, Khenchela, Algeria; ^14^Department of Food Sciences and Nutrition, University of Malta, Msida, Malta; ^15^Department of Biotechnology and Verona University Culture Collection (VUCC-DBT), University of Verona, Verona, Italy; ^16^Department of Dairy Technology, Ege University, Izmir, Türkiye; ^17^Department of Nutrition and Dietetics, Sivas Cumhuriyet University, Sivas, Türkiye; ^18^Faculty of Food Technology, University “Isa Boletini, Mitrovica, Republic of Kosovo; ^19^Institute of the Sciences of Food Production, National Research Council, Turin, Italy; ^20^Department of Food Science and Human Nutrition, Agricultural University of Athens, Athens, Greece; ^21^Department of Yuhan Biotechnology, School of Bio-Health Sciences, Yuhan University, Bucheon, Republic of Korea; ^22^UCA, INRAE, VetAgro Sup, UMRF, Aurillac, France; ^23^Institute for Biological Research Siniša Stanković, National Institute of the Republic of Serbia, University of Belgrade, Belgrade, Serbia; ^24^Agroscope, Bern, Switzerland; ^25^Vistamilk, Cork, Ireland

**Keywords:** fermented foods, gut microbiome, constipation, stool frequency, stool consistency, gastrointestinal health, bloating, flatulence

## Abstract

**Objective:**

In recent years, the consumption of fermented foods (FFs) has been linked with gastrointestinal health and wellbeing. Here, we systematically review and meta-analyse the currently available evidence relating to this as part of the COST Action PIMENTO and guided by the European Food Safety Authority (EFSA) health claim dossiers.

**Methods:**

MEDLINE, Scopus and Cochrane CENTRAL bibliographic libraries were searched for relevant literature up to 31st January 2025. All eligible studies were included for narrative review as per EFSA guidelines, but only randomised controlled trials (RCTs) were considered for meta-analyses. Risk of bias, mechanisms of action, bioactive compounds and safety were additionally discussed. Data was pooled using mean difference (MD)/standardized MD for continuous data and relative risk (RR) for dichotomous data. Certainty of evidence was evaluated through GRADE assessment.

**Results:**

A total of 25 studies (19 RCTs included in meta-analysis) with 4,328 participants were included in the systematic review. Meta-analysis demonstrated the beneficial impact of FF consumption on frequency of bowel movements (MD 0.60, CI 0.04, 1.16, *p* = 0.04, I^2^ = 74%), stool consistency (Bristol Stool Form Scale) (MD 0.25, CI 0.03, 0.47, *p* = 0.03, I^2^ = 72%), gastrointestinal symptoms (SMD −0.60, CI −1.05, −0.15, *p* = 0.009, I^2^ = 90%) and intestinal transit time (−13.65 CI −21.88, −5.43, *p* = 0.001, I^2^ = 95%), among others. Certainty of evidence was highly variable and mostly low.

**Conclusion:**

Our analysis suggests that FF consumption beneficially impacts the frequency of bowel movements, stool consistency, incidence of hard stools, intestinal transit time, abdominal symptoms, bloating, borborygmi, flatulence and degree of constipation.

**Systematic review registration:**

This study was registered at the Open Science Framework (osf.io, registration number: q8yzd).

## 1 Introduction

The modern human diet, particularly in industrialised nations, has been greatly impacted by food processing and preservation-related approaches developed in the 19th and 20th centuries. Indeed, diets in high-income countries with large urban populations now consist of many highly processed foods. Importantly, despite advances in large-scale production and the public health benefits of more hygienic food processing in highly controlled environments, there may be unforeseen negative consequences for human health (1). Indeed, recent research suggests that the industrialised, Western diet has contributed to the rise of several contemporary chronic metabolic, immune and “lifestyle” diseases (2, 3). The consumption of more foods that are sterile or have a low microbial load, and a concomitant decreased consumption of fermented foods (FFs), may also impact health, as proposed by the “Old Friends Hypothesis,” which argues that exposure to foodborne, non-harmful microbes provides an important source of stimuli to fine tune the immune system, improving gut function and rendering the symbiotic human less susceptible to the development of these chronic conditions (4). More recently, these suboptimal health conditions have been associated with dysbiosis of the gut microbiota and perturbation of associated gut microbial bioactive compounds (5).

Fermented foods and beverages, recently defined as “foods made through desired microbial growth and enzymatic conversions of food components,” have been consumed as staples of human diets for millennia, with Elie Metchnikoff first attributing good health and longevity to the consumption of fermented milk in 1910 (6–8). Indeed, FFs represent a unique category of foodstuffs that can act as an important vehicle to transfer beneficial microbes and bioactive components to the human gut and therefore have the potential to impact human health through various mechanisms (9–11). Several advances in our understanding of FFs have been made in recent years, including an ever-greater elucidation of their microbial and bioactive compositions as well as their health promoting potential (9, 12), with a concomitant resurgence of interest from the general population (13, 14). Evidence for health benefits of FFs have rapidly accumulated in recent years, catalysed by the emergence of omics-based technologies, particularly massive parallel sequencing technologies that have not only helped to understand the microbial composition and metabolic potential of FFs, but also their possible effects on the human gut microbiota. Genomic and metagenomic data have shown that FF microbiomes are taxonomically diverse, enriched in potentially health associated gene clusters, and can contain microbes that can be found in the gut as well as share metabolic capabilities of gut microbes (10, 15–17). Combined with an increasing number of *in vivo* trials, these advances have provided important insights into how FFs, which can contain probiotics, prebiotics and other bioactive compounds (9), might positively modulate the gut microbiota and the gut-brain axis (18), gastrointestinal wellbeing, and cardiovascular, immune and metabolic health, and alleviate symptoms related to lactose, raffinose and fructose intolerances, among others (19–25).

Gastrointestinal (GI) wellbeing, which relates to general, day-to-day wellbeing, is an important subcategory of GI health and can be impacted by consumption of FFs, through modulation of the gut microbiota or otherwise, as mentioned above. However, while evidence of the potential of FF consumption to prevent or address various diseases and suboptimal health conditions accumulate and has been reviewed elsewhere (21, 23, 26), a comprehensive qualitative and quantitative review of the impact of FF consumption specifically on GI wellbeing in the general population, an obvious area of public health interest, is currently lacking. In the present study, we systematically review the available evidence regarding the impact of FFs on GI wellbeing, contextualised through the research question: “Does consumption of fermented foods improve gastrointestinal wellbeing in typical, non-patient, healthy, adult populations?” To this end, we focused on investigating an array of GI symptoms that might be experienced regularly by the general population, such as GI discomfort or pain, bloating, borborygmi, flatulence, constipation, and associated physiological outcomes such as stool frequency and stool consistency, among others (see Table 1 for details). The present systematic review and meta-analysis is one among 16 conducted by Working Group 3 (WG3) of the COST Action CA20128—Promoting Innovation of Fermented Foods (PIMENTO) with the broader work guided by the European Food Safety Authority (EFSA) “Scientific and technical guidance for the preparation and presentation of a health claim application” (27) along with specific topical guidance from the EFSA guidance document “Guidance on the scientific requirements for health claims related to the immune system, the GI tract and defence against pathogenic microorganisms” (28). In accordance with the former, the present review will provide: (i) a systematic review of human studies; (ii) a non-systematic review of the characteristics of the investigated FFs; and (iii) a non-systematic review of evidence supporting the functional properties of the investigated FFs, in particular the mechanisms of action and the bioaccessibility and bioavailability of the active compounds. Additionally, the safety of relevant FFs is briefly discussed.

**Table 1:** Inclusion and exclusion criteria for population, intervention, comparator, outcomes and study designs.

Characteristic	Inclusion and exclusion criteria	Data extracted
Population	Included: Adults (> 18 years). No restrictions on age, sex, or ethnicity were applied. All settings including community and outpatient settings were included. Individuals with chronic constipation were included. Excluded: Adolescents (under 18 years of age), pregnant women, lactating and feeding mothers, individuals with professions that may lead to a leaner phenotype than usually observed in the normal population such as athletes, soldiers, astronauts in training, researchers in expeditions etc. Individuals with a BMI ≥ 30 kg m^−2^ (considered obese) or ≤ 18.5 kg m^−2^ (considered as being underweight in the general population), patients in critical care (chemotherapy, emergency ward, recurring infections etc). Individuals with IBS, IBD, functional constipation and functional dyspepsia were excluded. Interventions as adjuvants in populations undergoing treatment (e.g., triple therapy for *Helicobacter pylori* eradication) were excluded. Clinically diagnosed constipation patients were excluded	Age, sex, location, inclusion and exclusion criteria, number of participants in groups, potential confounding variables, e.g., smoking, alcohol intake, dietary intake and physical activity
Intervention	The intervention/exposure consists of the ingestion of any of the fermented foods (FFs) contained in the PIMENTO search string (Supplementary Table S1) (32) for FFs across the following food groups: dairy, meat and fish, fruits and vegetables, beverages, legumes, cereals and grains. Alcoholic beverages with an alcohol content of more than 1.25% were excluded. No limits were set for duration or dosage of the ingested fermented food(s). Studies investigating application of fermented foods other than for nutritional purpose (e.g., nasal or topical) were excluded. In addition, studies investigating probiotics were excluded unless the probiotic(s) is/are added at the beginning of the fermentation process and that there are indications from the literature that the probiotic strain(s) contribute(s) to the fermentation of the food matrix. Interventions including any possible confounders such as prebiotic fibres or added bioactive/fortification compounds were not included. Intervention could be designed as a stand-alone intervention or as a combined intervention if the comparator conditions are adequately controlled for non-fermented interventions.	Study product, fermenting genus, species and strain, ingredients, form, dose, schedule, and duration
Comparator	Most comparators, which would allow to isolate the beneficial effect of the fermentative procedure on health were included. The comparator (or control) can be the absence of consumption, or consumption of a lower amount or lower frequency of the fermented food/diet of interest or the consumption of a corresponding non-fermented food/diet. Any adequate non-fermented placebo or control (such as another medication or treatment) was also accepted as a valid comparator. Comparators were graded according to their appropriateness to the corresponding fermented intervention as: ‘Ideal’ (fermented and identical to intervention in taste, appearance, smell etc. with only active components and microbial components missing); ‘Good’ (non-fermented and identical to intervention in taste, appearance, smell etc. with only active components and microbial components missing); and ‘Bad’ (when above conditions are not met, such as no control or water as comparator).	Type, form, dose, schedule and duration
Outcomes	Studies reporting continuous or dichotomous data on various outcomes relevant to gastrointestinal wellbeing were included and reported. These were guided by the “Guidance on the scientific requirements for health claims related to the immune system, the gastrointestinal tract and defence against pathogenic microorganisms” (28) but not limited to it. Accordingly, some of the outcomes being considered in this review were broadly: (1) Symptoms associated with GI discomfort such as abdominal pain, cramps, bloating, straining, borborygmi (rumbling), and sensation of incomplete evacuation, among others. Reduction of GI discomfort is considered an indicator of improved GI function and hence a beneficial physiological effect (28); (2) Symptoms of excessive intestinal gas accumulation. The EFSA Nutrition, Novel Foods and Food Allergens (NDA) Panel has opined that the reduction of excessive intestinal gas accumulation generally leads to a reduction in GI discomfort, which is a beneficial physiological effect for the general population (28); (3) Maintenance of normal defaecation (a bowel function). The review investigated the impact of FF consumption on maintenance of normal defaecation (a bowel function) in the context: increasing the frequency of bowel movements, increasing faecal bulk, improving the consistency of stools (using Bristol Stool Form Scale, other study specific scales), and shortening transit time. Maintenance of normal defaecation is considered a beneficial physiological effect for the general population given that it does not result in diarrhoea (28). Additional outcomes recorded included integrative symptom questionnaires, quality of life questionnaires, faecal pH, faecal water content, and constipation-related symptoms, among others. It must be noted that not all outcomes that were recorded were ultimately included and/or discussed in the review or meta-analysis. Outcomes related to changes in the gut microbiota composition were not considered eligible for the work as such outcomes are not considered by EFSA as substantive on their own (28). They are however, accepted as supporting evidence, for example, for elaborating mechanisms of action; this has been included in the current study.	Outcomes, measurement methodology, baseline, midpoint, and endpoint values and change from baseline, details of compliance and adverse events
Study designs	All human studies were searched systematically and included according to article 4.2.1. of the EFSA guidance (27). These included (i) publications reporting human intervention (efficacy) studies (e.g., randomised controlled studies, randomised uncontrolled studies, non-randomised controlled studies, other intervention studies such as repeated measures studies), (ii) publications reporting human observational studies (e.g., cohort studies, case–control studies, cross-sectional studies, other observational studies). Reference lists in relevant systematic reviews with or without meta-analysis were also hand screened for potentially missing studies. Included studies were always peer reviewed and published to be eligible for inclusion. Animal and *in vitro* studies were excluded (only considered for supportive evidence sections—Section 5 and 6 under Results and Discussion). Studies recording only effects on gut microbiota were not eligible for inclusion. Non-peer reviewed documents such as commentaries, preprints, conference proceedings and abstracts, lectures, Letters to the Editor, book chapters, posters, clinical trial registries, and grey literature were excluded. Documents without abstracts were removed.	Study design, washout period duration, type of analysis (intention to treat or per protocol), number of participants excluded and reasons, method of randomisation, allocation concealment, blinding, funding source, funder involvement, conflicts of interest

## 2 Materials and methods

The systematic review and meta-analysis carried out in the present work was performed in accordance with the guidelines presented in the Cochrane Handbook (29) and reported according to the updated Preferred Reporting Items for Systematic Reviews and Meta-Analyses (PRISMA) (30). The design, coordination, progress, updating, and evidence summarisation of the current systematic review were carried out according to the steps outlined by Muka et al. (31). The inclusion/exclusion criteria, search strategy, screening methodology, data extraction, and analyses pipelines were set in the protocol and are available at the Open Science Framework^1^ as well as through a previous position paper from the PIMENTO WG3 (32). The systematic review and meta-analysis were carried out by the E1 subgroup of the PIMENTO WG3, consisting of 17 researchers, including co-leaders PC, AM, and SM. The workings of E1 were further supervised by WG3 co-leads GV and ST, with internal reviews carried out by co-leads of WG3 sub-group E2, SB, BH, and KP.

### 2.1 Eligibility criteria

Eligibility criteria for the present work were developed using the PICOS (Population, Intervention, Comparator, Outcome, Studies) methodology and are elaborated in Table 1. Briefly, we included human studies investigating the effect of FFs consumption compared to appropriate placebos or controls on GI wellbeing in a healthy, non-patient population. For the purposes of this study, ‘gastrointestinal wellbeing’ is defined as a state of health where a normal, healthy, typical, non-patient population experiences physiologically optimal or improved gastrointestinal functions, and in turn, can maintain a lifestyle free of intermittent remedial consultations with doctors, lifestyle interruptions (from GI symptoms such as abdominal pain, flatulence, bloating, and others), or equivalent. Details of the outcomes considered in this regard and the rationale for their choice are provided in Table 1 and were used to screen the eligible studies.

### 2.2 Literature search

Studies were identified through a systematic search of electronic databases and manual searches of the reference lists in relevant systematic reviews. The following electronic databases were searched on March 5, 2024: MEDLINE (January 1970 to August 2023), Scopus (January 1970 to August 2023) and The Cochrane Central Register of Controlled Trials (all years; The Cochrane library). A final top-up search was carried out up to 31st January 2025. Search strategies are presented in Supplementary Table S1 and have been reported previously (32).

### 2.3 Study selection

Once retrieved, references were imported into a systematic review manager software, CADIMA (33), and deduplicated. Study selection was conducted roughly in accordance with the guidance of Muka et al. (31): steps 4 (Define selection criteria), 8 (Collection of references and abstracts in a single file), 9 (Elimination of duplicates), 10 (Screening of the titles and abstracts by at least two reviewers), 11 (Collection, comparison, and selection of references for retrieval), 12 (Retrieval of full text and application of selection criteria), 13, if needed (Contact experts), and 14 (Search for additional references). Before title and abstract screening, a consistency check was carried out on a subset of the literature dataset between members of the E1 subgroup, where the members used the set population, intervention, and outcome (PIO) criteria to screen documents. The wording and interpretation of the PIO selection strategy were further adapted to improve the efficiency, accuracy, and systematicity of the reviewing process based on the results of the consistency check. Subsequently, members of E1 screened the title and abstract, and later the full text, using predefined inclusion and exclusion criteria, with at least two members reviewing each document. Once all studies that met the PIO criteria were selected, the remaining articles were evaluated for comparators. Defining appropriate controls is often difficult in nutritional science. To highlight the research gaps on this issue, we collected comparator data from all human studies, as described in the EFSA guidance (27). Accordingly, human studies were selected irrespective of the quality of the control, with the only criterion being that the comparator cannot be fermented to enable comparison with a fermented intervention. However, we added a gradation system for comparators to enable an understanding of the suitability of the comparator being used (see Table 1 for details). Disagreements throughout the study selection process were resolved through discussions with AM and SM.

### 2.4 Data extraction

Data extraction was conducted based on the guidance of Muka et al. (31): steps 5 (Design data collection form), 16 (Application of the data collection form), and 18 (Preparation of the database for analysis). The data extraction form(s) of the interventional and observational studies were based on combined information provided in the handbook of the Cochrane interactive learning course “Conducting an Intervention Review” (29), Appendix B of “Information to be presented in a full study report for human efficacy studies” of the EFSA guidance (27) and the STROBE guidelines for reporting observational studies (34), respectively. A standardised data extraction form was created for the extraction of relevant data from the selected studies, where at least two Reviewers independently extracted the data. Recorded data were compared, and discrepancies were resolved by AM and SM. In cases where the article provided insufficient data or in a form that was not usable in the present review, the authors were contacted to provide additional information. When trial reporting was allowed, the data were extracted for intention-to-treat analyses. Therefore, when considering dichotomous data, dropouts were considered as intervention failures. In cases where this was unclear, the analysis was carried out on all participants with reported data deemed evaluable. When necessary and possible, data were extracted from figures using the open-source WebPlotDigitizer software (35).

### 2.5 Risk of bias assessment

Risk of bias was assessed by a subgroup of E1 involving five Reviewers (AM, DF, SM, LA, and SK) who were trained in quality and bias assessments through online and in-person workshops organised through PIMENTO. The Reviewers were divided into two groups and assigned a subset of documents that passed full-text evaluation. Each document was reviewed by at least two Reviewers (36). Differences in judgements were resolved through group discussion among the five Reviewers. The Cochrane risk-of-bias 2 tool (RoB 2.0), which evaluates bias arising from randomisation, blinding, missing outcome data, deviations from intended trial protocol, outcome measurement, and selective reporting, among others, was used to assess randomised controlled trials (RCTs) (36). For crossover studies (RCoT), an additional domain that assesses period and carryover effects are included in the tool. Trial evaluations were classified as “low risk,” “some concerns,” and “high risk” following the RoB2 guidelines. Information gleaned from any study protocols and/or clinical trial registrations that could be obtained was used to ensure that the final publication results corresponded to the pre-specified outcomes. Non-randomised intervention trials were assessed for risk of bias using the ROBINS-I v2.0 tool (37), with studies classified as being at “low,” “moderate,” “serious” or “critical” risk of bias. For observational studies, risk of bias was assessed using the Newcastle-Ottawa quality assessment scale (NOS) (38). The NOS employs a ‘star system’ through which a study is evaluated on three broad perspectives: selection of study groups, comparability of the groups, and the ascertaining of either the outcome of interest or the exposure for cohort studies and case–control studies, respectively. NOS scores were in turn used to categorise observational studies as “Good,” “Fair” or “Poor” quality as per the Agency for Healthcare Research and Quality standards for NOS evaluation of observational studies (39). As for RCTs, any conflicting judgements for assessment of observational studies were resolved through group discussion among the Reviewers.

### 2.6 Data synthesis and statistical analysis

Data synthesis was based on steps 19 (Conduct descriptive synthesis) and 23 (Check the quality of the evidence: the confidence in the results presented) of Muka et al. (31). The quantitative analysis (meta-analysis) of the data was conducted using appropriate statistical approaches according to Module 6 (Analysing the data) of the handbook of the Cochrane interactive learning course “Conducting an Intervention Review” (29). Meta-analysis was performed for outcomes that were reported in at least two studies using RevMan Web version 9.0.0 (40). Dichotomous outcomes were evaluated using the Mantel–Haenszel method and expressed as Risk Ratio (RR) and 95% confidence interval (CI) (41, 42). A mean difference (MD) was calculated for continuous outcomes that were measured using the same instrument and reported in the same units or where the reported units could be directly converted to units used for calculations (e.g., stool frequency per day to per week). For continuous outcomes that were measured using different units or reported differently, a standardised mean difference (SMD) was calculated using Hedges’ (adjusted) “g” as employed in RevMan (29). In case of cross-over studies, the data for intervention and control periods were recorded separately, with only the data from the first period used in the meta-analysis (43). Primary metrics extracted for analyses were means, standard deviations (SDs), sample sizes, and *p*-values. SDs were calculated from standard errors (SEs) or 95% CI where applicable. Means and SDs were additionally imputed from medians and interquartile values using methods previously described by Wan et al. (44) as recommended by the Cochrane Handbook for Systematic Reviews of Interventions (29). Where applicable, changes in mean and SD at intervention endpoint from baseline were recorded, with missing SD values imputed by methods recommended by Cochrane (a correlation coefficient of 0.5 was imputed where applicable) (29). For studies with multiple intervention arms (e.g., studies where placebo is compared against two different doses), each intervention was compared to intervention separately, and the sample size of the control group was divided by the number of intervention arms to reduce unit-of-analysis error (29).

A random-effects model was used to carry out meta-analyses; the model was chosen as it accounts for variation in effects across studies inherited from heterogeneity and as it is more suitable for generalising results beyond the meta-analysis (45). Analysis of heterogeneity was based on step 21 (Exploration of heterogeneity) of Muka et al. (31) with confidence intervals for the summary effect calculated using the Wald method (29). Heterogeneity was assessed using the Chi-squared test and quantified with the I^2^ statistic and Tau^2^ estimated using the restricted maximum likelihood (REML) method (46). For evaluating heterogeneity in meta-analysis of dichotomous outcomes, the DerSimonian and Laird method was used to determine Tau^2^, as recommended by Cochrane (29, 47). As recommended by Cochrane, thresholds of 50 and 75% for the I^2^ statistic indicated substantial and considerable heterogeneity, respectively (29). Further, subgroup analyses were conducted to investigate heterogeneity and understand the effects of fermentation matrix/substrate type, fermenting microbes, dosage of microbes, and duration of intervention, among others. Subgroup analyses were conducted according to Module 6 (analysing the data) of the handbook of the Cochrane interactive learning course “Conducting an Intervention Review” (48). A *p*-value < 0.1 was considered statistically significant for subgroup analyses (49). For studies where outliers or “high risk of bias” are observed, sensitivity analysis was undertaken and as recommended by Cochrane, data from analysis both with and without outliers were reported (29). Publication bias, if applicable, was determined through funnel plots for meta-analysis including ≥ 10 studies with evidence of asymmetry identified through visual inspection (29).

### 2.7 Certainty of evidence assessment

An evaluation of the quality of the evidence derived from the human studies and included in the meta-analysis was conducted for each outcome following the Grading of Recommendations Assessment (GRADE) approach (50) according to Module 7 (Interpreting the findings) of the handbook of the Cochrane interactive learning course “Conducting an Intervention Review” (48). GRADE evaluation was carried out using the GRADEpro GDT software (51). Briefly, factors such as risk of bias, consistency of effect, imprecision, indirectness and publication bias (for downgrading), as well as large magnitude of effect, dose–response gradient, effect of potential residual confounding factors (for upgrading) were considered for grading the outcomes into “high,” “moderate,” “low” and “very low” certainty of evidence. The GRADE assessments were used to compose the summary of findings table.

### 2.8 Non-systematic review of food characteristics, mechanism of action and safety

Following the requirements of the EFSA, we also carried a non-systematic, exploratory, narrative review of the diverse characteristics of the foodstuffs included in this review (biological, nutritional and other compositional characteristics, manufacturing and fermentation protocols, shelf-life, aspects of food safety etc.) as well as the supportive evidence available in context of the outcomes and studies described in this work (including excluded human trials, animal trials and *in vitro* experiments, among others). Concerning the latter, relevant experiments and evidence, including associative evidence from studies on the gut microbiota, was discussed, focusing on the key mechanisms that might affect the interactions between the different components of FFs and the outcomes being considered in this review. Finally, the safety issues (side effects etc.) encountered for the given interventions were also recorded in brief.

## 3 Results and discussion

### 3.1 Identification of pertinent human efficacy studies

**Figure 1:**
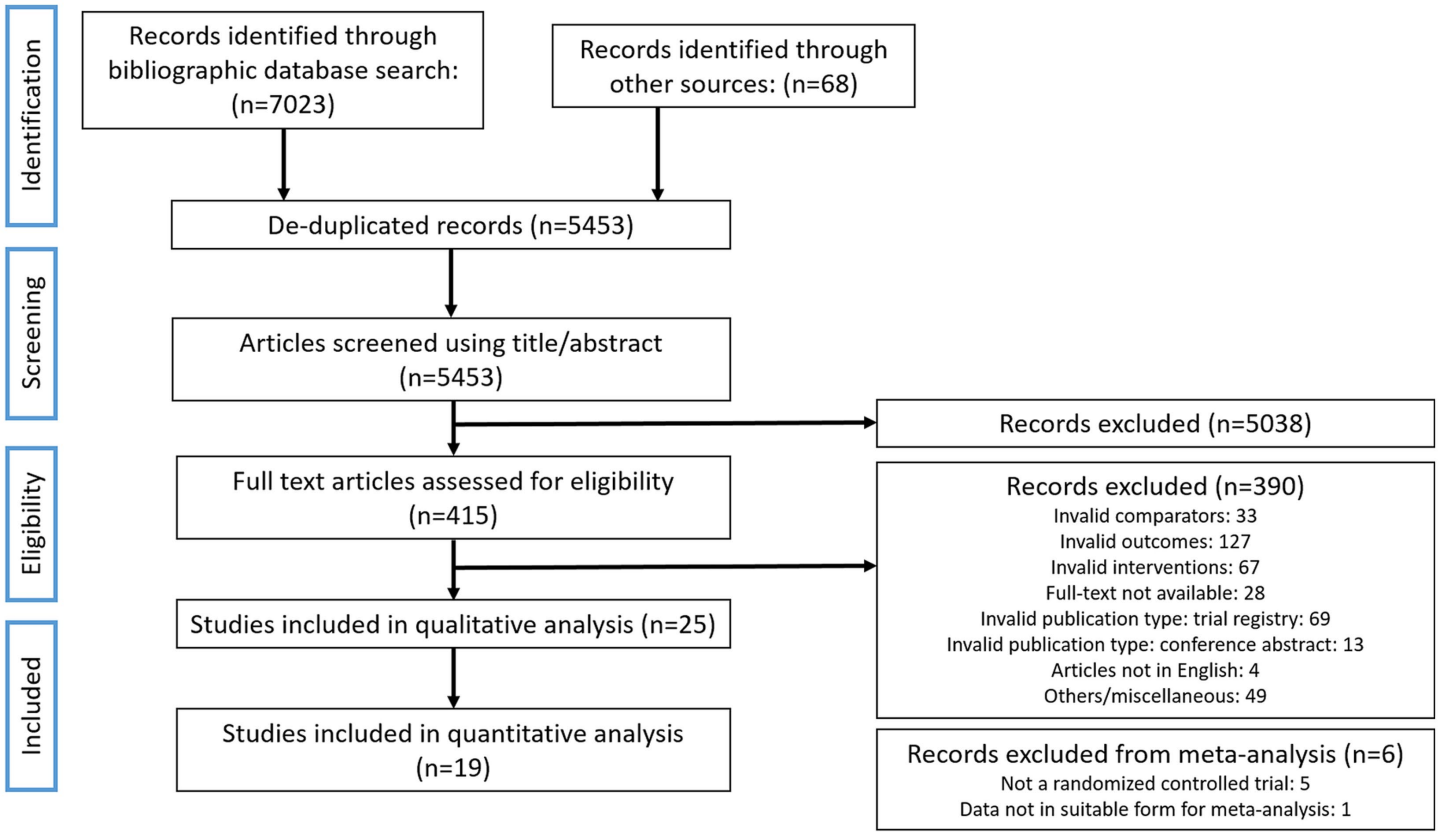
Bibliographic search flowchart. The flowchart outlines the literature search and the progression of the evaluation of studies in this systematic review as recommended by the PRISMA-SR guidelines.

The bibliographic search for the current work was carried out according to the PICOS criteria set out in Table 1. A total of 5,453 non-duplicated documents were retrieved in the primary bibliographic search (search strategy presented in Supplementary Table S1) with 415 deemed eligible for full-text screening after screening of their title and abstract (Figure 1). Of these, 390 studies were excluded due to incompatibility with inclusion criteria and 25 studies were retained for final qualitative analysis and review (Table 1, Figure 1). Several potentially eligible records (*n* = 28) had to be removed from the screening as full-texts were not available. The 25 eligible studies included 4 non-randomised studies, 1 observational study, 2 randomised crossover studies (RCoT), with the rest being randomised, controlled trials (RCTs) (Table 2). Among these 25 studies, 19 were retained for a quantitative meta-analysis with a total participant count of 4,328; 5 studies were removed from meta-analysis as they were not randomised, controlled trials (52–56), and 1 was removed as the data format presented was not suitable for a meta-analysis (57) (Figure 1). Detailed characteristics for all included studies, totalling 4,328 participants, are tabulated in Table 2. For two RCoTs, data from only the first period were included in the meta-analysis due to concerns with inadequate washout (58, 59). Three studies were separated into two cases of RCTs each. This reflected the fact that, for one study, two different doses of the FF intervention were employed (60), and for two other studies, two different cohorts, both eligible, were included (58, 61). Interestingly, a substantial number of eligible studies were found to be conducted in Japan (*n* = 13), with France and the Netherlands being represented by three and two studies, respectively; seven additional countries were represented by a single study (Table 2). Among fermenting microorganisms, *Lacticaseibacillus casei* strain Shirota was the most reported microbe present among the eligible studies (*n* = 8) (Table 2). The duration of interventions varied considerably across the included studies, ranging from 2 to 24 weeks, with the most widely used duration being 2 and 4 weeks (Table 2). Potential confounding factors were reported in most studies, including dietary intake (*n* = 24), alcohol use (*n* = 2), medications (*n* = 22), comorbidities (*n* = 16), indicators of nutritional status (*n* = 19), smoking (*n* = 6), physical activity (*n* = 8) and assessment of gut microbiota (*n* = 14). Further information can be found in Supplementary Table S5. Unfortunately, although intestinal gas accumulation was one of the outcomes of interest, eligible studies that addressed this were not found. Additionally, we also found that the outcome of gastrointestinal well-being was reported in Guyonnet et al. only among the eligible studies (60).

**Table 2:** Characteristics of eligible studies included in the systematic review investigating the effect of fermented foods consumption on gastrointestinal wellbeing.

Study, year, country (reference)	Study design	Sample size (% female)	Age (years), mean (range)^$^	Intervention microbes; total microbial dose	Fermentation matrix	Intervention duration (weeks)	Comparator (grade)^£^	Relevant outcomes and effect of intervention (meta-analysed)*
Noda et al., 2024, Japan (67)	Randomised, double-blind, placebo-controlled, parallel-group	Placebo (*n* = 25; NI^a^); Fermented intervention (*n* = 25; NI)	Placebo: 56.5 (20–74); Fermented intervention: 58.8 (20–74)	*Lactococcus lactis* BM32-1; 2 × 10^10^ CFU/d^b^	Sericin-fibroin mixture	12 weeks	100 mL of 1.52% (w/v) maltodextrin solution/d. (bad)	(Stool frequency [POS], Stool consistency [NEU], Stool bulk [NEU], Stool consistency (BSFS) [NEU])
Tanihiro et al., 2024, Japan (68)	Randomised, double-blind, placebo-controlled	Placebo (*n* = 60; 36.7%); Fermented intervention (*n* = 57; 36.8%)	Placebo: 46.3 (20–59); Fermented intervention: 46.9 (20–59)	*Lactobacillus helveticus* CP790; 1 × 10^10^ CFU/day	Milk	4 weeks	10 mL of placebo beverages: skim milk and whey protein concentrate, and adding lactic acid to match the appearance, taste, nutritional content, and pH of the test beverages. (Good)	(Abdominal symptoms [NEU], Stool frequency [NEU], Stool consistency [POS], feeling of incomplete evacuation [NEU], Straining during defaecation [POS], Stool consistency (BSFS) [POS])
Kaga et al., 2024, Japan (69)	Randomised, double-blind, placebo-controlled	Placebo (*n* = 55; 65.4%); Fermented intervention (*n* = 57; 66.7%)	Placebo: 42.5 (20–60); Fermented intervention: 43.4 (20–60)	*Lcb. paracasei* Shirota; 3 × 10^10^ CFU/day	Soy milk	4 weeks	100 mL/day of placebo: Unfermented Soy Milk contained 0.09 g/100 mL raffinose and 0.39 g/100 mL stachyose. Lactic acid was added to the placebo to achieve the same pH as the intervention. (Good)	(Stool pH [NEU], Stool frequency [POS], Stool water content [NEU])
Nagata et al., 2016, Japan (61)	Randomised, double-blind, placebo-controlled, parallel-group	Elderly cohort: Placebo (*n* = 36; 75%); Fermented intervention (*n* = 36; 72.2%); Nursing cohort: Placebo (*n* = 10; 70%); Fermented intervention (*n* = 10; 80%)	Elderly cohort: Placebo: 86 (NI); Fermented intervention: 84 (NI); Nursing cohort: Placebo: 38 (NI); Fermented intervention: 36 (NI)	*Lcb. paracasei* Shirota; 4 × 10^10^ CFU/day	Milk	24 weeks	Placebo (80 mL/bottle) was dosed every day. The placebo consisted of skim milk, high-fructose,corn syrup, and flavoring. Taste, texture, appearance etc. were adjusted same as intervention. (Good)	Constipation incidence [POS], (Stool pH [POS])
Marteau et al., 2013, France (66)	Randomised, double-blind, placebo-controlled, parallel-group	Placebo (*n* = 162; 100%); Fermented intervention (*n* = 162; 100%)	Placebo: 33 (18–60); Fermented intervention: 32.3 (18–60)	*Bifidobacterium lactis* I-2494/DN-173010, *Lc. lactis* CNCM I-1631, *Streptococcus thermophilus* CNCM I-1630, *Lactobacillus delbrueckii* subsp. *bulgaricus* (*L. bulgaricus*) CNCM I-1632/I-1519; 2.74 × 10^10^ CFU/day	Milk	4 weeks	Two cups of placebo daily ingested. The control product was a milk-based non-fermented dairy product without probiotics and made similar to the test product through enzymatic acidification with similar flavour, appearance, texture, and taste. (Good)	Gastrointestinal well-being [NEU], (Abdominal symptoms [POS])
Ling et al., 1992, Finland (54)	Repeated measures pilot study	6 (NI)	78–91	*Lcb. rhamnosus* GG; 2 × 10^10^ CFU/day	Whey	2 weeks	No true control as not an intervention study. 200 mL of apple-peach drink every day for 2 weeks before intervention	Stool pH [NEU], stool frequency [NEU], hard stools [POS], stool bulk [NEU]
Guyonnet et al., 2009, Germany and France (62)	Randomised, double-blind, placebo-controlled, parallel-group	Placebo (*n* = 97; 100%); Fermented intervention (*n* = 100; 100%)	Placebo: 32.5 (18–60); Fermented intervention: 31.9 (18–60)	*Bifidobacterium lactis* I-2494/DN-173010, *Lc. lactis* CNCM I-1631, *Streptococcus thermophilus* CNCM I-1630, *L. bulgaricus* CNCM I-1632/I-1519; 2.74 × 10^10^ CFU/day	Milk	4 weeks	125 g serving twice a day for placebo. The control product was a milk-based non-fermented dairy product without probiotics and acidified using an enzymic process. Control product had similar appearance, flavour, texture and taste. (Good)	Gastrointestinal well-being [POS], (Abdominal pain [NEU], Bloating [NEU], Borborygmi [POS], Flatulence [POS], Abdominal symptoms [POS], Stool frequency [NEU], Stool consistency [POS])
Sakai et al., 2011, Belgium (72)	Randomised, open label, controlled, parallel-group	Control (*n* = 20; 60%); Fermented intervention (*n* = 19; 57.9%)	Control 32.1 (18–65); Fermented intervention 35.4 (18–65)	*Lcb. paracasei* Shirota; 6.5 × 10^9^ CFU/day	Milk	3 weeks	Normal diet without consumption of intervention product (Bad)	Abdominal discomfort [NEU], (Stool frequency [POS], Stool consistency [POS], Stool water content [NEU], Hard stools [POS], feeling of incomplete evacuation [NEU], Straining during defaecation [NEU], Stool consistency (BSFS) [POS])
Nemoto et al., 2011, Japan (59)	Randomised, double blinded, placebo-controlled, crossover	Control (*n* = 18; 66.7%); fermented intervention (*n* = 18; 55.5%)	Control 32.1 (18–65); Fermented intervention 35.4 (18–65)	*Aspergillus oryzae*; NI	Brown rice	2 weeks	3.5 g packs made with 21 g ingestion every day. Non-fermented control was made from roasted flour and cornstarch (85:15, W/W). No fermenting microbes present. (Good)	Stool frequency [NEU], stool bulk [NEU], stool consistency [NEU], stool water content [NEU], (stool pH [NEU])
Takii et al., 2013, Japan (55)	Controlled, parallel group	Non-constipation group: Placebo (*n* = 24; 100%); viable cell (*n* = 16; 100%); dead cell (*n* = 14; 100%)	Non-constipation group: 20.2 (18–21)	*Levilactobacillus brevis* NSB2; 8.1 × 10^7^ CFU/day	Turnips	2 weeks	30 g sterilized turnips taken per day. No fermentation. (Good)	For the non-constipation group: stool frequency [NEU], feeling of incomplete evacuation [POS], hard stools [POS]
Guyonnet et al., 2009, UK (60)	Randomised, open label, controlled	Control (*n* = 69; 79.7%); 1-pot fermented intervention: (*n* = 144; 77%); 2-pot fermented intervention: (*n* = 147; 74.8%)	Control 38.5 (18–65); 1-pot fermented intervention: 39.5 (18–65); 2-pot fermented intervention: 38.5 (18–65)	*B. lactis* DN-173010, *S. thermophilus* and *L. bulgaricus*; 1 pot: 1.37 × 10^10^ CFU/day; 2-pot: 2.74 × 10^10^ CFU/day	Milk	2 weeks	Normal diet without consumption of intervention product. (Bad)	Abdominal pain [NEU], Bloating [POS], Borborygmi [POS; 1-pot], Flatulence, Abdominal symptoms [POS; 1-pot], Degree of constipation [NEU], general digestive wellbeing [POS], intestinal gas accumulation [POS]
Kinoshita et al., 2021, Japan (63)	Randomised, open label, controlled, parallel intervention	Control (*n* = 482; 100%); Fermented intervention (*n* = 479; 100%)	Control: 39.4 (20–71); Fermented intervention: 39.3 (20–71)	*L. bulgaricus* OLL1073R-1 and a strain of *S. thermophilus*; 1.12 × 10^9^ CFU/day	Milk	16 weeks	No control product. No consumption of intervention product as control. (Bad)	Abdominal symptoms [NEU], abdominal pain [NEU], degree of constipation [POS]
Galena et al., 2022, USA (57)	Randomised, double-blinded, controlled, parallel intervention	Fermented vegetables (*n* = 10; 100%); Control (*n* = 10; 100%)	Fermented vegetables 37.4 (18–69); Control 29.8 (18–69)	Complex mix of microbes in the fermented vegetables. Mainly *Lactobacillus* spp., *Leuconostoc* spp., and *Weissella* spp.; NI	Cabbage/cucumber	6 weeks	No control product. No consumption of intervention product as control. (Bad)	Stool consistency^c^, bloating^c^, abdominal pain^c^
Kata-Kataoka et al., 2016, Japan (70)	Randomised, double-blinded, controlled, parallel intervention	Placebo: *n* = 24 (45.8%); Fermented intervention: *n* = 23 (47.8%)	Placebo: 22.8 (NI); Fermented intervention: 22.8 (NI)	*Lcb. paracasei* Shirota; 1 × 10^11^ CFU/day	Milk	8 weeks	100 mL of placebo milk was administered per day to control subjects. Placebo was nonfermented milk with the same nutritional content, color, flavour, taste, and pH as the fermented intervention. (Good)	(Abdominal pain [POS], Bloating^c^, Abdominal symptoms [POS], feeling of incomplete evacuation^c^, Straining during defaecation^c^, Degree of constipation [NEU]), Total GSRS^&^ score [POS]
Matsumoto et al., 2010, Japan (71)	Randomised, double-blinded, controlled, parallel intervention	Placebo: *n* = 16 (28.6%); Fermented intervention: *n* = 14 (47.8%)	Placebo: 44.6 (NI); Fermented intervention: 40.3 (NI)	*Lcb. paracasei* Shirota; 4 × 10^10^ CFU/day	Milk	4 weeks	80 mL of placebo administered per day. Ingredients same as test products with fermentation and microbes. (Good)	(Stool pH [NEU], Stool frequency [NEU], Stool consistency [NEU], Stool bulk [POS], Stool water content [NEU])
Spanhaak et al., 1998, Netherlands (76)	Randomised, double-blinded, controlled, parallel intervention	Placebo: *n* = 10; Fermented intervention: *n* = 10	Mean for total cohort: 55.8 (40–65)	*Lcb. paracasei* Shirota; 3 × 10^11^ CFU/day	Milk	4 weeks	3 × 100 mL placebo administered per day. The placebo was same amount of unfermented milk having a similar basic composition as the fermented product and packaged in identical bottles.	(Stool pH [NEU], Intestinal transit time [POS], Stool water content [POS])
Meance et al., 2003, Italy (58)	Randomised, open label, controlled crossover	Slow transit time: Group A: *n* = 40 (NI); Group B: *n* = 38 (NI); Medium transit time: Group A: *n* = 40 (NI); Group B: *n* = 41 (NI)	Slow transit time: Group A: 63.5 (50–75); Group B: 64.1 (50–75); Medium transit time: Group A: 63.5 (50–75); Group B: 63.1 (50–75)	*B. lactis* DN-173010, *S. thermophilus* and *L. bulgaricus*; Group A: 1.375 × 10^10^ CFU/day; Group B: 2.75 × 10^10^ CFU/day	Milk	2 weeks	No intake control; no control product. (Bad)	(Intestinal transit time [POS])
Ozaki et al., 2018, Japan (75)	Randomised, double-blind, placebo-controlled, parallel-group	Placebo: *n* = 16 (100%); fermented intervention: *n* = 15 (100%)	Total cohort mean age: 20.1 (18–31)	*Lc. lactis* subsp*. cremoris* FC; 2 × 10^10^ CFU/day	Milk	4 weeks	200 g per day dosage. The placebo product was non-fermented gelled milk that had a similar texture and appearance with the test product. (Good)	(Stool pH [NEU], Stool frequency [NEU], Stool bulk [NEU], Stool water content [NEU], Hard stools [NEU])
Aslam et al., 2021, Australia (77)	Observational study	Geelong osteoporosis study cohort: *n* = 1,241 (50.9%)	Mean age across cohort: 55	Dairy fermenting microbes; NA	Milk	NA	NA	Association between fermented dairy consumption and constipation [NEU]
Kurahashi et al., 2021, Japan (65)	Randomised, double-blind, placebo-controlled, parallel-group	Total cohort: *n* = 44 (59%)	Total cohort: 20–66 years (NI on means)	*A. oryzae*; 302 ± 15.5 mg of *A. oryzae* cells per 118 g serving of koji amazake (not used in meta-analysis)	Koji rice	3 weeks	Placebo was administered once a day (118 g/bottle). Rice syrup (prepared using hydrolyzing enzymes without fermentation with *A. oryzae*) as was used as placebo.(Good)	Stool pH [NEU], Stool frequency [POS], Stool consistency [NEU], Stool bulk [NEU]
Tanaka et al., 2021, Japan (56)	Repeated measures study	Total cohort: i = 20 (45%)	Total cohort: 52 (40–64)	Autochthonous microorganisms in *Brassica rapa* L.; NI	*Brassica rapa* L.	4 weeks	Repeated measures study. No control products. (Bad)	Stool frequency [POS], stool consistency [NEU]
Alves et al., 2022, Portugal (52)	Controlled, parallel	Atopic group: *n* = 19 (94.7%); healthy group: *n* = 33 (82%)	Atopic group: 31.7 (19–56); healthy group: 27.0 (20–60)	Kefir grain consortium from CIDCA AGK1 kefir grains; NI	Milk	8 weeks	No kefir intake, (Bad)	Degree of constipation [POS], abdominal pain [POS], bloating [POS], flatulence [NEU]
Koebnick et al., 2003, Germany (64)	Randomised, double-blind, placebo-controlled, parallel-group	Placebo (*n* = 35; 49%); Fermented intervention (*n* = 35; 60%)	Placebo: 44.6 (18–70); Fermented intervention: 43.3 (18–70)	*Lcb. paracasei* Shirota; 6.5 × 10^9^ CFU/day	Milk	4 weeks	65 mL of placebo ingested every day. Except for the microbes, the placebo and fermented intervention were sensorially (taste, texture, odour) and nutritionally similar. (Good)	Bloating [NEU], Flatulence [NEU], Stool frequency [POS], Stool consistency [POS], Hard stools [POS], Degree of constipation [POS]
Takada et al., 2016, Japan (73)	Randomised, double-blind, placebo-controlled, parallel-group	Placebo (*n* = 70; 45.7%); Fermented intervention (*n* = 70; 45.7%)	Placebo: 22.8 (18–30); Fermented intervention: 23.0 (18–30)	*Lcb. paracasei* Shirota; 1 × 10^11^ CFU/day	Milk	8 weeks	100 mL of placebo once day during intervention period. Placebo nutritionally and ingredient wise similar to fermented intervention. Flavour, texture etc. similar. (Good)	Abdominal symptoms [POS]
Tilley et al., 2014, Belgium (74)	Randomised, double-blind, placebo-controlled, parallel	Placebo (*n* = 56; NI); Fermented intervention (*n* = 50; NI)	Total cohort: 18–65	*Lcb. paracasei* Shirota; 6.5 × 10^9^ CFU/day	Milk	4 weeks	65 mL/day of placebo dosage. All ingredients were identical in the treatment and placebo products, except for the fermenting microbe and were indistinguishable by the subjects or investigators. (Good)	Stool frequency [NEU], (Stool consistency [POS], Stool consistency (BSFS) [POS])

^a^ NI: No information; ^b^CFU/d: colony forming units per day; ^c^ The authors do not report changes. Results are discussed in the text as applicable; ^&^ GSRS: Gastrointestinal symptom rating scale; ^$^ A breakdown among the intervention groups is provided wherever the information is available. ^£^ Comparators were graded for suitability as detailed in the study selection section in Methods; * Outcomes in parentheses were used in meta-analysis. Other outcomes are discussed narratively in text. [POS, NEU, NEG] refer to significantly positive, neutral or negative results, respectively, for the outcome for intervention compared to the control at the end of the intervention period.

Observations and remarks, potential research gaps and subjective EFSA-grade evaluations for this section are summarised in Table 3.

**Table 3:** Evidence, research gaps, and potential EFSA evaluation for the findings in this systematic review.

EFSA evaluation features	Observations and remarks: current evidence and gaps	Potential EFSA evaluation^a^
Identification of pertinent human efficacy studies	Twenty-five studies totalling 4,328 individuals were included from a pool of 5,453 documents retrieved from bibliographic databases in the systematic review. While most of the retrieved studies were RCTs, there were four non-randomised trials, two randomised cross-over trials and one observational study. The number of RCTs was quite low when considered per outcome. For example, the highest number of RCTs were attributed to the stool frequency outcome in meta-analysis (*n* = 9), which did not even allow a publication bias analysis. Ideally, more observational studies would have provided important supportive evidence. Certain populations deemed eligible by EFSA such as those with IBS or functionally constipated were excluded along with articles not in English. Additionally, Embase, a bibliographic database recommended as a minimal requirement by Cochrane for medicine related systematic reviews, was not included. Addition of these criteria can lead to a significant strengthening of the evidence base in an updated meta-analysis.	Neither convincing nor sufficient
Quality and bias of the human studies	One observational trial was assessed as good quality, with three among four non-randomised trials being evaluated as “critical.” Among the 20 RCTs, of both parallel and crossover designs, three studies were deemed “high risk” for the bias due to deviation from the intended intervention and seven studies did not use the appropriate analysis method (intention-to-treat), among others. Although sensitivity analysis revealed that removal of the “high risk” RCTs in the meta-analysis did not change the results for the outcomes analysed, it is deemed a significant concern where the number of studies per outcome are quite low. Broadly, the several studies lacked a statistically rigorous plan and deviated from the intention-to-treat analysis. Future studies should ensure statistical conformity with pre-stated plans, preferably developed in consultation with a biostatistician who remains involved with the trial to the end.	Neither convincing nor sufficient
Relationship between consumption of the fermented food and functional effect	All eligible studies involved healthy study group and were representative of the target demographic. These were almost always free-living subject with some restrictions to dietary intake for RCTs. Meta-analysis of eligible studies in relation to the outcomes of interest revealed some important inferences, however substantial heterogeneity was observed (Figures 2–5) among studies. Substantial variability was observed among comparators for eligible studies ranging from placebos resembling the FF intervention closely (viz. acidified milk) to no intervention consumption and water consumption controls. Such variability can make establishment of causality difficult. Dose–response could not be established in the current analyses as only one study investigated different dosages of FF intervention intake. Extrapolating from that, a lowest dose for eliciting an effect also could not be determined. An effective dose for possible bioactive compounds or probiotic microorganisms were not determined and/or available. The magnitude of effect was not large enough to upgrade the certainty of evidence in GRADE assessment; however, this can be an artefact of the low number of studies for several outcomes. The effects are physiologically relevant, however. The duration of interventions was quite variable ranging from 2–24 weeks. EFSA suggest an intervention duration of at least 4 weeks for foodstuff intervention to ensure that the effects are not influenced by perturbations in the gut. Due to the variability of the intervention duration, whether a sustained effect could be obtained for FF consumption *vis-à-vis* a particular outcome, could not be determined. The amounts of FF used as intervention can be taken up as part of a balanced diet and is reasonable for uptake in the general population. Publication bias can be a factor but could not be determined as none of the outcomes included 10 or more studies, the minimum studies recommended for such an analysis. There were studies where beneficial effects for the outcome were achieved in a shorter intervention duration (2–3 weeks) and with a lower CFU/day than the threshold of 10^10^ CFU/day used in our work. While the former can be attributed as too short a duration to alleviate concerns regarding fluctuations in the gut, an optimal microbial dosage for health benefits remains elusive due to these somewhat contradictory findings. These will require further research expenditure in the future. Moving forward there is a need to create standardised, non-fermented controls for different FFs resembling the FF counterpart as closely as possible. GRADE assessments for the certainty of evidence for most outcomes were determined as “low” or “very low.” This indicates multiple issues in FF trial design including imprecision, risk of bias, and inconsistency, among others. A clinical trial database for FF and standard operating guidelines for such trials may be a good step forward to standardise the ecosystem for such studies.	Neither convincing nor sufficient
Characterisation of the fermented foods and their bioactive compounds	Most products used as FF interventions were incompletely described with little to no information on the fermentation process (type, time, conditions etc.). Other critical information required for a complete evaluation of such products including GMP/HACCP compliance, shelf-life information, storage conditions, batch-to-batch consistency, and sensory properties (or acceptance), chemical or microbiological details (particularly over storage time), were not available. Bioactive compounds were never discussed as potential active components of the fermented food interventions. No information on them was available other than from external literature. Our review also revealed that research into the impact of traditional/artisanal FFs, particularly in non-dairy matrices, is currently limited and studies vertical integration of investigating the impact of a FF intervention to the molecular level (such as correlating with luminal SCFA levels) are scant. These potential research gaps regarding non-dairy FFs, characterisation of potential bioactive compounds, and an absence of manufacturing, transport and storage condition details for the interventions, will need to be resolved in the future.	Neither convincing nor sufficient
Mechanism of action	None of the eligible and included studies elaborated on the mechanisms of action in relation to the outcomes of interest nor were the trials designed in a manner that could elucidate possible mechanisms of action. For example, SCFAs, an important bioactive was never evaluated in the FF interventions. Some level of direct and indirect evidence regarding the possible mechanisms of action (mostly in relation to the potentially probiotic biocomponents of FFs) are however present in literature, but further clarity is required. Studies and trials involving vertically integrated investigations up to the molecular level might be required in the future.	Neither convincing nor sufficient
Bioavailability of bioactive compounds	Bioavailability of relevant bioactive compounds or any factors (e.g., formulation and processing) that could affect their absorption or utilisation in the body were not discussed in any of the eligible studies. Similar to mechanisms of action, some evidence regarding the bioavailable status with regards to the bioactive compounds in the included FF interventions are present in existing literature. However, most of these connect to the outcomes discussed in the review indirectly. Much work is required to gain clarity regarding improved bioavailability of certain bioactive compounds and how they impact outcomes of GI wellbeing. This must be carried in conjunction with experiments and trials investigating the possible mechanisms of action.	Neither convincing nor sufficient
Safety	Adverse effects for the FF interventions were reported in 56% of studies (14/25). While almost half of the eligible studies did not report any adverse effects, no information was usually provided for vulnerable populations, excess consumption amounts, and effects of the fermentation/manufacturing process on the safety of the product, among others. The absence of information as mentioned above should be rectified in future FF trials.	No or very limited evidence

a Represents the 3-step EFSA evaluation of the evidence (Convincing and sufficient; Neither convincing nor sufficient, No or very limited evidence) for each section of conclude the review in the section “Conclusion - Summary of evidence” based on a qualitative evaluation of the main evidence and gaps derived from each of the main sections in Results and Discussion.

### 3.2 Quality and bias of the human studies

**Figure 2:**
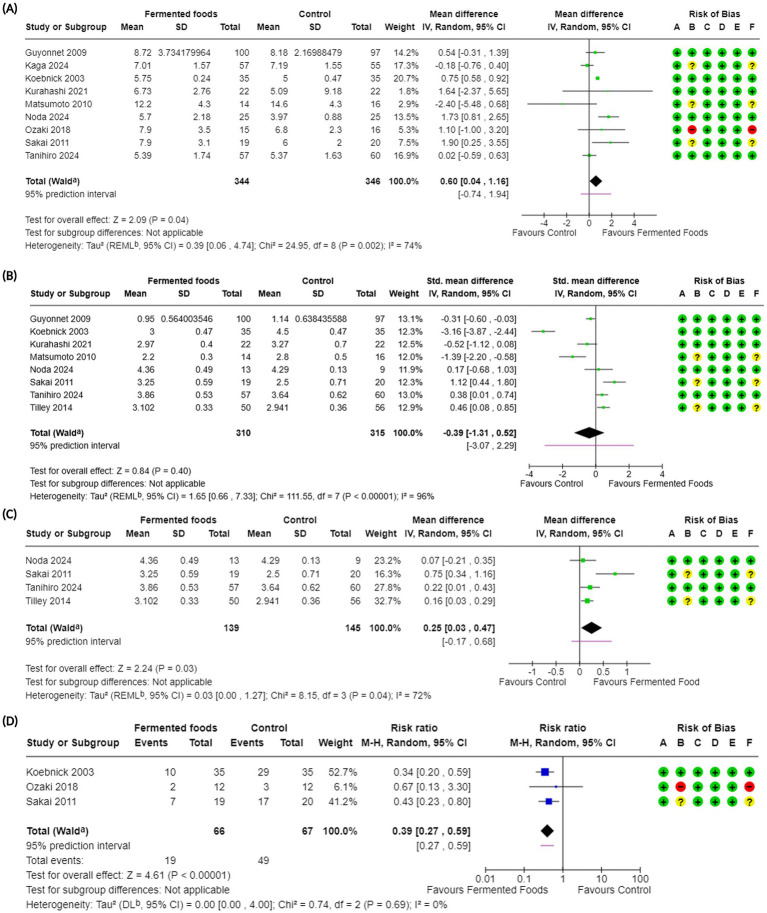
Forest plots of stool frequency and stool consistency in randomised controlled trials comparing fermented foods with control in healthy adults. **(A)** Stool frequency, **(B)** stool consistency, **(C)** stool consistency (only BSFS), and **(D)** incidence of hard stools. Values were calculated as mean difference (95% CIs), standardised mean difference (95% CIs), or risk ratio (95% Cis) using a random-effects model. BSFS, Bristol stool form scale; CI, confidence interval; IV, inverse variance; M-H, Mantel–Haenszel; RR, risk ratio; SD, standard deviation; ^a^ CI calculated by Wald-type method; ^b^ Tau^2^ calculated using the Restricted Maximum-Likelihood method [for (A), (B) and (C)] and the DerSimonian and Laird method (D); Risk of bias legend: (A) bias arising from the randomisation process, (B) bias due to deviations from intended interventions, (C) bias due to missing outcome data, (D) bias in the measurement of the outcome, (E) bias in the selection of the reported result, (F) overall bias.

**Figure 3:**
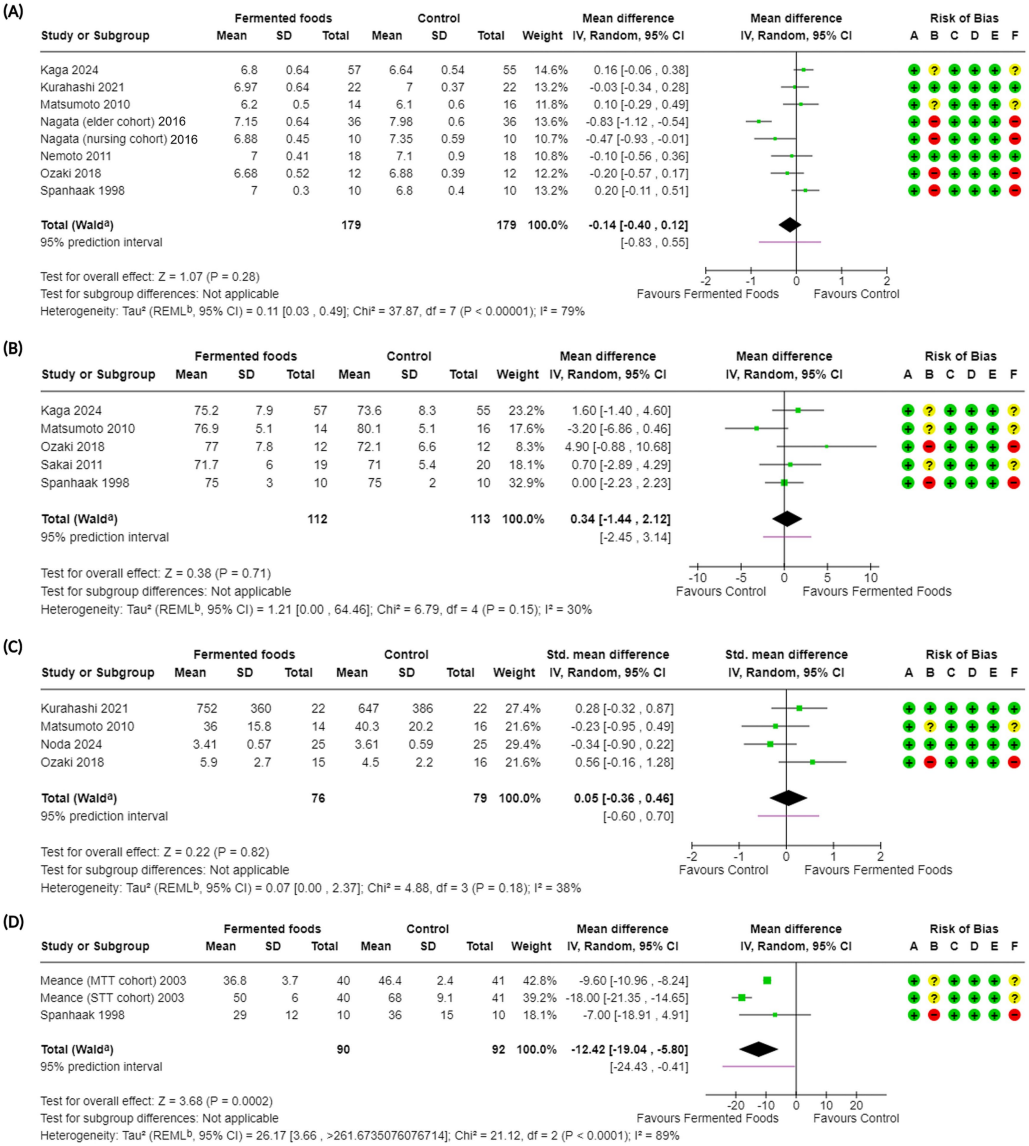
Forest plots of bowel function-related outcomes in randomised controlled trials comparing fermented foods with control in healthy adults. **(A)** Stool pH, **(B)** stool water content, **(C)** stool bulk, and **(D)** intestinal transit time. Values were calculated as mean difference (95% CIs) and standardised mean difference (95% CIs) using a random-effects model. CI, confidence interval; IV, inverse variance; SD, standard deviation; ^a^ CI calculated by Wald-type method; ^b^ Tau^2^ calculated using the Restricted Maximum-Likelihood method; Risk of bias legend: (A) bias arising from the randomization process, (B) bias due to deviations from intended interventions, (C) bias due to missing outcome data, (D) bias in the measurement of the outcome, (E) bias in the selection of the reported result, (F) overall bias.

**Figure 4:**
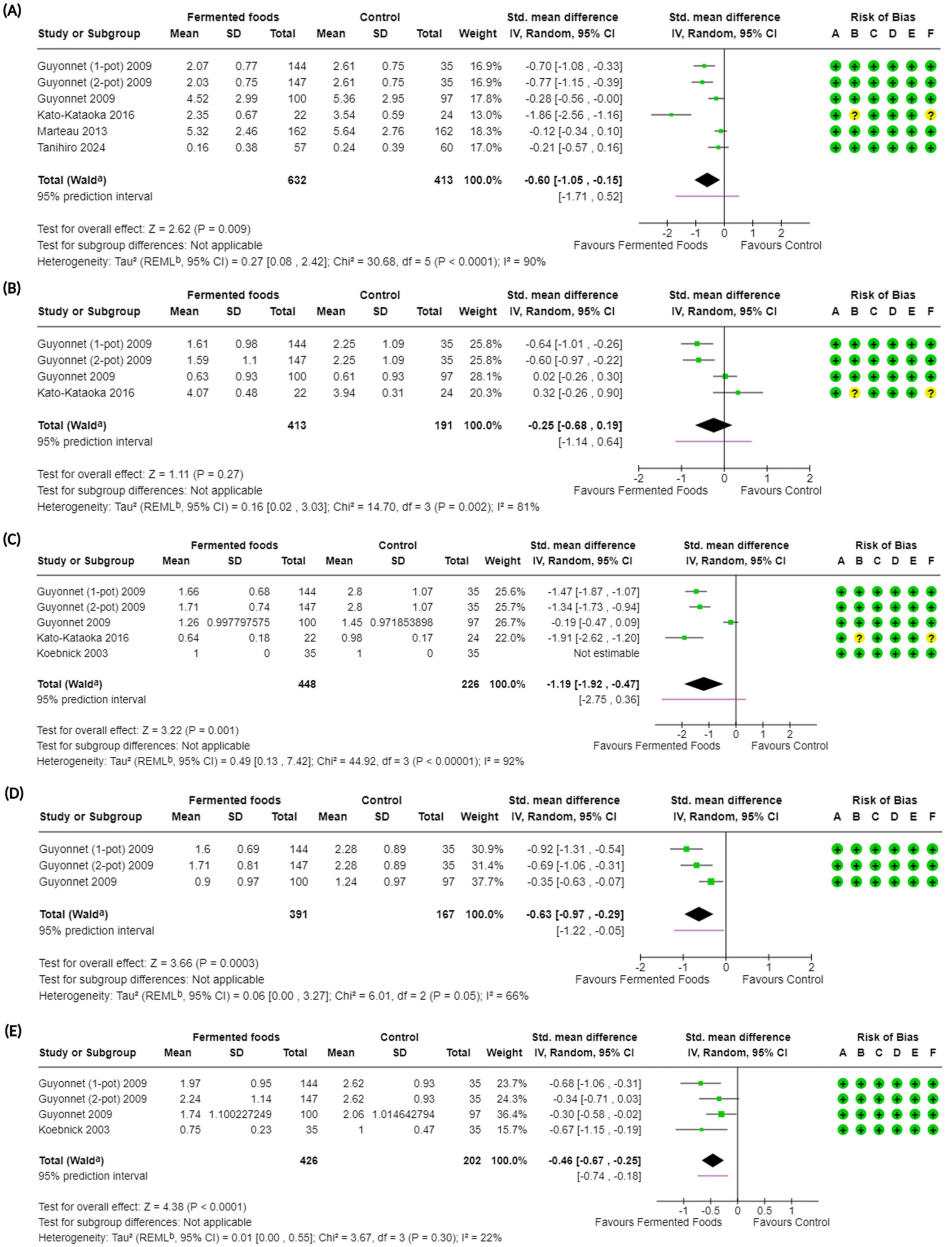
Forest plots of gastrointestinal symptoms in randomised controlled trials comparing fermented foods with control in healthy adults. **(A)** Abdominal symptoms, **(B)** abdominal pain, **(C)** bloating, **(D)** borborygmi and **(E)** flatulence. Values were calculated as standardised mean difference (95% CIs) using a random-effects model. CI, confidence interval; IV, inverse variance; SD, standard deviation; ^a^ CI calculated by Wald-type method; ^b^ Tau^2^ calculated using the Restricted Maximum-Likelihood method; Risk of bias legend: (A) bias arising from the randomization process, (B) bias due to deviations from intended interventions, (C) bias due to missing outcome data, (D) bias in the measurement of the outcome, (E) bias in the selection of the reported result, (F) overall bias.

**Figure 5:**
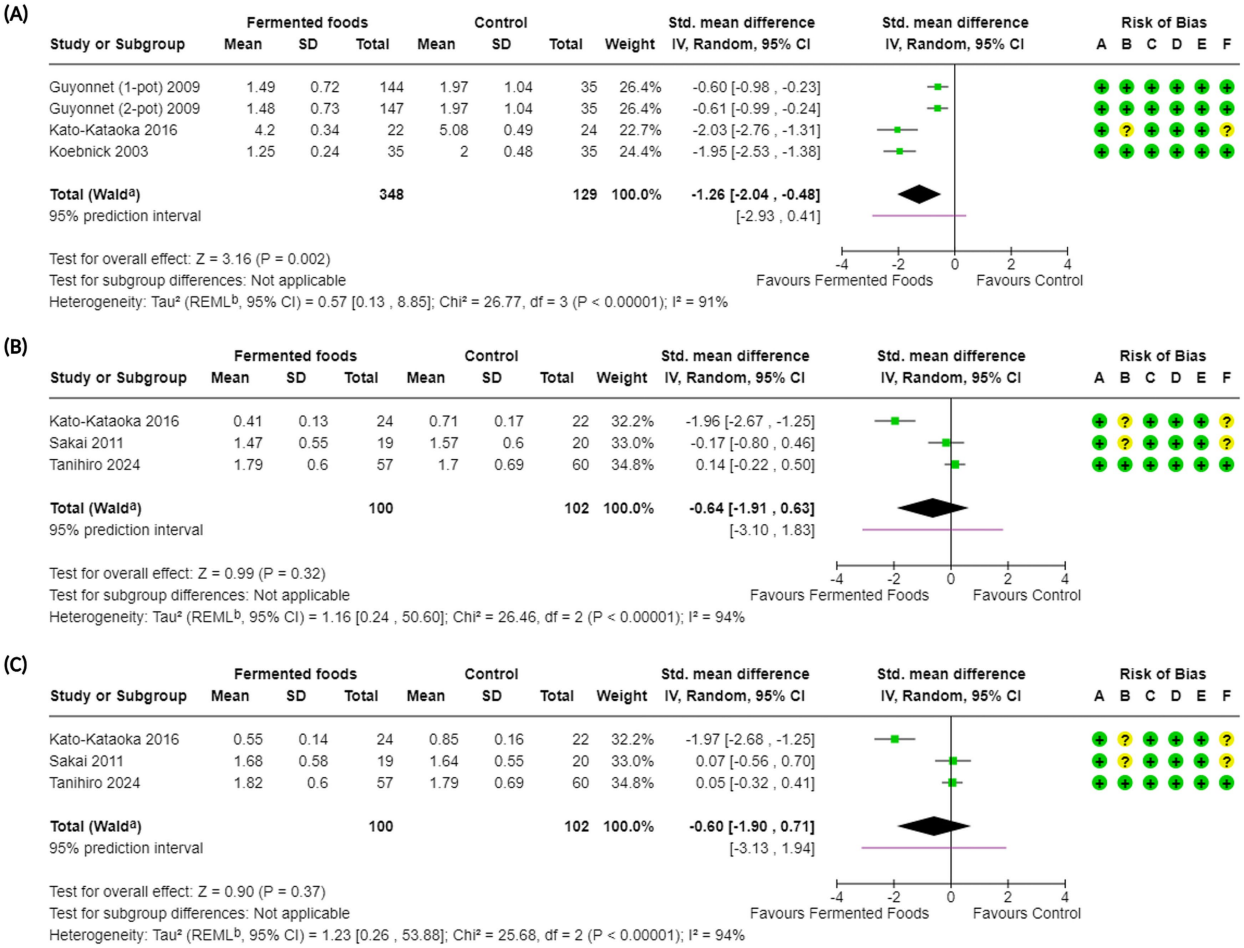
Forest plots of constipation-related symptoms in randomised controlled trials comparing fermented foods with control in healthy adults. **(A)** Degree of constipation, **(B)** feeling of incomplete evacuation, and **(C)** straining during defaecation. Values were calculated as standardised mean difference (95% CIs) using a random-effects model. CI, confidence interval; IV, inverse variance; SD, standard deviation; ^a^CI calculated by Wald-type method; ^b^ Tau^2^ calculated using the Restricted Maximum-Likelihood method; Risk of bias legend: (A) bias arising from the randomisation process, (B) bias due to deviations from intended interventions, (C) bias due to missing outcome data, (D) bias in the measurement of the outcome, (E) bias in the selection of the reported result, (F) overall bias.

As part of the EFSA requirements for evidentiary studies in support of a health claim, we carried out an outcome-related assessment for the risk of bias in each eligible study (Figure 2–5, Supplementary Table S2). Four non-randomised studies (52, 54–56) were evaluated for risk of bias using the ROBINS-I tool. Except for Takii et al., none of the other studies controlled/corrected for confounding and all studies except (52), were evaluated as being at a “critical” risk of bias for all outcomes, with Alves et al., being classified as “moderate risk” (Supplementary Table S2). Twenty randomised controlled trials (of both parallel and crossover design) were evaluated using the Cochrane RoB2 tool (36) for assessing risk of bias in randomised trials. For all randomised trials, bias due to randomisation was “low risk.” Bias due to deviation from the intended intervention (i.e., effect of assignment to intervention) was “low risk” in nine studies (59, 60, 62–68), “some concerns” for eight trials (57, 58, 69–74) and “high risk” for three studies (61, 75, 76). The ratings for this evaluation domain were influenced primarily by the blinding and analysis type of the study (Supplementary Table S2). Among the 20 randomised trials, six studies had an open label design, no blinding, or did not report any blinding (57, 58, 60, 63, 72, 76) with all other studies reporting a double blind design. To evaluate intention-to-treat (ITT) analysis in the studies, an attrition rate of up to 5% was considered acceptable, as advised by Cochrane (36). Using this threshold, seven studies among the 20 randomised trials were identified to have deviated from the desired ITT analysis (57, 58, 61, 69–71, 75) with no information available for one study (76) (Supplementary Table S2). For the two crossover trials (58, 59) among the 20 randomised trials, the risk of bias from period and carryover effects was determined as “low risk.” The Newcastle-Ottawa scale (NOS) for evaluating observational studies was used to determine the risk of bias for the only observational trial (53) included in our study. The cross-sectional study was evaluated as a “Good” quality study as per the Agency for Healthcare Research and Quality standards for NOS evaluation of observational studies (39) with scores of 4 out of a possible 5 in the selection domain, 2/2 for comparability, and 2/3 for the outcome domains (Supplementary Table S2).

Although not directly related to the quality and bias of studies and not a mandatory reporting requirement for EFSA, the degree of compliance to dietary intervention remains an important indicator of adherence and acts as proxy for certain aspects of the quality of a study. Compliance was reported in 11 studies (57, 58, 62, 64, 66–70, 74, 76) with the study by Aslam et al. (77) being ineligible as an observational study (Supplementary Table S5). Among the studies reporting compliance, four did not report a method of determining compliance (67–69, 73) with four others using a daily diary/log maintained for self-reporting by the participants along with the non-used returned interventions to determine compliance (57, 62, 66, 70) and one study determining compliance via interviews every 3 days (along with non-used servings of the intervention) (58) (Supplementary Table S5). Most of the studies that determined compliance reported it as high or satisfactory. For example, compliance rates among intervention and placebo/control groups ranged from 94 to 99.94% across several studies (58, 62, 66–70, 73) (Supplementary Table S5). Galena et al. (57) however reported a lower compliance for the intervention group at 79.3%. Three studies did not provide a quantitative measure with compliance being mentioned as being “good” or “high” (64, 74, 76) (Supplementary Table S5).

Observations and remarks, potential research gaps and subjective EFSA-grade evaluations for this section are summarised in Table 3.

### 3.3 Relationship between consumption of the fermented food and the functional effect

For the purposes of the current work, a health claim for FFs (as is usual in EFSA dossiers) would entail “a potential beneficial physiological effect of the consumption of FFs on healthy adults in relation to the diversity of outcomes outlined in Table 1 or simply on gastrointestinal wellbeing in totality.” Below we have discussed our findings regarding the effect of consumption of FFs on different gastrointestinal outcomes as analysed through meta-analytical methods with some studies excluded from the meta-analysis discussed narratively. Importantly, these outcomes are related to the different physiological benefits as elaborated in Table 1, but are grouped roughly as outcomes related to gastrointestinal (bowel) function, gastrointestinal discomfort and constipation to enable inclusion of all discussed outcomes, some of which may not be mentioned by EFSA as required for a health claim (28). Results of the meta-analysis and GRADE assessment of the outcomes are summarised in Table 4. Subgroup analyses for each outcome on the fermentation matrix type, intervention duration, microbial dosage of the FF intervention and types of fermenting microorganisms are presented in Supplementary Figures S1–S16. Although there is a lack of certainty with respect to the optimal level of consumption of potentially probiotic microbial strains needed to convey health benefits, we chose a cut-off of 10^10^ CFU/day for our subgroup analyses for microbial dosage based on previous reports, including a meta-analysis that revealed a reduced risk for antibiotic-associated diarrhoea at 4 × 10^8^–12 × 10^10^ CFU of *Lacticaseibacillus rhamnosus* GG strain consumed daily (78, 79).

**Table 4:** Results of meta-analysis and certainty of evidence assessments comparing fermented foods with control for outcomes of gastrointestinal/bowel function, abdominal symptoms and constipation in health adults.

Outcomes	Number of studies in meta-analysis (reference)	Results		Heterogeneity	Certainty of evidence^£^	What happens^&^
		Participants (n)	Meta-analysis overall estimate (95% CI)^a^; *p*-value	Chi-square test; *p*-value; I^2^ (%)		
Gastrointestinal/bowel function						
Stool frequency	9 (62, 64, 65, 67–69, 71, 72, 75)	690	MD 0.60 (0.04, 1.16); 0.04*	24.95; 0.002; 74	⊕ ⊕ ⊕◯ Moderate	Consumption of fermented foods likely results in a large increase in stool frequency.
Stool consistency	8 (62, 64, 65, 67, 68, 71, 72, 74)	625	SMD −0.39 (−1.31, 0.52); 0.40	111.55; <0.00001; 96	⊕◯◯◯ Very low	The evidence is very uncertain about the effect of consumption of fermented foods on stool consistency.
Stool consistency (BSFS)^$^	4 (67, 68, 72, 74)	284	MD 0.25 (0.03, 0.47); 0.03*	8.15; 0.04; 72	⊕ ⊕ ◯◯ Low	The evidence suggests that consumption of fermented foods results in a slight increase in stool consistency (BSFS).
Hard stools	3 (64, 72, 75)	133	RR 0.39 (0.27, 0.59); <0.00001*	0.74; 0.69; 0	⊕ ⊕ ⊕◯ Moderate	Consumption of fermented foods likely results in a large reduction in hard stools.
Stool bulk	4 (65, 67, 71, 75)	155	SMD 0.05 (−0.36, 0.46); 0.82	4.88; 0.18; 38	⊕◯◯◯ Very low	The evidence is very uncertain about the effect of consumption of fermented foods on stool bulk.
Stool pH	7^b^ (59, 61, 65, 69, 71, 75, 76)	358	MD −0.14 (−0.40, 0.12); 0.28	37.87; <0.00001; 79	⊕◯◯◯ Very low	The evidence is very uncertain about the effect of consumption of fermented foods on stool pH.
Stool water content	5 (69, 71, 72, 75, 76)	225	MD 0.34 (−1.44, 2.12); 0.71	6.79; 0.15; 30	⊕◯◯◯ Very low	The evidence is very uncertain about the effect of consumption of fermented foods on stool water content.
Intestinal transit time	2^c^ (58, 76)	182	MD −12.42 (−19.04, −5.80); 0.0002*	21.12; <0.00001; 89	⊕◯◯◯ Very low	The evidence is very uncertain about the effect of consumption of fermented foods on intestinal transit time.
Gastrointestinal symptoms						
Abdominal symptoms	5^d^ (60, 62, 66, 68, 70)	1,045	SMD −0.60 (−1.05, −0.15); 0.009*	30.68; <0.0001; 90	⊕ ⊕ ◯◯ Low	Consumption of fermented foods may result in a reduction in abdominal symptoms.
Abdominal pain	3^b^ (60, 62, 70)	604	SMD −0.25 (−0.68, 0.19); 0.27	14.70; 0.002; 81	⊕◯◯◯ Very low	The evidence is very uncertain about the effect of consumption of fermented foods on abdominal pain
Bloating	4^b^ (60, 62, 64, 70)	674	SMD −1.19 (−1.92, −0.47); 0.001*	44.92; <0.00001; 92	⊕ ⊕ ◯◯ Low	Consumption of fermented foods may result in a large reduction in bloating.
Borborygmi	2^b^ (60, 62)	558	SMD −0.63 (−0.97, −0.29); 0.0003*	6.01; 0.05; 66	⊕ ⊕ ⊕◯ Moderate	Consumption of fermented foods likely results in a reduction in borborygmi.
Flatulence	3^b^ (60, 62, 64)	628	SMD −0.46 (−0.67, −0.25); <0.0001*	3.67; 0.30; 22	⊕ ⊕ ⊕⊕ High	Consumption of fermented foods results in a slight reduction in flatulence.
Constipation-related symptoms						
Degree of constipation	3^b^ (60, 64, 70)	477	SMD -1.26 (−2.04, −0.48); 0.002*	26.77; <0.00001; 91	⊕ ⊕ ◯◯ Low	Consumption of fermented foods may result in a large reduction in degree of constipation.
Feeling of incomplete evacuation	3 (68, 70, 72)	202	SMD −0.64 (−1.91, 0.63); 0.32	26.46; <0.00001; 94	⊕◯◯◯ Very low	The evidence is very uncertain about the effect of consumption of fermented foods on feeling of incomplete evacuation.
Straining during defaecation	3 (68, 70, 72)	202	SMD −0.60 (−1.90, 0.71); 0.37	25.68; <0.00001; 94	⊕◯◯◯ Very low	The evidence is very uncertain about the effect of consumption of fermented foods on straining during defaecation.

^$^ BSFS: Bristol Stool Form Scale; ^£^ Certainty of evidence was assessed using the GRADE approach and carried out in GRADEpro GDT; ^&^ Provides a narrative estimation of the effect of the intervention; ^a^ Meta-analysis was carried out using a random effects model. Statistical heterogeneity was estimated using the chi-square test and quantified using the I^2^ statistic and Tau^2^ (restricted maximum-likelihood method). *p*-values with asterisks represent statistically significant results (*p* < 0.05); ^b^ Analysis includes one study that has two separate entries for different cohorts (61); ^c^ Analysis includes one study that has two separate entries for different cohorts (58); ^d^ Analysis includes one study that has two separate entries for different doses (60); MD, mean difference; RR, Risk ratio; SMD, standardised mean difference.

#### 3.3.1 Gastrointestinal (bowel) function

##### 3.3.1.1 Stool frequency

Stool frequency was one of the primary outcomes investigated in this meta-analysis. This choice was made to reflect the central phenotypic nature of stool frequency in gastrointestinal wellbeing in everyday life. For our work, to best reflect the general demographic, individuals with diagnosed constipation were excluded, with only studies involving participants with self-diagnosed/mild constipation included along with healthy individuals (Tables 1, 2). This is also deliberate and is aimed at reflecting the real and current world, where mild constipation due to various lifestyle factors is not uncommon nowadays. *In toto*, 14 studies reported the outcome of stool frequency (54–56, 59, 62, 64, 65, 67–69, 71, 72, 74, 75) with nine included in the meta-analysis (Table 2, 4). Across 14 studies, the outcome was investigated in 912 participants with 690 individuals included in the meta-analysis (Figure 2, Tables 2, 4). Studies reported stool frequency in a diversity of units such as number of evacuations/week, number of evacuations/day, and number of days with bowel movement/week, among others; units were converted as required with only one unit from a study reporting two or more units used. Overall, FFs consumption exhibited a positive impact on stool frequency compared to control (MD 0.60, CI 0.04, 1.16, *p* = 0.04) with included studies showing a moderate level of heterogeneity (I^2^ = 74%, *p* = 0.002) (Figure 2, Table 4). Subgroup analyses on stool frequency for the fermentation matrix revealed a beneficial impact for dairy and sericin-fibroin matrices (Supplementary Figure S1A). Further subgroup analyses involving intervention duration and microbial dosage revealed a beneficial impact at 3 and 12 weeks and at a dosage of < 10^10^ CFU/day, respectively, (Supplementary Figures S1B,C). When analysed for fermenting microbes, a subgroup analysis revealed a clear benefit for only *Lactococcus lactis* BM32 strain (MD 1.73, CI 0.81, 2.65, *p* = 0.0002) (Supplementary Figure S1D). A sensitivity analysis was carried out for the meta-analysis by removing the only high risk of bias study, which interestingly changed the above conclusion to one of no effect for FFs consumption on stool frequency (MD 0.57, CI −0.03, 1.17, *p* = 0.06, I^2^ = 78%, *p* = 0.0008) (Supplementary Figure S17A). The certainty of evidence for the outcome was carried out using the GRADE assessment approach which downgraded it to a “moderate” certainty of evidence due to the moderate heterogeneity observed among the studies included in the meta-analysis (Table 4). Besides the studies involved in the meta-analysis, five other studies reported the outcome of stool frequency (54–56, 59, 74). Among these, three were not included in the meta-analysis as they were not randomised (54–56), while the others did not provide the results data in a format usable for meta-analysis (59, 74). Across these studies, a variety of fermented interventions were employed including fermented whey, milk, *Brassica rapa L.*, and brown rice, where only Tanaka et al. reported a significantly positive (or beneficial) impact on stool frequency, with the rest reporting a neutral result for the outcome (Table 2).

##### 3.3.1.2 Stool consistency

Stool consistency was investigated as one of the primary outcomes of bowel function and in turn gastrointestinal wellbeing in the present work. Hard, lumpy stools that are difficult to evacuate and are an atypical phenotype in normal populations, is an indicator of possible constipation and reduced GI wellbeing (79). Indeed, stool consistency is central to a typical, healthy lifestyle and GI wellbeing, and is therefore commonly reported as an outcome in food-based interventions. Stool consistency was presented across studies in the form of the Bristol Stool Form Scale (BSFS), other modified scales based on the BSFS or simply as incidence of hard stools, all indicative of stool consistency (Table 2). We investigated each separately to understand the breadth of evidence for FFs consumption in relation to stool consistency. Stool consistency as an outcome was reported in 11 studies (56, 57, 59, 62, 64, 65, 67, 68, 71, 72, 74), including both BSFS and BSFS-based modified scales, with eight being involved in the meta-analysis (Figure 2, Table 4). These studies involved a total of 701 participants among which 615 were included in the meta-analysis (Figure 2, Tables 2, 4). Overall, FFs consumption did not seem to beneficially impact stool consistency compared to control (SMD −0.39, CI −1.31, 0.52, *p* = 0.40) with a high degree of heterogeneity among studies (I^2^ = 96%, *p* < 0.00001) (Figure 2, Table 4). Subgroup analyses for this outcome were generally uninformative (Supplementary Figures S2A–D). Opposing results were observed when considering the subgroup analyses for fermenting microbes: while a beneficial effect on stool consistency could be seen for milk fermented with *Lactobacillus helveticus* CP790, results for a milk fermented with a mixture of *Bifidobacterium lactis*, *Lactococcus cremoris*, *Streptococcus thermophilus* and *Lactobacillus delbrueckii* subsp. *bulgaricus* (*L. bulgaricus*) favoured the control (Supplementary Figure S2D). Certainty of evidence for stool consistency was downgraded to “very low” during GRADE assessment due to the high level of heterogeneity among studies and imprecision (Table 4). Besides the RCTs involved in the meta-analysis, a few other studies reported the outcome of stool consistency. All these studies involved non-dairy fermented products with a fermented brown rice used as intervention by Nemoto et al. (59), fermented vegetables by Galena et al. (57) and fermented *B. rapa* by Tanaka et al. (56). None of the studies reported a significantly positive impact for the consumption of the fermented intervention on stool consistency (Table 2).

Given the high level of heterogeneity in the meta-analysis with all stool consistency results, we further investigated the outcome with studies reporting stool consistency only using the BSFS scale to ensure a uniform starting point. Stool consistency using the BSFS scale was reported in four studies (67, 68, 72, 74), all of which were included in the meta-analysis and spanned a total of 284 participants (Figure 2, Table 4). Overall, when considering BSFS-based stool consistency as an outcome, FFs consumption exhibited an improvement compared to control (MD 0.25, CI 0.03, 0.47, *p* = 0.03) with moderate heterogeneity among the studies (I^2^ = 72%, *p* = 0.04) (Figure 2). Subgroup analyses of the meta-analyses revealed interesting inferences (Supplementary Figures S3A–D). For example, a beneficial effect for BSFS-based stool consistency was seen only for fermented dairy (MD 0.33, CI 0.01, 0.64, p = 0.04), although the subgroup heterogeneity was high (I^2^ = 83%, *p* = 0.02) (Supplementary Figure S3A). Additionally, a positive effect was also reported for a microbial dosage of ≥ 10^10^ CFU/day (MD 0.17, CI 0.00, 0.33, *p* = 0.05, I^2^ = 0%, *p* = 0.4) and for fermentation with *L. helveticus* CP790 (MD 0.22, CI 0.01, 0.34, p = 0.04) (Supplementary Figures S3C,D). Certainty of evidence for this outcome was downgraded to “low” using GRADE assessment given the moderate heterogeneity among the reported studies and the lower number of total participants in the meta-analysis (Figure 2, Table 4).

The difference in results for the meta-analyses involving all stool consistency-based results and BSFS-based results only encouraged us to further investigate the outcome reported in slightly different terms in certain studies. Indeed, in some studies stool consistency was also provided indirectly in terms of a dichotomous incidence of hard stools in the intervention and control groups where a reduction of incidence indicated a positive outcome (Table 2). This outcome was reported in five studies (54, 55, 64, 72, 75) with three studies involved in the meta-analysis (Figure 2, Table 4). In terms of incidence of hard stools, FFs consumption exhibited a clear benefit compared to control (RR 0.39, CI 0.27, 0.59, *p* < 0.00001) with a possible low reported heterogeneity (I^2^ = 0%, *p* = 0.69) (Figure 2, Table 4). Subgroup analyses for the outcome revealed benefits on consumption of FFs irrespective of intervention duration, for a microbial dosage lower than 10^10^ CFU/day (although there was only one study with a dosage of ≥ 10^10^ CFU/day) and for fermentation with *Lcb. casei* Shirota (Supplementary Figures S4A–D). A sensitivity analysis where the single high risk of bias study was removed did not change the inference with consumption of FFs still exhibiting a benefit compared to control (RR 0.38, CI 0.25, 0.57, *p* < 0.0001, I^2^ = 0%, *p* = 0.58) (Supplementary Figure S17B). Apart from the RCTs included in the meta-analysis, incidence of hard stools was also reported by Ling et al. (54) and Takii et al. (55). The former involved an intervention with a fermented whey drink and the latter with fermented turnips, with both returning a significantly positive impact for their consumption compared to control (or baseline) for the incidence of hard stools, i.e., a reduction in incidence (Table 2).

##### 3.3.1.3 Stool pH and water content

While not considered primary outcomes in this work, stool pH and stool water content were investigated due to their potential indicative role in gut health and constipation-like symptoms. A gut pH of 5.5–7.0 (average of ~6.6) is considered healthy, associated with an unperturbed gut microbiota, with deviations resulting from various factors including stress, improper diet, lack of exercise, low-grade inflammation in the gut, medications, and a dysbiotic gut microbiota, among others (80). Interestingly, deviations in stool pH are also linked to constipation (81, 82). In contrast, stool water content is directly related to constipation, where normal stool consists of ~75% water. Excessive colonic absorption of water from the stool as it moves through can produce hard, dry stool difficult to evacuate (83). Similar to stool pH, a reduced water content can be brought about by lifestyle factors and improper diets reduced in dietary fibres, among others.

Stool pH was reported in nine studies (54, 59, 61, 65, 69, 71, 75, 76) with eight of them included in a meta-analysis totalling 364 participants (358 in meta-analysis) (Table 4). Overall, FFs had no impact on stool pH compared to control (MD −0.14, 95% CI −0.40, 0.12, *p* = 0.28, I^2^ = 79%, *p* < 0.00001). Subgroup analyses showed that the fermenting matrix of the FF and fermenting microorganisms did not impact the stool pH (Supplementary Figures S6A,D). However, when analysed for intervention duration, a longer intervention duration of 24 weeks improved stool pH compared to control (MD −0.70, CI −1.04, −0.36, *p* < 0.001) with low heterogeneity (I^2^ = 41%, *p* = 0.19) (Supplementary Figure S6B). Sensitivity analysis carried out by removal of the high risk of bias RCTs from the meta-analysis did not produce a change in the results although heterogeneity was substantially lower (MD 0.07, CI −0.08, 0.23, *p* = 0.34, I^2^ = 0%, *p* = 0.66) (Supplementary Figure S17C). GRADE assessment of the evidence for stool pH was downgraded to a “very low” certainty of evidence due to a low number of total participants, high heterogeneity and high risk of bias (Table 4). One non-randomised study not included in the meta-analysis (54) reported a slight decrease in the stool pH after the intervention with *Lb*. *rhamnosus* GG fermented whey drink.

Stool water content was reported in six studies (59, 69, 71, 72, 75, 76) with five being included in meta-analysis involving 26 participants in total (225 included in meta-analysis). Similar to stool pH, meta-analysis showed that consumption of FFs did not impact the stool water content (MD 0.34, CI −1.44, 2.12, *p* = 0.71) although heterogeneity was low (I^2^ = 30%, *p* = 0.15) (Table 4). Subgroup analyses for stool water content did not provide any additional insights into other factors that may be influencing the outcome (Supplementary Figures S7A–D). Sensitivity analysis carried out for this meta-analysis by removal of high risk of bias studies did not change the overall inference and retained moderate heterogeneity (MD −0.18, CI −3.02, 2.67, *p* = 0.90, I^2^ = 52%, *p* = 0.12) (Supplementary Figure S17D). Similar to stool pH, GRADE assessment for stool water content rated the evidence at a “very low” certainty due to the high risk of bias in 2 studies and a low number of participants in the meta-analysis (Table 4). Nemoto et al. (59) reported no changes in the stool water content for intervention and control groups in their RCoT; this was mentioned only narratively and therefore could not be included in the meta-analysis.

##### 3.3.1.4 Stool bulk

Stool bulk (or total amount of faeces) was also investigated as an indirect outcome related to bowel function. Along with its frequency and consistency, stool bulk is a good indicator of gut health in the general population. Healthy stool is typically a medium to dark brown, soft to semi-firm, and easy to pass, usually between three times a week and three times a day, with deviations leading to reduced GI wellbeing. In constipated or otherwise afflicted individuals, stool bulk is reduced (in individual passages and with overall fewer bowel movements); a diet rich in fibres is crucial for adding bulk to stool, making it easier to pass and reducing the risk of constipation (79, 84). Stool bulk was reported as an outcome in six studies (54, 59, 65, 67, 71, 75) with four of them being included in the meta-analysis (Table 2, Figure 3). In total, the outcome was investigated in 197 individuals among which 155 were included for meta-analysis (Figure 3, Table 2). Overall, FFs consumption did not have a beneficial impact on stool bulk compared to control (SMD 0.05, CI −0.36, 0.46, *p* = 0.82) with low level of heterogeneity seen among studies (I^2^ = 38%, *p* = 0.18) (Figure 3). Subgroup analyses for the outcome did not reveal any specific factor contributing to the outcome (Supplementary Figures S5A–D). Sensitivity analysis carried out for the outcome where one high risk of bias study was removed did not change the inference (SMD −0.09, CI −0.49, 0.31, *p* = 0.65, I^2^ = 21%, *p* = 0.31) (Supplementary Figure S17E). GRADE assessment for the certainty of evidence for this outcome was downgraded to “very low” due to the high risk of bias, low participant numbers and imprecision (Table 4). Finally, Ling et al. (54), a non-randomised study, and Nemoto et al. (59), used fermented whey and fermented brown rice as interventions, respectively, to investigate their effect on stool bulk and reported no impact on the same after the intervention period (Table 2).

##### 3.3.1.5 Intestinal transit time

We also investigated whether FFs consumption has any impact on intestinal transit time given its close association with bowel movement, stool consistency and general GI wellbeing. Intestinal transit time, which is the time required for food to travel through the digestive tract, is normally 30–40 h with a slower transit being linked to constipation where excessive water is absorbed during the transit, resulting in hard, dry stools and, in turn, fewer bowel movements and difficulty in evacuation, among others (85). Intestinal transit time was reported in two studies, both of which were included in the meta-analysis, spanning 182 individuals in total (Figure 3, Tables 2, 4). Overall, FFs consumption had a positive impact on intestinal transit time compared to control (MD −12.42 CI −19.04, −5.80, *p* = 0.0002) with studies showing substantial heterogeneity (I^2^ = 89%, *p* < 0.0001) (Figure 3, Table 4). Subgroup analyses revealed benefits to be associated with an intervention duration of two weeks and with a mixture of fermenting microbes comprising of *B. lactis*, *S. thermophilus* and *L. bulgaricus*; heterogeneity remained high between subgroups (Supplementary Figures S8A–D). Sensitivity analysis was also carried out for the outcome with removal of one study with a high risk of bias; this did not change the previous inference of a beneficial impact by FFs on intestinal transit time (MD −13.65 CI −21.88, −5.43, *p* = 0.001, I^2^ = 95%, *p* < 0.001) (Supplementary Figure S17F). The certainty of evidence for this outcome was downgraded to a “very low” mark due to the higher risk of bias, low participant count and high heterogeneity (Table 4).

#### 3.3.2 Gastrointestinal symptoms

A diversity of outcomes was considered to understand the impact of FF consumption on GI (or abdominal) symptoms that would be experienced in the general population daily (Tables 1, 4). These included severity of total GI (or abdominal) symptoms, abdominal pain, bloating, borborygmi (rumbling in the abdomen) and flatulence. GI wellbeing was measured using questionnaires in a few studies; however, other than one study by Guyonnet et al. (60) who reported an improvement of GI wellbeing upon consumption of fermented milk, the results in other studies were not usable for this review. The severity of abdominal symptoms was reported in a total of seven studies (60, 62, 63, 66, 68, 70, 73) among which five were included in the meta-analysis with two studies presenting data in a format not usable for meta-analysis (63, 73). Across seven studies, severity of abdominal symptoms was investigated in 2,146 participants from which 1,045 were included in the meta-analysis (Table 2, Figure 4). Overall, consumption of FFs had a beneficial effect on severity of abdominal symptoms compared to control (SMD −0.60, CI −1.05, −0.15, *p* = 0.009) with a high degree of heterogeneity among studies (I^2^ = 90%, *p* < 0.0001) (Figure 5). Subgroup analyses for abdominal symptoms revealed that benefits can be affected by consumption of FFs across a range of intervention durations (2–8 weeks) with a longer duration of 8 weeks contributing to more significant positive impact (or reduction) on abdominal symptoms (SMD −1.86, CI −2.56, −1.16, *p* < 0.00001) (Supplementary Figure S9B). Subgroup analyses additionally revealed that fermenting microorganisms mixtures containing *B. lactis*, *S. thermophilus* and *L. bulgaricus* along with *Lcb*. *casei* Shirota were more effective in reducing abdominal symptoms compared to other fermenting microbes (Supplementary Figure S9D). GRADE assessment of the outcome downgraded the certainty of evidence to “low” primarily due to the significant heterogeneity in the studies (Table 4). Beyond the RCTs included in the meta-analysis, studies by Kinoshita et al. and Takada et al. (63, 73) respectively reported a neutral and significantly positive effect for the consumption of fermented milk on abdominal symptoms (Table 2).

Severity of abdominal pain (or simply abdominal pain) was reported in six studies (52, 57, 60, 62, 63, 70) among which three were included in a meta-analysis (Table 4, Figure 4). Across six studies, 1,637 participants were involved in the investigation for this outcome with 604 participants included meta-analysis (Table 2, Figure 4). Overall, consumption of FFs did not have a beneficial effect on abdominal pain compared to control (SMD −0.25, CI −0.68, 0.19, *p* = 0.27) with the studies showing considerable heterogeneity (I^2^ = 81%, *p* = 0.002) (Figure 4, Table 4). Subgroup analyses for this outcome revealed a beneficial effect for FF consumption on abdominal pain for only the shortest duration of intervention, i.e., 2 weeks (Supplementary Figure S10B) as well as for the mix of fermenting microbes containing *B. lactis*, *S. thermophilus* and *L. bulgaricus* (Supplementary Figure S10D). GRADE assessment for the outcome was downgraded to a “very low” certainty of evidence due to high heterogeneity among studies as well as imprecision (Table 4). Severity of abdominal pain was also reported in three other studies, with either non-randomised designs or data in non-usable formats for meta-analysis. Among these, Galena et al. (57) did not report any significant difference between FF consumption and control, Kinoshita et al. (63) reported a neutral outcome while Alves et al. (52) reported an improvement in severity of abdominal pain.

A third gastrointestinal outcome considered for our investigation was bloating. Degree of bloating (or simply bloating) was reported in six studies with four included in the meta-analysis (52, 57, 60, 62, 64, 70) (Table 2). In total, the outcome was investigated in 1,707 participants with 674 individuals included in the meta-analysis (Figure 4, Table 2). Overall, FF consumption had a positive effect on the degree of bloating compared to control with a decrease in the summary measure (SMD −1.19, CI −1.92, −0.47, *p* = 0.001); a high degree of heterogeneity was observed (I^2^ = 92%, *p* < 0.00001) (Figure 4, Table 4). In terms of subgroup analyses for this outcome (Supplementary Figures S11A–D), a positive impact for consumption of FFs on bloating was revealed for interventions of durations of 2 and 8 weeks (Supplementary Figure S11B) as well as for the mix of fermenting microbes containing *Bifidobacterium lactis*, *S. thermophilus* and *L. bulgaricus* (Supplementary Figure S11D). GRADE assessment downgraded the certainty of evidence for this outcome to “low” due to the high level of heterogeneity in the studies (Figure 4, Table 4). Among the studies that were not included in the meta-analysis, Galena et al. (57) did not report any significant impact of FFs consumption on bloating while Alves et al. (52) reported a significantly positive impact.

Borborygmi (or rumbling in the stomach) was the fourth gastrointestinal symptom that was investigated in this study. The outcome was reported in two studies, both included in the meta-analysis, spanning 558 individuals (60, 62) (Table 2, Figure 4). Overall, FFs consumption had a beneficial effect on the severity of borborygmi compared to control (SMD −0.63, CI −0.97, −0.29, *p* = 0.0003) with studies showing moderate heterogeneity (I^2^ = 66%, *p* = 0.05) (Figure 4). Subgroup analyses for the outcome revealed a positive impact of FFs consumption on the severity of bloating irrespective of intervention duration and fermenting microbes; microbial dosage and fermentation matrix subgroup analyses were uninformative (Supplementary Figures S12A–D). GRADE assessment marked the certainty of evidence for borborygmi as “moderate” with the only downgrade because of moderate heterogeneity (Table 4).

The fifth and final gastrointestinal symptom examined for our work was the degree of flatulence. To be noted, the outcome flatulence should not be confused with intestinal gas accumulation, which would be an objective metric and would be measured differently (28). Flatulence was reported in four studies as an outcome (52, 60, 62, 64) with three being included in the meta-analysis and one non-randomised study being excluded (Tables 2, 4). Across studies, the outcome was reported for 680 individuals with 628 participants being included in the meta-analysis (Tables 2, 4, Figure 4). Overall, FFs consumption showed a significant improvement (or reduction) in the degree of flatulence as compared to control (SMD −0.46, CI −0.67, −0.25, *p* < 0.0001) with studies exhibiting a low heterogeneity (I^2^ = 22%, *p* = 0.3) (Figure 4). Interestingly, subgroup analyses revealed that FFs consumption reduced flatulence across all intervention durations, microbial dosages and types of fermentation microbes (Supplementary Figures S13A–D). GRADE assessment for the certainty of evidence regarding flatulence was marked “high” due to its low heterogeneity, risk of bias, indirectness and high precision (Table 4). Alves et al. (52), which was not included in the meta-analysis, reported a neutral outcome for the consumption of milk kefir against control in relation to flatulence (Table 2).

#### 3.3.3 Constipation and related symptoms

To understand if consumption of FFs has an impact on constipation-related symptoms, a few different outcomes were considered (Tables 2, 4). The first outcome we investigated was the incidence of constipation reported in participants consuming FFs compared to control. This was addressed in two studies, including one RCT and one observational study (61, 77). In the observational study by Aslam et al., no association was reported between the consumption of fermented dairy, such as cheese and yogurt, and constipation for both men (*n* = 609) and women (*n* = 632) of the Geelong osteoporosis cohort (77) (Table 2). In the RCT performed by Nagata et al. however, *Lcb. casei* strain Shirota fermented milk was reported to significantly reduce the incidence of constipation after the intervention period (61) (Table 2).

The second constipation-related outcome considered was the degree (or severity) of constipation. Degree of constipation was reported in five studies (52, 60, 63, 64, 70) with three studies used eventually in the meta-analysis (Figure 5, Table 4). In total, the degree of constipation was investigated in 1,490 individuals across all studies with 477 involved in the meta-analysis (Tables 2, 4, Figure 5). Overall, consumption of FFs had a beneficial effect on the degree of constipation compared to control with a summary decline in its severity (SMD −1.26, CI −2.04, −0.48, *p* = 0.002) although heterogeneity was high (I^2^ = 91%, *p* < 0.00001) (Figure 5, Table 4). Subgroup analyses for degree of constipation revealed that benefits could be observed irrespective of intervention duration, microbial dosage or the fermentation microorganism, at least for the reported studies (Supplementary Figures S14A–D). An indicative example would be microbial dosage, where doses of < 10^10^ CFU/day (SMD −1.95, CI −2.53, −1.38, *p* < 0.00001) and ≥ 10^10^ CFU/day (SMD −1.03, CI −1.91, −0.15, *p* = 0.02) showed no difference in providing a positive effect for consumption of FFs on the degree of constipation (Supplementary Figure S14C). GRADE assessment for certainty of the evidence was however downgraded to “low” due to the high level of heterogeneity observed among studies (Table 4). Apart from the RCTs involved in the meta-analysis a non-randomised, controlled study by Alves et al. (52) reported a significantly beneficial effect on the degree of constipation due to the consumption of milk kefir. Additionally, Kinoshita et al. (63) also reported a significantly beneficial effect on the degree of constipation from the consumption of milk fermented by *L. bulgaricus* OLL1073R-1, although the data was not in a format that could be used in the meta-analysis.

The third outcome we investigated concerning constipation was the feeling of incomplete evacuation. The outcome was reported in four studies (55, 68, 70, 72), with three being included in the meta-analysis (Figure 5, Table 4). Overall, the outcome was investigated in 256 individuals, with 202 included in the meta-analysis (Figure 5, Table 4). Meta-analysis of effect measures did not indicate a benefit in the feeling of incomplete evacuation for consumption of FFs compared to control (SMD −0.64, CI −1.91, 0.63, *p* = 0.32), with the heterogeneity being very high (I^2^ = 94%, *p* < 0.0001) (Figure 5, Table 4). Subgroup analyses for the outcome revealed that an intervention duration of 8 weeks provided a beneficial impact compared to interventions of shorter durations (3–4 weeks) (SMD −1.96, CI −2.67, −1.25, *p* < 0.00001) (Supplementary Figure S15B); other subgroup analyses were not informative (Supplementary Figures S15A–D). A GRADE assessment of the evidence downgraded the certainty to “very low” based on the considerable heterogeneity of studies as well as the low number of participants (Table 4). Apart from the RCTs involved in the meta-analysis, a non-randomised, controlled study by Takii et al. (55) reported a significantly positive impact for the consumption of *Levilactobacillus brevis* NSB2 fermented turnips on the feeling of incomplete evacuation compared to control (Table 2).

The fourth and final outcome investigated *vis-à-vis* constipation-related symptoms was straining during defaecation. The outcome was reported in three studies with 202 participants in total, all of whom were included in the meta-analysis (68, 70, 72) (Table 4). Overall, our meta-analysis indicated no beneficial effect of the consumption of FFs compared to control on straining during defaecation (SMD −0.60, CI −1.90, 0.71, *p* = 0.37) where heterogeneity among the studies was very high (I^2^ = 94%, *p* < 0.00001) (Figure 5, Table 4). Similar to the feeling of incomplete evacuation, subgroup analyses indicated a potential benefit for longer intervention durations, i.e., 8 weeks, for the outcome compared to shorter ones (SMD −1.97, CI −2.68, −1.25, *p* < 0.00001) (Supplementary Figure 16B); other subgroup analyses were not informative (Supplementary Figures S16A–D). Again, similar to the feeling of incomplete evacuation, the GRADE assessment for straining during defaecation was marked as a “very low” certainty of evidence due primarily to the considerable heterogeneity of studies as well as the low number of participants (Table 4).

Observations and remarks, potential research gaps and subjective EFSA-grade evaluations for this section are summarised in Table 3.

## 4 Characteristics of the fermented foods and their bioactive compounds

In the present systematic review, we investigated FFs as a whole for their impact on GI wellbeing and associated symptoms/outcomes. FF interventions reported in eligible studies varied widely with respect to their origin, substrate composition, microbiological characteristics, type of fermentation, and dosage, among others (Supplementary Table S3). Based on their biological source, the identified FFs could be broadly categorised into animal- and plant-derived FFs. Among animal-derived FFs, most eligible studies focused on fermented dairy products, such as fermented milk, yogurt, cheese, kefir, and whey (54, 58, 60–64, 66, 68, 70–77) whereas plant-derived FFs reported in the eligible studies included a range of products such as fermented rice, soy milk, *B. rapa*, cabbage, cucumbers, among others (55–57, 59, 65). The dairy-based interventions frequently employed substrates such as whole milk, skimmed milk, or non-fat dry milk solids, including additives and fortifiers such as sweeteners, flavouring agents, and prebiotic fibres, to enhance palatability and functionality. Notably, fermented dairy products were overrepresented among the included studies, reflecting both the historical dominance of dairy-based research in this field and possibly greater availability of standardised commercial products suitable for clinical use. Interestingly, one study reported an unusual FF intervention product in a fermented sericin-fibroin mixture extracted from silkworm; given its non-traditional nature as a fermented product, this was not categorised as an animal-derived product (67).

The nutritional composition of the FFs revealed a combination of consistent features and substantial variability, largely determined by substrate type, microbial strains, and product formulation (Supplementary Table S3). Across both dairy- and plant-based fermented products, carbohydrates, modest protein levels, low fat content, high moisture, and moderate energy values were recurrent features. Most fermented beverages, particularly those derived from milk, contained moderate carbohydrate levels ranging from approximately 4.8–18.0 g/ 100 mL, derived from intrinsic sugars such as lactose, glucose, or added sweeteners like sucrose and fructose. The protein content of FF interventions typically fell within the range of 0.0–3.6 g per serving, depending on the source (e.g., vegetables, milk, soy, or protein mixtures) (Supplementary Table S3). Though not protein-rich *per se*, these values contribute to meaningful daily protein intake, particularly in regularly consumed commercial products. While milk proteins dominate in conventional fermented dairy beverages, more unique proteins—such as sericin and fibroin from silkworm-derived substrates, can introduce novel bioactive peptides with potentially novel therapeutic effects. Fat content was found to be consistently low across most liquid fermented products, ranging between 0.00–1.28 g/100 mL, reflecting the widespread use of low-fat or skimmed milk in formulations (Supplementary Table S3). Lipid content showed minimal variation in milk-based products but increased notably in non-dairy fermentations. For instance, fermented brown rice reported in Nemoto et al. (59) contained over 5 g of lipids per serving, compared to less than 0.1 g in most fermented milks, highlighting the nutritional density of grain-based fermentations (Supplementary Table S3). In addition, the moisture content (when analysed) was universally high (> 80%), as expected in beverage forms, and energy values were relatively wide, ranging between 5.0–127.4 kcal per serving, depending on the sugar and fat content.

Among other ingredients in the FF interventions found in our eligible studies, dietary fibre was understandably absent in dairy-based fermented beverages but was present in various amounts in plant-based fermented products such as fermented rice bran (5.19 g), *B. rapa* (0.75 g), and vegetable-based preparations (1.00 g) (56, 57, 59) (Supplementary Table S3). Sodium content varied widely in the FF interventions, being negligible in most dairy-based FFs, particularly cheeses, but significantly elevated in fermented cabbage and cucumbers, exceeding 200 mg per serving, due to salt-based preservation methods such as brining and pickling. This variation can potentially have implications for populations with sodium-sensitive health conditions.

Eligible studies using animal derived FFs commonly used microbial strains from the *Lactobacillus* (and related) and *Lactococcus* genera, which are traditionally associated with fermented dairy products (Table 2, Supplementary Table S3). Some interventions included the use of probiotic bacteria for FF production. In contrast, plant-derived FFs were produced using a broader range of microorganisms, including *Aspergillus, Leuconostoc, Weissella,* and *Lacticaseibacillus* spp., reflecting the greater microbial diversity usually characteristic of traditional plant-based fermentations (Table 2). Additionally, most interventions used daily microbial doses in the range of 10^9^ to 10^11^ CFU/day. This corresponds to a range around 10^10^ CFU/day, a putative level of live microbe consumption that is thought to be beneficial for health, as mentioned above. The microbial loads reported in the FF interventions of the eligible studies was notably higher than the microbial counts typically found in many traditionally consumed fermented foods such as sauerkraut, kimchi, kefir, yogurt, cheese, kombucha, and miso that commonly contain viable microbial populations in the range of 10^6^ to 10^9^ CFU/g or CFU/mL (11, 14).

FFs are a rich source of diverse bioactive metabolites that significantly influence both food quality and potential health benefits. These bioactive compounds include peptides, amino acids, vitamins, exopolysaccharides, oligosaccharides, isoflavones, phenolic compounds, organic acids, and short-chain fatty acids (SCFAs) (86, 87). The composition and functionality of these compounds can vary widely depending on the fermentation substrate, microbial strains used, and fermentation conditions, resulting in products with distinct nutritional and functional profiles (88). Our review of the included studies revealed that many hypothesised a role for bioactive metabolites in the observed health effects of fermented foods; however, these claims were frequently made without direct evidence from the trials themselves to substantiate them (Supplementary Table S3).

In terms of physical form (texture), liquid fermented foods were the most commonly encountered in the interventions, whereas granulated and solid forms appeared less frequently (Supplementary Table S3). Importantly, while taste, texture, aroma, and overall palatability are crucial determinants of consumer acceptance and compliance, none of the included studies provided a formal sensory evaluation of the fermented products. This omission is particularly significant considering that flavour, mouthfeel, and appearance play a major role in shaping the perceptions and habitual consumption of FFs, particularly across diverse age groups and cultural contexts. The absence of such data not only limits the understanding of participant adherence and long-term feasibility but also disconnects clinical outcomes from real-world consumer experiences, especially relevant for public health applications and personalised nutrition approaches.

Notably, most studies omitted essential details related to the fermentation method. Specifically, many studies failed to report the type of fermentation employed (e.g., lactic acid, alcoholic, mixed), duration of fermentation, environmental conditions (e.g., temperature, oxygen levels, pH), or post-fermentation storage conditions (e.g., refrigeration, shelf-life, packaging protocols). In addition, none of the included studies reported adherence to Good Manufacturing Practices (GMP) or Hazard Analysis and Critical Control Points (HACCP) standards, which are critical for ensuring the safety, quality, and consistency of food products, especially in clinical settings. It is however possible that GMP (and if relevant HACCP) were followed and simply not mentioned, as it’s often a necessity for production licenses. Nevertheless, the lack of these quality assurance details raises concerns about batch-to-batch variability, product stability, and the reliability of health outcome assessments.

Observations and remarks, potential research gaps and subjective EFSA-grade evaluations for this section are summarised in Table 3.

## 5 Mechanisms of action

### 5.1 Current mechanistic understanding

Although the focus of our study was on healthy populations, most outcomes investigated hold relevance to constipation, characterised by infrequent bowel movements, hard or dry stools (related to stool consistency and water content), perturbed intestinal transit time (closely linked to gut motility), straining during defaecation, feeling of incomplete evacuation, abdominal discomfort, and bloating, among others (89). Constipation (and gut motility) is understood to be influenced by a complex interplay between the central nervous system (CNS), enteric nervous system (ENS), the gut microbiota and fermentation, as well as immune function, all of which can be influenced by FFs (8, 9). In this context, evidence suggests a depletion in *Bifidobacterium* spp. and *Lactobacillus* spp. in constipation, as well as a reduction in butyrate-producers *Roseburia intestinalis* and *Faecalibacterium*, with the latter correlating with impaired mucosal barrier function and reduced transit (90, 91). Further, faecal microbiota composition correlated with both colonic transit time and constipation status within a case–control study design, even following adjustment for age, body mass index (BMI), dietary intake, and transit time (92).

Metabolites produced by the gut microbiota such as SCFAs and peptides can impact the ENS and gut transit (93). Butyrate exerts a biphasic effect on gut motility with an enhancement of proximal colonic peristalsis at physiological concentrations (10–30 mM), while higher doses (> 50 mM) inhibit motility (94). This biphasic effect arises from butyrate’s ability to stimulate 5-hydroxytryptamine (5-HT) release from enterochromaffin cells, activating 5-HT₃ receptors on vagal afferents to modulate contractile activities and 5-HT₄ receptors on enteric neurons to facilitate secretion and propulsive motility (94–96). In this context, constipation-associated dysbiosis reduces butyrate synthesis while increasing propionate production in the gut, creating an imbalance that favours delayed transit (90, 91). Importantly, FFs can provide substrates to facilitate the production of such metabolites by way of lactate (conversion to SCFAs) and proteins (conversion to peptides) (97). It should also be noted that in addition to metabolites produced, the gut microbiota has the capacity to directly initiate 5-HT release in the gut (98).

Constipation (excepting transient diet-related constipation) linked to low-grade mucosal inflammation driven by increased intestinal permeability (91). Gut microbial dysbiosis can downregulate tight junction proteins and MUC2 (the major intestinal mucin) expression, culminating in compromised intestinal barrier integrity (99, 100). Butyrate counteracts this inflammation by suppressing nuclear factor-κB (NF-κB) activation and promoting regulatory T-cell differentiation through histone deacetylase inhibition (101). Defaecation also depends on appropriate intestinal secretion, with perturbed intestinal fluid and electrolyte homeostasis being another characteristic of constipation (102). SCFA regulate 5-HT-mediated intestinal fluid and electrolyte secretion via 5-HT_3_ receptors (103), as well as stimulating intestinal absorption of water and sodium (91). The microbial interplay is demonstrated by strong associations between stool consistency and water content with gut microbiota richness and enterotypes (104).

### 5.2 Mechanistic insights into fermented foods and GI wellbeing

FFs identified in the present review are evidenced to increase the abundance of putative SCFA-producing microbes in the gut as well as increase SCFAs. Kim et al. (105) investigated the effects of 210 g/day of kimchi, similar to fermented cabbages or turnips included in the present review, for 28 days in healthy young Korean adults, with an increased abundance of butyrate producing *Faecalibacterium* and *Roseburia* reported in stool samples (106). The kimchi intervention also reduced faecal pH, which can facilitate pathogen inhibition in the gut, with reductions in *Clostridium* sp. and *Escherichia coli* group counts notably reported. Faecal pH reduction can also suggest greater SCFA load, however, the impact of the intervention on SCFA production cannot be confirmed as this was not reported. A later study by the same group evaluated a reciprocal dosing of 210 g/day of kimchi over a longer period of 12 weeks (107) with an increase in faecal *Bifidobacterium adolescentis* reported. Importantly, although not a significant butyrate producer, *B. adolescentis* participates in bacterial cross-feeding mechanisms producing butyrogenic effects in the gut (108). SCFAs were again not measured, thus, the impact on SCFA production cannot be confirmed.

Veiga et al. (109), explored the impact of consuming 125 g/day of a fermented milk product containing the microbial consortium *B. animalis* subsp. *lactis*, *S. thermophilus*, *L. bulgaricus* and *L. lactis* over a 4-week study period in subjects with Rome III IBS with constipation. The product impacted the gut microbial butyrate producing community, increasing butyrogenic metabolic modules which coincided with increased faecal SCFA content, including butyrate. The nuanced findings concerning FFs’ impact on SCFA production may lie in the measurement of SCFA in stool, which is not wholly reflective of proximal concentrations, as > 95% SCFA are absorbed in the colon (110). This is corroborated by a recent study, which found that pasteurised sauerkraut caused an increase in serum SCFAs but not stool SCFAs (111). Nevertheless, the above evidence supports the widely accepted hypothesis that amelioration of gut function with live microbes, where gut SCFA concentrations can be increased, may indirectly benefit GI function, such as improving gut motility via the mechanisms indicated above (112).

There have also been several reports of beneficial, direct impacts of certain microorganisms associated with the FFs described in this review on GI wellbeing related outcomes. Faecal water content increased by 15–20% compared to controls in constipated rats with *B. lactis* and *Lcb*. *casei* Shirota, two microbes encountered in our studies (113). Kefir, reported in the included study of Alves et al. (52), demonstrated a beneficial effect on both stool bulk and water content. In this context, administration of *L. kefiranofaciens*, an important microbe in the kefir fermenting microbial consortium, was reported to increase stool weight (or bulk) by 25% and moisture content by 18% compared to controls (114). Additionally, kefiran, a complex polysaccharide found in kefir, was reported to improve stool moisture content in rats in a dose-dependent manner with high doses (200 mg/kg) achieving a 22% increase (115). Our subgroup analyses revealed that a mix of fermenting microbes containing *B. lactis*, *S. thermophilus* and *L. bulgaricus* can improve transit time, symptoms of bloating, as well as overall GI symptoms. Interestingly, when the effects of milk fermented with this microbial consortium was tested in participants with functional constipation, a significant improvement in defaecation frequency, number of incomplete defaecations and defaecation pain was reported via 200 g/day (116). Additionally, this same microbe consortium was investigated in the study by Veiga et al. (109) within a fermented milk product, where alongside the noted gut microbial changes, the intervention improved abdominal distension, gastrointestinal transit times, as well as overall IBS symptom severity. Interestingly, our analyses revealed a positive impact of *Lcb*. *casei* Shirota fermentation on incidence of hard stools as well as GI symptoms. Previous work observed that consumption of a *Lcb*. *casei* Shirota fermented beverage for 28 days led to benefits in markers of bowel function, with an increase in faecal pipecolinic acid (PIPA) concentrations which correlated with stool frequency (117). PIPA was then investigated in a mouse model which suggested augmentation of 5-HT and acetylcholine (ACh) levels could implicate PIPA in the interplay between the gut microbial mediated constipation alleviation, but elucidation of the distinct mechanism is required. There are several more examples where fermented interventions with beneficial microbes such as lactic acid bacteria and *Bifidobacterium* spp. have contributed to GI function and symptom improvement, discussion of which is beyond the scope of this review.

It should be noted that some of our outcomes are subjective in nature, i.e., abdominal pain, bloating, and borborygmi, among others, and can be difficult to evaluate in animals (and certainly not possible *in vitro*). Supporting evidence for such outcomes can therefore only be provided through clinical studies conducted in healthy, IBS or constipated individuals (as mandated as acceptable populations by EFSA). Indeed, a recent meta-analysis examined 16 RCTs including 1,264 individuals to understand the impact of FFs in IBS across similar outcomes detailed in our study, and reported FFs are efficacious in symptom relief and improve global symptom scores (118). Similarly, previous meta-analyses have investigated the impact of probiotics and symbiotics in constipation. A meta-analysis by Dimidi et al. on 14 RCTs including 1,182 constipated patients demonstrated that probiotics significantly reduced whole gut transit time and increased both stool consistency and frequency (84). A more recent meta-analysis by van der Schoot et al. suggested that individuals suffering from chronic constipation can benefit from probiotic administration in terms of stool frequency and global constipation-related symptoms, with a higher response rate to probiotics compared to controls (79). Interestingly, both of these latter studies reported no beneficial impact for *Lcb. casei* Shirota on stool frequency and stool consistency. While this was largely consistent with our results involving *Lcb. casei* Shirota fermented dairy products, we did find a beneficial effect when considering the incidence of hard stools (Supplementary Figure S4D). This discrepancy might be attributed to this particular outcome not being investigated in these two studies as well as the difference in eligible cohorts (constipated vs. healthy). As mentioned above, no meta-analyses for GI wellbeing related symptoms have been undertaken previously. Due to the availability of the recent meta-analyses as mentioned above, we have not detailed the individual studies here and a list of relevant studies are made available in Supplementary Table S4.

Overall, it is evident that FFs have the potential to improve GI function in healthy populations without major gastrointestinal disorders. Our current understanding indicates that the gut microbiota plays a major role in facilitating gastrointestinal function, with gut microbial metabolites, particularly SCFAs such as butyrate, influencing bowel function through a multitude of complex, interconnected mechanisms. However, the mechanisms underlying the impact of FFs on some of these outcomes need further clarification. An important point to consider is that the effect of FF and probiotic interventions appear to be strain and study population specific (119, 120), reflected by inconsistent findings in the current evidence base.

Observations and remarks, potential research gaps and subjective EFSA-grade evaluations for this section are summarised in Table 3.

## 6 Bioavailability of bioactive compounds

Fermentation has the capacity to enhance the digestibility and nutritional properties of foodstuffs via the release and generation of compounds within the food matrix. As such, a plethora of bioactive compounds enter the gut following FFs consumption, with potential to elicit positive influences on gastrointestinal function and wellbeing. Importantly, as noted above, increased bioactive compounds in the FFs likely modulate GI function in an indirect fashion rather than direct, as discussed below in brief.

A key theme in the present review is the beneficial impact of dairy-based FFs, often enriched in bioavailable, health-promoting compounds. For example, cheese fermentation by LAB and propionic acid bacteria can concentrate SCFA content in the matrix via casein hydrolysis and lactose metabolism, particularly the SCFAs acetate and propionate, although these might be absorbed in the proximal colon and not reach the distal colon (121–123). Yogurt fermentation by *L. bulgaricus* and *S. thermophilus* generates acetate and lactate, which reduce luminal pH, inhibiting pathogen expansion and favouring beneficial microbes such as butyrate producing *Faecalibacterium prausnitzii* in the colon (124). Kefir, comprising a diverse microbial consortium including *Lentilactobacillus kefiri* and *Acetobacter*, can be enriched in acetate and butyrate, while its *β*-glucans and kefiran can serve as prebiotics, enhancing microbial SCFA generation (9, 125). Fermentation is also notable for metabolites it reduces, such as lactose, enabling individuals with lactose intolerance to consume fermented dairy products without symptom induction (126), while it may also reduce global GI symptoms in populations with less severe forms of lactose malabsorption.

Fermented plant-based foodstuffs hold potential to contribute to planetary health through the leverage of plant-based sources. However, the differential production of plant-based FFs results in different bioactive profiles compared to animal based FFs, which may contribute to health improvement in an additive facet. In East Asian fermentation tradition, rice products are fermented with *Aspergillus oryzae*, a filamentous fungus (127), which generates an array of bioactive compounds during rice fermentation. Examples include compounds of interest in gastrointestinal health such as *γ*-aminobutyric acid (GABA), other bioactive peptides, as well as β-glucans. In the context of the present review, oral administration of GABA-producing *Bifidobacterium* species increased caecal GABA levels and reduced colon-specific sensory neuron excitability, mechanisms involved in abdominal pain induction (128). As noted for kefir above, β-glucans act as prebiotic substrates and further improve the composition and metabolic output of the gut microbial community (129). Fermented rice, as well as fermented vegetables such as sauerkraut and kimchi, comprise microbial consortia which facilitate the production of organic acids in the matrix like dairy FFs, producing metabolites such as lactic acid, which contribute to pH reduction in the gut and promotion of beneficial microbes in conjunction with inhibition of harmful microbes (130). Fermented vegetables can also be a reservoir for phenolic compounds, which have an array of bioactive traits including antioxidant and anti-inflammatory properties (131).

More information on bioactive compounds in these FFs can be found in previously published reviews (132, 133). Observations and remarks, potential research gaps and subjective EFSA-grade evaluations for this section are summarised in Table 3.

## 7 Safety

Regarding safety of the included FF interventions, adverse events were reported in 14 studies (57–60, 62–68, 73, 75, 76) with Aslam et al. (77) not applicable being an observational study (Supplementary Table S5). Several studies simply reported that there were no adverse events (58–60, 62–66, 68, 73, 75, 76). Noda et al. (67) reported that some subjects suffered from transient diarrhoea during the study period; no other adverse effects were reported (Supplementary Table S5). Galena et al. (57) reported GI symptoms such as bloating and abdominal pain as adverse effects; however, for the purposes of this review they were included as outcomes (Table 2). Briefly, ~50 and 18% of the participants in the intervention group experienced bloating and abdominal pain, respectively, with 30 and 40% of the control group experiencing the same symptoms, respectively, (Supplementary Table S5).

Observations and remarks, potential research gaps and subjective EFSA-grade evaluations for this section are summarised in Table 3.

## 8 Limitations and summary of evidence

Even though the current study was extensive, some limitations still exist (several are covered in Table 3). For example, individuals with IBS and functional (and severe) constipation are accepted by the EFSA as a target demographic for physiological benefits concerning GI wellbeing but were not included in the present work. It should be noted that healthy populations with some GI symptoms identified as the population of interest in the current study may be part of an undiagnosed IBS (IBS-U) demographic; this was however not possible to be verified as this level of granularity is seldom provided in studies. Additionally, only articles in English were included to enable the cross-national collaborative ecosystem characteristic of COST Actions. Furthermore, the Embase bibliographic database, recommended by Cochrane as a minimum requirement for medicine-oriented systematic reviews, was not included in our work due to logistical issues. Notably, some data could not be obtained from authors due to the inordinate time taken for agreements and memoranda. While these factors do not take anything away from our findings, they do provide an opportunity for updating and expansion of the research in the recent future, not least because of the increasing number of clinical trials being conducted for complex FFs in recent years.

An important consideration regarding the limitations of the study is related to the interpretation of the results discussed. In the current work, we have deliberately considered FFs *in toto*, where an argument can be made that fermented dairy, soy, and vegetables, among the many other FF types considered in this review, are inherently too different to be considered together. For example, certain plant-based FFs may have a higher concentration of fermentable fibres that could possibly contribute more to alleviating constipation compared to other FF types; considering FFs together might give the impression that all FFs are good for ameliorating constipation-related symptoms. While the objective in this current work was to isolate the effects of fermentation on consequent health benefits, we have considered this and provided subgroup analyses for base fermentation matrices for each outcome (Supplementary material) that helps understand if a certain FF type might be having a significantly greater influence on the final summary effect metric. However, due to the paucity of studies eligible for the meta-analysis, these subgroups often have 1–2 studies under each group. Related to this, contributions of certain FFs delivering dietary live microbes and health benefits being associated to these microorganisms should be considered too. Indeed, in several instances these microbes can be the primary determinants of health benefits from a particular FF and has contributed to the growing discourse in the space for a possible recommended dietary allowance for microorganisms (1, 134, 135). Interpretations for each outcome therefore must be made with due diligence and caution. We have outlined the issues with FF trial design throughout the manuscript and particularly in Table 3. With better designed trials, we should be able to isolate contributions made to health benefits not only by the food matrices themselves, but the process of fermentation, microorganisms and specific bioactive compounds, among others. This extensive systematic review and meta-analysis, however, provides an important foundation for furthering FF research on GI health outcomes.

Some notable exceptions, although not necessarily limitations, must also be mentioned. For example, in this work we did not consider alcoholic fermented beverages. While fermented alcoholic beverages have been shown to provide important and diverse health benefits when consumed in moderation (136, 137), we took this decision to align with that of the EFSA, which does not consider health claims for beverages with an alcohol content by volume of 1.2% or higher (27). Within our analysis, there is also an absence of meat-based FFs, and we are therefore unable to comment on the benefits from such foodstuffs, if any. When identifying studies which fulfilled our eligibility criteria, no studies comprising meat based FF were deemed eligible, with very few meat-based FF studies retrieved during the bibliographic search, indicating a general lack of investigations in these foods. This raises an interesting notion, as consumption of processed meat has strong evidence of negative health consequences, notably with colorectal cancer in both observational (138) and interventional (139) models. It would therefore be of interest to determine whether meat-based FF carry negative consequences for consumers, or indeed, if the fermentation element renders such foodstuffs capable of eliciting similar benefits as what has been found amongst the FFs in the present analysis. Additionally, most studies found eligible for this review are focused on lactic acid bacteria and to some extent, yeasts. This was not due to a specified exclusion of soy-based FFs, most often fermented by bacilli and fungal genera such as *Aspergillus*, which are known to contribute to health (140); rather this was merely a consequence of the eligibility criteria implemented in the current work. Furthermore, FFs are deeply influenced by geographical and cultural traits, with trials and studies in Northa America and Europe often focused on fermented dairy and vegetables, whereas more studies on fermented soy and cereal based foodstuffs can be recovered from Asia.

It must be additionally noted that our study has deliberately not investigated changes in the gut microbiota brought about by consumption of FFs. This decision was based on the EFSA guidance for health claims that do not consider such evidence substantive on their own for health claims (28) (Table 1). The role of the gut microbiota in GI health benefits is however discussed in the mechanisms of action, as part of the accepted supportive evidence section of EFSA health claims. Importantly, the gut microbiota remains a critical component of actualising health benefits through foodstuffs even though it is currently not considered as a clinical outcome by EFSA; the beneficial modulation of the gut microbiota by FFs is widely accepted (10, 18).

Several of the outcomes discussed in the review are also subjective in nature, derived from various questionnaires on gastrointestinal health. It’s known that for such outcomes, sometimes the placebo response rate for improvement in symptoms can be quite high (up to 40%). This should be taken into consideration during interpretation of results. None of the studies included in this work reported any such deviation, however, some studies included sensorial validations for the placebo and intervention products (data not reported here) providing more certainty for the results obtained. Somewhat related, we have also deliberately included all controls that were not fermented in nature. Several of these are not ideal controls (for, e.g., no consumption, water etc.) but were included as they do not essentiality void the eligibility criteria. A gradation of comparators is provided in Table 2, and results should be interpreted with caution in relation to quality of comparators. Again, this raises the notion of the requirement for better designed trials in the future.

We have summarised our observations and remarks regarding EFSA-guided requirements for an evidence-based health claim for FFs in gastrointestinal wellbeing in Table 3, with an overall subjective claim for the level of evidence made as well. Beyond our observations, research gaps, particularly in unstandardised non-fermented comparators, heterogeneity in studies (as has been discussed previously as well in Iyer et al. (26)), understanding the molecular mechanisms driving the effect of FFs on the outcomes of interest and relevant bioactive enrichment in FFs were identified. A general lack of evidence and research funding to investigate the impact of consuming traditional/artisanal FFs, particularly in non-dairy matrices, was also apparent. An absence of studies vertically integrating the impact of an FF intervention to the molecular level (such as correlating with luminal SCFA levels) was also apparent. Most studies lacked information on batch-to-batch variability, which would also need to be addressed in future trials in terms of consistently conveyed health benefits across multiple batches of FFs. Overall, we have therefore subjectively attributed the EFSA evaluation wording “Neither convincing nor sufficient” for our research question/simulated health claim in relation to FF consumption and gastrointestinal wellbeing. Information on research gaps and other potential barriers including non-standardised experimental designs, will be further elaborated and structured in the upcoming ‘Strategic Roadmap for fermented foods research’ document that will be the capstone deliverable for PIMENTO WG3, discussing additionally the possible future directions of FFs research and what should be the critical areas of research interest. Ultimately, this review is not meant to be a fully conclusive piece of evidence for FF consumption *vis-à-vis* GI wellbeing but is anticipated to provide and inform a strong foundation for FF research in gastrointestinal health and wellbeing moving forward.

## Data Availability

The original contributions presented in the study are included in the article/Supplementary material, further inquiries can be directed to the corresponding authors.

## Footnotes

^1^ https://osf.io/q8yzd
