# Supplementary material for: Impact of fermented foods consumption on gastrointestinal wellbeing in healthy adults: a systematic review and meta-analysis
Source: Front Nutr. 2025 Oct 10;12:1668889. doi: 10.3389/fnut.2025.1668889 (PMC12549620; doi:10.3389/fnut.2025.1668889)
Supplement: Supplementary file 1 [file Data_Sheet_1.pdf]

Supplementary Information

Impact of fermented foods consumption on gastrointestinal wellbeing in healthy adults: a systematic review and meta-analysis

Arghya Mukherjee[1,2]*, Dominic N. Farsi[3], Enriqueta Garcia-Gutierrez[1,2,4§], Ecem Akan[5,§], Jose Angel Salas Millan[2,6,§], Ljupco Angelovski[7,§], Thomas Bintsis[8,§], Amaury Gérard[9,§], Ziba Güley[10,§], Sümeyye Kabakcı[11,§], Minna Kahala[12,§], Ryma Merabti[13,#,§], Foteini Pavli[14,§], Elisa Salvetti[15,§], Cem Karagözlü[16], Nurcan Bağlam[17], Bahtir Hyseni[18], Simona Bavaro[19], Konstantinos Papadimitriou[20], Eun-Hee Doo[21], Christophe Chassard[3], Smilja Praćer[22], Guy Vergères[23], Paul D. Cotter[1,2,24] and Sandra Mojsova[7]*

[1]Department of Food Biosciences, Teagasc, Fermoy, Cork, Ireland
[2]APC Microbiome Ireland, Cork, Ireland
[3]Human Nutrition Unit, INRAE, Université Clermont-Auvergne, Clermont-Ferrand, France
[4] Agronomic Engineering Department, Technical University of Cartagena, Murcia, Spain
[5]Adnan Menderes University, Türkiye
[6]University College Cork, Cork, Ireland
[7]Faculty of Veterinary Medicine, University Ss Cyril and Methodius, Skopje, North Macedonia
[8]Faculty of Veterinary Medicine, Aristotle University of Thessaloniki, Greece
[9]Brewing & Food Science Unit, LABIRIS, Anderlecht, Belgium
[10]Department of Food Engineering, Alanya Alaaddin Keykubat University, Türkiye
[11]National Food Reference Laboratory, Ministry of Agriculture and Forestry, Ankara, Türkiye
[12]Productions systems, Food and Bioproducts, Natural Resources Institute Finland (Luke), Finland
[13]Faculty of Natural and Life Sciences, Department of Cellular and Molecular Biology, Abbes Laghrour University, Khenchela, Algeria
[14]Department of Food Sciences and Nutrition, University of Malta
[15]Department of Biotechnology and Verona University Culture Collection (VUCC-DBT), University of Verona, Italy
[16]Department of Dairy Technology, Ege University, Izmir, Türkiye
[17]Department of Nutrition and Dietetics, Sivas Cumhuriyet University, Türkiye
[18]Faculty of Food Technology, University "Isa Boletini" in Mitrovica, Kosovo
[19]Institute of the Sciences of Food Production, National Research Council, Turin, Italy.
[20]Department of Food Science and Human Nutrition, Agricultural University of Athens, Athens, Greece
[21]Department of Yuhan Biotechnology, School of Bio-Health Sciences, Yuhan University, Bucheon, Republic of Korea
[22]Institute for Biological Research Siniša Stanković, National Institute of the Republic of Serbia, University of Belgrade, Serbia
[23]Agroscope, Switzerland
[24]Vistamilk, Cork, Ireland
[#]Laboratory of Biotechnology and Food Quality, Institute of Nutrition, Food and Agri-Food Technologies (INATAA), Constantine 1 University, Algeria

[§]These authors contributed equally.

Correspondence should be addressed to: arghya.mukherjee@teagasc.ie and kostova.sandra@fvm.ukim.edu.mk

Table of contents

Item	Description	Page number
Supplementary Table S1	Search strategy for bibliographic search of published human studies related to the health benefits and risks of fermented foods.	3
Supplementary Table S2	Risk of bias assessements for outcomes of interest across studies.	7
Supplementary Table S3	Characteristics of the fermented food interventions in eligible studies included in the review.	10
Supplementary Table S4	Supportive evidence: relevant studies for mechanisms of action	13
Supplementary Table S5	Summary of confounding variables, adverse events and compliance in included studies.	15
Supplementary Figure S1A-D	Subgroup analysis for stool frequency	29
Supplementary Figure S2A-D	Subgroup analysis for stool consistency	34
Supplementary Figure S3A-D	Subgroup analysis for stool consistency (BSFS).	39
Supplementary Figure S4A-D	Subgroup analysis for incidence of hard stools	44
Supplementary Figure S5A-D	Subgroup analysis for stool bulk	49
Supplementary Figure S6A-D	Subgroup analysis for stool pH	54
Supplementary Figure S7A-D	Subgroup analysis for stool water content	59
Supplementary Figure S8A-D	Subgroup analysis for intestinal transit time	64
Supplementary Figure S9A-D	Subgroup analysis for abdominal symptoms	68
Supplementary Figure S10A-D	Subgroup analysis for abdominal pain	73
Supplementary Figure S11A-D	Subgroup analysis for bloating	78
Supplementary Figure S12A-D	Subgroup analysis for borborygmi	83
Supplementary Figure S13A-D	Subgroup analysis for flatulence	88
Supplementary Figure S14A-D	Subgroup analysis for degree of constipation	92
Supplementary Figure S15A-D	Subgroup analysis for feeling of incomplete evacuation	97
Supplementary Figure S16A-D	Subgroup analysis for straining during defaecation	102
Supplementary Figure S17A	Forest plot of sensitivity analysis for stool frequency	107
Supplementary Figure S17B	Forest plot of sensitivity analysis for hard stools	108
Supplementary Figure S17C	Forest plot of sensitivity analysis for stool pH	109
Supplementary Figure S17D	Forest plot of sensitivity analysis for stool water content	110
Supplementary Figure S17E	Forest plot of sensitivity analysis for stool bulk	111
Supplementary Figure S17F	Forest plot of sensitivity analysis for intestinal transit time	112
References	Reference list	113

Supplementary Table S1. Search strategy for bibliographic search of published human studies related to the health benefits and risks of fermented foods. The "OR" operator separates different food groups.

Search string #	Search code
**MEDLINE**	
#1	"Fermented Foods"[Mesh] OR "Fermentation"[Mesh] OR ("Food"[Mesh] AND ferment*[tiab]) OR ((ferment*[tiab] OR cultur*[tiab] OR leaven*[tiab]) AND (food*[tiab] OR drink*[tiab] OR beverage*[tiab])) OR "Fermented product"[tiab:~6] OR "Fermented products"[tiab:~6] OR "cultured product"[tiab:~6] OR "cultured products"[tiab:~6] OR "product fermentation"[tiab:~6] OR "products fermentation"[tiab:~6] OR "starter culture*"[tiab] OR ((ferment*[tiab] OR culture*[tiab] OR sour*[tiab]) AND (milk[tiab] OR dairy[tiab])) OR buttermilk[tiab] OR sour cream*[tiab] OR cheese*[tiab] OR yoghurt[tiab] OR yogurt[tiab] OR "yoghourt"[tiab] OR "yakult"[tiab] OR "quark"[tiab] OR "kefir"[tiab] OR "lassi"[tiab] OR "kumis"[tiab] OR "koumiss"[tiab] OR "kajmak"[tiab] OR "airag"[tiab] OR "ayran"[tiab] OR "calpis"[tiab] OR "borhani"[tiab] OR "chal"[tiab] OR "doogh"[tiab] OR kvass[tiab] OR skyr[tiab] OR amasi[tiab] OR bouza[tiab] OR butter*[tiab] OR chal[tiab] OR filmjolk[tiab] OR kishk[tiab] OR labne*[tiab] OR ((Ferment*[tiab] OR cultur*[tiab] OR cured[tiab]) AND (meat*[tiab] OR fish*[tiab] OR seafood*[tiab] OR shellfish[tiab] OR sausage*[tiab])) OR "salami"[tiab] OR "pepperoni"[tiab] OR peperoni[tiab] OR "chorizo"[tiab] OR "cervelat"[tiab] OR "mettwurst"[tiab] OR "summer sausage"[tiab] OR "sucuk"[tiab] OR "dried meat"[tiab] OR "dried sausage"[tiab] OR "dry sausage"[tiab] OR "fish sauce"[tiab] OR "shrimp paste"[tiab] OR "shrimp sauce"[tiab] OR "oyster sauce"[tiab] OR "prosciutto"[tiab] OR "pancetta"[tiab] OR "saucisson"[tiab] OR sucuk[tiab] OR ((Ferment*[tiab] OR cultur*[tiab]) AND (fruit*[tiab] OR vegetable*[tiab] OR coconut*[tiab] OR almond*[tiab] OR hazelnut*[tiab] OR nut[tiab] OR cucumber*[tiab] OR lemon*[tiab] OR citrus[tiab] OR cabbage*[tiab] OR cauliflower*[tiab] OR pepper*[tiab] OR carrot*[tiab] OR olive*[tiab] OR onion*[tiab] OR sago[tiab])) OR "sauerkraut"[tiab] OR "table olive"[tiab] OR pickle*[tiab] OR "kimchi"[tiab] OR "paocai"[tiab] OR torshi[tiab] OR ((Ferment*[tiab] OR cultur*[tiab]) AND (tea[tiab] OR teas[tiab] OR juice*[tiab])) OR "beer"[tiab] OR "wine"[tiab] OR "cider*"[tiab] OR shochu[tiab] OR "kombucha*"[tiab] OR "pulque"[tiab] OR puer[tiab] OR pu'er[tiab] OR pu-er[tiab] OR pu-erh[tiab] OR "pu erh"[tiab] OR "fuzhuan"[tiab] OR "dark tea*"[tiab] OR "yellow tea*"[tiab] OR coffee[tiab] OR shalgam[tiab] OR hardaliye[tiab] OR ((Ferment*[tiab] OR cultur*[tiab]) AND (soy[tiab] OR soya[tiab] OR bean*[tiab] OR pea[tiab] OR peas[tiab] OR lentil*[tiab] OR chickpea*[tiab] OR legume*[tiab] OR pulse*[tiab] OR (poi[tiab])) OR "soy sauce"[tiab] OR "soya sauce"[tiab] OR "soybean paste"[tiab] OR "miso"[tiab] OR "tempeh"[tiab] OR tempe[tiab] OR "natto"[tiab] OR "doenjang"[tiab] OR "doubanjiang"[tiab] OR douchi[tiab] OR "gochujang"[tiab] OR cheonggukjang[tiab] OR tsukemono[tiab] OR garri[tiab] OR ((Ferment*[tiab] OR cultur*[tiab] OR leaven*[tiab]) AND (cereal*[tiab] OR grain*[tiab] OR wheat*[tiab] OR oat[tiab] OR oats[tiab] OR rice*[tiab] OR millet*[tiab] OR sorghum*[tiab] OR maize*[tiab] OR rye[tiab] OR barley*[tiab] OR chia[tiab] OR oilseed*[tiab] OR teff[tiab])) OR "bread"[tiab] OR "sourdough"[tiab] OR "crispbread"[tiab] OR "boza"[tiab] OR "ogi"[tiab] OR dosa[tiab] OR "tarhana"[tiab] OR "buckwheat"[tiab] OR "spelt"[tiab] OR "einkorn"[tiab] OR "quinoa"[tiab] OR "amaranth"[tiab] OR "tef"[tiab] OR "bushera"[tiab] OR chica[tiab] OR chicha[tiab] OR choujiu[tiab] OR injera[tiab] OR mahewu[tiab] OR ogiri[tiab] OR pozol[tiab] OR ugba[tiab]) OR ((Ferment*[tiab] OR cultur*[tiab]) AND ("condiment*"[tiab] OR relish*[tiab] OR horseradish[tiab] OR "dressing*"[tiab] OR "seasoning*"[tiab] OR "sauce*"[tiab] OR cocoa*[tiab] OR tuber[tiab] OR "acetic acid"[tiab])) OR "chocolate*"[tiab] OR "vinegar*"[tiab] OR "tabasco"[tiab] OR "sriracha"[tiab] OR "Worcestershire"[tiab] OR "Worcester"[tiab]
#2	"Diet"[Mesh] OR "Life Style"[Mesh] OR "Eating"[Mesh] OR "Feeding Behavior"[Mesh] OR ((food[tiab] OR macronutrient*[tiab] OR eating[tiab]) AND (intake*[tiab] OR habit*[tiab] OR behavior*[tiab] OR pattern*[tiab])) OR diet*[tiab] OR intake[tiab] OR ingestion[tiab] OR suppl*[tiab] OR consumption[tiab] OR meal*[tiab] OR nutrient*[tiab] OR nutrit*[tiab]
#3	Bowel[tiab] OR (Bowel[tiab] AND (function[tiab] OR movement*[tiab])) OR defecation[tiab] OR defaecation[tiab] OR "defecation frequency"[tiab:~2] OR "defaecation frequency"[tiab:~2] OR ((gastrointestin*[tiab] OR intestin*[tiab] OR abdomin*[tiab]) AND (stasis[tiab] OR symptom*[tiab] OR transit*[tiab] OR transit time[tiab] OR discomfort[tiab] OR bloating[tiab] OR pain[tiab])) OR gut motility[tiab] OR stool consistency[tiab] OR improving defaecation[tiab] OR improving defecation[tiab] OR borborygmi[tiab] OR stool*[tiab] OR feces[tiab] OR faeces[tiab] OR "faecal impaction"[tiab:~3] OR "fecal impaction"[tiab:~3] OR colon spasm*[tiab] OR bloating[tiab] OR constipation[tiab] OR flatulence[tiab] OR constipation[mesh] OR flatulence[mesh] OR gastrointestinal microbiome[mesh] OR gastrointestinal motility[mesh] OR gastrointestinal

	transit[mesh] OR gastrointestinal tract[mesh] OR gastrointestinal disease/prevention[mesh] OR intestines[mesh] OR defecation[mesh] OR feces[mesh] OR intestinal elimination[mesh] OR fecal impaction[mesh]
#4	#1 AND #2 AND #3
#5	"Diet Surveys"[Mesh] OR "Cohort Studies"[Mesh] OR cohort*[Tiab] OR prospective[Tiab] OR longitudinal[Tiab]
#6*	Randomized Controlled Trial[Publication Type] OR Controlled Clinical Trial[Publication Type] OR Pragmatic Clinical Trial[Publication Type] OR Clinical Study[Publication Type] OR Adaptive Clinical Trial[Publication Type] OR Equivalence Trial[Publication Type] OR Clinical Trial[Publication Type] OR Clinical Trial, Phase I[Publication Type] OR Clinical Trial, Phase II[Publication Type] OR Clinical Trial, Phase III[Publication Type] OR Clinical Trial, Phase IV[Publication Type] OR Clinical Trial Protocol[Publication Type] OR multicenter study[Publication Type] OR "Clinical Studies as Topic"[Mesh] OR "Clinical Trials as Topic"[Mesh] OR "Clinical Trial Protocols as Topic"[Mesh] OR "Multicenter Studies as Topic"[Mesh] OR "Random Allocation"[Mesh] OR "Double-Blind Method"[Mesh] OR "Single-Blind Method"[Mesh] OR "Placebos"[Mesh:NoExp] OR "Control Groups"[Mesh] OR "Cross-Over Studies"[Mesh] OR random*[Title/Abstract] OR sham[Title/Abstract] OR placebo*[Title/Abstract] OR ((singl*[Title/Abstract] OR doubl*[Title/Abstract]) AND (blind*[Title/Abstract] OR dumm*[Title/Abstract] OR mask*[Title/Abstract])) OR ((tripl*[Title/Abstract] OR trebl*[Title/Abstract]) AND (blind*[Title/Abstract] OR dumm*[Title/Abstract] OR mask*[Title/Abstract])) OR "control study"[tiab:~3] OR "control studies"[tiab:~3] OR "control group"[tiab:~3] OR "control groups"[tiab:~3] OR "healthy volunteers"[tiab:~3] OR "control trial"[tiab:~3] OR "control trials"[tiab:~3] OR "controlled study"[tiab:~3] OR "controlled trial"[tiab:~3] OR "controlled studies"[tiab:~3] OR "controlled trials"[tiab:~3] OR "clinical study"[tiab:~3] OR "clinical studies"[tiab:~3] OR "clinical trial"[tiab:~3] OR "clinical trials"[tiab:~3] OR Nonrandom*[Title/Abstract] OR non random*[Title/Abstract] OR non-random*[Title/Abstract] OR quasi-random*[Title/Abstract] OR quasirandom*[Title/Abstract] OR "phase study"[tiab:~3] OR "phase studies"[tiab:~3] OR "phase trial"[tiab:~3] OR "phase trials"[tiab:~3] OR "crossover study"[tiab:~3] OR "crossover studies"[tiab:~3] OR "crossover trial"[tiab:~3] OR "crossover trials"[tiab:~3] OR "cross-over study"[tiab:~3] OR "cross-over studies"[tiab:~3] OR "cross-over trial"[tiab:~3] OR "cross-over trials"[tiab:~3] OR ((multicent*[tiab] OR multi-cent*[tiab] OR open label[tiab] OR open-label[tiab] OR equivalence[tiab] OR superiority[tiab] OR non-inferiority[tiab] OR noninferiority[tiab] OR quasiexperimental[tiab] OR quasi-experimental[tiab]) AND (study[tiab] OR studies[tiab] OR trial*[tiab])) OR allocated[tiab] OR pragmatic study[tiab] OR pragmatic studies[tiab] OR pragmatic trial*[tiab] OR practical trial*[tiab].
#7*	"Epidemiologic Methods"[Mesh:NoExp] OR "Epidemiologic Studies"[Mesh] OR "Observational Studies as Topic"[Mesh] OR "Clinical Studies as Topic"[Mesh] OR "Single-Case Studies as Topic"[Mesh] OR "Organizational Case Studies"[Mesh] OR observational study[Publication Type] OR validation study[Publication Type] OR clinical study[Publication Type] OR case reports[Publication Type] OR "observational study"[tiab:~3] OR "observational studies"[tiab:~3] OR "observational design"[tiab:~3] OR "observational analysis"[tiab:~3] OR "observational analyses"[tiab:~3] OR ((cohort*[tiab] OR prospective[tiab] OR follow-up[tiab] OR longitudinal[tiab] OR long-term[tiab] OR retrospective[tiab]) AND (study[tiab] OR studies[tiab] OR design[tiab] OR analysis[tiab] OR analyses[tiab] OR data[tiab] OR review[tiab])) OR case control*[tiab] OR case comparison*[tiab] OR case-referent[tiab] OR "population study"[tiab:~3] OR "population studies"[tiab:~3] OR "population analysis"[tiab:~3] OR "population analyses"[tiab:~3] OR "descriptive study"[tiab:~3] OR "descriptive studies"[tiab:~3] OR "descriptive design"[tiab:~3] OR "descriptive analysis"[tiab:~3] OR "descriptive analyses"[tiab:~3] OR "multidimensional study"[tiab:~3] OR "multidimensional studies"[tiab:~3] OR "multidimensional design"[tiab:~3] OR "multidimensional analysis"[tiab:~3] OR "multidimensional analyses"[tiab:~3] OR "cross-sectional study"[tiab:~3] OR "cross-sectional studies"[tiab:~3] OR "cross-sectional design"[tiab:~3] OR "cross-sectional analysis"[tiab:~3] OR "cross-sectional analyses"[tiab:~3] OR "cross-sectional research"[tiab:~3] OR "cross-sectional survey"[tiab:~3] OR "cross-sectional findings"[tiab:~3] OR natural experiment*[tiab] OR quasi experiment*[tiab] OR "nonexperimental study"[tiab:~3] OR "nonexperimental studies"[tiab:~3] OR "nonexperimental design"[tiab:~3] OR "nonexperimental analysis"[tiab:~3] OR "nonexperimental analyses"[tiab:~3] OR "prevalence study"[tiab:~3] OR "prevalence studies"[tiab:~3] OR "prevalence analysis"[tiab:~3] OR "prevalence analyses"[tiab:~3] OR case series[tiab] OR "case report"[tiab:~3] OR "case reports"[tiab:~3] OR "case study"[tiab:~3] OR "case studies"[tiab:~3] OR "case histories"[tiab:~3]
#8*	"systematic review"
#9	#5 OR #6 OR #7 OR #8
#10	#4 AND #9
#11	#10 NOT (("Child"[Mesh] OR "Infant"[Mesh] OR "Adolescent"[Mesh]) NOT "Adult"[Mesh])

#12	#11 NOT (("Animals"[Mesh] OR "Animal Experimentation"[Mesh] OR "Models, Animal"[Mesh] OR "Vertebrates"[Mesh]) NOT ("Humans"[Mesh] OR "Human Experimentation"[Mesh]))
#13	#12 NOT ("Breast Feeding"[Majr] OR "Milk, Human"[Majr])
#14	#13 AND (English[Filter])
#15	#14 AND (("1970/01/01"[Date - Publication] : "2023/08/31"[Date - Publication]))
**Scopus**	
#1	TITLE-ABS-KEY ((ferment* OR cultur* OR leaven*) W/6 (food* OR drink* OR beverage*) OR "starter culture*") OR TITLE-ABS-KEY ((ferment* OR cultur* OR leaven*) W/2 product*) **OR** TITLE-ABS-KEY (((ferment* OR culture* OR sour*) W/6 (milk OR dairy OR cream* OR quark)) OR buttermilk OR cheese* OR yoghurt OR yogurt OR yoghourt OR yakult OR kefir OR lassi OR kumis OR koumiss OR kajmak OR airag OR ayran OR calpis OR borhani OR chal OR doogh OR kvass OR skyr OR amasi OR bouza OR butter* OR chal OR filmjolk OR kishk OR labne*) **OR** TITLE-ABS-KEY (((Ferment* OR cultur* OR cured) W/6 (meat* OR fish* OR seafood* OR shellfish OR sausage*)) OR "salami" OR "pepperoni" OR peperoni OR "chorizo" OR "cervelat" OR "mettwurst" OR "summer sausage" OR "sucuk" OR "dried meat*" OR "dried sausage*" OR "dry sausage*" OR "fish sauce*" OR "shrimp paste" OR "shrimp sauce" OR "oyster sauce" OR "prosciutto" OR "pancetta" OR "saucisson" OR sucuk) **OR** TITLE-ABS-KEY (((Ferment* OR cultur*) W/6 (fruit* OR vegetable* OR coconut* OR almond* OR hazelnut* OR nut OR cucumber* OR lemon* OR citrus OR cabbage* OR cauliflower* OR pepper* OR carrot* OR olive* OR onion* OR sago)) OR "sauerkraut" OR "table olive*" OR pickle* OR "kimchi" OR "paocai" OR torshi) **OR** TITLE-ABS-KEY (((Ferment* OR cultur*) W/6 (tea OR teas OR juice*)) OR "beer" OR "wine" OR cider* OR shochu OR kombucha* OR "pulque" OR puer OR "pu-er*" OR "fuzhuan" OR "dark tea*" OR "yellow tea*" OR coffee OR shalgam OR hardaliye) **OR** TITLE-ABS-KEY (((Ferment* OR cultur*) W/6 (soy OR soya OR bean* OR pea OR peas OR lentil* OR chickpea* OR legume* OR pulse* OR poi)) OR "soy* sauce*" OR "soybean paste*" OR miso* OR tempeh* OR tempe OR "natto" OR "doenjang" OR "doubanjiang" OR douchi OR "gochujang" OR cheonggukjang OR tsukemono OR garri) **OR** TITLE-ABS-KEY (((Ferment* OR cultur* OR leaven*) W/6 (cereal* OR grain* OR wheat* OR oat OR oats OR rice* OR millet* OR sorghum* OR maize* OR rye OR barley* OR chia OR oilseed* OR teff)) OR "bread" OR "sourdough" OR "crispbread" OR "boza" OR "ogi" OR dosa OR "tarhana" OR "buckwheat" OR "spelt" OR "einkorn" OR "quinoa" OR "amaranth" OR "tef" OR "bushera" OR chica OR chicha OR choujiu OR injera OR mahewu OR ogiri OR pozol OR ugba) **OR** TITLE-ABS-KEY (((Ferment* OR cultur*) W/6 (condiment* OR relish* OR horseradish OR dressing* OR seasoning* OR sauce* OR cocoa* OR tuber OR "acetic acid")) OR chocolate* OR vinegar* OR "tabasco" OR "sriracha" OR "Worcestershire" OR "Worcester")
#2	TITLE-ABS-KEY (((food OR *nutrient* OR eating OR nutrit*) W/6 (intake* OR habit* OR behavior* OR pattern* OR consumption OR suppl* OR ingestion)) OR diet* OR meal*)
#3	TITLE-ABS-KEY(“bowel” OR “defecation” OR “defaecation” OR “colon spasm*” OR “bloating” OR “constipation” OR “flatulence” OR “improving defaecation” OR “improving defecation” OR “borborygmi” OR “stool*” OR “feces” OR “faeces”) OR TITLE-ABS-KEY ((“gastrointestin*” OR “intestin*” OR “abdomin*”) W/2 (“stasis” OR “symptom*” OR “transit*” OR “motility” OR “microbiome” OR “discomfort” OR “pain” OR “transit time” OR “disease prevention” OR “bloating”)) OR TITLE-ABS-KEY ((“intestin*” OR “abdominal”) W/2 (“bloating” OR “flatulence” OR “pain” OR “transit time”)) OR TITLE-ABS-KEY ((“faecal” OR “fecal”) W/3 “impaction”) OR TITLE-ABS-KEY (“bowel” W/2 (“movement*” OR “function*”)) OR TITLE-ABS-KEY ((“defecation” OR “defaecation”) W/2 “frequency”)
#4	#1 AND #2 AND #3
#5*	TITLE-ABS-KEY (random* OR sham OR placebo*) OR TITLE-ABS-KEY ((singl* OR doubl*) W/1 (blind* OR dumm* OR mask*)) OR TITLE-ABS-KEY ((tripl* OR trebl*) W/1 (blind* OR dumm* OR mask*)) OR TITLE-ABS-KEY (control* W/3 (study OR studies OR trial* OR group*)) OR TITLE-ABS-KEY (clinical W/3 (study OR studies OR trial*)) OR TITLE-ABS-KEY (Nonrandom* OR "non random*" OR non-random* OR quasi-random* OR quasirandom*) OR TITLE-ABS-KEY (phase W/3 (study OR studies OR trial*)) OR TITLE-ABS-KEY ((crossover OR cross-over) W/3 (study OR studies OR trial*)) OR TITLE-ABS-KEY ((multicent* OR multi-cent*) W/3 (study OR studies OR trial*)) OR TITLE-ABS (allocated) OR TITLE-ABS-KEY (("open label" OR open-label) W/5 (study OR studies OR trial*)) OR TITLE-ABS-KEY ((equivalence OR superiority OR non-inferiority OR noninferiority) W/3 (study OR studies OR trial*)) OR TITLE-ABS-KEY ("pragmatic study" OR "pragmatic studies") OR TITLE-ABS-KEY ((pragmatic OR practical) W/3 trial*) OR TITLE-ABS-KEY ((quasiexperimental OR quasi-experimental) W/3 (study OR studies OR trial*)) OR TITLE (trial) OR KEY (trial)
#6*	TITLE-ABS-KEY (observational W/3 (study OR studies OR design OR analysis OR analyses)) OR TITLE-ABS-KEY (cohort*) OR TITLE-ABS-KEY (prospective W/7 (study

	OR studies OR design OR analysis OR analyses)) OR TITLE-ABS-KEY (("follow up" OR followup) W/7 (study OR studies OR design OR analysis OR analyses)) OR TITLE-ABS-KEY ((longitudinal OR longterm OR (long W/1 term)) W/7 (study OR studies OR design OR analysis OR analyses OR data)) OR TITLE-ABS-KEY (retrospective W/7 (study OR studies OR design OR analysis OR analyses OR data OR review)) OR TITLE-ABS-KEY ((case W/1 control) OR (case W/1 comparison) OR (case W/1 controlled)) OR TITLE-ABS-KEY (case-referent W/3 (study OR studies OR design OR analysis OR analyses)) OR TITLE-ABS-KEY (population W/3 (study OR studies OR analysis OR analyses)) OR TITLE-ABS-KEY (descriptive W/3 (study OR studies OR design OR analysis OR analyses)) OR TITLE-ABS-KEY ((multidimensional OR (multi W/1 dimensional)) W/3 (study OR studies OR design OR analysis OR analyses)) OR TITLE-ABS-KEY (cross W/1 sectional W/7 (study OR studies OR design OR research OR analysis OR analyses OR survey OR findings)) OR TITLE-ABS-KEY ((natural W/1 experiment) OR (natural W/1 experiments)) OR TITLE-ABS-KEY (quasi W/1 (experiment OR experiments OR experimental)) OR TITLE-ABS-KEY (("non experiment" OR nonexperiment OR "non experimental" OR nonexperimental) W/3 (study OR studies OR design OR analysis OR analyses)) OR TITLE-ABS-KEY (prevalence W/3 (study OR studies OR analysis OR analyses)) OR TITLE-ABS-KEY ("case series") OR TITLE-ABS-KEY (case W/3 (report OR reports OR study OR studies OR histories))
#7*	TITLE-ABS-KEY ("systematic review")
#8	#5 OR #6 OR #7
#9	#4 AND #8
#10	(KEY (animal* OR nonhuman)) AND NOT (KEY (human*))
#11	#9 AND NOT #10
#12	(KEY (infant* OR child*)) AND NOT (KEY (adult* OR aged))
#13	#11 AND NOT #12
Limits	Limit #13 to English using the language filter; Limit #13 to 1970 - 2023 using the Year filter (Range from 1970 to 2023)
**Cochrane CENTRAL**	
#1[&]	((ferment* OR cultur* OR leaven*) NEAR/6 (food* OR drink* OR beverage* OR product*) OR (starter NEXT culture*)) OR (((ferment* OR culture* OR sour*) NEAR/6 (milk OR dairy OR cream*)) OR buttermilk OR cheese* OR yoghurt OR yogurt OR yoghourt OR yakult OR quark OR kefir OR lassi OR kumis OR koumiss OR kajmak OR airag OR ayran OR calpis OR borhani OR chal OR doogh OR kvass OR skyr OR amasi OR bouza OR butter* OR chal OR filmjolk OR kishk OR labne*) OR (((Ferment* OR cultur* OR cured) NEAR/6 (meat* OR fish* OR seafood* OR shellfish OR sausage*)) OR "salami" OR "pepperoni" OR peperoni OR "chorizo" OR "cervelat" OR "mettwurst" OR "summer sausage" OR "sucuk" OR (dried NEXT meat*) OR (dried NEXT sausage*) OR (dry NEXT sausage*) OR (fish NEXT sauce*) OR "shrimp paste" OR "shrimp sauce" OR "oyster sauce" OR "prosciutto" OR "pancetta" OR "saucisson" OR sucuk) OR (((Ferment* OR cultur*) NEAR/6 (fruit* OR vegetable* OR coconut* OR almond* OR hazelnut* OR nut OR cucumber* OR lemon* OR citrus OR cabbage* OR cauliflower* OR pepper* OR carrot* OR olive* OR onion* OR sago)) OR "sauerkraut" OR (table NEXT olive*) OR pickle* OR "kimchi" OR "paocai" OR torshi) OR (((Ferment* OR cultur*) NEAR/6 (tea OR teas OR juice*)) OR "beer" OR "wine" OR cider* OR shochu OR kombucha* OR "pulque" OR puer OR pu-er* OR "fuzhuan" OR (dark NEXT tea*) OR (yellow NEXT tea*) OR coffee OR shalgam OR hardaliye) OR (((Ferment* OR cultur*) NEAR/6 (soy OR soya OR bean* OR pea OR peas OR lentil* OR chickpea* OR legume* OR pulse* OR poi)) OR (soy* NEXT sauce*) OR (soybean NEXT paste*) OR miso* OR tempeh* OR tempe OR "natto" OR "doenjang" OR "doubanjiang" OR douchi OR "gochujang" OR cheonggukjang OR tsukemono OR garri) OR (((Ferment* OR cultur* OR leaven*) NEAR/6 (cereal* OR grain* OR wheat* OR oat OR oats OR rice* OR millet* OR sorghum* OR maize* OR rye OR barley* OR chia OR oilseed* OR teff)) OR "bread" OR "sourdough" OR "crispbread" OR "boza" OR “ogi” OR dosa OR "tarhana" OR "buckwheat" OR "spelt" OR "einkorn" OR "quinoa" OR "amaranth" OR "tef" OR "bushera" OR chica OR chicha OR choujiu OR injera OR mahewu OR ogiri OR pozol OR ugba) OR (((Ferment* OR cultur*) NEAR/6 (condiment* OR relish* OR horseradish OR dressing* OR seasoning* OR sauce* OR cocoa* OR tuber OR "acetic acid")) OR chocolate* OR vinegar* OR "tabasco" OR "sriracha" OR "Worcestershire" OR "Worcester")
#2[&]	(((food OR macronutrient* OR eating) NEAR/6 (intake* OR habit* OR behavior* OR pattern*)) OR diet* OR intake OR ingestion OR suppl* OR consumption OR meal* OR nutrient* OR nutrit*)
#3[&]	(bowel OR defecation OR defaecation OR (colon NEXT spasm*) OR bloating OR constipation OR flatulence OR “improving defaecation” OR “improving defecation” OR borborygmi OR stool* OR feces OR faeces OR ((gastrointestin* OR intestin* OR abdomin*) NEAR/3 (“stasis” OR symptom* OR transit* OR motility OR microbiome OR

	discomfort OR pain OR "transit time" OR (prevent* NOT disease*) OR bloating)) OR ((intestin* OR abdominal) NEAR/3 (bloating OR flatulence OR pain OR "transit time")) OR ((faecal OR fecal) NEAR/2 impaction) OR (bowel NEAR/2 (movement* OR function*)) OR ((defecation OR defaecation) NEAR/2 frequency))
#4[&]	#1 AND #2 AND #3 in Trials and in Reviews
Limits	Choose in the field "Limits" the following options (Use results from both Cochrane Reviews and Trials for data selection): (a) Content type: "Cochrane Reviews" and "Trials"; (b) Date published on the Cochrane Library: Between "January 1970" and "August 2023"

*searches for systematic reviews and other study types were carried out separately, exclusive to each other. Once the relevant systematic reviews were retreieved, the reference lists were searched for relevant studies which were subsequently merged with other retrieved studies and de-duplicated prior to title and abstract screening.

[&]Search with filter "Title Abstract Keyword"

Supplementary Table S2. Risk of bias assessements for outcomes of interest across studies.

Study	Remarks	ROBINS-I	Outcome	Domain 1[‡]: Bias arising from the randomization process	Domain S[§]: Risk of bias arising from period and carryover effects	Domain 2[‡]: Bias due to deviations from intended interventions	Domain 3[‡]: Bias due to missing outcome data	Domain 4[‡]: Bias in measurement of the outcome	Domain 5[‡]: Bias in selection of the reported result	Overall bias[‡]
Noda 2024 (Noda et al., 2024)	RCT parallel[a]	NA	All outcomes	Low risk	NA	Low risk	Low risk	Low risk	Low risk	Low risk
Tanihiro 2024 (Tanihiro et al., 2024)	RCT parallel	NA	All outcomes	Low risk	NA	Low risk	Low risk	Low risk	Low risk	Low risk
Kaga 2024 (Kaga et al., 2024)	RCT parallel	NA	All outcomes	Low risk	NA	Some concerns	Low risk	Low risk	Low risk	Some concerns
Nagata 2015 (Nagata et al., 2016)	RCT parallel	NA	All outcomes	Low risk	NA	High risk	Low risk	Low risk	Low risk	High risk
Marteau 2013 (Marteau et al., 2013)	RCT parallel	NA	All outcomes	Low risk	NA	Low risk	Low risk	Low risk	Low risk	Low risk
Ling 1992 (Ling et al., 1992)	Repeated measures, non-randomised pilot study	Critical risk	All outcomes	NA	NA	NA	NA	NA	NA	NA
Guyonnet 2009	RCT parallel	NA	All outcomes	Low risk	NA	Low risk	Low risk	Low risk	Low risk	Low risk

(Guyonnet et al., 2009a)										
Sakai 2011 (Sakai et al., 2011)	RCT parallel	NA	All outcomes	Low risk	NA	Some concerns	Low risk	Some concerns	Low risk	Some concerns
Nemoto 2011 (Nemoto et al., 2011)	Randomised crossover trial - only first period taken	NA	All outcomes	Low risk	Low risk	Low risk	Low risk	Low risk	Low risk	Low risk
Takii 2013 (Takii et al., 2013)	Non-randomised trial	Critical risk	All outcomes	NA	NA	NA	NA	NA	NA	NA
Guyonnet 2009 (Guyonnet et al., 2009b)	RCT parallel	NA	All outcomes	Low risk	NA	Low risk	Low risk	Low risk	Low risk	Low risk
Kinoshita 2021 (Kinoshita et al., 2021)	RCT parallel	NA	All outcomes	Low risk	NA	Low risk	Low risk	Some concerns	Low risk	Some concerns
Galena 2022 (Galena et al., 2022)	RCT parallel	NA	All outcomes	Low risk	NA	Some concerns	Low risk	Low risk	Low risk	Some concerns
Guyonnet 2013 (Guyonnet et al., 2013)	RCT parallel	NA	All outcomes	Low risk	NA	Low risk	Low risk	Low risk	Low risk	Low risk
Kata-Kataoka 2016 (Kato-Kataoka et al., 2016)	RCT parallel	NA	All outcomes	Low risk	NA	Some concerns	Low risk	Low risk	Low risk	Some concerns
Matsumoto 2010 (Matsumoto et al., 2010)	RCT parallel	NA	All outcomes	Low risk	NA	Some concerns	Low risk	Low risk	Low risk	Some concerns
Spanhaak 1998 (Spanhaak et al., 1998)	RCT parallel	NA	All outcomes	Low risk	NA	High risk	Low risk	Low risk	Low risk	High risk

Meance 2003 (Meance et al., 2003)	Randomised crossover trial - only first period taken	NA	All outcomes	Low risk	Low risk	Some concerns	Low risk	Low risk	Low risk	Some concerns
Ozaki 2018 (Ozaki et al., 2018)	RCT parallel	NA	All outcomes	Low risk	NA	High risk	Low risk	Low risk	Low risk	High risk
Aslam 2022 (Aslam et al., 2022)	Observational study[b]; 4/5 selection domain, 2/2 comparability and 2/3 for outcome domain	NA	NA	NA	NA	NA	NA	NA	NA	NA
Kurahashi 2021 (Kurahashi et al., 2021)	RCT parallel	NA	All outcomes	Low risk	NA	Low risk	Low risk	Low risk	Low risk	Low risk
Tanaka 2021 (Tanaka et al., 2021)	Repeated measures, non-randomised study	Critical risk	All outcomes	NA	NA	NA	NA	NA	NA	NA
Alves 2022 (Alves et al., 2022)	Non-randomised study	Moderat e risk	All outcomes	NA	NA	NA	NA	NA	NA	NA
Koebnick 2003 (Koebnick et al., 2003)	RCT parallel	NA	All outcomes	Low risk	NA	Low risk	Low risk	Low risk	Low risk	Low risk
Takada 2016 (Takada et al., 2016)	RCT parallel	NA	All outcomes	Low risk	NA	Some concerns	Low risk	Low risk	Low risk	Some concerns
Tilley 2014 (Tilley et al., 2014)	RCT parallel	NA	All outcomes	Low risk	NA	Some concerns	Low risk	Low risk	Low risk	Some concerns

[a]Unless mentioned otherwise, all parallel group, randomised, controlled trials were assessed using the Risk of Bias 2 tool (Sterne et al., 2019).
[b]The observational study was assessed for risk of bias using the Newcastle-Ottawa scale (Wells et al., 2014).
[‡]These domains are part of the Risk of Bias 2 assessment for both randomised, controlled parallel and crossover trials.
[§]This domain is specific to the Risk of Bias 2 assessment for randomised, controlled crossover trials only.

Supplementary Table S3. Characteristics of the fermented food interventions in eligible studies included in the review*.

Study	Fermented product	FF detailed description	FF texture	Nutritional data	Production details (storage conditions, shelf-life); batch to batch variability
Noda et al., 2024 (Noda et al., 2024)	Fermented sericin-fibroin mixture	Protein mixture of sericin and fibroin, supplemented with pineapple juice, yeast extract, and skim milk	Liquid	NI[a]	One batch of 50 L
Tanihiro et al., 2024 (Tanihiro et al., 2024)	Fermented milk	Milk	Liquid	NI	NI
Kaga et al., 2024 (Kaga et al., 2024)	Fermented soymilk	Soymilk	Liquid	Moisture 85.8 g; protein 2.3 g; lipid 0.3 g; carbohydrates 11.2 g; energy 56 kcal; sodium 8.4 mg per 100 g	NI
Nagata et al., 2016 (Nagata et al., 2016)	Fermented milk	Skim milk, high-fructose corn syrup, and flavouring	Liquid	Energy 62 kcal; protein, 1.0 g; lipids, 0.1 g; carbohydrates, 14.4 g; and sodium, 15 mg per 80 mL bottle	Commercially available product
Marteau et al., 2013 (Marteau et al., 2013)	Fermented milk	Milk	Liquid	NI	NI
Ling et al., 1992 (Ling et al., 1992)	Fermented apple-peach whey drink	Whey, apricot-peach juice	Liquid	Energy 240 kJ; protein 1.4 g; fat 0.1 g; carbohydrate 12.5 g; lactose < 1.0 g per 100 mL	NI
Guyonnet et al., 2009a (Guyonnet et al., 2009a)	Fermented milk	Milk	Liquid	NI	NI
Sakai et al., 2011 (Sakai et al., 2011)	Fermented milk	Milk	Liquid	Energy 27.3 kcal/114 kJ; fat, ,0.1 g; carbohydrate, 6.6 g; protein, 0.9 g per 65 mL bottle	Refrigerated
Nemoto et al., 2011	Brown rice fermented by Aspergillus oryzae (FBRA)	Brown rice and rice bran	Granulated	Energy 88.4 kcal; Protein 3.42 g; Lipid 5.19 g; Glycol 4.47 g; Dietary fibre 5.06 g per 21 g	NI

(Nemoto et al., 2011)					
Takii et al., 2013 (Takii et al., 2013)	Fermented turnips	Turnips, long green onion, flavored soup (dried bonito extract, dried kelp extract, dried sardine extract), vegetable-based seasoning (grated ginger, sweet cooking rice wine, soy sauce, lemon juice, grated turnip)	Solid	NI	Storage at 4 °C
Guyonnet, et al., 2009b (Guyonnet et al., 2009b)	Fermented milk	Milk (added flavors: strawberry, apricots, rhubarb and figs)	Liquid	NI	Commercially available product
Kinoshita et al., 2021 (Kinoshita et al., 2021)	Yogurt	Milk	Liquid	Energy 76 kcal; Carbohydrates 13.9 g; Fat 0.67 g; Protein 3.6 g per 112 mL	Commercially available product
Galena et al., 2022 (Galena et al., 2022)	Fermented cabbage and cucumbers	1. Local producer 2. Local grocery store	Solid	Fermented cabbage and cucumbers in 36 g: Energy 5 kcal; Fat 0 g; Sodium 220 mg; Carbohydrates 0 g; Fiber 1 g; Protein 0 g. 2. Pickled cabbage or cucumbers in 30 g: Energy 5 kcal; Fat 0 g; Sodium 180 and 260 mg; Carbohydrates 1 g; Fiber 1 and 0.4 g; Protein 0 g	Commercially available product
Kato-Kataoka et al., 2016 (Kato-Kataoka et al., 2016)	Fermented milk	Milk	Liquid	Energy 62 kcal; Protein 1.4 g; Fat 0.1 g; Carbohydrate 13.9 g; Water 83.8 g per 100 mL	Stored at 0 to 10°C
Matsumoto et al., 2010 (Matsumoto et al., 2010)	Fermented milk	Sugar, defatted milk, fructose, glucose–fructose solution, soybean hemicellulose, flavoring agents and sucralose	Liquid	Energy 43 kcal; Protein 1.0 g; Lipids 0.1 g; Carbohydrates 9.4 g; Sodium 18 mg per 80 mL bottle	NI
Spanhaak et al., 1998	Fermented milk	Nonfat dry milk solids, sucrose and flavours	Liquid	3.1% non-fat dry milk solids, 17% sucrose and flavours	NI

(Spanhaak et al., 1998)					
Meance et al., 2003 (Meance et al., 2003)	Fermented milk	Milk	Liquid	Carbohydrates 4.8 g; Lipids 3.0 g; Protein 3.4 g per 100 g	Commercially available product
Ozaki et al., 2018 (Ozaki et al., 2018)	Fermented milk	Milk	Liquid	NI	NI
Aslam et al., 2022 (Aslam et al., 2022)	Hard, firm and soft cheese, fruche/fromage frais, ricotta and yogurt intake questionnaire	Milk	Various	NI	NI
Kurahashi et al., 2021 (Kurahashi et al., 2021)	Koji amazake	Rice	Liquid	Energy kcal 127.4; Protein 1.4 g; Fat 0.4 g; Carbohydrate 29.7 g; Dietary fibre 0.2 g; Moisture 86.4 g per 118 g	Commercially available product
Tanaka et al., 2021 (Tanaka et al., 2021)	Fermented *Brassica rapa*	*Brassica rapa*	Solid	0.37%. NaCl; ~0.75 g. dietary fibre per 30 g	Commercial products-contains all the production details
Alves et al., 2022 (Alves et al., 2022)	Kefir	Semi-skimmed cow milk	Liquid	Fat 1.28 ± 0.04 g; Protein 3.15 ± 0.19 g; Carbohydrates 4.91 ± 0.19 g; Lactic acid 0.6 g	NI
Koebnick et al., 2003 (Koebnick et al., 2003)	Fermented milk	Milk	Liquid	Protein 1.3 g; Fat 0.004 g; Carbohydrates 18.0 g per 100 mL and 580 mg of lactic acid	NI
Takada et al., 2016 (Takada et al., 2016)	Fermented milk	Sugar, powdered skim milk, fructose, stabilizer (soybean polysaccharide), and flavouring	Liquid	NI	Refrigerated below 10°C
Tilley et al., 2013 (Tilley et al., 2014)	Fermented milk	Milk	Liquid	Protein 1.3 g; Fat 0.004 g; Carbohydrates 18.0 g per 100 mL and 580 mg of lactic acid	NI

[a]NI: no information; *No information for fermentation type, analytical methods and quality standards used was found in any of the eligible studies.

Supplementary Table S4. Supportive evidence: relevant studies for mechanism of action

#	Study reference
1	Abdullah M, Maulahela H, Utari AP, Kusumo PD, Soebandrio A, Surono IS, et al. The role of probiotics in lowering severity of symptoms in urban women with functional constipation: a randomized double-blind controlled trial. AIP Conf Proc 2019;2155:020026.
2	Airaksinen K, Yeung N, Lyra A, Lahtinen SJ, Huttunen T, Shanahan F, et al. The effect of a probiotic blend on gastrointestinal symptoms in constipated patients: a double blind, randomised, placebo controlled 2-week trial. Benef Microbes 2019;10(6):617e27.
3	Araujo AM, Botelho PB, Ribeiro DJS, Magalhaes KG, Nakano EY, Arruda SF. A multiple-strain probiotic product provides a better enzymatic antioxidant response in individuals with constipation in a double-blind randomized controlled trial. Nutrition 2021;89:111225.
4	Araújo PG de, Abreu CSM, Donato L, Almeida J, Crippa M, Dumont A, et al. Effects of association of probiotic strains containing lactobacillus and bifidobacterium on modulation of the intestinal microbiota in constipated patients. GED Gastroenterol Endosc Dig 2017;36(3):89e98.
5	Del Piano M, Carmagnola S, Anderloni A, Andorno S, Ballare M, Balzarini M, Montino F, Orsello M, Pagliarulo M, Sartori M, et al. The use of probiotics in healthy volunteers with evacuation disorders and hard stools a double-blind, randomized, placebo-controlled study. J Clin Gastroenterol 2010;44:S30–4.
6	Dimidi E, Zdanaviciene A, Christodoulides S, Taheri S, Louis P, Duncan PI, et al. Randomised clinical trial: bifidobacterium lactis NCC2818 probiotic vs placebo, and impact on gut transit time, symptoms, and gut microbiology in chronic constipation. Aliment Pharmacol Ther 2019;49(3):251e64.
7	Favretto DC, Pontin B, Moreira TR. Effect of the consumption of a cheese enriched with probiotic organisms (Bifidobacterium lactis bi-07) in improving symptoms of constipation. Arq Gastroenterol 2013; 50:196–201.
8	He M, Hu G, Yang Y. Effect of probiotic yogurt containing Bifidobacterium animalis strain DN-173 010 on symptoms of constipation. [Chinese]. Chin J Gastroenterol 2009;14:287–9.
9	Ibarra A, Latreille-Barbier M, Donazzolo Y, Pelletier X, Ouwehand AC. Effects of 28-day Bifidobacterium animalis subsp. lactis HN019 supplementation on colonic transit time and gastrointestinal symptoms in adults with functional constipation: a double-blind, randomized, placebo-controlled, and dose ranging trial. Gut Microb 2018;9(3):236e51.
10	Ishizuka A, Tomizuka K, Aoki R, Nishijima T, Saito Y, Inoue R, Ushida K, Mawatari T, Ikeda T. Effects of administration of Bifidobacterium animalis subsp. lactis GCL2505 on defecation frequency and bifidobacterial microbiota composition in humans. J Biosci Bioeng 2012;113:587–91.
11	Kang S, Park MY, Brooks I, Lee J, Kim SH, Kim JY, et al. Spore-forming Bacillus coagulans SNZ 1969 improved intestinal motility and constipation perception mediated by microbial alterations in healthy adults with mild intermittent constipation: a randomized controlled trial. Food Res Int 2021;146:110428.
12	Kommers MJ, Silva Rodrigues RA, Miyajima F, Zavala Zavala AA, Ultramari VRLM, Fett WCR, et al. Effects of probiotic use on quality of life and physical activity in constipated female university students: a randomized, double-blind placebo-controlled study. J Alternative Compl Med 2019;25(12):1163e71.
13	Krammer HJ, von Seggern H, Schaumburg J, Neumer F. Effect of Lactobacillus casei Shirota on colonic transit time in patients with chronic constipation. Coloproctology 2011;33:109–13.
14	Madempudi RS, Neelamraju J, Ahire JJ, Gupta SK, Shukla VK. Bacillus coagulans Unique IS2 in constipation: a double-blind, placebo-controlled study. Probiotics Antimicrob Proteins 2020;12(2):335e42.
15	Martoni CJ, Evans M, Chow CT, Chan LS, Leyer G. Impact of a probiotic product on bowel habits and microbial profile in participants with functional constipation: a randomized controlled trial. J Dig Dis 2019;20(9):435e46.
16	Mazlyn MM, Nagarajah LH, Fatimah A, Norimah AK, Goh KL. Effects of a probiotic fermented milk on functional constipation: a randomized, double-blind, placebo-controlled study. J Gastroenterol Hepatol 2013;28:1141–7.
17	Minamida K, Nishimura M, Miwa K, Nishihira J. Effects of dietary fiber with Bacillus coagulans lilac-01 on bowel movement and fecal properties of healthy volunteers with a tendency for constipation. Biosci Biotechnol Biochem 2015;79(2):300e6.
18	Mollenbrink M, Bruckschen E. [Treatment of chronic constipation with physiologic Escherichia coli bacteria. Results of a clinical study of the effectiveness and tolerance of microbiological therapy with the E. coli Nissle 1917 strain (Mutaflor).] Med Klin 1994;89:587–93 (in German).
19	Moreira T, Lagemann M, Silva F. Evaluation of consumption yogurt with probiotics (lactobacillus acidophilus e bifidobacterium animalis) in the symptoms of constipation: randomized clinical trial. Rev Espanola Nutr Humana Diet_etica 2016;20(Supplement 1):471.
20	Moreira TR, Leonhardt D, Conde SR. Influence of Drinking a probiotic fermented milk beverage containing bifidobacterium animalis on the symptoms of constipation. Arq Gastroenterol 2017;54(3):206e10.

21	Ojetti V, Ianiro G, Tortora A, Bruno G, Laterza L, Gigante G, Ponziani FR, Gammarita G, Gasbarrini A. Lactobacillus reuteri (DSM 17938) for the treatment of functional constipation in adult patients: a double-blind, randomised, placebo-controlled trial. Dig Liver Dis 2013;45:S132 (abstr).
22	Ojetti V, Ianiro G, Tortora A, D'Angelo G, di Rienzo TA, Bibbo S, et al. The effect of Lactobacillus reuteri supplementation in adults with chronic functional constipation: a randomized, double-blind, placebo-controlled trial. J Gastrointestin Liver Dis 2014;23(4):387e91.
23	Riezzo G, Orlando A, D'Attoma B, Guerra V, Valerio F, Lavermicocca P, De Candia S, Russo F. Randomised clinical trial: efficacy of Lactobacillus paracasei-enriched artichokes in the treatment of patients with functional constipation—a double-blind, controlled, crossover study. Aliment Pharmacol Ther 2012;35:441–50.
24	Takeda T, Asaoka D, Nojiri S, Yanagisawa N, Nishizaki Y, Osada T, et al. 106: THE usefulness of bifidobacterium longum BB536 intake in elderly patients with chronic constipation: a prospective randomized, double-blind trial. Gastroenterology 2022;162(7, Supplement). S-19-S-20.
25	Takii H, Nishijima T, Takami K, Tanaka Y, Inugami M, Mawatari T, Sugimura H, Aoki R. Effects of fermented milk containing Bifidobacterium animalis subsp. lactis GCL2505 on improvement of defecation, fecal properties, and intestinal microflora. Jpn Pharmacol Ther2012;40:657–65.
26	Tallyne de Aguiar Silva A, Lima Cavalcanti ID, Ayanny de Lima Fernandes M, Gisele de Oliveira Coimbra C, Manoella de Souza Lima G. Effect of zymomonas mobilis probiotic on cholesterol and its lipoprotein fractions and the intestinal regulation. Lin Nutr 2020;39(12):3750e5.
27	Waller PA, Gopal PK, Leyer GJ, Ouwehand AC, Reifer C, Stewart ME, Miller LE. Dose-response effect of Bifidobacterium lactis HN019 on whole gut transit time and functional gastrointestinal symptoms in adults. Scand J Gastroenterol 2011;46:1057–64.
28	Wang L, Wang L, Tian P, Wang B, Cui S, Zhao J, et al. A randomised, double blind, placebo-controlled trial of Bifidobacterium bifidum CCFM16 for manipulation of the gut microbiota and relief from chronic constipation. Food Funct 2022;13(3):1628e40.
29	Yang YX, He M, Hu G, Wei J, Pages P, Yang XH, Bourdu-Naturel S. Effect of a fermented milk containing Bifidobacterium lactis DN-173010 on Chinese constipated women. World J Gastroenterol 2008; 14:6237–43.
30	Yoon JY, Cha JM, Oh JK, Tan PL, Kim SH, Kwak MS, et al. Probiotics Ameliorate stool consistency in patients with chronic constipation: a randomized, double-blind, placebo-controlled study. Dig Dis Sci 2018 Oct;63(10):2754e64.

Supplementary Table S5. Summary of confounding variables, adverse events and compliance in included studies.

Study	Confounding variables								Adverse effects/events	Compliance
	Dietary intake	Alcohol use	Medications	Comorbidities/other relevant conditions	Indicators of nutritional status	Smoking	Physical activity	Gut microbiota assessment		
Noda et al., 2024 (Noda et al., 2024)	Subjects were instructed to maintain their ordinary life and eating habits as much as possible. They were also directed to consume a bottle (100 mL) of either the test or placebo drink daily at any time during the day for 12 weeks. Daily dated diary record forms were provided to the subjects throughout the trial period to record the contents of their meals (for 3 days before each examination/measurement), their intake of the drinks, their health conditions, and their medication.	NI[a]	Medication was one of the exclusion criteria, the study only including healthy adults.	NI	Changes in scores for skin texture and moisture, body mass index, body fat percentage, total serum immunoglobulin E (IgE), blood glucose, and serum liver function indices, specifically aspartate aminotransferase (AST), alanine aminotransferase (ALT), and γ-glutamyl transpeptidase (γ-GTP), were also analyzed. There were no statistically significant differences.	NI	NI	The 16S rRNA-based microbiota analysis was done. There were significant differences in the changes in relative abundance for 13 taxa (*Enterobacteriales* at the order level; *Porphyromonadaceae*, *Streptococcaceae*, *Enterococcaceae*, *Enterobacteriaceae*, *Peptococcaceae*, and *Gemellaceae* at the family level; and *Parabacteroides*, *Streptococcus*, *Enterococcus*, *Clostridium*, *Klebsiella*, and *Anaerotruncus* at the genus level) between the two groups after treatment.	Some subjects suffered from transient diarrhoea during the study period. No other serious adverse effects and no differences between the two groups were reported	The average compliance rates, including omissions in taking daily drinks, were 94.9% and 97.6% in the test and placebo groups, respectively.
Tanihiro et al., 2024 (Tanihiro et al., 2024)	The participants were instructed to avoid supplements and health foods and to maintain their usual lifestyle. Consumption of lactic	Heavy drinking habits was an exclusion criterion.	Participants who received antibiotics during the study were excluded.	Exclusion of patients with BMI > 30.0 kg/m^2; exclusion of patients with reported severe	No significant differences were observed in baseline BMI between treatment and	Smoking was an exclusion criterion.	NI	In this study, consumption of CP790-fermented milk reduced the *Desulfobacterota* abundance	None	High compliance was attained in this study; the frequencies of CP790 and placebo

Study	Confounding variables								Adverse effects/events	Compliance
	Dietary intake	Alcohol use	Medications	Comorbidities/other relevant conditions	Indicators of nutritional status	Smoking	Physical activity	Gut microbiota assessment		
	acid drinks, oligosaccharides, dietary fibers more than once a week was prohibited. Yogurt was authorized max 2 times/week.	No details are provided on what was considered as heavy drinking.		hepatic, cardiac, renal or digestive diseases.	control groups (p = 0.97).			compared to placebo (p = 0.036) but did not change diversity. *Bacteroidota*, *Firmicutes*, *Proteobacteria* and *Actinobacteriota* changed from baseline, but did not differ significantly between treatment and control groups.		beverage consumption (%) were 99.94 ± 0.47 for CP790 and 100.00 ± 0.00 for placebo (mean ± standard deviation).
Kaga et al., 2024 (Kaga et al., 2024)	NI	NI	Inclusion criteria included no medication use. Two subjects who took antibiotics prior to sample collection were excluded from analysis.	Inclusion criteria included no disease.	BMI was exactly same between treatment and control groups (22.4 ± 2.8)	NI	NI	16S rRNA performed. No baseline comparison. Significant differences found between groups. *Bifidobacterium* increased in both groups but greater in FF (p < 0.001). Lactobacilli higher in FF group at endpoint vs control (p < 0.001). In FF, *Faecalibacterium* (P = 0.018) increased, Anaerovoracaceae (P = 0.009) and *Barnesiella* (P = 0.003) decreased, changed level was	NI	The self-reported compliance rate regarding the consumption of the test and placebo beverages was >99.0% in both groups.

Study	Confounding variables								Adverse effects/events	Compliance
	Dietary intake	Alcohol use	Medications	Comorbidities/other relevant conditions	Indicators of nutritional status	Smoking	Physical activity	Gut microbiota assessment		
								significantly different from placebo group. In placebo, *Clostridiaceae* (P = 0.031) and *Enterobac-teriaceae* (P = 0.023) increased and higher than FF.		
Nagata et al., 2016 (Nagata et al., 2016)	Dietary data not reported but indicated that other than consuming the beverages each day at fixed intervals, the participants did not have to alter their previous lifestyles, such as their eating or exercise habits. Moreover, during the test period, the subjects were not allowed to consume any other sources of fermented milk or oligosaccharides.	NI	outside of study metrics. Regarding the duration of antimicrobial agent and laxative use during the 6-month study period, the duration of antimicrobial use in the FF group was 5.1 ± 11.6 days, in placebo group was 8.3 ± 16.8 days. The mean duration of laxative use in FF group was 73.2 ± 82.1 days, in placebo group was 82.7 ± 79.7 days. The differences between the 2 groups were not statistically significant.	Not explicitly reported. But participants were recruited from elderly residents' facility who could not exercise on their own and who required general assistance in living.	For participants (elderly residents) the BMI was 19.7 ± 3.0 and 19.4 ± 3.0 in the FF and control groups respectively. For staff members, 21.7 ± 2.2 and 21.7 ± 2.8 for FF and control groups respectively.	NI	As indicated, participants were recruited from elderly residents' facility who could not exercise on their own and who required general assistance in living.	After 1, 3 and 6 months, numbers of *Bifidobacterium* cells higher in FF than placebo ($p < 0.05$). *Lactobacillus* higher than baseline in FF group at all time points ($p < 0.05$) but not stated if significant compared to placebo. Cell numbers of the following bacteria in the faeces were significantly lower in the LcS-fermented milk than in the placebo group at 1, 3, and 6 months after test beverage ingestion: *Clos-tridioidesdifficile, Clostridium perfringens* ,	NI	NI

Study	Confounding variables								Adverse effects/events	Compliance
	Dietary intake	Alcohol use	Medications	Comorbidities/other relevant conditions	Indicators of nutritional status	Smoking	Physical activity	Gut microbiota assessment		
								Enterobacteriaceae, *Staphylococcus*, and *Pseudomonas.*		
Marteau et al., 2013 (Marteau et al., 2013)	Throughout the study, the subjects were not allowed to consume any probiotic (including food supplements) or fermented dairy product other than those provided. They were encouraged to continue with all the other aspects of their dietary and physical exercise habits. No difference in nutrient intake at baseline between PFM and control groups. No significant changes in nutrient intake between PFM and control groups.	No significant difference in alcohol intake	Subjects recorded use of medications during the study, but no medication use reported in the publication	Inclusion criteria included no functional gastrointestinal disorder as well as significant systemic disease.	BMI of fermented foods and control groups was 22.9 ± 2.5 and 22.9 ± 2.9, respectively.	NI	Physical activity was assessed with the international physical activity questionnaires (IPAQ). No significant difference occurred between groups at baseline and during the 4-week intervention in physical activity.	NI	None	Compliance was assessed on the basis of the data reported by subjects on their diaries and of the number of non-used servings returned. Compliance was 99.7% and 99.5% for control and PFM group, respectively.
Ling et al., 1992 (Ling et al., 1992)	Fibers and liquids intake was recorded using a questionnaire. It remained unchanged during baseline, GG and follow-up periods.	NI	People using laxative were excluded. No mention of other medication.	NI	NI	NI	NI	*Lacticaseibacillus* GG was enumerated in faeces. Levels of 3 log CFU/g were identified at baseline for two individuals. During follow-up period, 5 log CFU/g were reported for two people.	NI	NI
Guyonnet et al., 2009a	No strict exclusion of other fermented foods from diet: only probiotics	NI	Exclusion in case of medication for digestive	Only women with minor digestive symptoms were	NI	NI	Participants were encouraged	NI	None	Compliance was calculated on the basis of the

Study	Confounding variables								Adverse effects/events	Compliance
	Dietary intake	Alcohol use	Medications	Comorbidities/other relevant conditions	Indicators of nutritional status	Smoking	Physical activity	Gut microbiota assessment		
(Guyonnet et al., 2009a)	and fermented dairy products were prohibited; no dietary recordings.		symptoms; exclusion when antibiotics were consumed within one month before the beginning of the study. Use of any new medications during the study was recorded in participant diaries.	included (no functional bowel disorders); any systemic disease resulted in the exclusion of the individual.			to maintain their regular activity levels. No further information is provided.			data reported by subjects in their diaries and the number of non-used servings returned. Compliance was 99.2% and 99.3% for control and PFM group, respectively.
Sakai et al., 2011 (Sakai et al., 2011)	No standardised diet nor dietary intake monitoring aside from restrictions on probiotics, prebiotics, and yogurt products.	NI	No medication was allowed, except paracetamol, oral contraceptives and hormonal replacement therapies.	Participants with chronic gastrointestinal diseases or a history of GI surgery were excluded.	Baseline BMI was 23.7 ± 3.5 and 23.9 ± 3.1 for treatment and control groups, respectively.	NI	NI	NI	NI	NI
Nemoto et al., 2011 (Nemoto et al., 2011)	Participants were instructed not to change their normal daily dietary habits.	NI	Periodic taking of antibiotic drugs is an exclusion criterion.	NI	Organic acid concentrations were measured. Concentrations of total and individual organic acids did not significantly increase after the ingestion of test food and were not significantly different between groups.	NI	NI	Gut microbiota was evaluated by using viable cell counting methods and molecular techniques (T-RFLP and Real Time PCR). Viable cell numbers of bifidobacteria and *Enterobacteriaceae* in faeces were not significantly changed after the consumption of test food in both groups. After the consumption of the	None	NI

Study	Confounding variables								Adverse effects/events	Compliance
	Dietary intake	Alcohol use	Medications	Comorbidities/other relevant conditions	Indicators of nutritional status	Smoking	Physical activity	Gut microbiota assessment		
								test food Bifidobacteria levels remained the same as before consumption.		
Takii et al., 2013 (Takii et al., 2013)	Subjects were instructed not to change their eating habits.	NI	NI	NI	ANOVA was conducted on the differences in body weight among the viable cell intake group, dead cell intake group, and placebo intake group, and no statistically significant differences were observed, except that the body weight in the viable cell intake group showed a tendency to be lower.	NI	Subjects were instructed not to change their physical activities.	NI	NI	NI
Guyonnet et al., 2009b (Guyonnet et al., 2009b)	The control group had their normal diet without intervention; subject group one consumed one pot (125 g) of the fermented milk (Activia, Danone, containing *B. lactis* DN-173 010) daily for 14 days and the	NI	Individuals having used laxatives or remedies to promote digestion and those who had consumed probiotics or the test fermented milk in the	Individuals rating their overall well-being (when considering digestive problems experienced in the previous 2 weeks) as either 'well' or	NI	NI	NI	No	None	NI

Study	Confounding variables								Adverse effects/events	Compliance
	Dietary intake	Alcohol use	Medications	Comorbidities/other relevant conditions	Indicators of nutritional status	Smoking	Physical activity	Gut microbiota assessment		
	subject group two consumed two pots of the fermented milk daily for 14 days. No dietary intake data reported beyond that.		previous two weeks were excluded. The investigator recorded medications taken during the study and any adverse events declared by participants.	'very well' at the screening visit, or with GI symptoms requiring treatment were excluded.						
Kinoshita et al., 2021 (Kinoshita et al., 2021)	All participants were prohibited from consuming other yogurt or fermented dairy products during the study period.	NI	NI	Pregnancy, allergic responses to dairy products, lactose intolerance, instructed to restrict calorie intake by a physician, history of diseases involving the immune system (e.g., rheumatism, cancer, thyroid disorder, systemic lupus erythemato-sus, myasthenia gravis, Graves' disease, scleroderm, participation in other clinical trials within the past 3 months were excluded.	At baseline, no significant difference in BMI between control and intervention groups.	NI	NI	No	None	NI

Study	Confounding variables								Adverse effects/events	Compliance
	Dietary intake	Alcohol use	Medications	Comorbidities/other relevant conditions	Indicators of nutritional status	Smoking	Physical activity	Gut microbiota assessment		
Galena et al., 2022 (Galena et al., 2022)	Women individuals who did not consume fermented vegetables on a regular basis and willing to consume fermented vegetables for six weeks were selected. Participants that were included in the treatment groups (Group A and Group B) were asked to limit consumption of sodium. Participants reported the amount of vegetable consumed in cups.	NI	Women subject who are on weight loss medication, taking antibiotics at least three months prior to enrolling, taking monoamine oxidase inhibitors, and taking anti-inflammatory medication regularly were not included in the study.	NI	Participants' height, weight, BMI and percent body fat weight were measured. No significant difference was observed in BMI within groups and between groups.	Non-smoker individuals were selected for this study.	NI	Next-generation sequencing of the V4 region of the 16S rDNA gene was done. *Firmicutes*, *Actinobacteria*, and *Bacteroidetes* were the three predominant phyla in the stool samples of participants. No significant differences were found in relative abundance of the top phyla or top 20 genera within or between groups. There was a significant increase in the Shannon index in group A compared with group C, at week 6. Also *Faecalibacterium prausnitzii* and *Roseburia faecis* were significantly enriched in group A at week 6 compared with week 0, but not in groups B or C.	Gastrointestinal symptoms were reported as adverse effects. Half of the participants in group A and 60% in group B experienced bloating with 30% of participants in the control group. Abdominal pain was reported by 40% of participants in group B and group C, compared with 18% in group A.	Participants randomized to groups A and B were also asked to fill out a log about their daily compliance to the intervention. Overall compliance for group A was 79.3% and for group B, 89.9%. No compliance data for control as the group had no intake.
Kata-Kataoka et al., 2016	Participants avoided consuming fermented milks, yogurt, lactic acid	NI	Having used medication continuously for	Participants with mental disease and/or other	Baseline BMI of the placebo group was	Habitual smoking is	NI	In both groups, the dominant phyla in the gut microbiota	NI	Daily consumption was self-

Study	Confounding variables								Adverse effects/events	Compliance
	Dietary intake	Alcohol use	Medications	Comorbidities/other relevant conditions	Indicators of nutritional status	Smoking	Physical activity	Gut microbiota assessment		
(Kato-Kataoka et al., 2016)	bacteria beverages, and probiotic or prebiotic products.		three months prior to study enrolment was one of the exclusion criteria.	diseases were excluded	20.5± 0.4 while the *Lcb. casei* Shirota group was 20.8± 0.4. General health was assessed using the Japanese version of the General Health Questionnaire-28 (GHQ-28) throughout the experimental period.	an exclusion criteria		were *Firmicutes*, *Bacteroidetes*, and *Actinobacteria*. In the placebo group, there was a tendency for the percentage of *Bacteroidetes* to increase between the baseline and pre-examination periods (P = 0.079, Wilcoxon rank-sum test), whereas no significant change was observed in the *Lcb. casei* Shirota group. Before the examination, the percentage of *Bacteroidaceae* was significantly lower in the *L. casei* strain Shirota group compared to the placebo group (P < 0.05, Mann-Whitney U test). Additionally, the number of observed species was significantly higher in the *Lcb. casei* Shirota group than in the placebo group before the examination (P <0.05), but no		recorded in a diary to check the compliance rate of consumption. The compliance rates for the consumption of placebo and *Lcb. casei* Shirota milk was 99% and 99%, respectively

Study	Confounding variables								Adverse effects/events	Compliance
	Dietary intake	Alcohol use	Medications	Comorbidities/other relevant conditions	Indicators of nutritional status	Smoking	Physical activity	Gut microbiota assessment		
								significant difference was found at baseline.		
Matsumoto et al., 2010 (Matsumoto et al., 2010)	Participants were instructed not to change their daily activities, including diet, during the trial, and both intervention and placebo beverages were matched for nutritional content.	NI	Participants were excluded if they were taking intestinal-conditioning drugs or other pharmaceuticals that might affect the gut. Medication use during the study was monitored, and participants were excluded if criteria were violated.	Participants with a history of serious diseases, severe anaemia, alcoholism, or food allergies were excluded.	BMI (FF group 24.4 ± 3.3 and placebo group 23.0 ± 3.5) and body weight were recorded and balanced between groups at baseline.	NI	Participants were instructed to maintain their usual physical activity	16S rRNA qPCR, culturing, and plate counts were used. Significant increases were noted at 2nd and 4th week of FF intake for *Bifidobacterium* (p<0.05 and p<0.01), and *Lactobacillus* (p<0.01 and p<0.01), respectively. Organic acids and putrefactive metabolites (ammonia, indole, phenol, p-cresol) were also measured.	NI	NI
Spanhaak et al., 1998 (Spanhaak et al., 1998)	All subjects (both treatment and control group) in the study received a strictly controlled diet with a constant composition of protein, fat, carbohydrates and energy.	NI	Any ongoing medication that would affect intestinal flora Immune system, haematological, biochemical parameters was excluded.	NI	NI	NI	NI	Enumeration of *Lcb. casei* Shirota, Lactobacilli, *Bifidobacteria*, *Staphylococcus, Sta-phylococcus aureus, Bacillus, Clostridium, Enterococcus* and yeasts in faecal samples was carried	None	The authors only mention compliance as being "Good".

Study	Confounding variables								Adverse effects/events	Compliance
	Dietary intake	Alcohol use	Medications	Comorbidities/other relevant conditions	Indicators of nutritional status	Smoking	Physical activity	Gut microbiota assessment		
								out. Significant increases were observed in the number of *Lcb. casei* Shirota and Bifidobacteria in the treatment group (reached 10^7 CFU/g).		
Meance et al., 2003 (Meance et al., 2003)	Subjects were asked to maintain their usual lifestyle and to follow dietary guidelines from 7 days before treatment until the end of the study, excluding other fermented dairy products and foods that could alter stool colour (e.g., carrots)	NI	Exclusion criteria included the use of antibiotics during the previous 3 months. However, level and consumption of concomitant medications were not statistically different in the studied groups (baseline demographics).	NI	No statistically significant difference was observed in the distribution of heavy, medium, and light participants between the groups (baseline demographics).	NI	There were no statistically significant differences in physical activity levels between the groups (baseline demographics).	NI	None	Compliance with treatment was monitored every 3 days by interview and by counting the amount of interventions remaining in the subject's refrigerator. All subjects complied with the intervention and consumed the correct number of servings of BM according to the protocol (compliance/95 %).
Ozaki et al., 2018 (Ozaki et al., 2018)	All participants were instructed to avoid consuming other fermented dairy products and foods likely to influence the gut	NI	Exclusion criteria included taking medicine.	NI	NI	NI	NI	NI	None	NI

Study	Confounding variables								Adverse effects/events	Compliance
	Dietary intake	Alcohol use	Medications	Comorbidities/other relevant conditions	Indicators of nutritional status	Smoking	Physical activity	Gut microbiota assessment		
	microbiota, such as oligosaccharides and dietary fiber.									
Aslam et al., 2021 (Aslam et al., 2022)	Dietary questionnaire filled, focusing on milk and dairy products intake, with total dietary fibers recording. Nevertheless, forms and calculation methods were distinct between men and women.	NI	Medication not prohibited. Impact of medication influencing gut mobility was studied using a multivariate model. However, medication consumption did not differ significantly between groups.	IBS was not an exclusion criterion, but the proportion of individuals suffering from IBS in each group did not differ significantly.	BMI was calculated. There were no significant differences between groups.	NI	Mobility level was part of the questionnaire. Distribution between active and sedentary people did not differ significantly between groups.	NI	NA (not applicable as observational study)	NA
Kurahashi et al., 2021 (Kurahashi et al., 2021)	Consumption of amazake drinks other than the test drinks and amazake-like beverages was prohibited. Participants were instructed to avoid overheating and to not change their diet	Alcohol was prohibited	Individuals under medical treatment were excluded. Only healthy individuals included.	NI	Body weight, body mass index and body fat percentage of the participants were measured.	NI	NI	Next Generation Sequencing analysis of bacterial 16S rDNA gene was performed. *Firmicutes, Actinobacteria, Bacteroidetes, Verrucomicrobia*, and *Proteobacteria* were detected as dominant phyla and members of these phyla constituted an average of 97.8% in both groups. Firmicutes to Bacteroidetes ratios decreased in the koji amazake group compared to	None	NI

Study	Confounding variables								Adverse effects/events	Compliance
	Dietary intake	Alcohol use	Medications	Comorbidities/other relevant conditions	Indicators of nutritional status	Smoking	Physical activity	Gut microbiota assessment		
								placebo. No significant changes were observed in the *Bifidobacterium* values either within the groups or between the groups.		
Tanaka et al., 2021 (Tanaka et al., 2021)	No between-group dietary intake differences reported. Participants advised to keep habitual diet unchanged. No specific intake data analysed between time points or groups.	NI	NI	Excluded if history of acute or chronic debilitating illness	Inclusion criteria BMI between 23 and 30 kg/m² and a stool frequency of 2 to 4 times per week, with no recent history of serious illness. The average BMI slightly decreased from 26.56 ± 0.5 kg/m² at baseline to 26.4 ± 0.4 kg/m² after the consumption period. Height, body weight, fat percentage, and blood pressure were measured. AST/ALT decreased significantly.	NI	NI	Microbiota assessed pre- and post-intervention using terminal-restriction fragment length polymorphism (T-RFLP) and 16S rRNA sequencing. Significant changes observed post-ingestion, including increased abundance of *Prevotella*, *Lachnospira*, and *Ruminococcaceae*. No control/placebo group; within-subject comparison only. SCFA production also increased.	NI	NI

Study	Confounding variables								Adverse effects/events	Compliance
	Dietary intake	Alcohol use	Medications	Comorbidities/other relevant conditions	Indicators of nutritional status	Smoking	Physical activity	Gut microbiota assessment		
Alves et al., 2022 (Alves et al., 2022)	Dietary intake assessed, but no between-group comparisons reported. Participants were instructed not to change their usual food intake during the study.	NI	Exclusion if retinoid treatment in the 3 months prior to the study or during the study; Antibiotic treatment in the 30 days prior to the study or during the study; Topical treatment with corticosteroids / anti-inflammatories in the study area in the 8 days prior to the study or during the study; Chronic illness involving taking regular (daily) medications such as insulin, oral antidiabetics, anti-inflammatories or immunosuppressants. Additionally, Participants were told to avoid use of laxatives and food supplements.	Exclusion criteria included systemic diseases that may impact skin conditions; atopic group had AD, rhinitis (74%), asthma (32%)	NI	NI	Participants were instructed to avoid excessive exercise during the study. No measurement or reporting of physical activity.	NI	NI	NI
Koebnick et al., 2003 (Koebnick et al., 2003)	No significant dietary intake differences reported between groups. Participants were advised not to change their habitual diet during	NI	Exclusion criteria: use of laxatives, anticholinergics, anti-diarrheal meds, or antibiotics before study.	Excluded: organic or neurological causes of constipation, milk protein allergy, and pregnancy.	BMI recorded (mean approx. 23.8 ± 2.4 kg/m² for treatment group). No other	40% of smokers in treatment group, 51% in placebo. Not used for	NI	NI	None	The authors only mention compliance as being "High".

Study	Confounding variables								Adverse effects/events	Compliance
	Dietary intake	Alcohol use	Medications	Comorbidities/other relevant conditions	Indicators of nutritional status	Smoking	Physical activity	Gut microbiota assessment		
	the study. No detailed dietary data collected.				indicators provided.	stratification.				
Takada et al., 2016 (Takada et al., 2016)	No between-group dietary intake comparison reported. Subjects were instructed to refrain from other probiotic/prebiotic products, fermented milk, and yogurt. No dietary intake data reported beyond that.	NI	Exclusion criteria included being on medication at the time of recruitment.	Excluded if they had mental disorders, milk allergy, or scored above 60 on the Self-rating Depression Scale.	BMI recorded (mean approx. 21 kg/m²), but no other nutritional biomarkers measured.	Smokers excluded from participating.	NI	No direct gut microbiome evaluation carried out. Indirect — study investigated gut–brain axis via vagal nerve activation and stress hormone modulation (*Lcb.. casei* Shirota stimulated vagal afferents and reduced cortisol).	None	Mean percentage of self-reported compliance with test beverage intake was > 96% in all trials.
Tilley et al., 2014 (Tilley et al., 2014)	No dietary intake differences between probiotic and placebo groups reported. Subjects were asked not to change dietary habits during the study. No detailed intake data provided.	NI	Exclusion included regular use of laxatives, anti-diarrheal medications, drugs with anticholinergic effect, or antibiotics in the prior 3 weeks.	Subjects with severe constipation due to organic or neurological causes were excluded.	Not directly assessed (no BMI data presented, but said that no significant difference between groups in general health or dietary habits).	Medical history questionnaire, but no data reported.	NI	No direct gut microbiome evaluation carried out. Survival of *Lcb. casei* Shirota (LcS) assessed via stool culture and ELISA. LcS detected in most Yakult group participants post-ingestion	NI	The authors only mention compliance as being “Good”.

[a]NI: no-information

Study or Subgroup	Fermented foods Mean	Fermented foods SD	Fermented foods Total	Control Mean	Control SD	Control Total	Weight	Mean difference IV, Random, 95% CI	A	B	C	D	E	F
**2.1.1 Sericin-fibroin mixture**														
Noda 2024	5.7	2.18	25	3.97	0.88	25	13.5%	1.73 [0.81 , 2.65]	+	+	+	+	+	+
**Subtotal**			**25**			**25**	**13.5%**	**1.73 [0.81 , 2.65]**						
Test for overall effect: Z = 3.68 (P = 0.0002)														
Heterogeneity: Not applicable														
**2.1.2 Milk**														
Guyonnet 2009	8.72	3.734179964	100	8.18	2.16988479	97	14.2%	0.54 [-0.31 , 1.39]	+	+	+	+	+	+
Koebnick 2003	5.75	0.24	35	5	0.47	35	20.7%	0.75 [0.58 , 0.92]	+	+	+	+	+	+
Matsumoto 2010	12.2	4.3	14	14.6	4.3	16	2.9%	-2.40 [-5.48 , 0.68]	+	?	+	+	+	?
Ozaki 2018	7.9	3.5	15	6.8	2.3	16	5.3%	1.10 [-1.00 , 3.20]	+	-	+	+	+	-
Sakai 2011	7.9	3.1	19	6	2	20	7.5%	1.90 [0.25 , 3.55]	+	?	+	+	+	?
Tanihiro 2024	5.39	1.74	57	5.37	1.63	60	16.9%	0.02 [-0.59 , 0.63]	+	+	+	+	+	+
**Subtotal (Wald[a])**			**240**			**244**	**67.5%**	**0.55 [0.10 , 1.01]**						
Test for overall effect: Z = 2.38 (P = 0.02)														
Heterogeneity: Tau² (REML[b], 95% CI) = 0.11 [0.00 , 11.13]; Chi² = 11.25, df = 5 (P = 0.05); I² = 43%														
**2.1.3 Brown rice**														
**Subtotal**			**0**			**0**		**Not estimable**						
Test for overall effect: Not applicable														
Heterogeneity: Not applicable														
**2.1.4 Turnips**														
**Subtotal**			**0**			**0**		**Not estimable**						
Test for overall effect: Not applicable														
Heterogeneity: Not applicable														
**2.1.5 Cabbage/cucumber**														
**Subtotal**			**0**			**0**		**Not estimable**						
Test for overall effect: Not applicable														
Heterogeneity: Not applicable														
**2.1.6 Koji rice**														
Kurahashi 2021	6.73	2.76	22	5.09	9.18	22	1.8%	1.64 [-2.37 , 5.65]	+	+	+	+	+	+
**Subtotal**			**22**			**22**	**1.8%**	**1.64 [-2.37 , 5.65]**						
Test for overall effect: Z = 0.80 (P = 0.42)														
Heterogeneity: Not applicable														
**2.1.7 Soy milk**														
Kaga 2024	7.01	1.57	57	7.19	1.55	55	17.3%	-0.18 [-0.76 , 0.40]	+	?	+	+	+	?
**Subtotal**			**57**			**55**	**17.3%**	**-0.18 [-0.76 , 0.40]**						
Test for overall effect: Z = 0.61 (P = 0.54)														
Heterogeneity: Not applicable														
**Total (Wald[a])**			**344**			**346**	**100.0%**	**0.60 [0.04 , 1.16]**						
95% prediction interval								[-0.74 , 1.94]						

Test for overall effect: Z = 2.09 (P = 0.04)
Test for subgroup differences: Chi² = 12.50, df = 3 (P = 0.006), I² = 76.0%
Heterogeneity: Tau² (REML[b], 95% CI) = 0.39 [0.06 , 4.74]; Chi² = 24.95, df = 8 (P = 0.002); I² = 74%

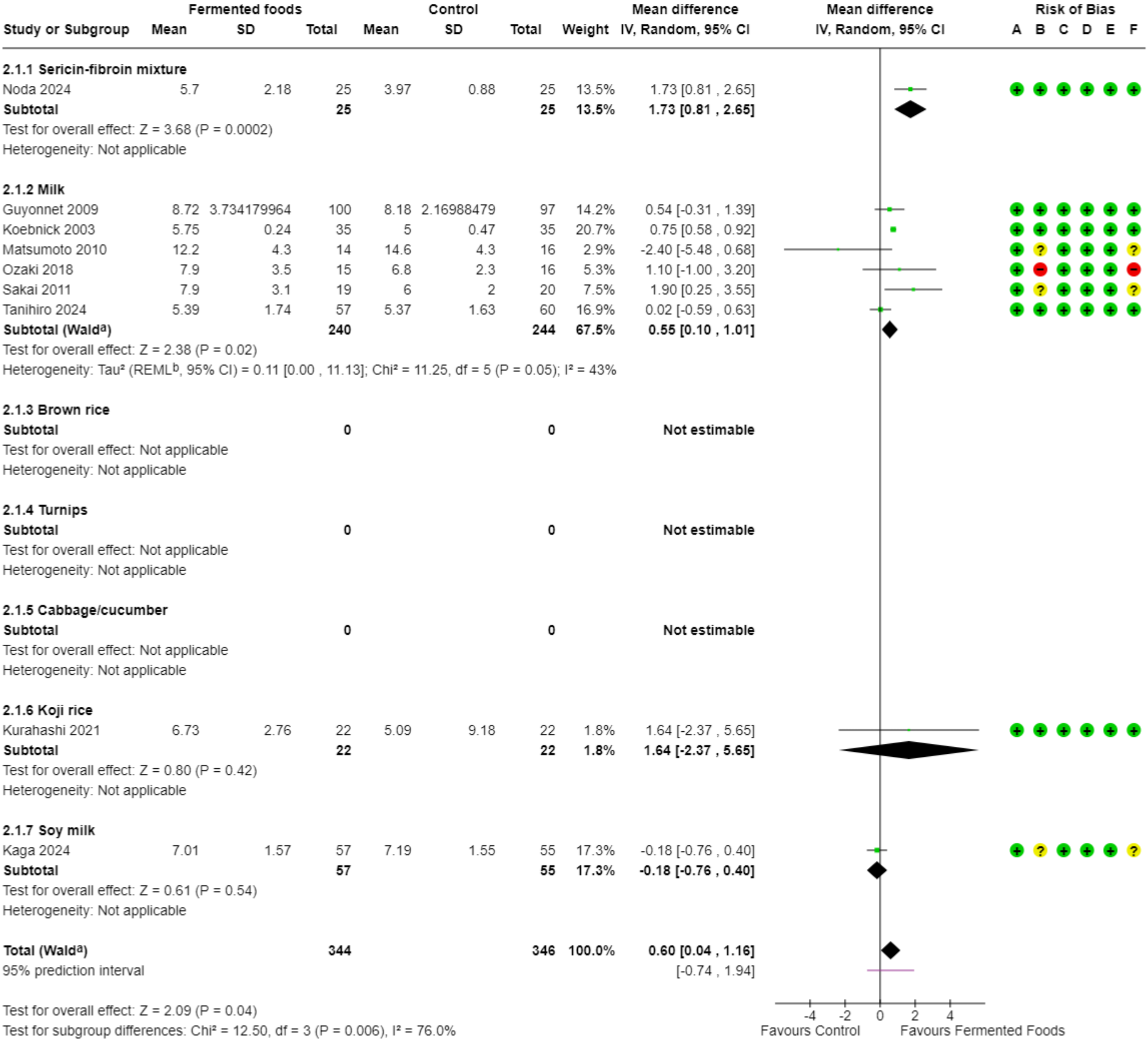

Supplementary Figure S1A. Subgroup analysis based on the type of fermentation matrix for stool frequency. Forest plot of subgroup analysis based on the type of fermentation matrix for stool frequency in randomised controlled trials comparing fermented foods with control in healthy adults (n=690). Values were calculated as mean difference (95% CIs) using a random-effects model. CI, confidence interval; IV, inverse variance; SD, standard deviation; [a]CI calculated by Wald-type method; [b]Tau^2 calculated using Restricted Maximum-Likelihood method; Risk of bias legend: (A) bias arising from the randomization process, (B) bias due to deviations from intended interventions, (C) bias due to missing outcome data, (D) bias in the measurement of the outcome, (E) bias in the selection of the reported result, (F) overall bias.

Study or Subgroup	Fermented foods Mean	Fermented foods SD	Fermented foods Total	Control Mean	Control SD	Control Total	Weight	Mean difference IV, Random, 95% CI	Risk of Bias A	B	C	D	E	F
**2.3.1 8 weeks**														
**Subtotal**			**0**			**0**		**Not estimable**						
Test for overall effect: Not applicable														
Heterogeneity: Not applicable														
**2.3.2 4 weeks**														
Guyonnet 2009	8.72	3.734179964	100	8.18	2.16988479	97	14.2%	0.54 [-0.31 , 1.39]	+	+	+	+	+	+
Kaga 2024	7.01	1.57	57	7.19	1.55	55	17.3%	-0.18 [-0.76 , 0.40]	+	?	+	+	+	?
Koebnick 2003	5.75	0.24	35	5	0.47	35	20.7%	0.75 [0.58 , 0.92]	+	+	+	+	+	+
Matsumoto 2010	12.2	4.3	14	14.6	4.3	16	2.9%	-2.40 [-5.48 , 0.68]	+	?	+	+	+	?
Ozaki 2018	7.9	3.5	15	6.8	2.3	16	5.3%	1.10 [-1.00 , 3.20]	+	−	+	+	+	−
Tanihiro 2024	5.39	1.74	57	5.37	1.63	60	16.9%	0.02 [-0.59 , 0.63]	+	+	+	+	+	+
**Subtotal (Wald[a])**			**278**			**279**	**77.3%**	**0.29 [-0.18 , 0.76]**						
Test for overall effect: Z = 1.23 (P = 0.22)														
Heterogeneity: Tau² (REML[b], 95% CI) = 0.17 [0.02 , 7.52]; Chi² = 17.10, df = 5 (P = 0.004); I² = 62%														
**2.3.3 16 weeks**														
**Subtotal**			**0**			**0**		**Not estimable**						
Test for overall effect: Not applicable														
Heterogeneity: Not applicable														
**2.3.4 6 weeks**														
**Subtotal**			**0**			**0**		**Not estimable**						
Test for overall effect: Not applicable														
Heterogeneity: Not applicable														
**2.3.5 2 weeks**														
**Subtotal**			**0**			**0**		**Not estimable**						
Test for overall effect: Not applicable														
Heterogeneity: Not applicable														
**2.3.6 3 weeks**														
Kurahashi 2021	6.73	2.76	22	5.09	9.18	22	1.8%	1.64 [-2.37 , 5.65]	+	+	+	+	+	+
Sakai 2011	7.9	3.1	19	6	2	20	7.5%	1.90 [0.25 , 3.55]	+	?	+	+	+	?
**Subtotal (Wald[a])**			**41**			**42**	**9.3%**	**1.86 [0.34 , 3.39]**						
Test for overall effect: Z = 2.40 (P = 0.02)														
Heterogeneity: Tau² (REML[b], 95% CI) = 0.00 [0.00 , 31.98]; Chi² = 0.01, df = 1 (P = 0.91); I² = 0%														
**2.3.7 12 weeks**														
Noda 2024	5.7	2.18	25	3.97	0.88	25	13.5%	1.73 [0.81 , 2.65]	+	+	+	+	+	+
**Subtotal**			**25**			**25**	**13.5%**	**1.73 [0.81 , 2.65]**						
Test for overall effect: Z = 3.68 (P = 0.0002)														
Heterogeneity: Not applicable														
**2.3.8 24 weeks**														
**Subtotal**			**0**			**0**		**Not estimable**						
Test for overall effect: Not applicable														
Heterogeneity: Not applicable														
**Total (Wald[a])**			**344**			**346**	**100.0%**	**0.60 [0.04 , 1.16]**						
95% prediction interval								[-0.74 , 1.94]						

Test for overall effect: Z = 2.09 (P = 0.04)
Test for subgroup differences: Chi² = 9.91, df = 2 (P = 0.007), I² = 79.8%
Heterogeneity: Tau² (REML[b], 95% CI) = 0.39 [0.06 , 4.74]; Chi² = 24.95, df = 8 (P = 0.002); I² = 74%

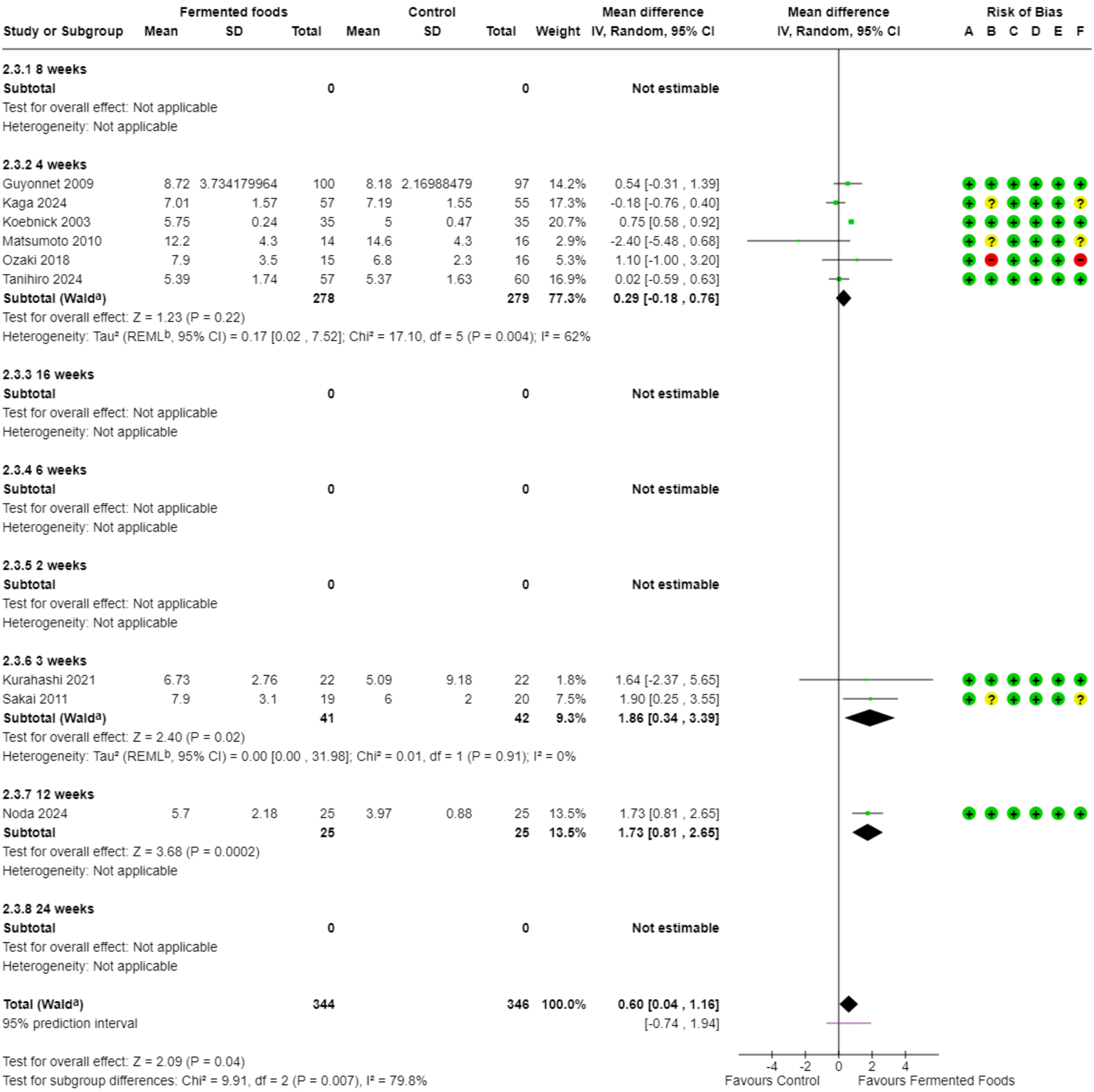

Supplementary Figure S1B. Subgroup analysis based on the intervention duration for stool frequency. Forest plot of subgroup analysis based on the intervention duration for stool frequency in randomised controlled trials comparing fermented foods with control in healthy adults (n=690). Values were calculated as mean difference (95% CIs) using a random-effects model. CI, confidence interval; IV, inverse variance; SD, standard deviation; [a]CI calculated by Wald-type method; [b]Tau[2] calculated using Restricted Maximum-Likelihood method; Risk of bias legend: (A) bias arising from the randomisation process, (B) bias due to deviations from intended interventions, (C) bias due to missing outcome data, (D) bias in the measurement of the outcome, (E) bias in the selection of the reported result, (F) overall bias.

Study or Subgroup	Fermented foods Mean	Fermented foods SD	Fermented foods Total	Control Mean	Control SD	Control Total	Weight	Mean difference IV, Random, 95% CI	A	B	C	D	E	F
**2.4.1 < 10^10 CFU/day**														
Koebnick 2003	5.75	0.24	35	5	0.47	35	20.9%	0.75 [0.58 , 0.92]	+	+	+	+	+	+
Sakai 2011	7.9	3.1	19	6	2	20	7.7%	1.90 [0.25 , 3.55]	+	?	+	+	+	?
**Subtotal (Wald[a])**			**54**			**55**	**28.6%**	**1.02 [0.06 , 1.98]**						
Test for overall effect: Z = 2.09 (P = 0.04)														
Heterogeneity: Tau² (REML[b], 95% CI) = 0.30 [0.00 , >100]; Chi² = 1.85, df = 1 (P = 0.17); I² = 46%														
**2.4.2 >= 10^10 CFU/day**														
Guyonnet 2009	8.72	3.734179964	100	8.18	2.16988479	97	14.5%	0.54 [-0.31 , 1.39]	+	+	+	+	+	+
Kaga 2024	7.01	1.57	57	7.19	1.55	55	17.5%	-0.18 [-0.76 , 0.40]	+	?	+	+	+	?
Matsumoto 2010	12.2	4.3	14	14.6	4.3	16	3.0%	-2.40 [-5.48 , 0.68]	+	?	+	+	+	?
Noda 2024	5.7	2.18	25	3.97	0.88	25	13.7%	1.73 [0.81 , 2.65]	+	+	+	+	+	+
Ozaki 2018	7.9	3.5	15	6.8	2.3	16	5.5%	1.10 [-1.00 , 3.20]	+	−	+	+	+	−
Tanihiro 2024	5.39	1.74	57	5.37	1.63	60	17.1%	0.02 [-0.59 , 0.63]	+	+	+	+	+	+
**Subtotal (Wald[a])**			**268**			**269**	**71.4%**	**0.39 [-0.36 , 1.13]**						
Test for overall effect: Z = 1.02 (P = 0.31)														
Heterogeneity: Tau² (REML[b], 95% CI) = 0.52 [0.06 , 10.57]; Chi² = 16.48, df = 5 (P = 0.006); I² = 72%														
**Total (Wald[a])**			**322**			**324**	**100.0%**	**0.58 [0.01 , 1.15]**						
95% prediction interval								[-0.78 , 1.95]						

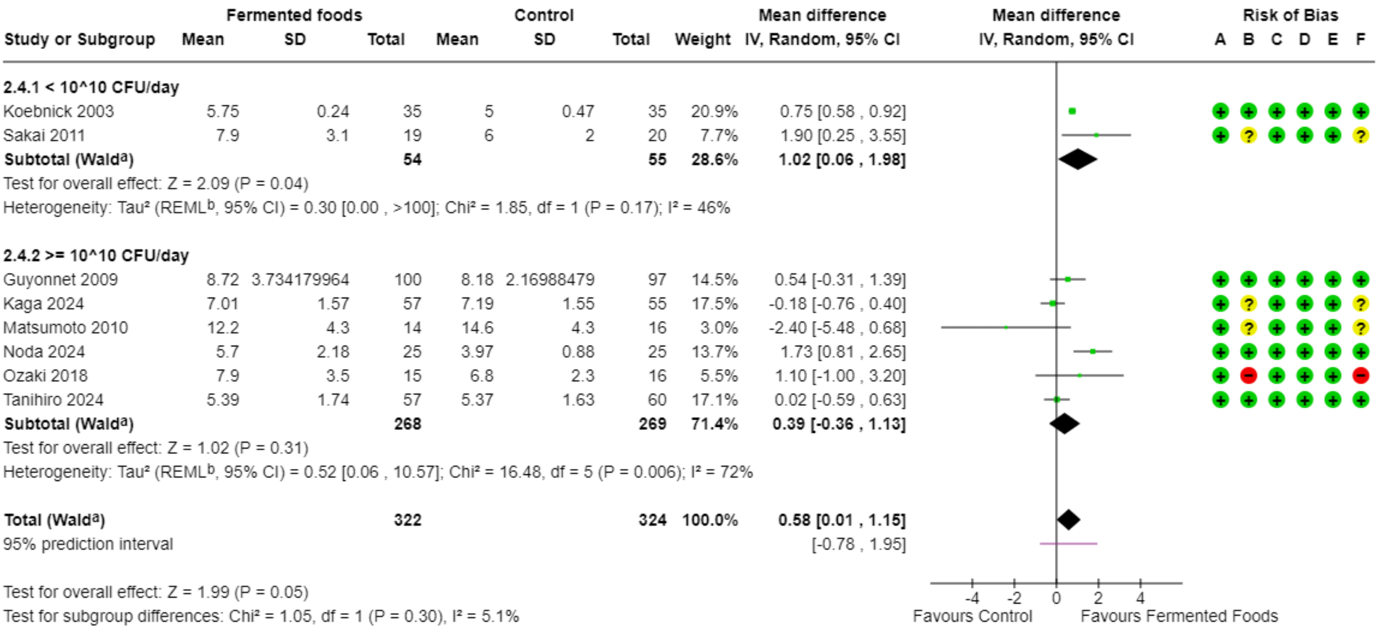

Test for overall effect: Z = 1.99 (P = 0.05)
Test for subgroup differences: Chi² = 1.05, df = 1 (P = 0.30), I² = 5.1%
Heterogeneity: Tau² (REML[b], 95% CI) = 0.40 [0.08 , 6.06]; Chi² = 24.72, df = 7 (P = 0.0008); I² = 77%

Supplementary Figure S1C. Subgroup analysis based on the microbial dosage for stool frequency. Forest plot of subgroup analysis based on the microbial dosage for stool frequency in randomized controlled trials comparing fermented foods with control in healthy adults (n=690). Values were calculated as mean difference (95% CIs) using a random-effects model. CI, confidence interval; IV, inverse variance; SD, standard deviation; [a]CI calculated by Wald-type method; [b]Tau^2 calculated using Restricted Maximum-Likelihood method; Risk of bias legend: (A) bias arising from the randomisation process, (B) bias due to deviations from intended interventions, (C) bias due to missing outcome data, (D) bias in the measurement of the outcome, (E) bias in the selection of the reported result, (F) overall bias.

Study or Subgroup	Fermented foods Mean	Fermented foods SD	Fermented foods Total	Control Mean	Control SD	Control Total	Weight	Mean difference IV, Random, 95% CI	Risk of Bias A	B	C	D	E	F
**2.2.1 Bif lactis, St thermo, Lb bulgar**														
**Subtotal**			**0**			**0**		**Not estimable**						
Test for overall effect: Not applicable														
Heterogeneity: Not applicable														
**2.2.2 Bif lactis, Lc cremoris, St thermo, Lb bulgar**														
Guyonnet 2009	8.72	3.734179964	100	8.18	2.16988479	97	14.2%	0.54 [-0.31 , 1.39]	+	+	+	+	+	+
**Subtotal**			**100**			**97**	**14.2%**	**0.54 [-0.31 , 1.39]**						
Test for overall effect: Z = 1.25 (P = 0.21)														
Heterogeneity: Not applicable														
**2.2.3 Lactobacillus helveticus CP790**														
Tanihiro 2024	5.39	1.74	57	5.37	1.63	60	16.9%	0.02 [-0.59 , 0.63]	+	+	+	+	+	+
**Subtotal**			**57**			**60**	**16.9%**	**0.02 [-0.59 , 0.63]**						
Test for overall effect: Z = 0.06 (P = 0.95)														
Heterogeneity: Not applicable														
**2.2.4 Aspergillus oryzae**														
Kurahashi 2021	6.73	2.76	22	5.09	9.18	22	1.8%	1.64 [-2.37 , 5.65]	+	+	+	+	+	+
**Subtotal**			**22**			**22**	**1.8%**	**1.64 [-2.37 , 5.65]**						
Test for overall effect: Z = 0.80 (P = 0.42)														
Heterogeneity: Not applicable														
**2.2.5 Lacticaseibacillus casei strain Shirota**														
Kaga 2024	7.01	1.57	57	7.19	1.55	55	17.3%	-0.18 [-0.76 , 0.40]	+	?	+	+	+	?
Koebnick 2003	5.75	0.24	35	5	0.47	35	20.7%	0.75 [0.58 , 0.92]	+	+	+	+	+	+
Matsumoto 2010	12.2	4.3	14	14.6	4.3	16	2.9%	-2.40 [-5.48 , 0.68]	+	?	+	+	+	?
Sakai 2011	7.9	3.1	19	6	2	20	7.5%	1.90 [0.25 , 3.55]	+	?	+	+	+	?
**Subtotal (Wald[a])**			**125**			**126**	**48.3%**	**0.37 [-0.71 , 1.46]**						
Test for overall effect: Z = 0.68 (P = 0.50)														
Heterogeneity: Tau² (REML[b], 95% CI) = 0.82 [0.08 , 44.47]; Chi² = 15.06, df = 3 (P = 0.002); I² = 87%														
**2.2.6 Lb bulgaricus OLL1073R-1, St thermophilus**														
**Subtotal**			**0**			**0**		**Not estimable**						
Test for overall effect: Not applicable														
Heterogeneity: Not applicable														
**2.2.7 COMPLEX**														
**Subtotal**			**0**			**0**		**Not estimable**						
Test for overall effect: Not applicable														
Heterogeneity: Not applicable														
**2.2.8 Lactococcus cremoris FC**														
Ozaki 2018	7.9	3.5	15	6.8	2.3	16	5.3%	1.10 [-1.00 , 3.20]	+	-	+	+	+	-
**Subtotal**			**15**			**16**	**5.3%**	**1.10 [-1.00 , 3.20]**						
Test for overall effect: Z = 1.03 (P = 0.30)														
Heterogeneity: Not applicable														
**2.2.9 Lactococcus lactis BM32-1**														
Noda 2024	5.7	2.18	25	3.97	0.88	25	13.5%	1.73 [0.81 , 2.65]	+	+	+	+	+	+
**Subtotal**			**25**			**25**	**13.5%**	**1.73 [0.81 , 2.65]**						
Test for overall effect: Z = 3.68 (P = 0.0002)														
Heterogeneity: Not applicable														
**2.2.10 Levilactobacillus brevis subsp NSB2**														
**Subtotal**			**0**			**0**		**Not estimable**						
Test for overall effect: Not applicable														
Heterogeneity: Not applicable														
**2.2.11 Bif lactis, Lc lactis, St thermo, Lb bulgar**														
**Subtotal**			**0**			**0**		**Not estimable**						
Test for overall effect: Not applicable														
Heterogeneity: Not applicable														
**Total (Wald[a])**			**344**			**346**	**100.0%**	**0.60 [0.04 , 1.16]**						
95% prediction interval								[-0.74 , 1.94]						

Test for overall effect: Z = 2.09 (P = 0.04)
Test for subgroup differences: Chi² = 9.83, df = 5 (P = 0.08), I² = 49.1%
Heterogeneity: Tau² (REML[b], 95% CI) = 0.39 [0.06 , 4.74]; Chi² = 24.95, df = 8 (P = 0.002); I² = 74%

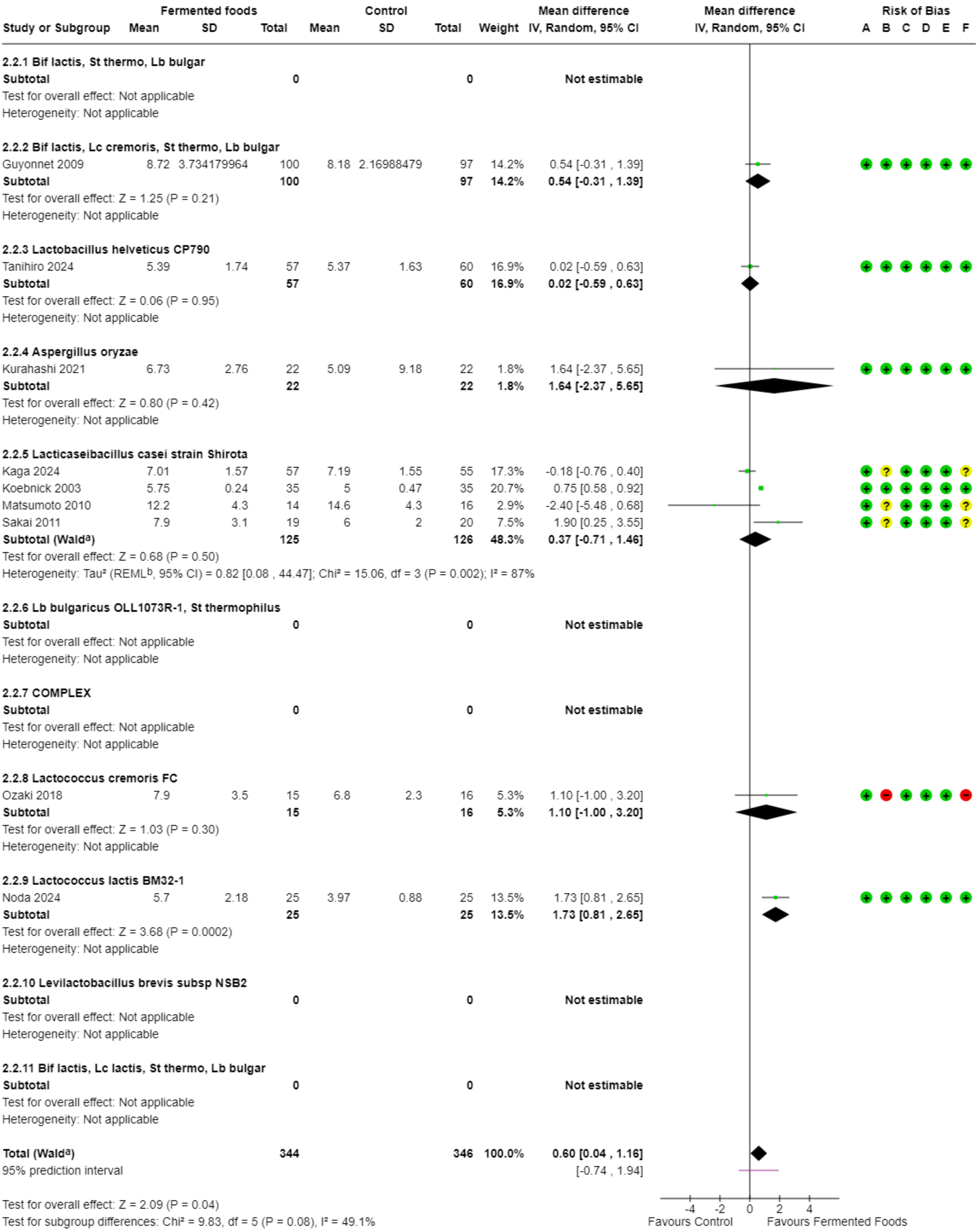

Supplementary Figure S1D. Subgroup analysis based on the fermentation microorganisms for stool frequency. Forest plot of subgroup analysis based on the fermentation microorganisms for stool frequency in randomised controlled trials comparing fermented foods with control in healthy adults (n=690). Values were calculated as mean

difference (95% CIs) using a random-effects model. CI, confidence interval; COMPLEX, complex fermenting microbial community of several microorganisms consisting primarily of lactic acid bacteria; IV, inverse variance; SD, standard deviation; [a]CI calculated by Wald-type method; [b]Tau^2 calculated using Restricted Maximum-Likelihood method; Risk of bias legend: (A) bias arising from the randomisation process, (B) bias due to deviations from intended interventions, (C) bias due to missing outcome data, (D) bias in the measurement of the outcome, (E) bias in the selection of the reported result, (F) overall bias.

Study or Subgroup	Fermented foods Mean	Fermented foods SD	Fermented foods Total	Control Mean	Control SD	Control Total	Weight	Std. mean difference IV, Random, 95% CI
**2.13.1 Sericin-fibroin mixture**								
Noda 2024	4.36	0.49	13	4.29	0.13	9	11.9%	0.17 [-0.68 , 1.03]
**Subtotal**			**13**			**9**	**11.9%**	**0.17 [-0.68 , 1.03]**
Test for overall effect: Z = 0.40 (P = 0.69)								
Heterogeneity: Not applicable								
**2.13.2 Milk**								
Guyonnet 2009	0.95	0.564003546	100	1.14	0.638435588	97	13.1%	-0.31 [-0.60 , -0.03]
Koebnick 2003	3	0.47	35	4.5	0.47	35	12.3%	-3.16 [-3.87 , -2.44]
Matsumoto 2010	2.2	0.3	14	2.8	0.5	16	12.0%	-1.39 [-2.20 , -0.58]
Sakai 2011	3.25	0.59	19	2.5	0.71	20	12.3%	1.12 [0.44 , 1.80]
Tanihiro 2024	3.86	0.53	57	3.64	0.62	60	13.0%	0.38 [0.01 , 0.74]
Tilley 2014	3.102	0.33	50	2.941	0.36	56	12.9%	0.46 [0.08 , 0.85]
**Subtotal (Wald[a])**			**275**			**284**	**75.6%**	**-0.47 [-1.70 , 0.77]**
Test for overall effect: Z = 0.74 (P = 0.46)								
Heterogeneity: Tau² (REML[b], 95% CI) = 2.29 [0.83 , 14.57]; Chi² = 109.48, df = 5 (P < 0.00001); I² = 98%								
**2.13.3 Brown rice**								
**Subtotal**			**0**			**0**		**Not estimable**
Test for overall effect: Not applicable								
Heterogeneity: Not applicable								
**2.13.4 Turnips**								
**Subtotal**			**0**			**0**		**Not estimable**
Test for overall effect: Not applicable								
Heterogeneity: Not applicable								
**2.13.5 Cabbage/cucumber**								
**Subtotal**			**0**			**0**		**Not estimable**
Test for overall effect: Not applicable								
Heterogeneity: Not applicable								
**2.13.6 Koji rice**								
Kurahashi 2021	2.97	0.4	22	3.27	0.7	22	12.5%	-0.52 [-1.12 , 0.08]
**Subtotal**			**22**			**22**	**12.5%**	**-0.52 [-1.12 , 0.08]**
Test for overall effect: Z = 1.68 (P = 0.09)								
Heterogeneity: Not applicable								
**2.13.7 Soy milk**								
**Subtotal**			**0**			**0**		**Not estimable**
Test for overall effect: Not applicable								
Heterogeneity: Not applicable								
**Total (Wald[a])**			**310**			**315**	**100.0%**	**-0.39 [-1.31 , 0.52]**
95% prediction interval								[-3.07 , 2.29]

Test for overall effect: Z = 0.84 (P = 0.40)
Test for subgroup differences: Chi² = 1.75, df = 2 (P = 0.42), I² = 0%
Heterogeneity: Tau² (REML[b], 95% CI) = 1.65 [0.66 , 7.33]; Chi² = 111.55, df = 7 (P < 0.00001); I² = 96%

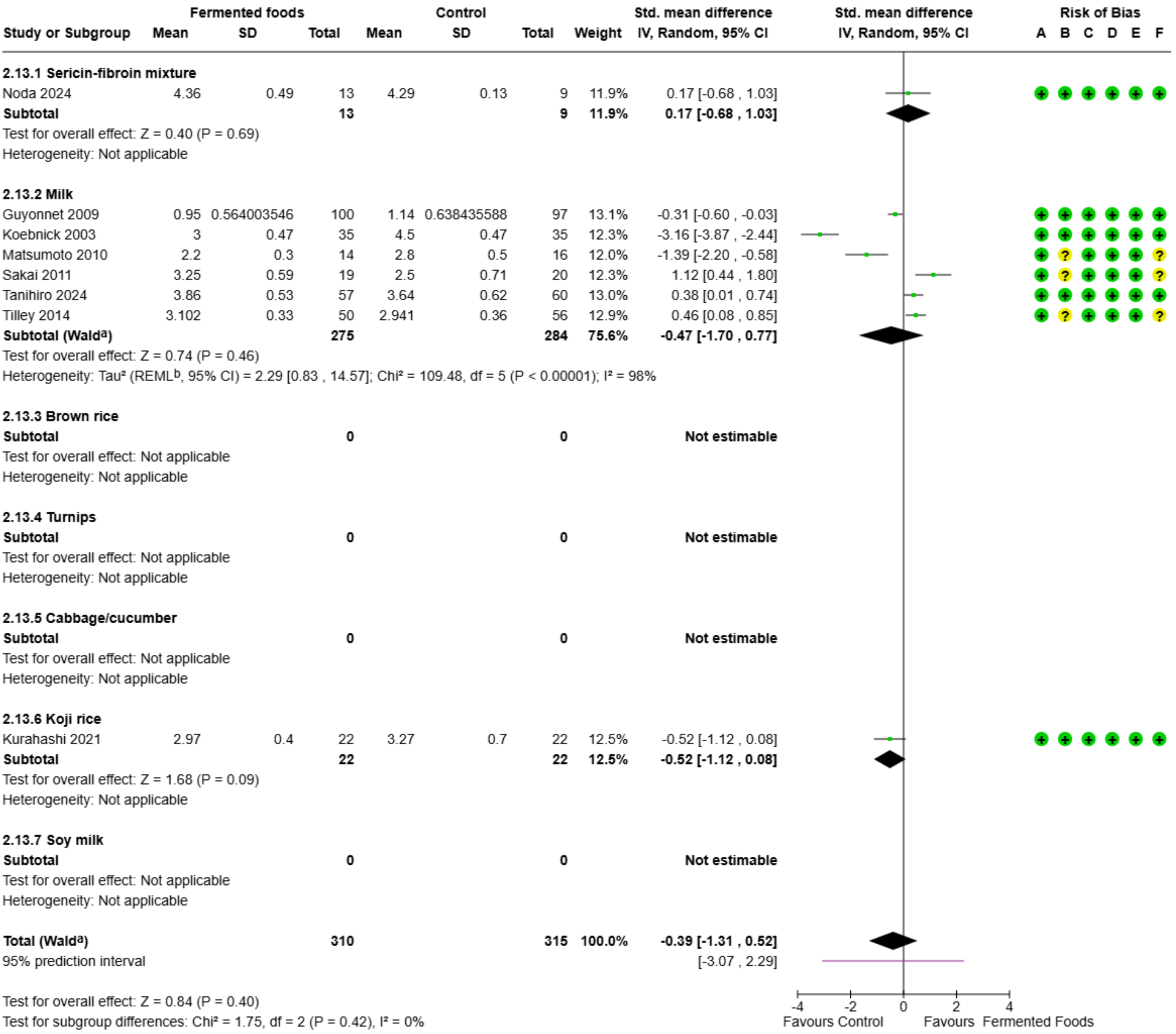

Supplementary Figure S2A. Subgroup analysis based on the fermentation matrix for stool consistency. Forest plot of subgroup analysis based on the fermentation matrix for stool consistency in randomised controlled trials comparing fermented foods with control in healthy adults (n=625). Values were calculated as standardised mean difference (95% CIs) using a random-effects model. CI, confidence interval; IV, inverse variance; SD, standard deviation; [a]CI calculated by Wald-type method; [b]Tau^2 calculated using Restricted Maximum-Likelihood method; Risk of bias legend: (A) bias arising from the randomisation process, (B) bias due to deviations from intended interventions, (C) bias due to missing outcome data, (D) bias in the measurement of the outcome, (E) bias in the selection of the reported result, (F) overall bias.

Study or Subgroup	Fermented foods Mean	Fermented foods SD	Fermented foods Total	Control Mean	Control SD	Control Total	Weight	Std. mean difference IV, Random, 95% CI	Risk of Bias A	B	C	D	E	F
**2.14.1 8 weeks**														
**Subtotal**			**0**			**0**		**Not estimable**						
Test for overall effect: Not applicable														
Heterogeneity: Not applicable														
**2.14.2 4 weeks**														
Guyonnet 2009	0.95	0.564003546	100	1.14	0.638435588	97	13.1%	-0.31 [-0.60 , -0.03]	+	+	+	+	+	+
Koebnick 2003	3	0.47	35	4.5	0.47	35	12.3%	-3.16 [-3.87 , -2.44]	+	+	+	+	+	+
Matsumoto 2010	2.2	0.3	14	2.8	0.5	16	12.0%	-1.39 [-2.20 , -0.58]	+	?	+	+	+	?
Tanihiro 2024	3.86	0.53	57	3.64	0.62	60	13.0%	0.38 [0.01 , 0.74]	+	+	+	+	+	+
Tilley 2014	3.102	0.33	50	2.941	0.36	56	12.9%	0.46 [0.08 , 0.85]	+	?	+	+	+	?
**Subtotal (Wald[a])**			**256**			**264**	**63.2%**	**-0.78 [-2.09 , 0.53]**						
Test for overall effect: Z = 1.16 (P = 0.25)														
Heterogeneity: Tau² (REML[b], 95% CI) = 2.16 [0.72 , 18.72]; Chi² = 95.72, df = 4 (P < 0.00001); I² = 98%														
**2.14.3 16 weeks**														
**Subtotal**			**0**			**0**		**Not estimable**						
Test for overall effect: Not applicable														
Heterogeneity: Not applicable														
**2.14.4 6 weeks**														
**Subtotal**			**0**			**0**		**Not estimable**						
Test for overall effect: Not applicable														
Heterogeneity: Not applicable														
**2.14.5 2 weeks**														
**Subtotal**			**0**			**0**		**Not estimable**						
Test for overall effect: Not applicable														
Heterogeneity: Not applicable														
**2.14.6 3 weeks**														
Kurahashi 2021	2.97	0.4	22	3.27	0.7	22	12.5%	-0.52 [-1.12 , 0.08]	+	+	+	+	+	+
Sakai 2011	3.25	0.59	19	2.5	0.71	20	12.3%	1.12 [0.44 , 1.80]	+	?	+	+	+	?
**Subtotal (Wald[a])**			**41**			**42**	**24.9%**	**0.29 [-1.31 , 1.90]**						
Test for overall effect: Z = 0.36 (P = 0.72)														
Heterogeneity: Tau² (REML[b], 95% CI) = 1.24 [0.16 , >100]; Chi² = 12.51, df = 1 (P = 0.0004); I² = 92%														
**2.14.7 12 weeks**														
Noda 2024	4.36	0.49	13	4.29	0.13	9	11.9%	0.17 [-0.68 , 1.03]	+	+	+	+	+	+
**Subtotal**			**13**			**9**	**11.9%**	**0.17 [-0.68 , 1.03]**						
Test for overall effect: Z = 0.40 (P = 0.69)														
Heterogeneity: Not applicable														
**2.14.8 24 weeks**														
**Subtotal**			**0**			**0**		**Not estimable**						
Test for overall effect: Not applicable														
Heterogeneity: Not applicable														
**Total (Wald[a])**			**310**			**315**	**100.0%**	**-0.39 [-1.31 , 0.52]**						
95% prediction interval								[-3.07 , 2.29]						

Test for overall effect: Z = 0.84 (P = 0.40)
Test for subgroup differences: Chi² = 1.62, df = 2 (P = 0.44), I² = 0%
Heterogeneity: Tau² (REML[b], 95% CI) = 1.65 [0.66 , 7.33]; Chi² = 111.55, df = 7 (P < 0.00001); I² = 96%

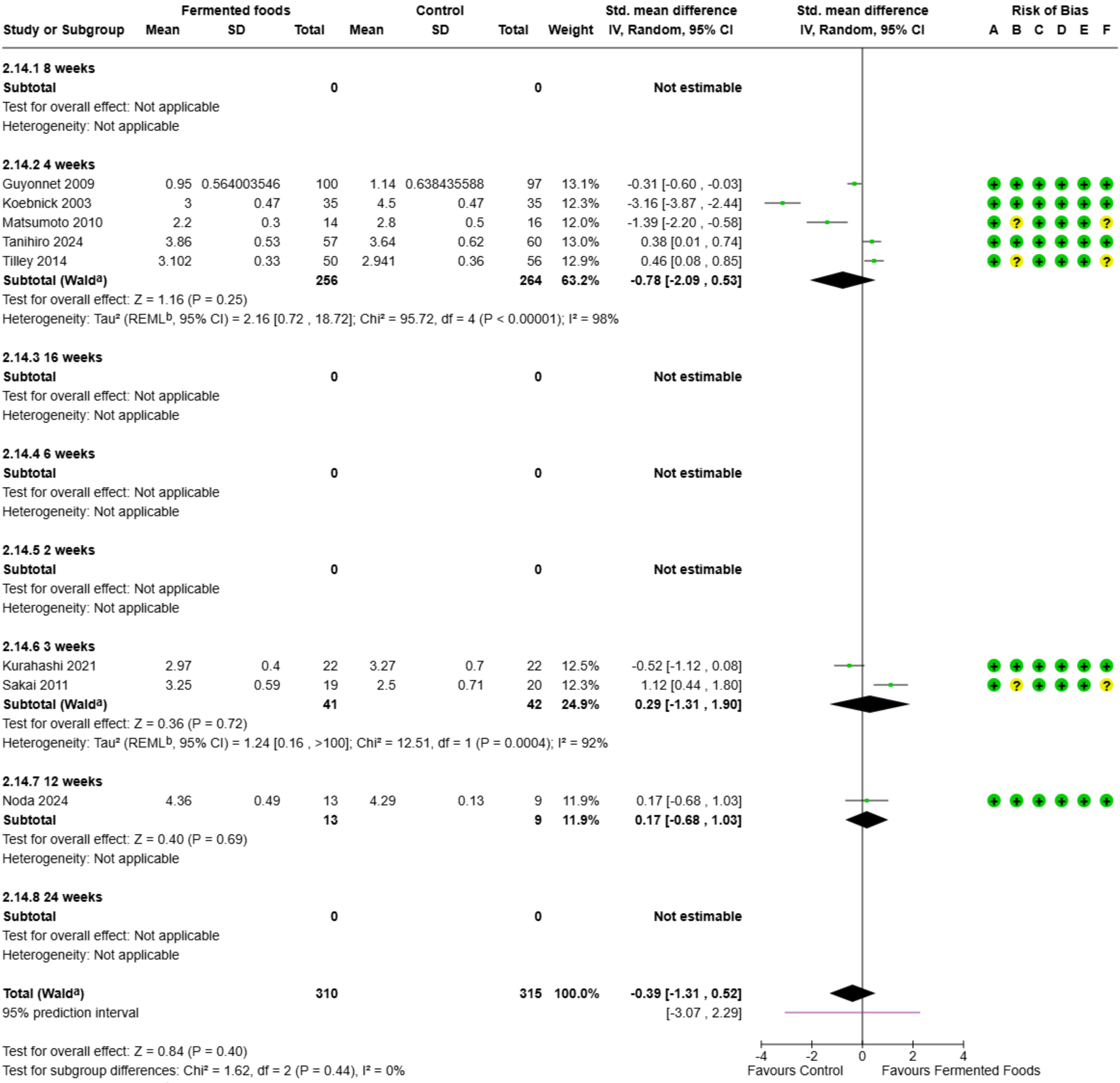

Supplementary Figure S2B. Subgroup analysis based on the intervention duration for stool consistency. Forest plot of subgroup analysis based on the intervention duration for stool consistency in randomised controlled trials comparing fermented foods with control in healthy adults (n=625). Values were calculated as standardised mean difference (95% CIs) using a random-effects model. CI, confidence interval; IV, inverse variance; SD, standard deviation; [a]CI calculated by Wald-type method; [b]Tau^2 calculated using Restricted Maximum-Likelihood method; Risk of bias legend: (A) bias arising from the randomisation process, (B) bias due to deviations from intended interventions, (C) bias due to missing outcome data, (D) bias in the measurement of the outcome, (E) bias in the selection of the reported result, (F) overall bias.

Study or Subgroup	Fermented foods Mean	Fermented foods SD	Fermented foods Total	Control Mean	Control SD	Control Total	Weight	Std. mean difference IV, Random, 95% CI	A	B	C	D	E	F
**2.15.1 < 10^10 CFU/day**														
Koebnick 2003	3	0.47	35	4.5	0.47	35	14.1%	-3.16 [-3.87 , -2.44]	+	+	+	+	+	+
Sakai 2011	3.25	0.59	19	2.5	0.71	20	14.1%	1.12 [0.44 , 1.80]	+	?	+	+	+	?
Tilley 2014	3.102	0.33	50	2.941	0.36	56	14.7%	0.46 [0.08 , 0.85]	+	?	+	+	+	?
**Subtotal (Wald[a])**			**104**			**111**	**42.9%**	**-0.52 [-3.11 , 2.08]**						
Test for overall effect: Z = 0.39 (P = 0.70)														
Heterogeneity: Tau² (REML[b], 95% CI) = 5.16 [1.32 , >100]; Chi² = 90.92, df = 2 (P < 0.00001); I² = 98%														
**2.15.2 >= 10^10 CFU/day**														
Guyonnet 2009	0.95	0.564003546	100	1.14	0.638435588	97	14.9%	-0.31 [-0.60 , -0.03]	+	+	+	+	+	+
Matsumoto 2010	2.2	0.3	14	2.8	0.5	16	13.8%	-1.39 [-2.20 , -0.58]	+	?	+	+	+	?
Noda 2024	4.36	0.49	13	4.29	0.13	9	13.7%	0.17 [-0.68 , 1.03]	+	+	+	+	+	+
Tanihiro 2024	3.86	0.53	57	3.64	0.62	60	14.7%	0.38 [0.01 , 0.74]	+	+	+	+	+	+
**Subtotal (Wald[a])**			**184**			**182**	**57.1%**	**-0.25 [-0.98 , 0.47]**						
Test for overall effect: Z = 0.68 (P = 0.49)														
Heterogeneity: Tau² (REML[b], 95% CI) = 0.45 [0.08 , 8.56]; Chi² = 18.87, df = 3 (P = 0.0003); I² = 89%														
**Total (Wald[a])**			**288**			**293**	**100.0%**	**-0.38 [-1.44 , 0.68]**						
95% prediction interval								[-3.31 , 2.56]						

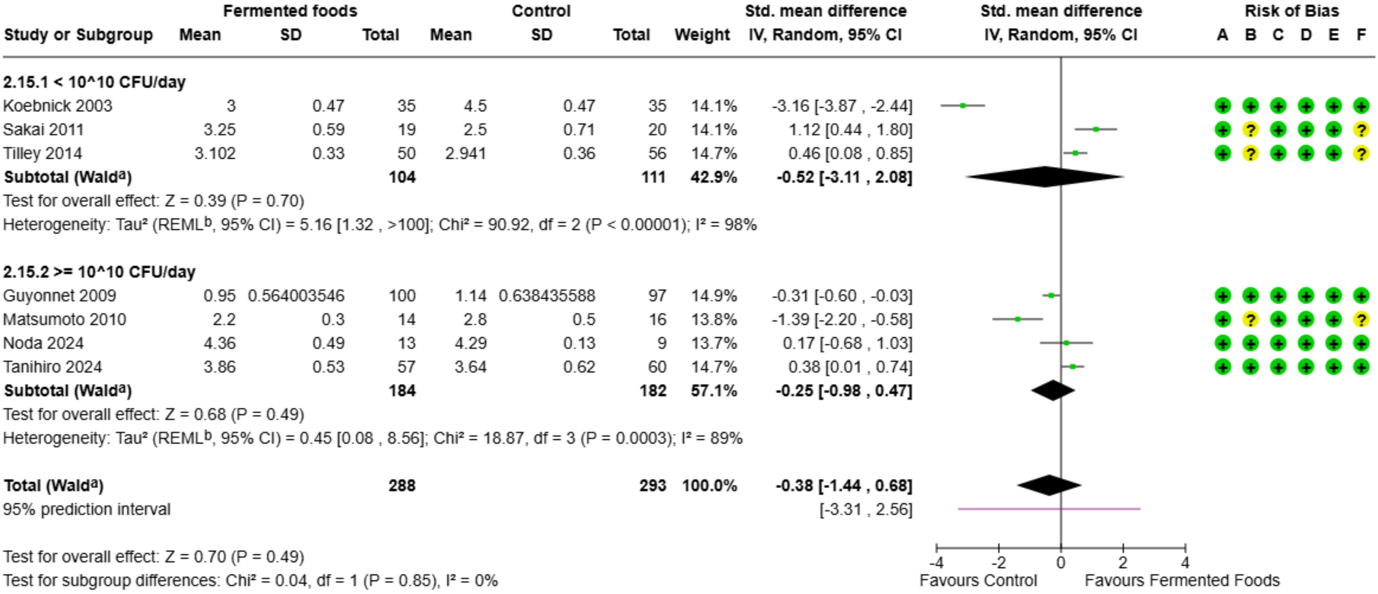

Test for overall effect: Z = 0.70 (P = 0.49)
Test for subgroup differences: Chi² = 0.04, df = 1 (P = 0.85), I² = 0%
Heterogeneity: Tau² (REML[b], 95% CI) = 1.95 [0.75 , 10.05]; Chi² = 109.92, df = 6 (P < 0.00001); I² = 97%

Supplementary Figure S2C. Subgroup analysis based on the microbial dosage for stool consistency. Forest plot of subgroup analysis based on the microbial dosage for stool consistency in randomised controlled trials comparing fermented foods with control in healthy adults (n=625). Values were calculated as standardised mean difference (95% CIs) using a random-effects model. CI, confidence interval; IV, inverse variance; SD, standard deviation; [a]CI calculated by Wald-type method; [b]Tau^2 calculated using Restricted Maximum-Likelihood method; Risk of bias legend: (A) bias arising from the randomisation process, (B) bias due to deviations from intended interventions, (C) bias due to missing outcome data, (D) bias in the measurement of the outcome, (E) bias in the selection of the reported result, (F) overall bias.

Study or Subgroup	Fermented foods Mean	Fermented foods SD	Fermented foods Total	Control Mean	Control SD	Control Total	Weight	Std. mean difference IV, Random, 95% CI	A	B	C	D	E	F
**2.16.1 Bif lactis, St thermo, Lb bulgar**														
**Subtotal**			**0**			**0**		**Not estimable**						
Test for overall effect: Not applicable														
Heterogeneity: Not applicable														
**2.16.2 Bif lactis, Lc cremoris, St thermo, Lb bulgar**														
Guyonnet 2009	0.95	0.564003546	100	1.14	0.638435588	97	13.1%	-0.31 [-0.60 , -0.03]	+	+	+	+	+	+
**Subtotal**			**100**			**97**	**13.1%**	**-0.31 [-0.60 , -0.03]**						
Test for overall effect: Z = 2.19 (P = 0.03)														
Heterogeneity: Not applicable														
**2.16.3 Lactobacillus helveticus CP790**														
Tanihiro 2024	3.86	0.53	57	3.64	0.62	60	13.0%	0.38 [0.01 , 0.74]	+	+	+	+	+	+
**Subtotal**			**57**			**60**	**13.0%**	**0.38 [0.01 , 0.74]**						
Test for overall effect: Z = 2.03 (P = 0.04)														
Heterogeneity: Not applicable														
**2.16.4 Aspergillus oryzae**														
Kurahashi 2021	2.97	0.4	22	3.27	0.7	22	12.5%	-0.52 [-1.12 , 0.08]	+	+	+	+	+	+
**Subtotal**			**22**			**22**	**12.5%**	**-0.52 [-1.12 , 0.08]**						
Test for overall effect: Z = 1.68 (P = 0.09)														
Heterogeneity: Not applicable														
**2.16.5 Lacticaseibacillus casei strain Shirota**														
Koebnick 2003	3	0.47	35	4.5	0.47	35	12.3%	-3.16 [-3.87 , -2.44]	+	+	+	+	+	+
Matsumoto 2010	2.2	0.3	14	2.8	0.5	16	12.0%	-1.39 [-2.20 , -0.58]	+	?	+	+	+	?
Sakai 2011	3.25	0.59	19	2.5	0.71	20	12.3%	1.12 [0.44 , 1.80]	+	?	+	+	+	?
Tilley 2014	3.102	0.33	50	2.941	0.36	56	12.9%	0.46 [0.08 , 0.85]	+	?	+	+	+	?
**Subtotal (Wald[a])**			**118**			**127**	**49.5%**	**-0.73 [-2.62 , 1.16]**						
Test for overall effect: Z = 0.76 (P = 0.45)														
Heterogeneity: Tau² (REML[b], 95% CI) = 3.61 [1.08 , 51.68]; Chi² = 99.97, df = 3 (P < 0.00001); I² = 97%														
**2.16.6 Lb bulgaricus OLL1073R-1, St thermophilus**														
**Subtotal**			**0**			**0**		**Not estimable**						
Test for overall effect: Not applicable														
Heterogeneity: Not applicable														
**2.16.7 COMPLEX**														
**Subtotal**			**0**			**0**		**Not estimable**						
Test for overall effect: Not applicable														
Heterogeneity: Not applicable														
**2.16.8 Lactococcus cremoris FC**														
**Subtotal**			**0**			**0**		**Not estimable**						
Test for overall effect: Not applicable														
Heterogeneity: Not applicable														
**2.16.9 Lactococcus lactis BM32-1**														
Noda 2024	4.36	0.49	13	4.29	0.13	9	11.9%	0.17 [-0.68 , 1.03]	+	+	+	+	+	+
**Subtotal**			**13**			**9**	**11.9%**	**0.17 [-0.68 , 1.03]**						
Test for overall effect: Z = 0.40 (P = 0.69)														
Heterogeneity: Not applicable														
**2.16.10 Levilactobacillus brevis subsp NSB2**														
**Subtotal**			**0**			**0**		**Not estimable**						
Test for overall effect: Not applicable														
Heterogeneity: Not applicable														
**2.16.11 Bif lactis, Lc lactis, St thermo, Lb bulgar**														
**Subtotal**			**0**			**0**		**Not estimable**						
Test for overall effect: Not applicable														
Heterogeneity: Not applicable														
**Total (Wald[a])**			**310**			**315**	**100.0%**	**-0.39 [-1.31 , 0.52]**						
95% prediction interval								[-3.07 , 2.29]						

Test for overall effect: Z = 0.84 (P = 0.40)
Test for subgroup differences: Chi² = 11.46, df = 4 (P = 0.02), I² = 65.1%
Heterogeneity: Tau² (REML[b], 95% CI) = 1.65 [0.66 , 7.33]; Chi² = 111.55, df = 7 (P < 0.00001); I² = 96%

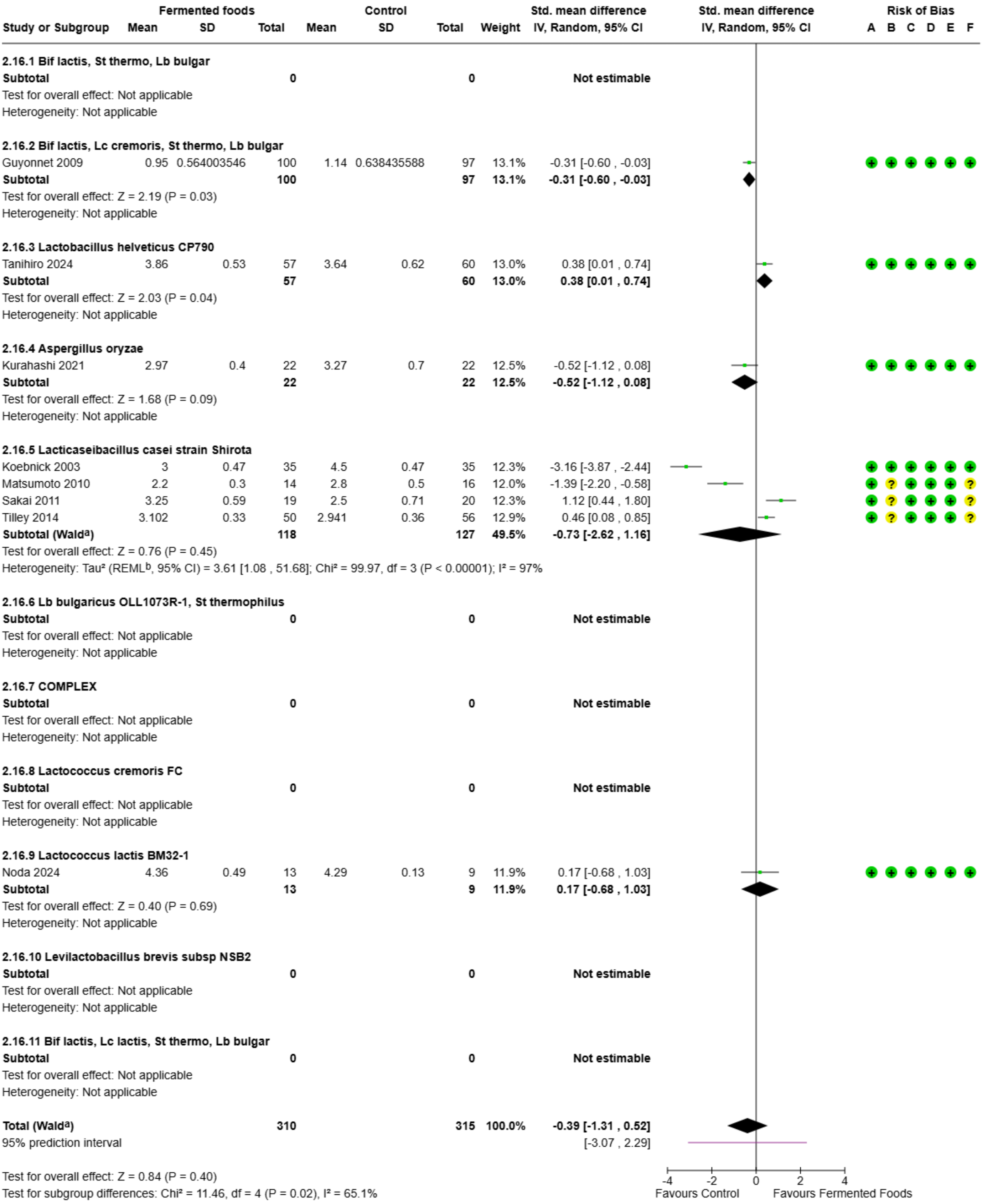

Supplementary Figure S2D. Subgroup analysis based on the fermentation microorganisms for stool consistency. Forest plot of subgroup analysis based on the fermentation microorganisms for stool consistency in randomised controlled trials comparing fermented foods with control in healthy adults (n=625). Values were calculated as standardised mean difference (95% CIs) using a random-effects model. COMPLEX, complex fermenting microbial

community of several microorganisms consisting primarily of lactic acid bacteria; CI, confidence interval; IV, inverse variance; SD, standard deviation; [a]CI calculated by Wald-type method; [b]Tau^2 calculated using Restricted Maximum-Likelihood method; Risk of bias legend: (A) bias arising from the randomisation process, (B) bias due to deviations from intended interventions, (C) bias due to missing outcome data, (D) bias in the measurement of the outcome, (E) bias in the selection of the reported result, (F) overall bias.

Study or Subgroup	Fermented foods Mean	Fermented foods SD	Fermented foods Total	Control Mean	Control SD	Control Total	Weight	Mean difference IV, Random, 95% CI	Risk of Bias A	B	C	D	E	F
**2.21.1 Sericin-fibroin mixture**														
Noda 2024	4.36	0.49	13	4.29	0.13	9	23.2%	0.07 [-0.21 , 0.35]	+	+	+	+	+	+
**Subtotal**			**13**			**9**	**23.2%**	**0.07 [-0.21 , 0.35]**						
Test for overall effect: Z = 0.49 (P = 0.62)														
Heterogeneity: Not applicable														
**2.21.2 Milk**														
Sakai 2011	3.25	0.59	19	2.5	0.71	20	16.3%	0.75 [0.34 , 1.16]	+	?	+	+	+	?
Tanihiro 2024	3.86	0.53	57	3.64	0.62	60	27.8%	0.22 [0.01 , 0.43]	+	+	+	+	+	+
Tilley 2014	3.102	0.33	50	2.941	0.36	56	32.7%	0.16 [0.03 , 0.29]	+	?	+	+	+	?
**Subtotal (Wald[a])**			**126**			**136**	**76.8%**	**0.33 [0.01 , 0.64]**						
Test for overall effect: Z = 2.05 (P = 0.04)														
Heterogeneity: Tau² (REML[b], 95% CI) = 0.06 [0.00 , 4.12]; Chi² = 7.23, df = 2 (P = 0.03); I² = 83%														
**2.21.3 Brown rice**														
**Subtotal**			**0**			**0**		**Not estimable**						
Test for overall effect: Not applicable														
Heterogeneity: Not applicable														
**2.21.4 Turnips**														
**Subtotal**			**0**			**0**		**Not estimable**						
Test for overall effect: Not applicable														
Heterogeneity: Not applicable														
**2.21.5 Cabbage/cucumber**														
**Subtotal**			**0**			**0**		**Not estimable**						
Test for overall effect: Not applicable														
Heterogeneity: Not applicable														
**2.21.6 Koji rice**														
**Subtotal**			**0**			**0**		**Not estimable**						
Test for overall effect: Not applicable														
Heterogeneity: Not applicable														
**2.21.7 Soy milk**														
**Subtotal**			**0**			**0**		**Not estimable**						
Test for overall effect: Not applicable														
Heterogeneity: Not applicable														
**Total (Wald[a])**			**139**			**145**	**100.0%**	**0.25 [0.03 , 0.47]**						
95% prediction interval								[-0.17 , 0.68]						

Test for overall effect: Z = 2.24 (P = 0.03)
Test for subgroup differences: Chi² = 1.44, df = 1 (P = 0.23), I² = 30.8%
Heterogeneity: Tau² (REML[b], 95% CI) = 0.03 [0.00 , 1.27]; Chi² = 8.15, df = 3 (P = 0.04); I² = 72%

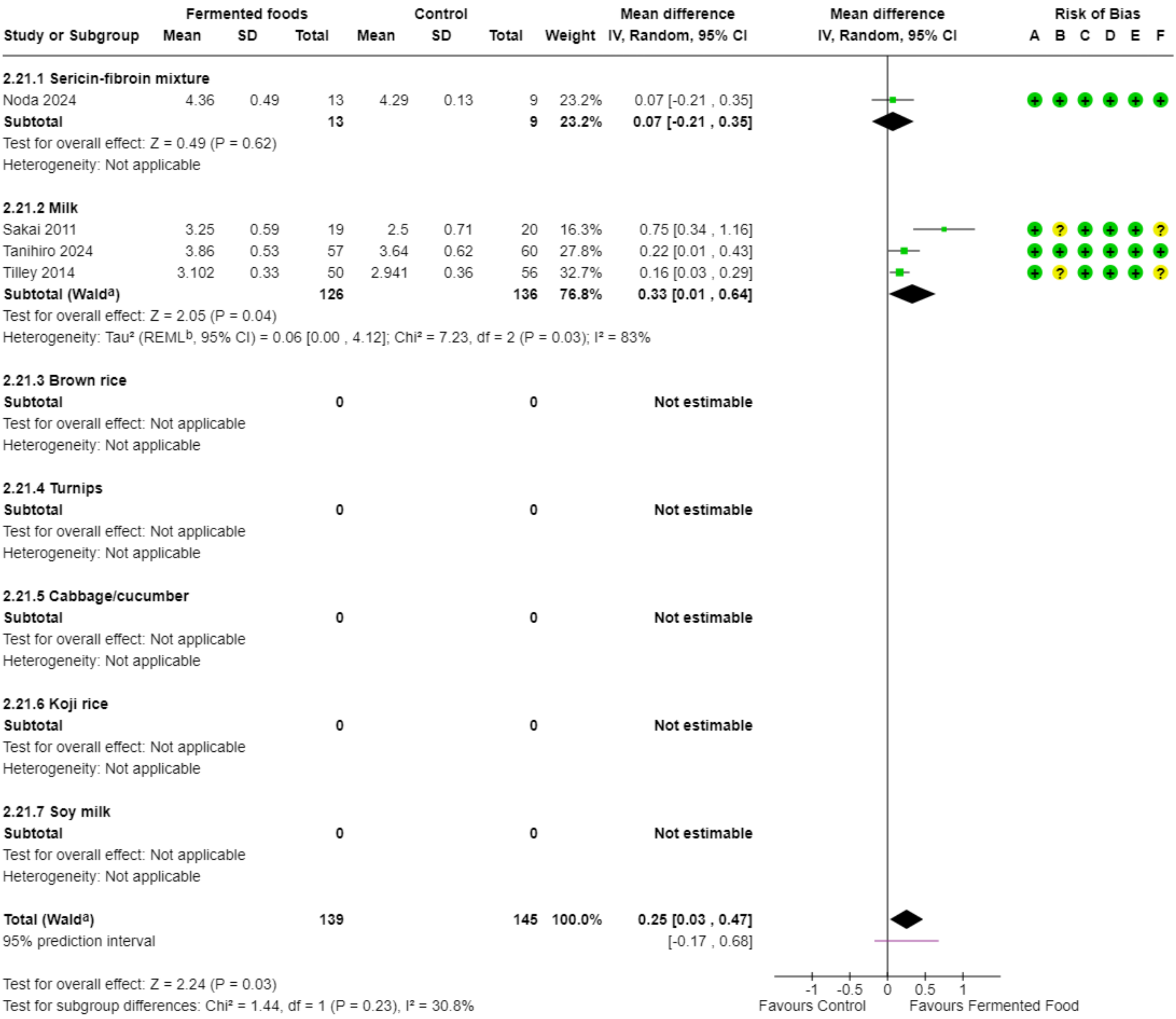

Supplementary Figure S3A. Subgroup analysis based on the fermentation matrix for stool consistency (BSFS). Forest plot of subgroup analysis based on the fermentation matrix for stool consistency (BSFS) in randomised controlled trials comparing fermented foods with control in healthy adults (n=284). Values were calculated as mean difference (95% CIs) using a random-effects model. BSFS, Bristol stool form scale; CI, confidence interval; IV, inverse variance; SD, standard deviation; [a]CI calculated by Wald-type method; [b]Tau^2 calculated using Restricted Maximum-Likelihood method; Risk of bias legend: (A) bias arising from the randomisation process, (B) bias due to deviations from intended interventions, (C) bias due to missing outcome data, (D) bias in the measurement of the outcome, (E) bias in the selection of the reported result, (F) overall bias.

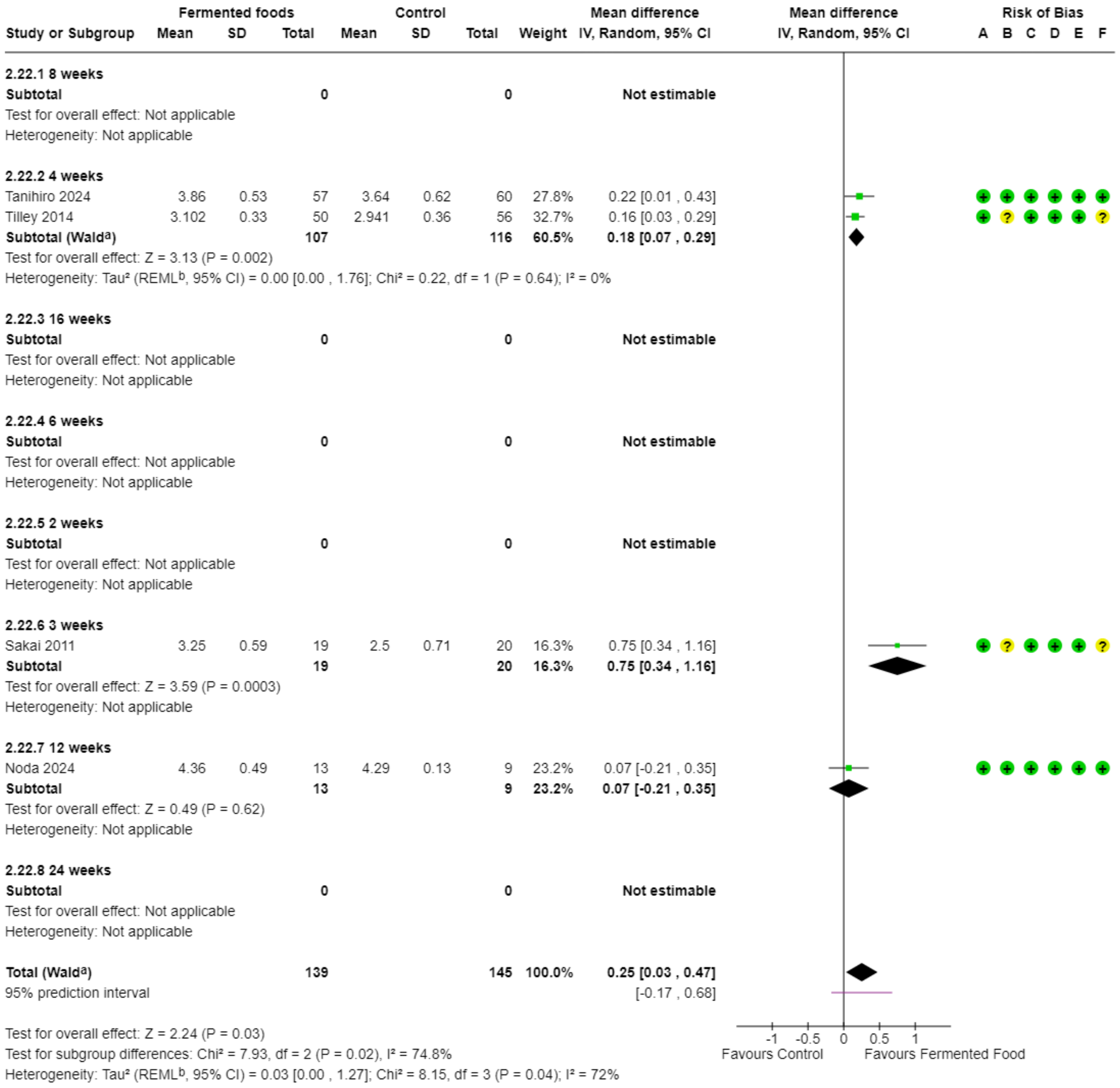

Supplementary Figure S3B. Subgroup analysis based on the intervention duration for stool consistency (BSFS). Forest plot of subgroup analysis based on the intervention duration for stool consistency (BSFS) in randomised controlled trials comparing fermented foods with control in healthy adults (n=284). Values were calculated as mean difference (95% CIs) using a random-effects model. BSFS, Bristol stool form scale; CI, confidence interval; IV, inverse variance; SD, standard deviation; [a]CI calculated by Wald-type method; [b]Tau^2 calculated using Restricted Maximum-Likelihood method; Risk of bias legend: (A) bias arising from the randomisation process, (B) bias due to deviations from intended interventions, (C) bias due to missing outcome data, (D) bias in the measurement of the outcome, (E) bias in the selection of the reported result, (F) overall bias.

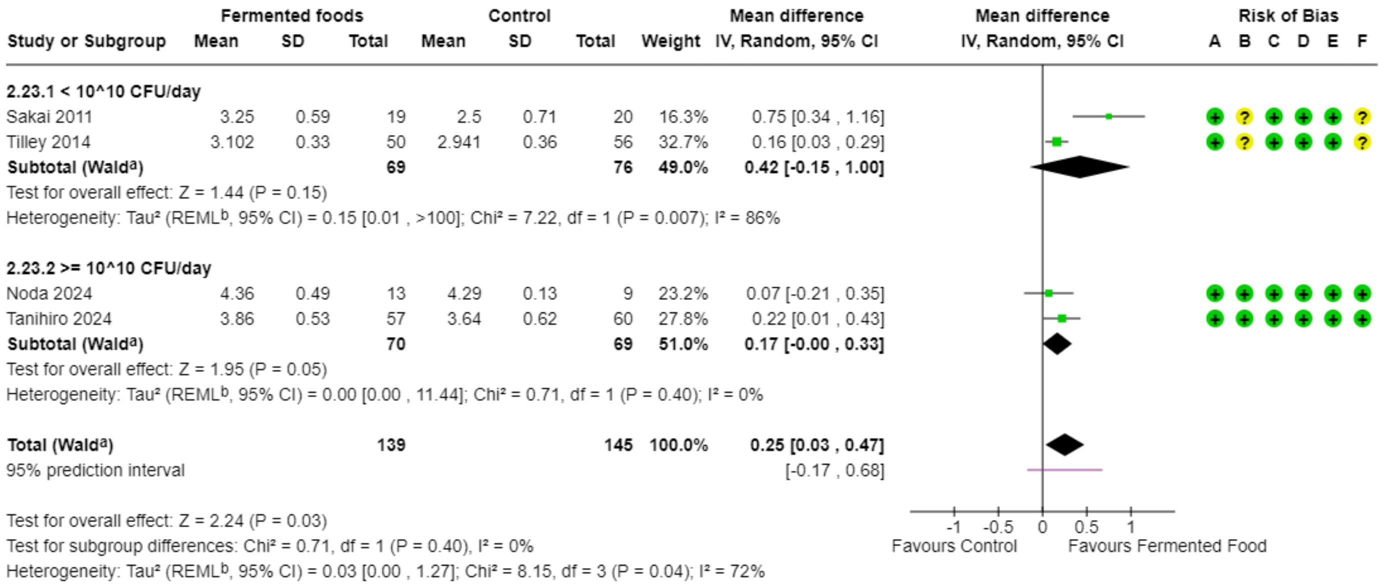

Supplementary Figure S3C. Subgroup analysis based on the microbial dosage for stool consistency (BSFS). Forest plot of subgroup analysis based on the microbial dosage for stool consistency (BSFS) in randomised controlled trials comparing fermented foods with control in healthy adults (n=284). Values were calculated as mean difference (95% CIs) using a random-effects model. BSFS, Bristol stool form scale; CI, confidence interval; IV, inverse variance; SD, standard deviation; [a]CI calculated by Wald-type method; [b]Tau^2 calculated using Restricted Maximum-Likelihood method; Risk of bias legend: (A) bias arising from the randomisation process, (B) bias due to deviations from intended interventions, (C) bias due to missing outcome data, (D) bias in the measurement of the outcome, (E) bias in the selection of the reported result, (F) overall bias.

Study or Subgroup	Fermented foods Mean	Fermented foods SD	Fermented foods Total	Control Mean	Control SD	Control Total	Weight	Mean difference IV, Random, 95% CI	A	B	C	D	E	F
**2.24.1 Bif lactis, St thermo, Lb bulgar**														
**Subtotal**			**0**			**0**		**Not estimable**						
Test for overall effect: Not applicable														
Heterogeneity: Not applicable														
**2.24.2 Bif lactis, Lc cremoris, St thermo, Lb bulgar**														
**Subtotal**			**0**			**0**		**Not estimable**						
Test for overall effect: Not applicable														
Heterogeneity: Not applicable														
**2.24.3 Lactobacillus helveticus CP790**														
Tanihiro 2024	3.86	0.53	57	3.64	0.62	60	27.8%	0.22 [0.01 , 0.43]	+	+	+	+	+	+
**Subtotal**			**57**			**60**	**27.8%**	**0.22 [0.01 , 0.43]**						
Test for overall effect: Z = 2.07 (P = 0.04)														
Heterogeneity: Not applicable														
**2.24.4 Aspergillus oryzae**														
**Subtotal**			**0**			**0**		**Not estimable**						
Test for overall effect: Not applicable														
Heterogeneity: Not applicable														
**2.24.5 Lacticaseibacillus casei strain Shirota**														
Sakai 2011	3.25	0.59	19	2.5	0.71	20	16.3%	0.75 [0.34 , 1.16]	+	?	+	+	+	?
Tilley 2014	3.102	0.33	50	2.941	0.36	56	32.7%	0.16 [0.03 , 0.29]	+	?	+	+	+	?
**Subtotal (Wald[a])**			**69**			**76**	**49.0%**	**0.42 [-0.15 , 1.00]**						
Test for overall effect: Z = 1.44 (P = 0.15)														
Heterogeneity: Tau² (REML[b], 95% CI) = 0.15 [0.01 , >100]; Chi² = 7.22, df = 1 (P = 0.007); I² = 86%														
**2.24.6 Lb bulgaricus OLL1073R-1, St thermophilus**														
**Subtotal**			**0**			**0**		**Not estimable**						
Test for overall effect: Not applicable														
Heterogeneity: Not applicable														
**2.24.7 COMPLEX**														
**Subtotal**			**0**			**0**		**Not estimable**						
Test for overall effect: Not applicable														
Heterogeneity: Not applicable														
**2.24.8 Lactococcus cremoris FC**														
**Subtotal**			**0**			**0**		**Not estimable**						
Test for overall effect: Not applicable														
Heterogeneity: Not applicable														
**2.24.9 Lactococcus lactis BM32-1**														
Noda 2024	4.36	0.49	13	4.29	0.13	9	23.2%	0.07 [-0.21 , 0.35]	+	+	+	+	+	+
**Subtotal**			**13**			**9**	**23.2%**	**0.07 [-0.21 , 0.35]**						
Test for overall effect: Z = 0.49 (P = 0.62)														
Heterogeneity: Not applicable														
**2.24.10 Levilactobacillus brevis subsp NSB2**														
**Subtotal**			**0**			**0**		**Not estimable**						
Test for overall effect: Not applicable														
Heterogeneity: Not applicable														
**2.24.11 Bif lactis, Lc lactis, St thermo, Lb bulgar**														
**Subtotal**			**0**			**0**		**Not estimable**						
Test for overall effect: Not applicable														
Heterogeneity: Not applicable														
**Total (Wald[a])**			**139**			**145**	**100.0%**	**0.25 [0.03 , 0.47]**						
95% prediction interval								[-0.17 , 0.68]						

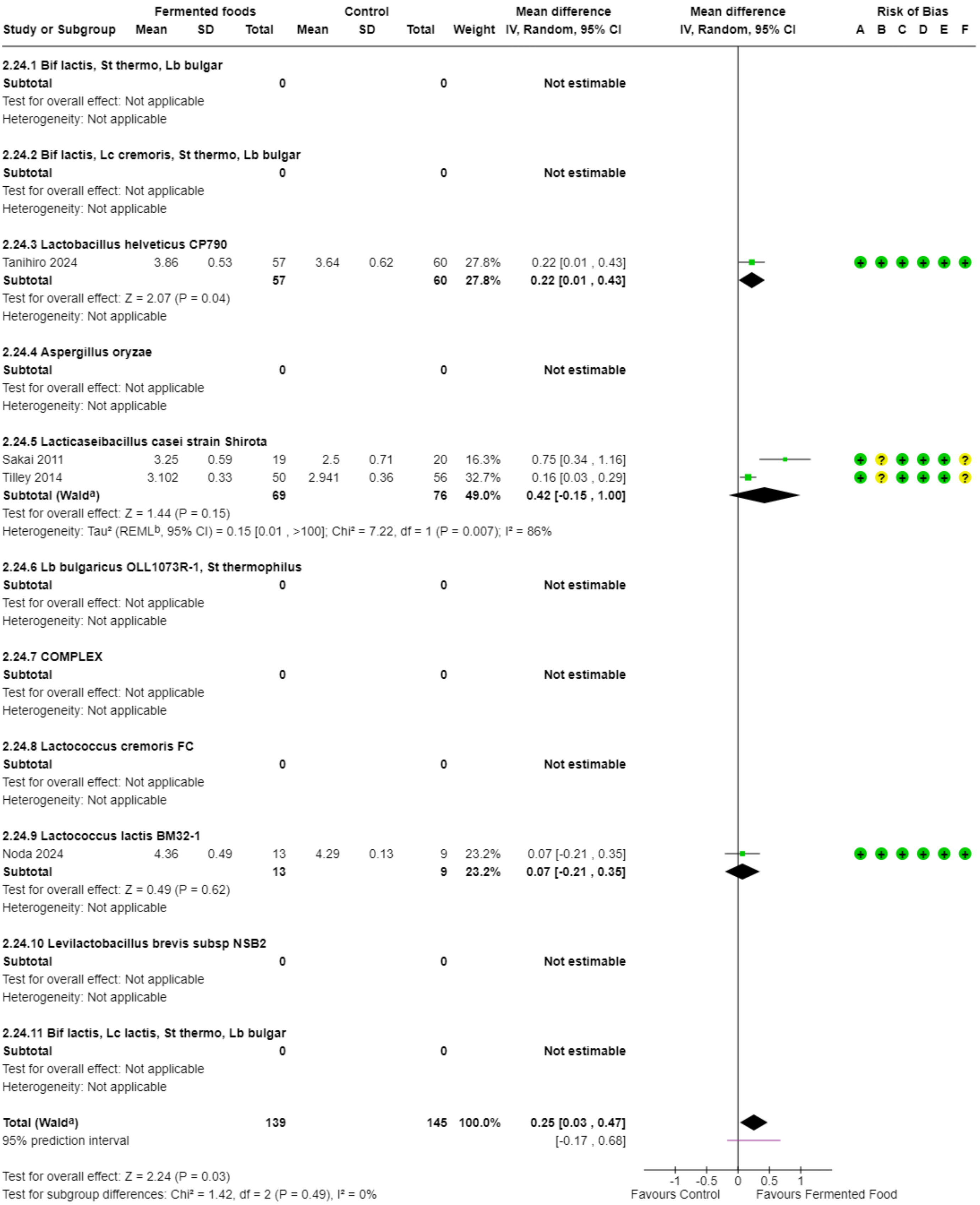

Test for overall effect: Z = 2.24 (P = 0.03)
Test for subgroup differences: Chi² = 1.42, df = 2 (P = 0.49), I² = 0%
Heterogeneity: Tau² (REML[b], 95% CI) = 0.03 [0.00 , 1.27]; Chi² = 8.15, df = 3 (P = 0.04); I² = 72%

Supplementary Figure S3D. Subgroup analysis based on the fermentation microorganisms for stool consistency (BSFS). Forest plot of subgroup analysis based on the fermentation microorganisms for stool consistency (BSFS) in randomised controlled trials comparing fermented foods with control in healthy adults (n=284). Values were calculated as mean difference (95% CIs) using a random-effects model. BSFS, Bristol stool form scale; COMPLEX,

complex fermenting microbial community of several microorganisms consisting primarily of lactic acid bacteria; CI, confidence interval; IV, inverse variance; SD, standard deviation; [a]CI calculated by Wald-type method; [b]Tau^2 calculated using Restricted Maximum-Likelihood method; Risk of bias legend: (A) bias arising from the randomisation process, (B) bias due to deviations from intended interventions, (C) bias due to missing outcome data, (D) bias in the measurement of the outcome, (E) bias in the selection of the reported result, (F) overall bias.

Study or Subgroup	Fermented foods Events	Fermented foods Total	Control Events	Control Total	Weight	Risk ratio M-H, Random, 95% CI
**2.17.1 Sericin-fibroin mixture**						
**Subtotal**		**0**		**0**		**Not estimable**
Total events:	0		0			
Test for overall effect: Not applicable						
Heterogeneity: Not applicable						
**2.17.2 Milk**						
Koebnick 2003	10	35	29	35	52.7%	0.34 [0.20 , 0.59]
Ozaki 2018	2	12	3	12	6.1%	0.67 [0.13 , 3.30]
Sakai 2011	7	19	17	20	41.2%	0.43 [0.23 , 0.80]
**Subtotal (Wald[a])**		**66**		**67**	**100.0%**	**0.39 [0.27 , 0.59]**
Total events:	19		49			
Test for overall effect: Z = 4.61 (P < 0.00001)						
Heterogeneity: Tau² (DL[b], 95% CI) = 0.00 [0.00 , 4.00]; Chi² = 0.74, df = 2 (P = 0.69); I² = 0%						
**2.17.3 Brown rice**						
**Subtotal**		**0**		**0**		**Not estimable**
Total events:	0		0			
Test for overall effect: Not applicable						
Heterogeneity: Not applicable						
**2.17.4 Turnips**						
**Subtotal**		**0**		**0**		**Not estimable**
Total events:	0		0			
Test for overall effect: Not applicable						
Heterogeneity: Not applicable						
**2.17.5 Cabbage/cucumber**						
**Subtotal**		**0**		**0**		**Not estimable**
Total events:	0		0			
Test for overall effect: Not applicable						
Heterogeneity: Not applicable						
**2.17.6 Koji rice**						
**Subtotal**		**0**		**0**		**Not estimable**
Total events:	0		0			
Test for overall effect: Not applicable						
Heterogeneity: Not applicable						
**2.17.7 Soy milk**						
**Subtotal**		**0**		**0**		**Not estimable**
Total events:	0		0			
Test for overall effect: Not applicable						
Heterogeneity: Not applicable						
**Total (Wald[a])**		**66**		**67**	**100.0%**	**0.39 [0.27 , 0.59]**
95% prediction interval						[0.27 , 0.59]
Total events:	19		49			
Test for overall effect: Z = 4.61 (P < 0.00001)						
Test for subgroup differences: Not applicable						
Heterogeneity: Tau² (DL[b], 95% CI) = 0.00 [0.00 , 4.00]; Chi² = 0.74, df = 2 (P = 0.69); I² = 0%						

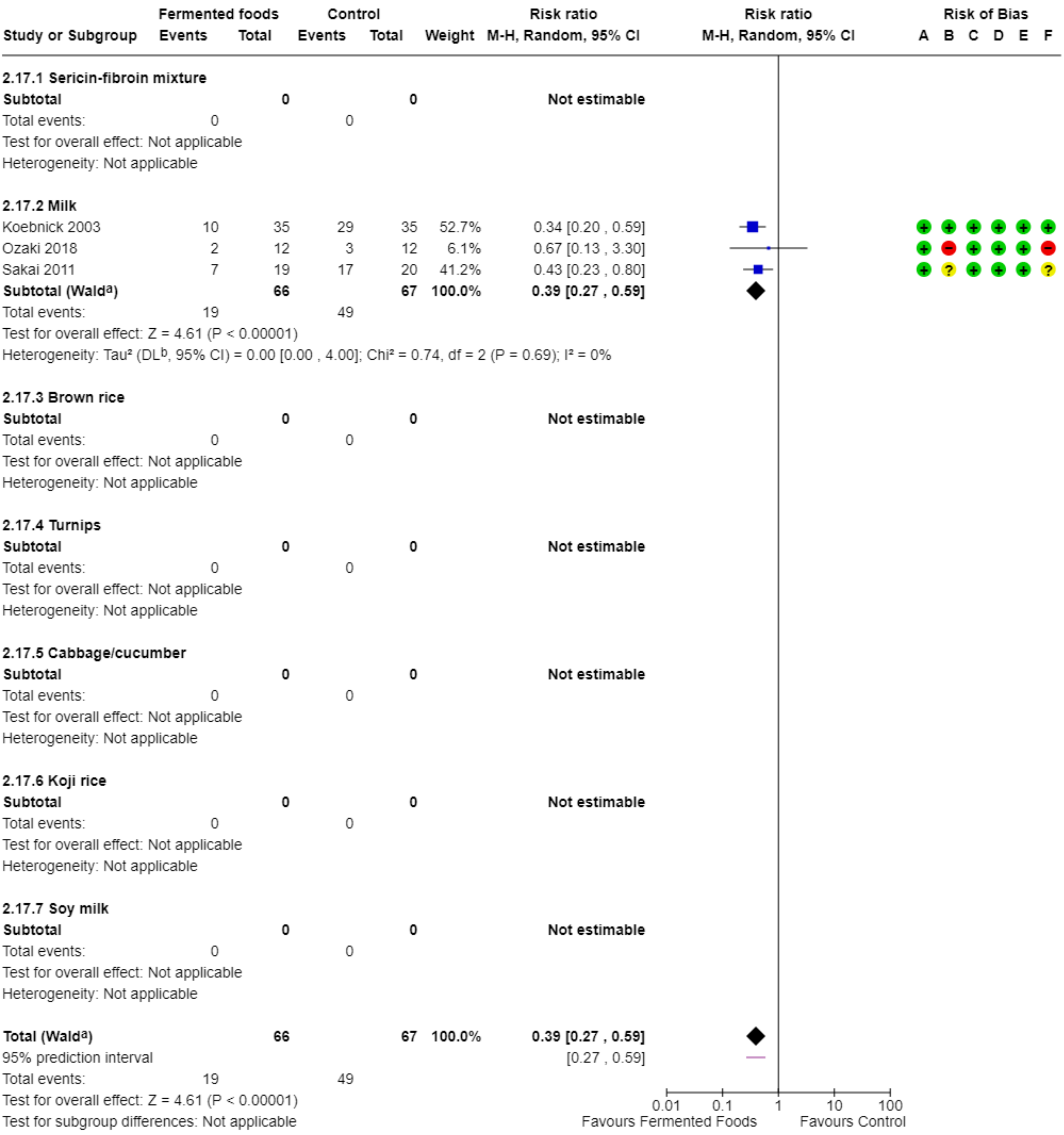

Supplementary Figure S4A. Subgroup analysis based on the fermentation matrix for incidence of hard stools. Forest plot of subgroup analysis based on the fermentation matrix for the incidence of hard stools in randomised controlled trials comparing fermented foods with control in healthy adults (n=133). Values were calculated as risk ratio (95% CIs) using a random-effects model. CI, confidence interval; M-H, Mantel-Haenszel; RR, risk ratio; [a]CI calculated by Wald-type method; [b]Tau^2 calculated using DerSimonian and Laird method; Risk of bias legend: (A) bias arising from the randomisation process, (B) bias due to deviations from intended interventions, (C) bias due to missing outcome data, (D) bias in the measurement of the outcome, (E) bias in the selection of the reported result, (F) overall bias.

Study or Subgroup	Fermented foods Events	Fermented foods Total	Control Events	Control Total	Weight	Risk ratio M-H, Random, 95% CI
**2.18.1 8 weeks**						
**Subtotal**		**0**		**0**		**Not estimable**
Total events:	0		0			
Test for overall effect: Not applicable						
Heterogeneity: Not applicable						
**2.18.2 4 weeks**						
Koebnick 2003	10	35	29	35	52.7%	0.34 [0.20 , 0.59]
Ozaki 2018	2	12	3	12	6.1%	0.67 [0.13 , 3.30]
**Subtotal (Wald[a])**		**47**		**47**	**58.8%**	**0.37 [0.22 , 0.62]**
Total events:	12		32			
Test for overall effect: Z = 3.78 (P = 0.0002)						
Heterogeneity: Tau² (DL[b], 95% CI) = 0.00 [0.00 , >100]; Chi² = 0.59, df = 1 (P = 0.44); I² = 0%						
**2.18.3 16 weeks**						
**Subtotal**		**0**		**0**		**Not estimable**
Total events:	0		0			
Test for overall effect: Not applicable						
Heterogeneity: Not applicable						
**2.18.4 6 weeks**						
**Subtotal**		**0**		**0**		**Not estimable**
Total events:	0		0			
Test for overall effect: Not applicable						
Heterogeneity: Not applicable						
**2.18.5 2 weeks**						
**Subtotal**		**0**		**0**		**Not estimable**
Total events:	0		0			
Test for overall effect: Not applicable						
Heterogeneity: Not applicable						
**2.18.6 3 weeks**						
Sakai 2011	7	19	17	20	41.2%	0.43 [0.23 , 0.80]
**Subtotal**		**19**		**20**	**41.2%**	**0.43 [0.23 , 0.80]**
Total events:	7		17			
Test for overall effect: Z = 2.66 (P = 0.008)						
Heterogeneity: Not applicable						
**2.18.7 12 weeks**						
**Subtotal**		**0**		**0**		**Not estimable**
Total events:	0		0			
Test for overall effect: Not applicable						
Heterogeneity: Not applicable						
**2.18.8 24 weeks**						
**Subtotal**		**0**		**0**		**Not estimable**
Total events:	0		0			
Test for overall effect: Not applicable						
Heterogeneity: Not applicable						
**Total (Wald[a])**		**66**		**67**	**100.0%**	**0.39 [0.27 , 0.59]**
95% prediction interval						[0.27 , 0.59]
Total events:	19		49			
Test for overall effect: Z = 4.61 (P < 0.00001)						
Test for subgroup differences: Chi² = 0.15, df = 1 (P = 0.70), I² = 0%						
Heterogeneity: Tau² (DL[b], 95% CI) = 0.00 [0.00 , 4.00]; Chi² = 0.74, df = 2 (P = 0.69); I² = 0%						

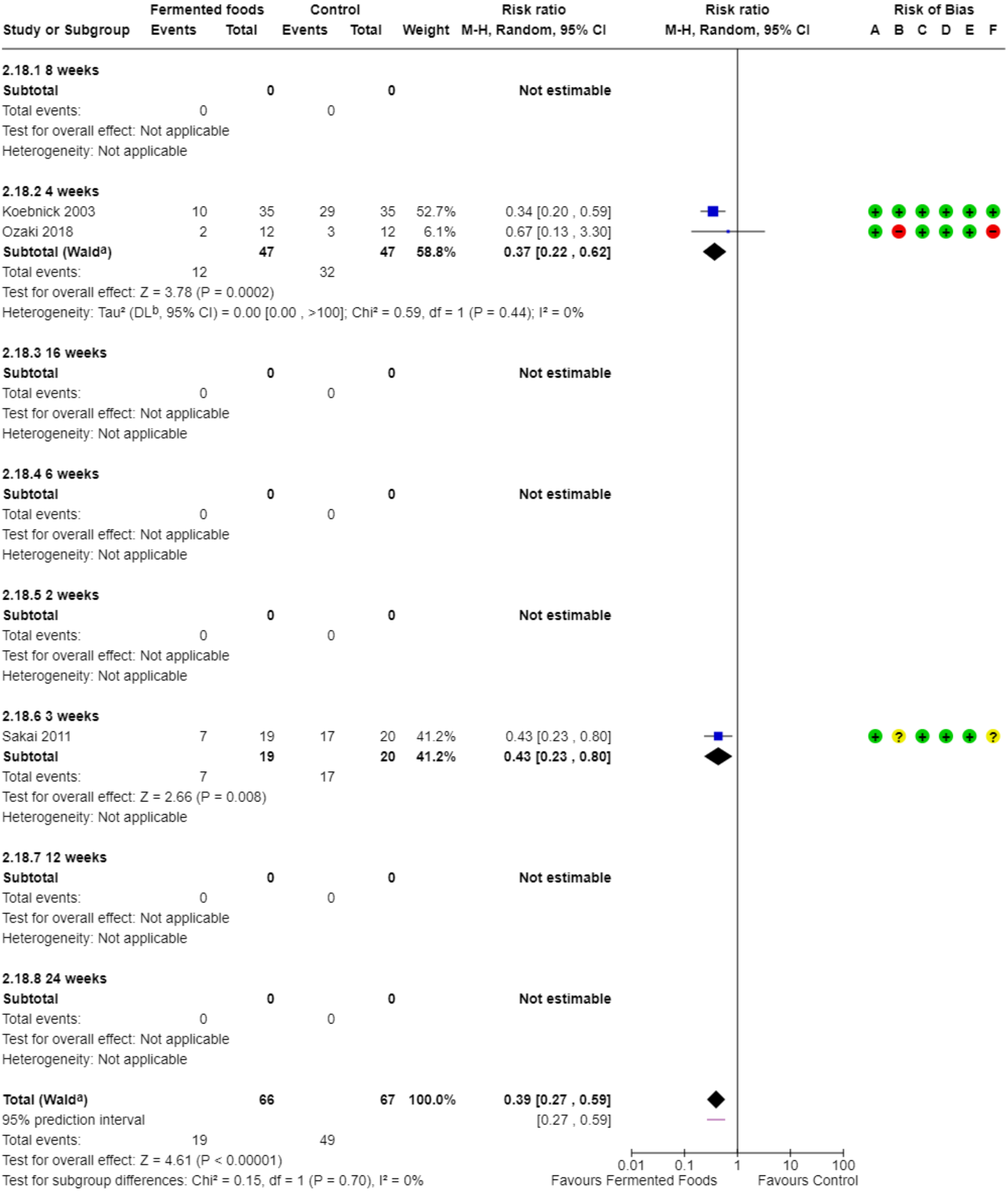

Supplementary Figure S4B. Subgroup analysis based on the intervention duration for incidence of hard stools. Forest plot of subgroup analysis based on the intervention duration for the incidence of hard stools in randomised controlled trials comparing fermented foods with control in healthy adults (n=133). Values were calculated as risk ratio (95% CIs) using a random-effects model. CI, confidence interval; M-H, Mantel-Haenszel; RR, risk ratio; [a]CI calculated by Wald-type method; [b]Tau^2 calculated using DerSimonian and Laird method; Risk of bias legend: (A) bias arising from the

randomisation process, (B) bias due to deviations from intended interventions, (C) bias due to missing outcome data, (D) bias in the measurement of the outcome, (E) bias in the selection of the reported result, (F) overall bias.

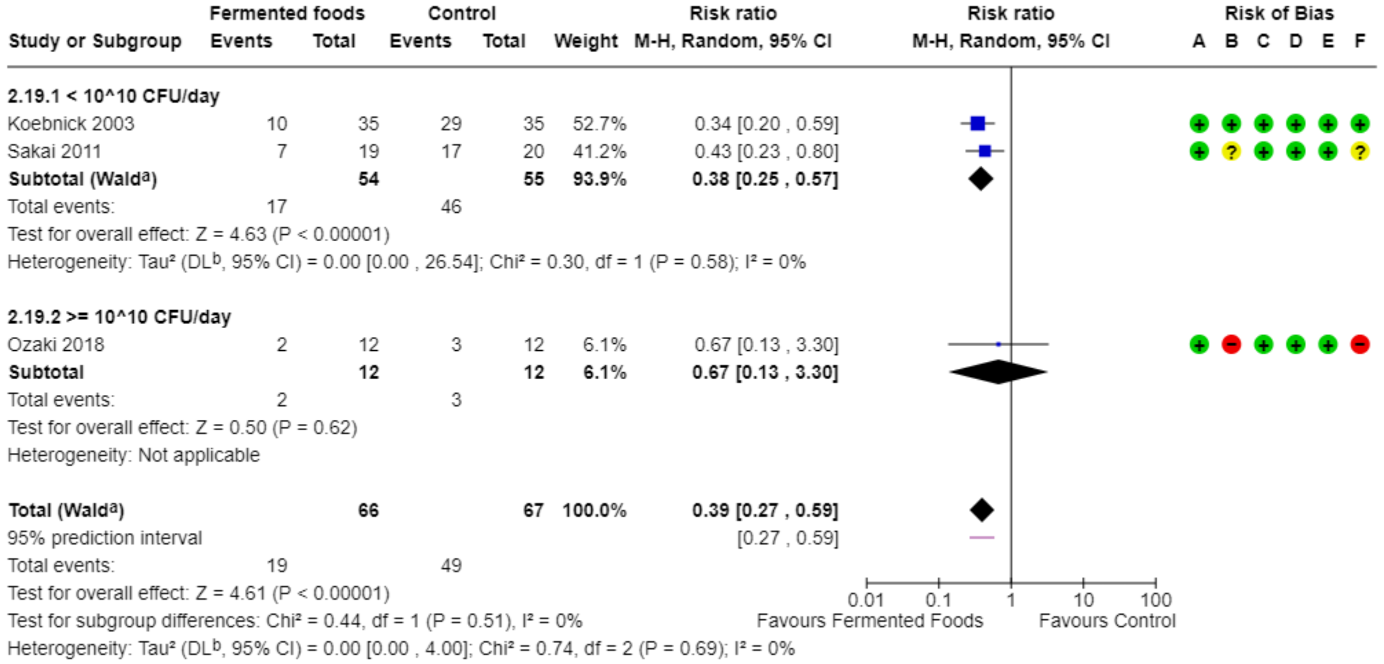

Supplementary Figure S4C. Subgroup analysis based on the microbial dosage for incidence of hard stools. Forest plot of subgroup analysis based on the microbial dosage for the incidence of hard stools in randomised controlled trials comparing fermented foods with control in healthy adults (n=133). Values were calculated as risk ratio (95% CIs) using a random-effects model. CI, confidence interval; M-H, Mantel-Haenszel; RR, risk ratio; [a]CI calculated by Wald-type method; [b]Tau^2 calculated using DerSimonian and Laird method; Risk of bias legend: (A) bias arising from the randomisation process, (B) bias due to deviations from intended interventions, (C) bias due to missing outcome data, (D) bias in the measurement of the outcome, (E) bias in the selection of the reported result, (F) overall bias.

Study or Subgroup	Fermented foods Events	Fermented foods Total	Control Events	Control Total	Weight	Risk ratio M-H, Random, 95% CI	A	B	C	D	E	F
**2.20.1 Bif lactis, St thermo, Lb bulgar**												
**Subtotal**		**0**		**0**		**Not estimable**						
Total events:	0		0									
Test for overall effect: Not applicable												
Heterogeneity: Not applicable												
**2.20.2 Bif lactis, Lc cremoris, St thermo, Lb bulgar**												
**Subtotal**		**0**		**0**		**Not estimable**						
Total events:	0		0									
Test for overall effect: Not applicable												
Heterogeneity: Not applicable												
**2.20.3 Lactobacillus helveticus CP790**												
**Subtotal**		**0**		**0**		**Not estimable**						
Total events:	0		0									
Test for overall effect: Not applicable												
Heterogeneity: Not applicable												
**2.20.4 Aspergillus oryzae**												
**Subtotal**		**0**		**0**		**Not estimable**						
Total events:	0		0									
Test for overall effect: Not applicable												
Heterogeneity: Not applicable												
**2.20.5 Lacticaseibacillus casei strain Shirota**												
Koebnick 2003	10	35	29	35	52.7%	0.34 [0.20 , 0.59]	+	+	+	+	+	+
Sakai 2011	7	19	17	20	41.2%	0.43 [0.23 , 0.80]	+	?	+	+	+	?
**Subtotal (Wald[a])**		**54**		**55**	**93.9%**	**0.38 [0.25 , 0.57]**						
Total events:	17		46									
Test for overall effect: Z = 4.63 (P < 0.00001)												
Heterogeneity: Tau^2 (DL[b], 95% CI) = 0.00 [0.00 , 26.54]; Chi^2 = 0.30, df = 1 (P = 0.58); I^2 = 0%												
**2.20.6 Lb bulgaricus OLL1073R-1, St thermophilus**												
**Subtotal**		**0**		**0**		**Not estimable**						
Total events:	0		0									
Test for overall effect: Not applicable												
Heterogeneity: Not applicable												
**2.20.7 COMPLEX**												
**Subtotal**		**0**		**0**		**Not estimable**						
Total events:	0		0									
Test for overall effect: Not applicable												
Heterogeneity: Not applicable												
**2.20.8 Lactococcus cremoris FC**												
Ozaki 2018	2	12	3	12	6.1%	0.67 [0.13 , 3.30]	+	-	+	+	+	-
**Subtotal**		**12**		**12**	**6.1%**	**0.67 [0.13 , 3.30]**						
Total events:	2		3									
Test for overall effect: Z = 0.50 (P = 0.62)												
Heterogeneity: Not applicable												
**2.20.9 Lactococcus lactis BM32-1**												
**Subtotal**		**0**		**0**		**Not estimable**						
Total events:	0		0									
Test for overall effect: Not applicable												
Heterogeneity: Not applicable												
**2.20.10 Levilactobacillus brevis subsp NSB2**												
**Subtotal**		**0**		**0**		**Not estimable**						
Total events:	0		0									
Test for overall effect: Not applicable												
Heterogeneity: Not applicable												
**2.20.11 Bif lactis, Lc lactis, St thermo, Lb bulgar**												
**Subtotal**		**0**		**0**		**Not estimable**						
Total events:	0		0									
Test for overall effect: Not applicable												
Heterogeneity: Not applicable												
**Total (Wald[a])**		**66**		**67**	**100.0%**	**0.39 [0.27 , 0.59]**						
95% prediction interval						[0.27 , 0.59]						
Total events:	19		49									
Test for overall effect: Z = 4.61 (P < 0.00001)												
Test for subgroup differences: Chi^2 = 0.44, df = 1 (P = 0.51), I^2 = 0%												
Heterogeneity: Tau^2 (DL[b], 95% CI) = 0.00 [0.00 , 4.00]; Chi^2 = 0.74, df = 2 (P = 0.69); I^2 = 0%												

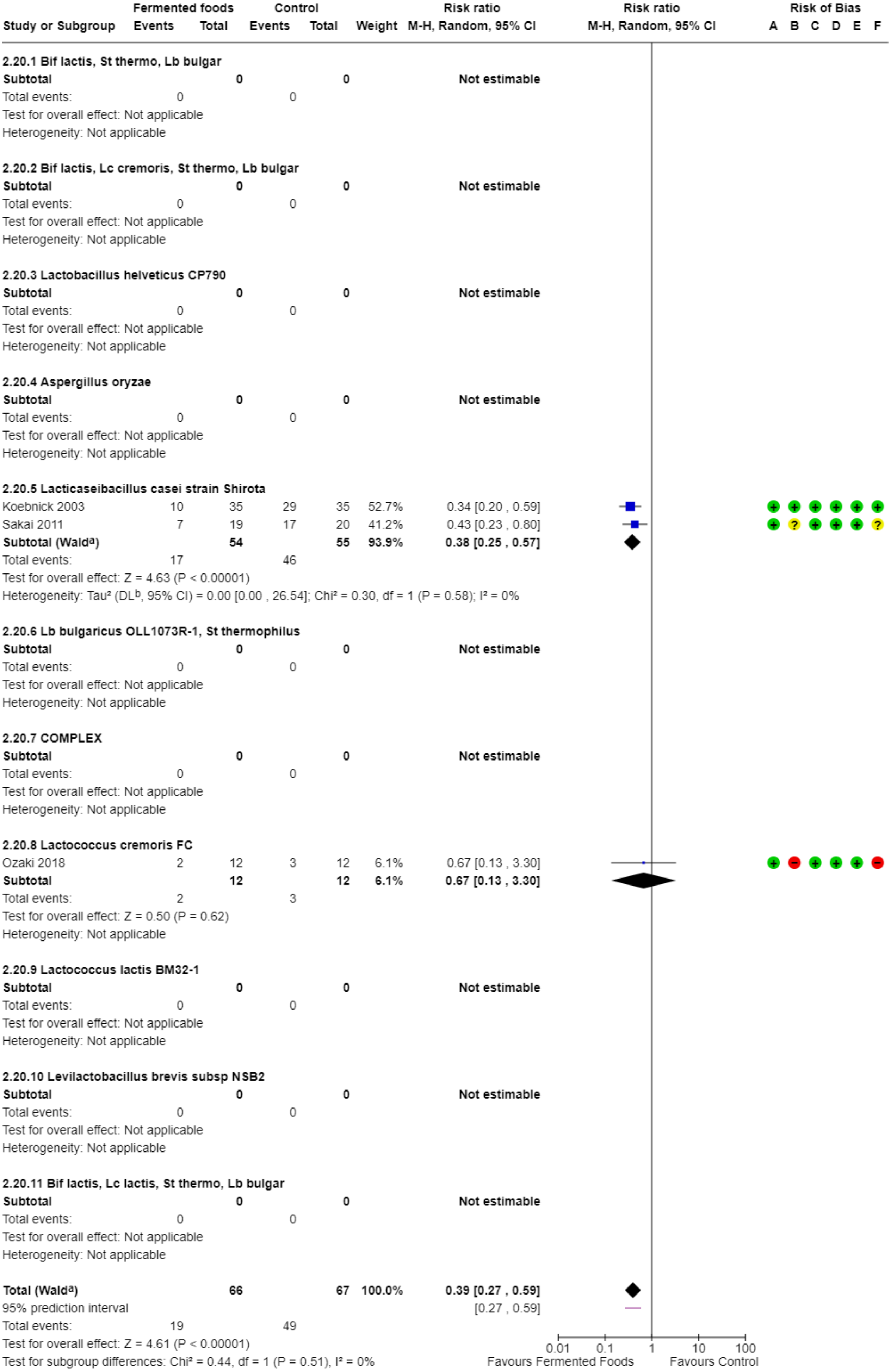

Supplementary Figure S4D. Subgroup analysis based on fermentation microorganisms for the incidence of hard stools. Forest plot of subgroup analysis based on fermentation microorganisms for the incidence of hard stools in randomised controlled trials comparing fermented foods with control in healthy adults (n=133). Values were calculated as risk ratio (95% CIs) using a random-effects model. COMPLEX, complex fermenting microbial community of several microorganisms consisting primarily of lactic acid bacteria; CI, confidence interval; M-H, Mantel-Haenszel; RR, risk ratio; [a]CI calculated by Wald-type method; [b]Tau^2 calculated using DerSimonian and Laird method; Risk of bias legend: (A) bias arising from the randomisation process, (B) bias due to deviations from intended interventions, (C) bias due to missing outcome data, (D) bias in the measurement of the outcome, (E) bias in the selection of the reported result, (F) overall bias.

Study or Subgroup	Fermented foods Mean	SD	Total	Control Mean	SD	Total	Weight	Std. mean difference IV, Random, 95% CI
**2.29.1 Sericin-fibroin mixture**								
Noda 2024	3.41	0.57	25	3.61	0.59	25	29.4%	-0.34 [-0.90 , 0.22]
**Subtotal**			**25**			**25**	**29.4%**	**-0.34 [-0.90 , 0.22]**
Test for overall effect: Z = 1.19 (P = 0.23)								
Heterogeneity: Not applicable								
**2.29.2 Milk**								
Matsumoto 2010	36	15.8	14	40.3	20.2	16	21.6%	-0.23 [-0.95 , 0.49]
Ozaki 2018	5.9	2.7	15	4.5	2.2	16	21.6%	0.56 [-0.16 , 1.28]
**Subtotal (Wald[a])**			**29**			**32**	**43.2%**	**0.16 [-0.61 , 0.93]**
Test for overall effect: Z = 0.42 (P = 0.68)								
Heterogeneity: Tau² (REML[b], 95% CI) = 0.17 [0.00 , >100]; Chi² = 2.28, df = 1 (P = 0.13); I² = 56%								
**2.29.3 Brown rice**								
**Subtotal**			**0**			**0**		**Not estimable**
Test for overall effect: Not applicable								
Heterogeneity: Not applicable								
**2.29.4 Turnips**								
**Subtotal**			**0**			**0**		**Not estimable**
Test for overall effect: Not applicable								
Heterogeneity: Not applicable								
**2.29.5 Cabbage/cucumber**								
**Subtotal**			**0**			**0**		**Not estimable**
Test for overall effect: Not applicable								
Heterogeneity: Not applicable								
**2.29.6 Koji rice**								
Kurahashi 2021	752	360	22	647	386	22	27.4%	0.28 [-0.32 , 0.87]
**Subtotal**			**22**			**22**	**27.4%**	**0.28 [-0.32 , 0.87]**
Test for overall effect: Z = 0.91 (P = 0.36)								
Heterogeneity: Not applicable								
**2.29.7 Soy milk**								
**Subtotal**			**0**			**0**		**Not estimable**
Test for overall effect: Not applicable								
Heterogeneity: Not applicable								
**Total (Wald[a])**			**76**			**79**	**100.0%**	**0.05 [-0.36 , 0.46]**
95% prediction interval								[-0.60 , 0.70]

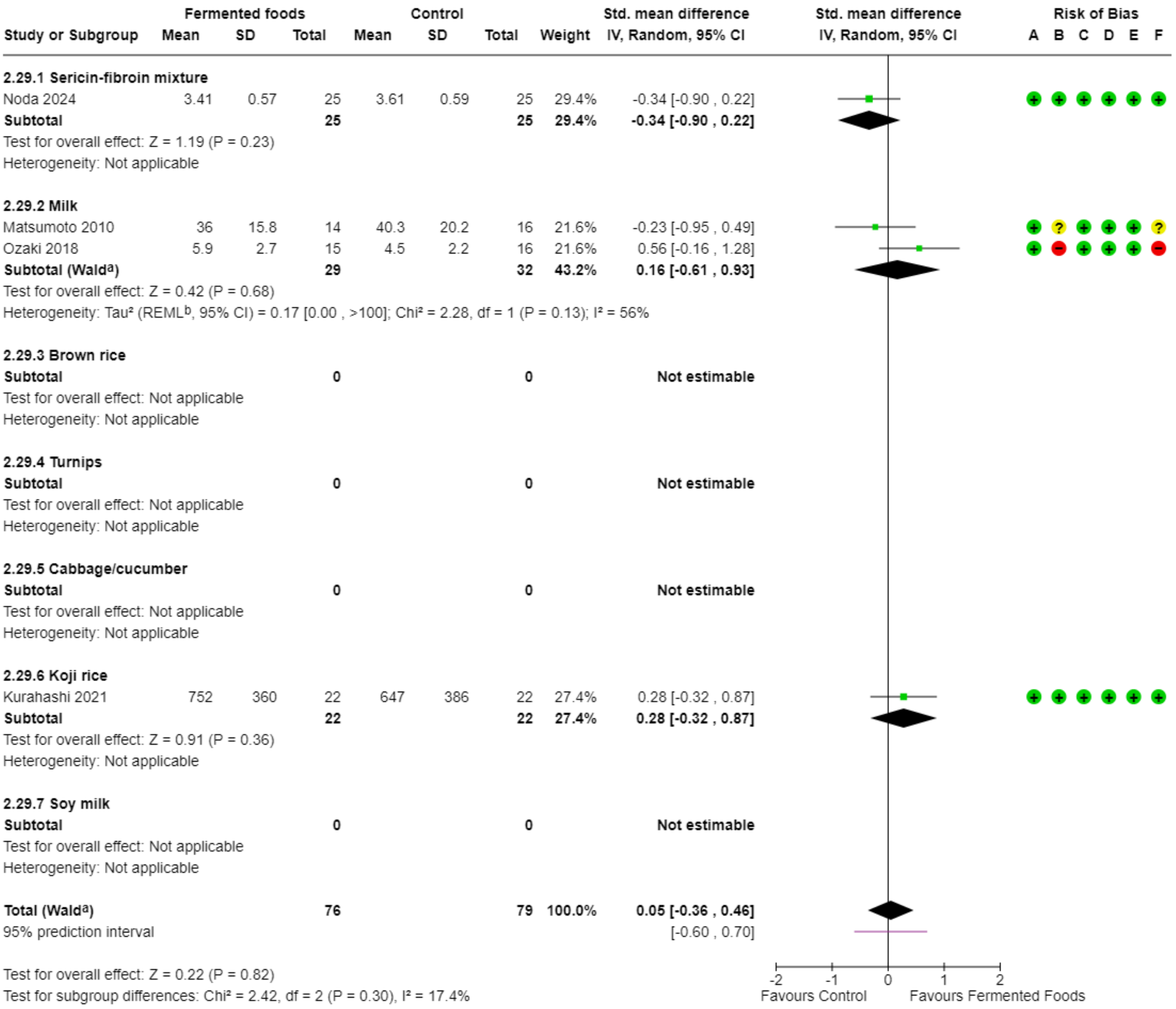

Test for overall effect: Z = 0.22 (P = 0.82)
Test for subgroup differences: Chi² = 2.42, df = 2 (P = 0.30), I² = 17.4%
Heterogeneity: Tau² (REML[b], 95% CI) = 0.07 [0.00 , 2.37]; Chi² = 4.88, df = 3 (P = 0.18); I² = 38%

Supplementary Figure S5A. Subgroup analysis based on the fermentation matrix for stool bulk. Forest plot of subgroup analysis based on the fermentation matrix for stool bulk in randomised controlled trials comparing fermented foods with control in healthy adults (n=155). Values were calculated as standardised mean difference (95% CIs) using a random-effects model. CI, confidence interval; IV, inverse variance; SD, standard deviation; [a]CI calculated by Wald-type method; [b]Tau^2 calculated using Restricted Maximum-Likelihood method; Risk of bias legend: (A) bias arising from the randomisation process, (B) bias due to deviations from intended interventions, (C) bias due to missing outcome data, (D) bias in the measurement of the outcome, (E) bias in the selection of the reported result, (F) overall bias.

Study or Subgroup	Fermented foods Mean	Fermented foods SD	Fermented foods Total	Control Mean	Control SD	Control Total	Weight	Std. mean difference IV, Random, 95% CI
**2.30.1 8 weeks**								
**Subtotal**			**0**			**0**		**Not estimable**
Test for overall effect: Not applicable								
Heterogeneity: Not applicable								
**2.30.2 4 weeks**								
Matsumoto 2010	36	15.8	14	40.3	20.2	16	21.6%	-0.23 [-0.95 , 0.49]
Ozaki 2018	5.9	2.7	15	4.5	2.2	16	21.6%	0.56 [-0.16 , 1.28]
**Subtotal (Wald[a])**			**29**			**32**	**43.2%**	**0.16 [-0.61 , 0.93]**
Test for overall effect: Z = 0.42 (P = 0.68)								
Heterogeneity: Tau² (REML[b], 95% CI) = 0.17 [0.00 , >100]; Chi² = 2.28, df = 1 (P = 0.13); I² = 56%								
**2.30.3 16 weeks**								
**Subtotal**			**0**			**0**		**Not estimable**
Test for overall effect: Not applicable								
Heterogeneity: Not applicable								
**2.30.4 6 weeks**								
**Subtotal**			**0**			**0**		**Not estimable**
Test for overall effect: Not applicable								
Heterogeneity: Not applicable								
**2.30.5 2 weeks**								
**Subtotal**			**0**			**0**		**Not estimable**
Test for overall effect: Not applicable								
Heterogeneity: Not applicable								
**2.30.6 3 weeks**								
Kurahashi 2021	752	360	22	647	386	22	27.4%	0.28 [-0.32 , 0.87]
**Subtotal**			**22**			**22**	**27.4%**	**0.28 [-0.32 , 0.87]**
Test for overall effect: Z = 0.91 (P = 0.36)								
Heterogeneity: Not applicable								
**2.30.7 12 weeks**								
Noda 2024	3.41	0.57	25	3.61	0.59	25	29.4%	-0.34 [-0.90 , 0.22]
**Subtotal**			**25**			**25**	**29.4%**	**-0.34 [-0.90 , 0.22]**
Test for overall effect: Z = 1.19 (P = 0.23)								
Heterogeneity: Not applicable								
**2.30.8 24 weeks**								
**Subtotal**			**0**			**0**		**Not estimable**
Test for overall effect: Not applicable								
Heterogeneity: Not applicable								
**Total (Wald[a])**			**76**			**79**	**100.0%**	**0.05 [-0.36 , 0.46]**
95% prediction interval								[-0.60 , 0.70]

Test for overall effect: Z = 0.22 (P = 0.82)
Test for subgroup differences: Chi² = 2.42, df = 2 (P = 0.30), I² = 17.4%
Heterogeneity: Tau² (REML[b], 95% CI) = 0.07 [0.00 , 2.37]; Chi² = 4.88, df = 3 (P = 0.18); I² = 38%

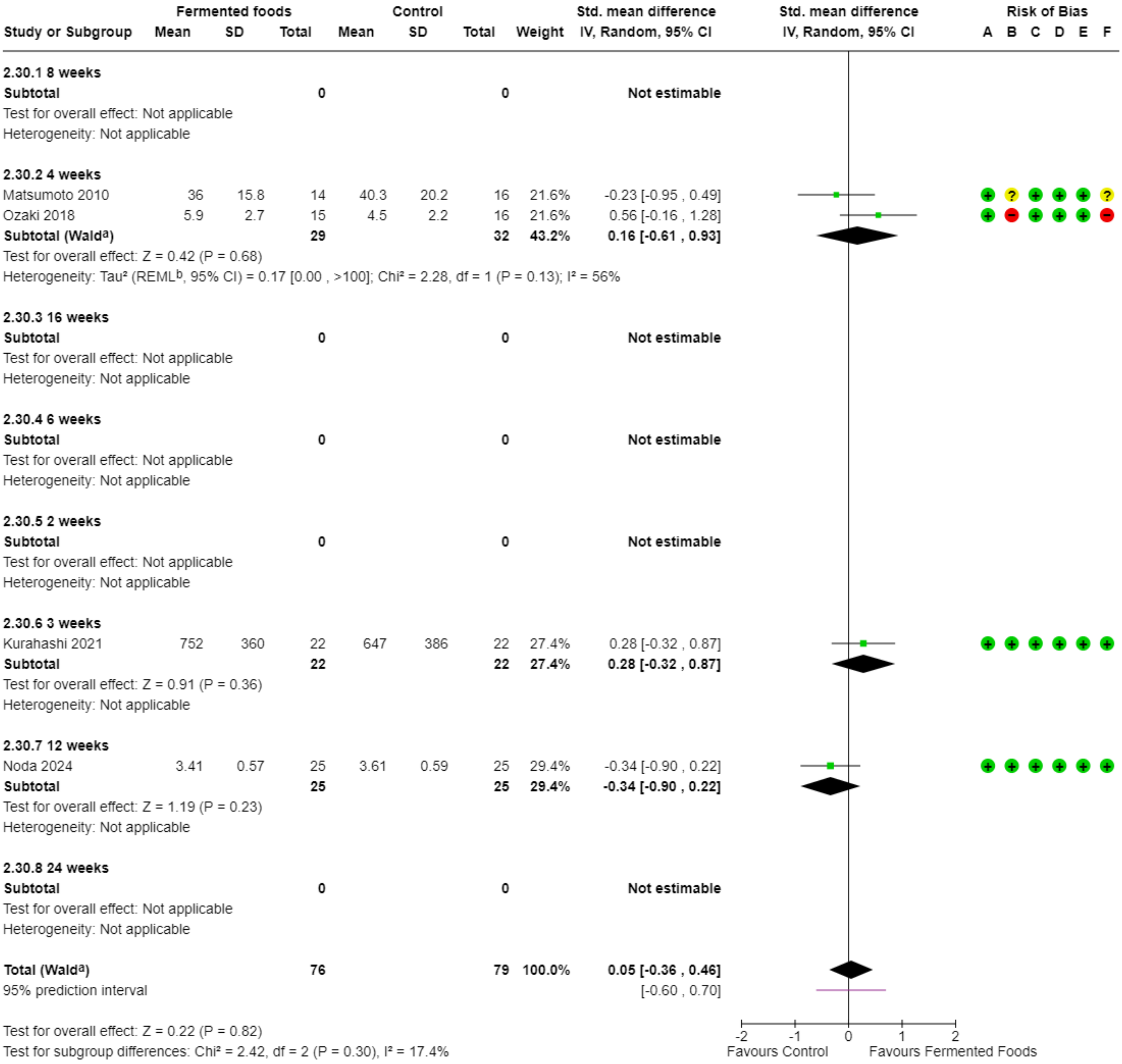

Supplementary Figure S5B. Subgroup analysis based on the intervention duration for stool bulk. Forest plot of subgroup analysis based on the intervention duration for stool bulk in randomised controlled trials comparing fermented foods with control in healthy adults (n=155). Values were calculated as standardised mean difference (95% CIs) using a random-effects model. CI, confidence interval; IV, inverse variance; SD, standard deviation; [a]CI calculated by Wald-type method; [b]Tau^2 calculated using Restricted Maximum-Likelihood method; Risk of bias legend: (A) bias arising from the randomisation process, (B) bias due to deviations from intended interventions, (C) bias due to missing outcome data, (D) bias in the measurement of the outcome, (E) bias in the selection of the reported result, (F) overall bias.

Study or Subgroup	Fermented foods Mean	Fermented foods SD	Fermented foods Total	Control Mean	Control SD	Control Total	Weight	Std. mean difference IV, Random, 95% CI	Risk of Bias A	B	C	D	E	F
**2.31.1 < 10^10 CFU/day**														
**Subtotal**			**0**			**0**		**Not estimable**						
Test for overall effect: Not applicable														
Heterogeneity: Not applicable														
**2.31.2 >= 10^10 CFU/day**														
Matsumoto 2010	36	15.8	14	40.3	20.2	16	30.5%	-0.23 [-0.95 , 0.49]	+	?	+	+	+	?
Noda 2024	3.41	0.57	25	3.61	0.59	25	39.0%	-0.34 [-0.90 , 0.22]	+	+	+	+	+	+
Ozaki 2018	5.9	2.7	15	4.5	2.2	16	30.5%	0.56 [-0.16 , 1.28]	+	-	+	+	+	-
**Subtotal (Wald[a])**			**54**			**57**	**100.0%**	**-0.03 [-0.57 , 0.51]**						
Test for overall effect: Z = 0.12 (P = 0.91)														
Heterogeneity: Tau² (REML[b], 95% CI) = 0.11 [0.00 , 9.28]; Chi² = 3.98, df = 2 (P = 0.14); I² = 50%														
**Total (Wald[a])**			**54**			**57**	**100.0%**	**-0.03 [-0.57 , 0.51]**						
95% prediction interval								[-0.88 , 0.82]						
Test for overall effect: Z = 0.12 (P = 0.91)														
Test for subgroup differences: Not applicable														
Heterogeneity: Tau² (REML[b], 95% CI) = 0.11 [0.00 , 9.28]; Chi² = 3.98, df = 2 (P = 0.14); I² = 50%														

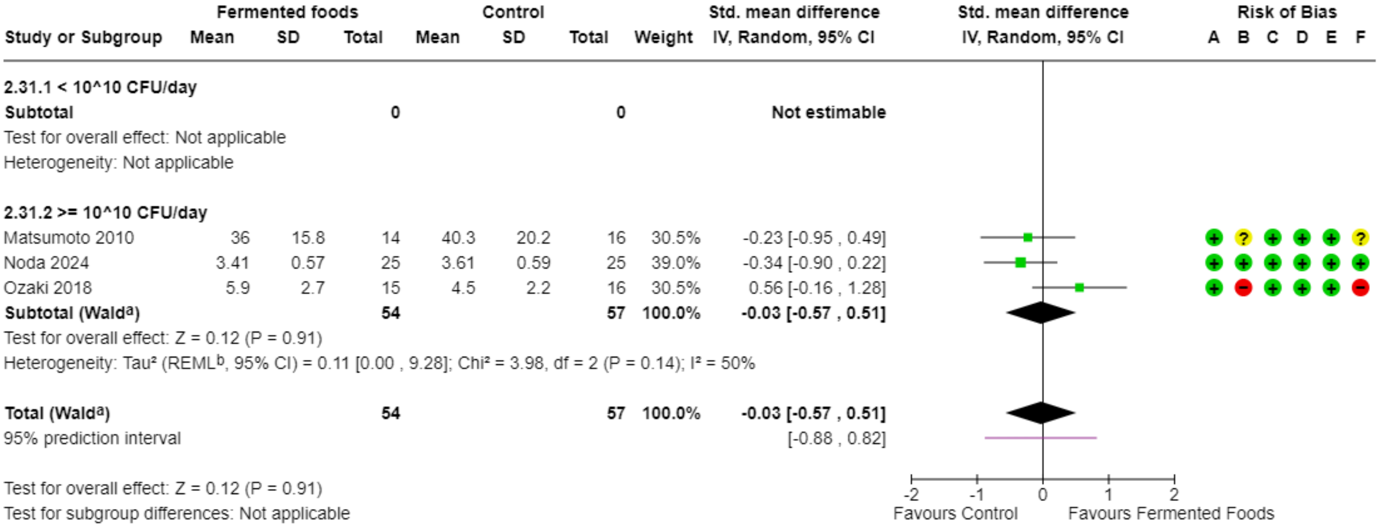

Supplementary Figure S5C. Subgroup analysis based on the microbial dosage for stool bulk. Forest plot of subgroup analysis based on the microbial dosage for stool bulk in randomised controlled trials comparing fermented foods with control in healthy adults (n=155). Values were calculated as standardised mean difference (95% CIs) using a random-effects model. CI, confidence interval; IV, inverse variance; SD, standard deviation; [a]CI calculated by Wald-type method; [b]Tau^2 calculated using Restricted Maximum-Likelihood method; Risk of bias legend: (A) bias arising from the randomisation process, (B) bias due to deviations from intended interventions, (C) bias due to missing outcome data, (D) bias in the measurement of the outcome, (E) bias in the selection of the reported result, (F) overall bias.

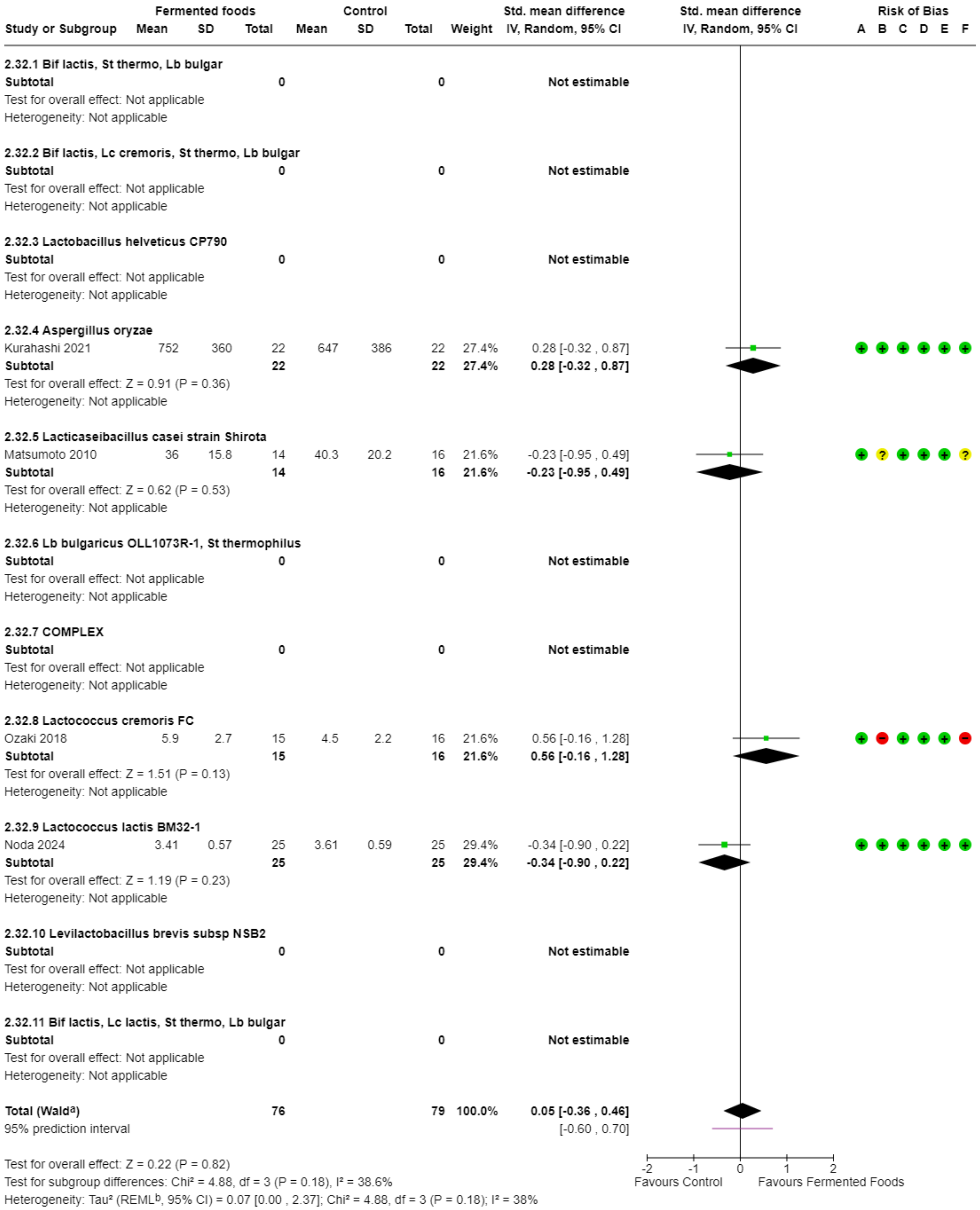

Supplementary Figure S5D. Subgroup analysis based on fermentation microorganisms for stool bulk. Forest plot of subgroup analysis based on the fermentation microorganisms for stool bulk in randomised controlled trials comparing fermented foods with control in healthy adults (n=155). Values were calculated as standardised mean difference (95% CIs) using a random-effects model. COMPLEX, complex fermenting microbial community of several microorganisms

consisting primarily of lactic acid bacteria; CI, confidence interval; IV, inverse variance; SD, standard deviation; [a]CI calculated by Wald-type method; [b]Tau^2 calculated using Restricted Maximum-Likelihood method; Risk of bias legend: (A) bias arising from the randomisation process, (B) bias due to deviations from intended interventions, (C) bias due to missing outcome data, (D) bias in the measurement of the outcome, (E) bias in the selection of the reported result, (F) overall bias.

Study or Subgroup	Fermented foods Mean	Fermented foods SD	Fermented foods Total	Control Mean	Control SD	Control Total	Weight	Mean difference IV, Random, 95% CI	A	B	C	D	E	F
**2.5.1 Sericin-fibroin mixture**														
**Subtotal**			**0**			**0**		**Not estimable**						
Test for overall effect: Not applicable														
Heterogeneity: Not applicable														
**2.5.2 Milk**														
Matsumoto 2010	6.2	0.5	14	6.1	0.6	16	11.8%	0.10 [-0.29 , 0.49]	+	?	+	+	+	?
Nagata (elder cohort) 2015	7.15	0.64	36	7.98	0.6	36	13.6%	-0.83 [-1.12 , -0.54]	+	−	+	+	+	−
Nagata (nursing cohort) 2015	6.88	0.45	10	7.35	0.59	10	10.7%	-0.47 [-0.93 , -0.01]	+	−	+	+	+	−
Ozaki 2018	6.68	0.52	12	6.88	0.39	12	12.2%	-0.20 [-0.57 , 0.17]	+	−	+	+	+	−
Spanhaak 1998	7	0.3	10	6.8	0.4	10	13.2%	0.20 [-0.11 , 0.51]	+	−	+	+	+	−
**Subtotal (Wald[a])**			**82**			**84**	**61.5%**	**-0.24 [-0.63 , 0.14]**						
Test for overall effect: Z = 1.23 (P = 0.22)														
Heterogeneity: Tau² (REML[b], 95% CI) = 0.16 [0.04 , 1.44]; Chi² = 27.76, df = 4 (P < 0.0001); I² = 83%														
**2.5.3 Brown rice**														
Nemoto 2011	7	0.41	18	7.1	0.9	18	10.8%	-0.10 [-0.56 , 0.36]	+	+	+	+	+	+
**Subtotal**			**18**			**18**	**10.8%**	**-0.10 [-0.56 , 0.36]**						
Test for overall effect: Z = 0.43 (P = 0.67)														
Heterogeneity: Not applicable														
**2.5.4 Turnips**														
**Subtotal**			**0**			**0**		**Not estimable**						
Test for overall effect: Not applicable														
Heterogeneity: Not applicable														
**2.5.5 Cabbage/cucumber**														
**Subtotal**			**0**			**0**		**Not estimable**						
Test for overall effect: Not applicable														
Heterogeneity: Not applicable														
**2.5.6 Koji rice**														
Kurahashi 2021	6.97	0.64	22	7	0.37	22	13.2%	-0.03 [-0.34 , 0.28]	+	+	+	+	+	+
**Subtotal**			**22**			**22**	**13.2%**	**-0.03 [-0.34 , 0.28]**						
Test for overall effect: Z = 0.19 (P = 0.85)														
Heterogeneity: Not applicable														
**2.5.7 Soy milk**														
Kaga 2024	6.8	0.64	57	6.64	0.54	55	14.6%	0.16 [-0.06 , 0.38]	+	?	+	+	+	?
**Subtotal**			**57**			**55**	**14.6%**	**0.16 [-0.06 , 0.38]**						
Test for overall effect: Z = 1.43 (P = 0.15)														
Heterogeneity: Not applicable														
**Total (Wald[a])**			**179**			**179**	**100.0%**	**-0.14 [-0.40 , 0.12]**						
95% prediction interval								[-0.83 , 0.55]						
Test for overall effect: Z = 1.07 (P = 0.28)														
Test for subgroup differences: Chi² = 3.71, df = 3 (P = 0.29), I² = 19.2%														
Heterogeneity: Tau² (REML[b], 95% CI) = 0.11 [0.03 , 0.49]; Chi² = 37.87, df = 7 (P < 0.00001); I² = 79%														

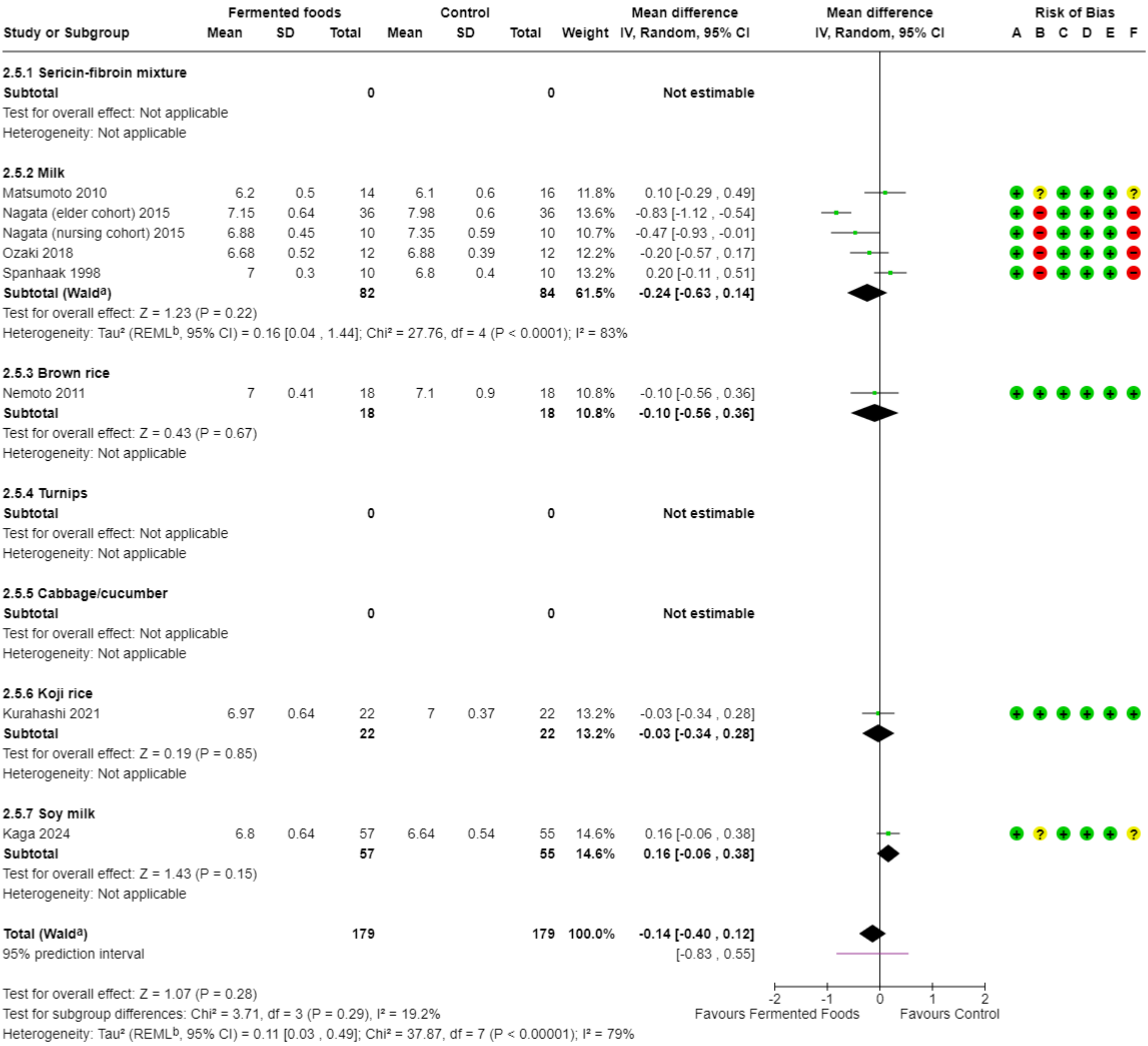

Supplementary Figure S6A. Subgroup analysis based on the fermentation matrix for stool pH. Forest plot of subgroup analysis based on the fermentation matrix for stool pH in randomised controlled trials comparing fermented foods with control in healthy adults (n=358). Values were calculated as mean difference (95% CIs) using a random-effects model. CI, confidence interval; IV, inverse variance; SD, standard deviation; [a]CI calculated by Wald-type method; [b]Tau^2 calculated using Restricted Maximum-Likelihood method; Risk of bias legend: (A) bias arising from the randomisation process, (B) bias due to deviations from intended interventions, (C) bias due to missing outcome data, (D) bias in the measurement of the outcome, (E) bias in the selection of the reported result, (F) overall bias.

Study or Subgroup	Fermented foods Mean	SD	Total	Control Mean	SD	Total	Weight	Mean difference IV, Random, 95% CI
**2.7.1 8 weeks**								
**Subtotal**			**0**			**0**		**Not estimable**
Test for overall effect: Not applicable								
Heterogeneity: Not applicable								
**2.7.2 4 weeks**								
Kaga 2024	6.8	0.64	57	6.64	0.54	55	14.6%	0.16 [-0.06 , 0.38]
Matsumoto 2010	6.2	0.5	14	6.1	0.6	16	11.8%	0.10 [-0.29 , 0.49]
Ozaki 2018	6.68	0.52	12	6.88	0.39	12	12.2%	-0.20 [-0.57 , 0.17]
Spanhaak 1998	7	0.3	10	6.8	0.4	10	13.2%	0.20 [-0.11 , 0.51]
**Subtotal (Wald[a])**			**93**			**93**	**51.8%**	**0.10 [-0.05 , 0.25]**
Test for overall effect: Z = 1.34 (P = 0.18)								
Heterogeneity: Tau² (REML[b], 95% CI) = 0.00 [0.00 , 0.43]; Chi² = 3.24, df = 3 (P = 0.36); I² = 0%								
**2.7.3 16 weeks**								
**Subtotal**			**0**			**0**		**Not estimable**
Test for overall effect: Not applicable								
Heterogeneity: Not applicable								
**2.7.4 6 weeks**								
**Subtotal**			**0**			**0**		**Not estimable**
Test for overall effect: Not applicable								
Heterogeneity: Not applicable								
**2.7.5 2 weeks**								
Nemoto 2011	7	0.41	18	7.1	0.9	18	10.8%	-0.10 [-0.56 , 0.36]
**Subtotal**			**18**			**18**	**10.8%**	**-0.10 [-0.56 , 0.36]**
Test for overall effect: Z = 0.43 (P = 0.67)								
Heterogeneity: Not applicable								
**2.7.6 3 weeks**								
Kurahashi 2021	6.97	0.64	22	7	0.37	22	13.2%	-0.03 [-0.34 , 0.28]
**Subtotal**			**22**			**22**	**13.2%**	**-0.03 [-0.34 , 0.28]**
Test for overall effect: Z = 0.19 (P = 0.85)								
Heterogeneity: Not applicable								
**2.7.7 12 weeks**								
**Subtotal**			**0**			**0**		**Not estimable**
Test for overall effect: Not applicable								
Heterogeneity: Not applicable								
**2.7.8 24 weeks**								
Nagata (elder cohort) 2015	7.15	0.64	36	7.98	0.6	36	13.6%	-0.83 [-1.12 , -0.54]
Nagata (nursing cohort) 2015	6.88	0.45	10	7.35	0.59	10	10.7%	-0.47 [-0.93 , -0.01]
**Subtotal (Wald[a])**			**46**			**46**	**24.3%**	**-0.70 [-1.04 , -0.36]**
Test for overall effect: Z = 4.01 (P < 0.0001)								
Heterogeneity: Tau² (REML[b], 95% CI) = 0.03 [0.00 , 65.94]; Chi² = 1.70, df = 1 (P = 0.19); I² = 41%								
**Total (Wald[a])**			**179**			**179**	**100.0%**	**-0.14 [-0.40 , 0.12]**
95% prediction interval								[-0.83 , 0.55]
Test for overall effect: Z = 1.07 (P = 0.28)								
Test for subgroup differences: Chi² = 17.81, df = 3 (P = 0.0005), I² = 83.2%								
Heterogeneity: Tau² (REML[b], 95% CI) = 0.11 [0.03 , 0.49]; Chi² = 37.87, df = 7 (P < 0.00001); I² = 79%								

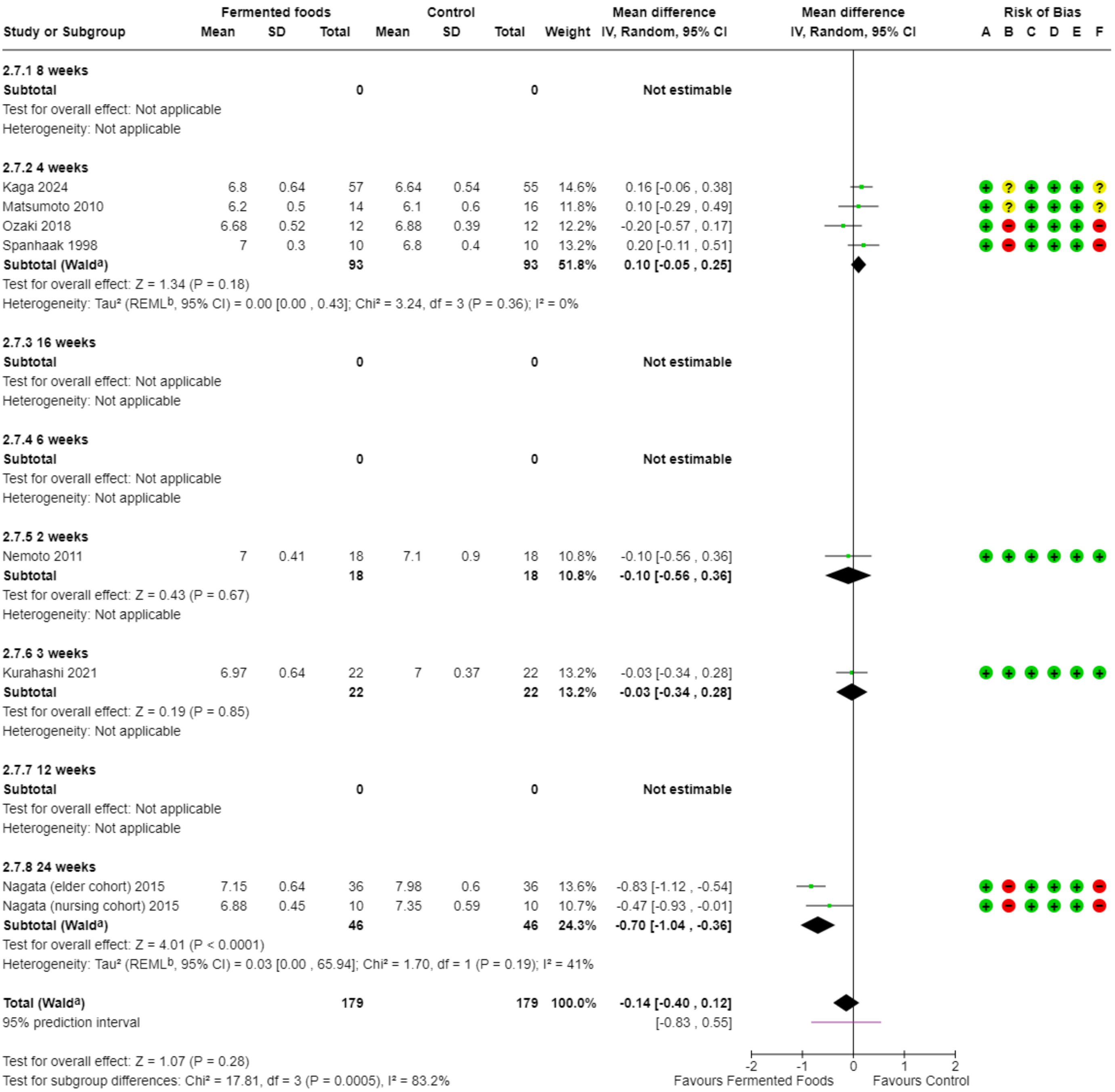

Supplementary Figure S6B. Subgroup analysis based on the intervention duration for stool pH. Forest plot of subgroup analysis based on the intervention duration for stool pH in randomised controlled trials comparing fermented foods with control in healthy adults (n=358). Values were calculated as mean difference (95% CIs) using a random-effects model. CI, confidence interval; IV, inverse variance; SD, standard deviation; [a]CI calculated by Wald-type method; [b]Tau^2 calculated using Restricted Maximum-Likelihood method; Risk of bias legend: (A) bias arising from the randomisation process, (B) bias due to deviations from intended interventions, (C) bias due to missing outcome data, (D) bias in the measurement of the outcome, (E) bias in the selection of the reported result, (F) overall bias.

Study or Subgroup	Fermented foods Mean	SD	Total	Control Mean	SD	Total	Weight	Mean difference IV, Random, 95% CI	Risk of Bias A	B	C	D	E	F
**2.8.1 < 10^10 CFU/day**														
**Subtotal**			**0**			**0**		**Not estimable**						
Test for overall effect: Not applicable														
Heterogeneity: Not applicable														
**2.8.2 >= 10^10 CFU/day**														
Kaga 2024	6.8	0.64	57	6.64	0.54	55	18.5%	0.16 [-0.06 , 0.38]	+	?	+	+	+	?
Matsumoto 2010	6.2	0.5	14	6.1	0.6	16	15.8%	0.10 [-0.29 , 0.49]	+	?	+	+	+	?
Nagata (elder cohort) 2015	7.15	0.64	36	7.98	0.6	36	17.6%	-0.83 [-1.12 , -0.54]	+	-	+	+	+	-
Nagata (nursing cohort) 2015	6.88	0.45	10	7.35	0.59	10	14.7%	-0.47 [-0.93 , -0.01]	+	-	+	+	+	-
Ozaki 2018	6.68	0.52	12	6.88	0.39	12	16.2%	-0.20 [-0.57 , 0.17]	+	-	+	+	+	-
Spanhaak 1998	7	0.3	10	6.8	0.4	10	17.2%	0.20 [-0.11 , 0.51]	+	-	+	+	+	-
**Subtotal (Wald[a])**			**139**			**139**	**100.0%**	**-0.17 [-0.51 , 0.17]**						
Test for overall effect: Z = 0.97 (P = 0.33)														
Heterogeneity: Tau² (REML[b], 95% CI) = 0.15 [0.04 , 0.99]; Chi² = 37.55, df = 5 (P < 0.00001); I² = 85%														
**Total (Wald[a])**			**139**			**139**	**100.0%**	**-0.17 [-0.51 , 0.17]**						
95% prediction interval								[-1.00 , 0.66]						

Test for overall effect: Z = 0.97 (P = 0.33)
Test for subgroup differences: Not applicable
Heterogeneity: Tau² (REML[b], 95% CI) = 0.15 [0.04 , 0.99]; Chi² = 37.55, df = 5 (P < 0.00001); I² = 85%

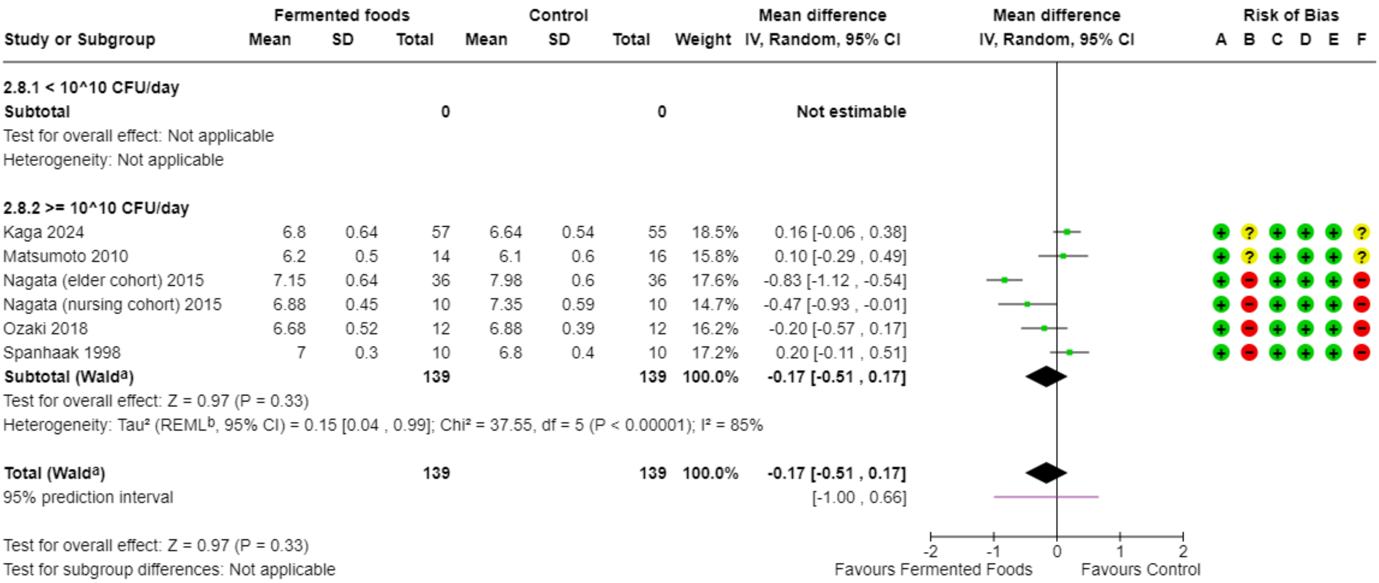

Supplementary Figure S6C. Subgroup analysis based on the microbial dosage for stool pH. Forest plot of subgroup analysis based on the microbial dosage for stool pH in randomised controlled trials comparing fermented foods with control in healthy adults (n=358). Values were calculated as mean difference (95% CIs) using a random-effects model. CI, confidence interval; IV, inverse variance; SD, standard deviation; [a]CI calculated by Wald-type method; [b]Tau^2 calculated using Restricted Maximum-Likelihood method; Risk of bias legend: (A) bias arising from the randomisation process, (B) bias due to deviations from intended interventions, (C) bias due to missing outcome data, (D) bias in the measurement of the outcome, (E) bias in the selection of the reported result, (F) overall bias.

Study or Subgroup	Fermented foods Mean	Fermented foods SD	Fermented foods Total	Control Mean	Control SD	Control Total	Weight	Mean difference IV, Random, 95% CI	Risk of Bias A	B	C	D	E	F
**2.6.1 Bif lactis, St thermo, Lb bulgar**														
**Subtotal**			**0**			**0**		**Not estimable**						
Test for overall effect: Not applicable														
Heterogeneity: Not applicable														
**2.6.2 Bif lactis, Lc cremoris, St thermo, Lb bulgar**														
**Subtotal**			**0**			**0**		**Not estimable**						
Test for overall effect: Not applicable														
Heterogeneity: Not applicable														
**2.6.3 Lactobacillus helveticus CP790**														
**Subtotal**			**0**			**0**		**Not estimable**						
Test for overall effect: Not applicable														
Heterogeneity: Not applicable														
**2.6.4 Aspergillus oryzae**														
Kurahashi 2021	6.97	0.64	22	7	0.37	22	13.2%	-0.03 [-0.34 , 0.28]	+	+	+	+	+	+
Nemoto 2011	7	0.41	18	7.1	0.9	18	10.8%	-0.10 [-0.56 , 0.36]	+	+	+	+	+	+
**Subtotal (Wald[a])**			**40**			**40**	**24.0%**	**-0.05 [-0.31 , 0.20]**						
Test for overall effect: Z = 0.40 (P = 0.69)														
Heterogeneity: Tau^2 (REML[b], 95% CI) = 0.00 [0.00 , 2.46]; Chi^2 = 0.06, df = 1 (P = 0.80); I^2 = 0%														
**2.6.5 Lacticaseibacillus casei strain Shirota**														
Kaga 2024	6.8	0.64	57	6.64	0.54	55	14.6%	0.16 [-0.06 , 0.38]	+	?	+	+	+	?
Matsumoto 2010	6.2	0.5	14	6.1	0.6	16	11.8%	0.10 [-0.29 , 0.49]	+	?	+	+	+	?
Nagata (elder cohort) 2015	7.15	0.64	36	7.98	0.6	36	13.6%	-0.83 [-1.12 , -0.54]	+	-	+	+	+	-
Nagata (nursing cohort) 2015	6.88	0.45	10	7.35	0.59	10	10.7%	-0.47 [-0.93 , -0.01]	+	-	+	+	+	-
Spanhaak 1998	7	0.3	10	6.8	0.4	10	13.2%	0.20 [-0.11 , 0.51]	+	-	+	+	+	-
**Subtotal (Wald[a])**			**127**			**127**	**63.8%**	**-0.16 [-0.57 , 0.25]**						
Test for overall effect: Z = 0.77 (P = 0.44)														
Heterogeneity: Tau^2 (REML[b], 95% CI) = 0.19 [0.05 , 1.72]; Chi^2 = 37.37, df = 4 (P < 0.00001); I^2 = 88%														
**2.6.6 Lb bulgaricus OLL1073R-1, St thermophilus**														
**Subtotal**			**0**			**0**		**Not estimable**						
Test for overall effect: Not applicable														
Heterogeneity: Not applicable														
**2.6.7 COMPLEX**														
**Subtotal**			**0**			**0**		**Not estimable**						
Test for overall effect: Not applicable														
Heterogeneity: Not applicable														
**2.6.8 Lactococcus cremoris FC**														
Ozaki 2018	6.68	0.52	12	6.88	0.39	12	12.2%	-0.20 [-0.57 , 0.17]	+	-	+	+	+	-
**Subtotal**			**12**			**12**	**12.2%**	**-0.20 [-0.57 , 0.17]**						
Test for overall effect: Z = 1.07 (P = 0.29)														
Heterogeneity: Not applicable														
**2.6.9 Lactococcus lactis BM32-1**														
**Subtotal**			**0**			**0**		**Not estimable**						
Test for overall effect: Not applicable														
Heterogeneity: Not applicable														
**2.6.10 Levilactobacillus brevis subsp NSB2**														
**Subtotal**			**0**			**0**		**Not estimable**						
Test for overall effect: Not applicable														
Heterogeneity: Not applicable														
**2.6.11 Bif lactis, Lc lactis, St thermo, Lb bulgar**														
**Subtotal**			**0**			**0**		**Not estimable**						
Test for overall effect: Not applicable														
Heterogeneity: Not applicable														
**Total (Wald[a])**			**179**			**179**	**100.0%**	**-0.14 [-0.40 , 0.12]**						
95% prediction interval								[-0.83 , 0.55]						
Test for overall effect: Z = 1.07 (P = 0.28)														
Test for subgroup differences: Chi^2 = 0.49, df = 2 (P = 0.78), I^2 = 0%														
Heterogeneity: Tau^2 (REML[b], 95% CI) = 0.11 [0.03 , 0.49]; Chi^2 = 37.87, df = 7 (P < 0.00001); I^2 = 79%														

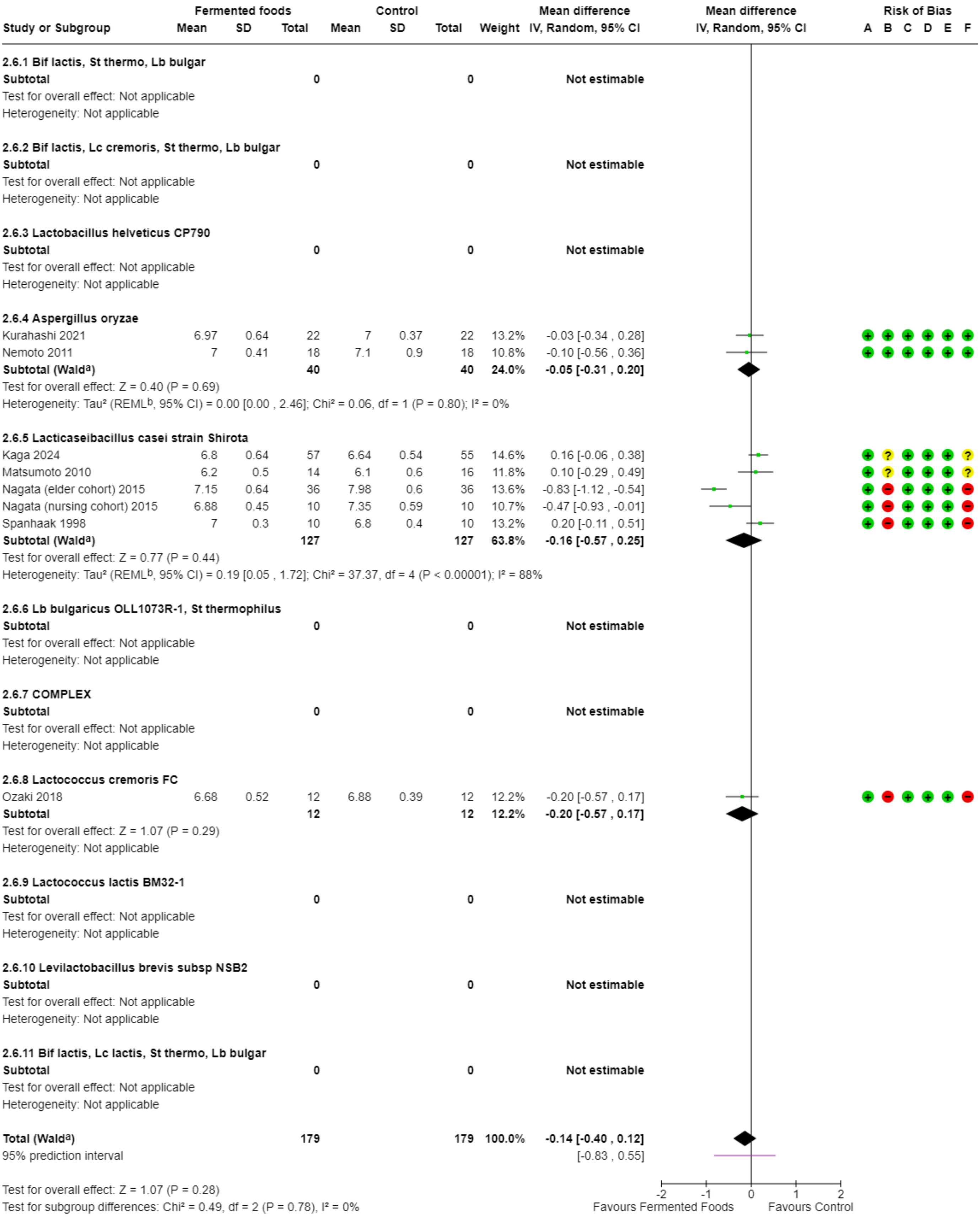

Supplementary Figure S6D. Subgroup analysis based on the fermentation microorganisms for stool pH. Forest plot of subgroup analysis based on the fermentation microorganisms for stool pH in randomised controlled trials comparing fermented foods with control in healthy adults (n=358). Values were calculated as mean difference (95% CIs) using a

random-effects model. COMPLEX, complex fermenting microbial community of several microorganisms consisting primarily of lactic acid bacteria; CI, confidence interval; IV, inverse variance; SD, standard deviation; [a]CI calculated by Wald-type method; [b]Tau^2 calculated using Restricted Maximum-Likelihood method; Risk of bias legend: (A) bias arising from the randomisation process, (B) bias due to deviations from intended interventions, (C) bias due to missing outcome data, (D) bias in the measurement of the outcome, (E) bias in the selection of the reported result, (F) overall bias.

Study or Subgroup	Fermented foods Mean	Fermented foods SD	Fermented foods Total	Control Mean	Control SD	Control Total	Weight	Mean difference IV, Random, 95% CI	A	B	C	D	E	F
**2.9.1 Sericin-fibroin mixture**														
**Subtotal**			**0**			**0**		**Not estimable**						
Test for overall effect: Not applicable														
Heterogeneity: Not applicable														
**2.9.2 Milk**														
Matsumoto 2010	76.9	5.1	14	80.1	5.1	16	17.6%	-3.20 [-6.86 , 0.46]	+	?	+	+	+	?
Ozaki 2018	77	7.8	12	72.1	6.6	12	8.3%	4.90 [-0.88 , 10.68]	+	-	+	+	+	-
Sakai 2011	71.7	6	19	71	5.4	20	18.1%	0.70 [-2.89 , 4.29]	+	?	+	+	+	?
Spanhaak 1998	75	3	10	75	2	10	32.9%	0.00 [-2.23 , 2.23]	+	-	+	+	+	-
**Subtotal (Wald[a])**			**55**			**58**	**76.8%**	**0.03 [-2.32 , 2.38]**						
Test for overall effect: Z = 0.03 (P = 0.98)														
Heterogeneity: Tau² (REML[b], 95% CI) = 2.45 [0.00 , >100]; Chi² = 5.83, df = 3 (P = 0.12); I² = 43%														
**2.9.3 Brown rice**														
**Subtotal**			**0**			**0**		**Not estimable**						
Test for overall effect: Not applicable														
Heterogeneity: Not applicable														
**2.9.4 Turnips**														
**Subtotal**			**0**			**0**		**Not estimable**						
Test for overall effect: Not applicable														
Heterogeneity: Not applicable														
**2.9.5 Cabbage/cucumber**														
**Subtotal**			**0**			**0**		**Not estimable**						
Test for overall effect: Not applicable														
Heterogeneity: Not applicable														
**2.9.6 Koji rice**														
**Subtotal**			**0**			**0**		**Not estimable**						
Test for overall effect: Not applicable														
Heterogeneity: Not applicable														
**2.9.7 Soy milk**														
Kaga 2024	75.2	7.9	57	73.6	8.3	55	23.2%	1.60 [-1.40 , 4.60]	+	?	+	+	+	?
**Subtotal**			**57**			**55**	**23.2%**	**1.60 [-1.40 , 4.60]**						
Test for overall effect: Z = 1.04 (P = 0.30)														
Heterogeneity: Not applicable														
**Total (Wald[a])**			**112**			**113**	**100.0%**	**0.34 [-1.44 , 2.12]**						
95% prediction interval								[-2.45 , 3.14]						
Test for overall effect: Z = 0.38 (P = 0.71)														
Test for subgroup differences: Chi² = 0.65, df = 1 (P = 0.42), I² = 0%														
Heterogeneity: Tau² (REML[b], 95% CI) = 1.21 [0.00 , 64.46]; Chi² = 6.79, df = 4 (P = 0.15); I² = 30%														

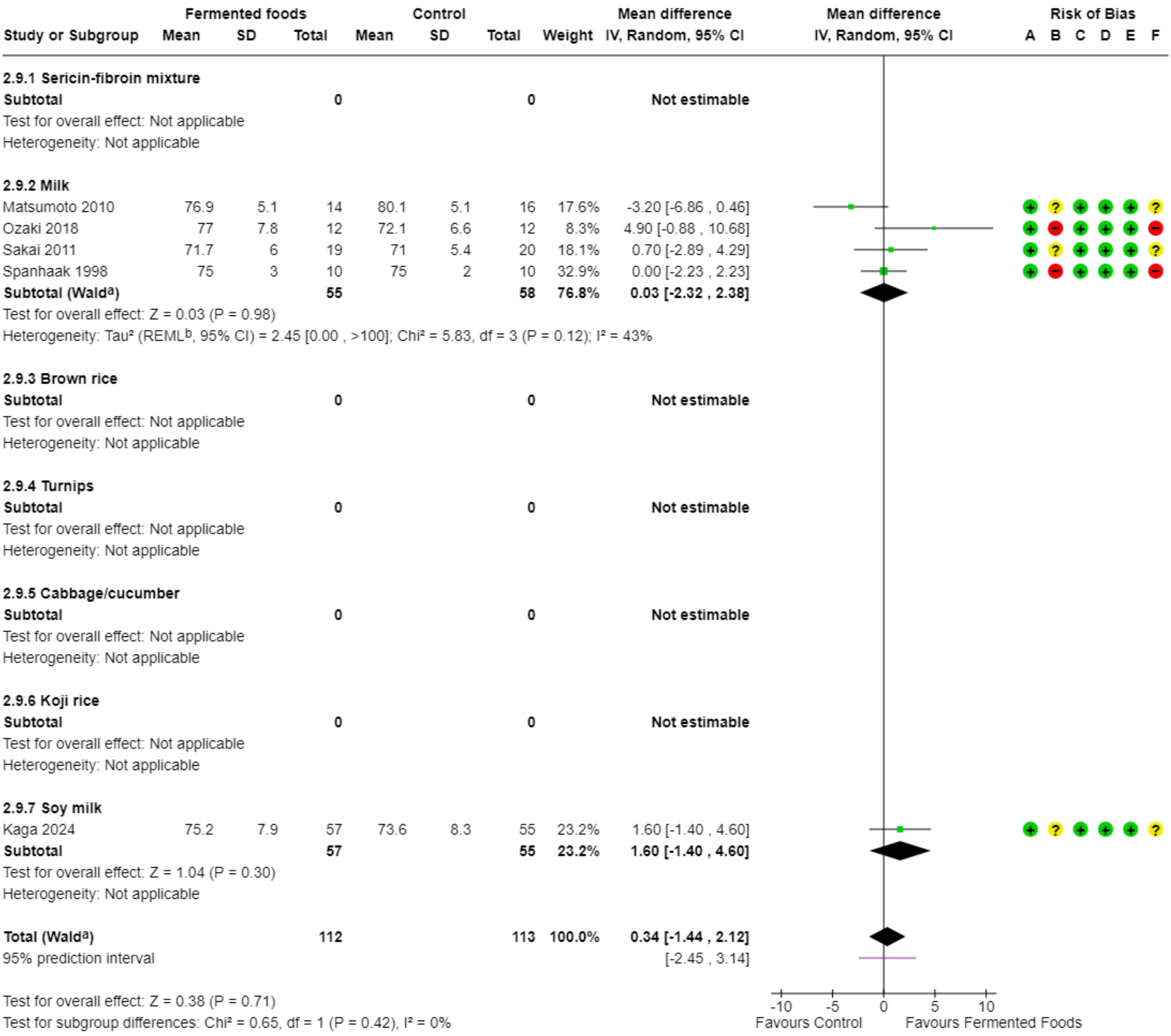

Supplementary Figure S7A. Subgroup analysis based on the fermentation matrix for stool water content. Forest plot of subgroup analysis based on the fermentation matrix for stool water content in randomised controlled trials comparing fermented foods with control in healthy adults (n=225). Values were calculated as mean difference (95% CIs) using a random-effects model. CI, confidence interval; IV, inverse variance; SD, standard deviation; [a]CI calculated by Wald-type method; [b]Tau^2 calculated using Restricted Maximum-Likelihood method; Risk of bias legend: (A) bias arising from the randomisation process, (B) bias due to deviations from intended interventions, (C) bias due to missing outcome data, (D) bias in the measurement of the outcome, (E) bias in the selection of the reported result, (F) overall bias.

Study or Subgroup	Fermented foods Mean	Fermented foods SD	Fermented foods Total	Control Mean	Control SD	Control Total	Weight	Mean difference IV, Random, 95% CI
**2.10.1 8 weeks**								
**Subtotal**			**0**			**0**		**Not estimable**
Test for overall effect: Not applicable								
Heterogeneity: Not applicable								
**2.10.2 4 weeks**								
Kaga 2024	75.2	7.9	57	73.6	8.3	55	23.2%	1.60 [-1.40 , 4.60]
Matsumoto 2010	76.9	5.1	14	80.1	5.1	16	17.6%	-3.20 [-6.86 , 0.46]
Ozaki 2018	77	7.8	12	72.1	6.6	12	8.3%	4.90 [-0.88 , 10.68]
Spanhaak 1998	75	3	10	75	2	10	32.9%	0.00 [-2.23 , 2.23]
**Subtotal (Wald[a])**			**93**			**93**	**81.9%**	**0.37 [-2.20 , 2.94]**
Test for overall effect: Z = 0.28 (P = 0.78)								
Heterogeneity: Tau² (REML[b], 95% CI) = 3.73 [0.00 , >100]; Chi² = 6.72, df = 3 (P = 0.08); I² = 57%								
**2.10.3 16 weeks**								
**Subtotal**			**0**			**0**		**Not estimable**
Test for overall effect: Not applicable								
Heterogeneity: Not applicable								
**2.10.4 6 weeks**								
**Subtotal**			**0**			**0**		**Not estimable**
Test for overall effect: Not applicable								
Heterogeneity: Not applicable								
**2.10.5 2 weeks**								
**Subtotal**			**0**			**0**		**Not estimable**
Test for overall effect: Not applicable								
Heterogeneity: Not applicable								
**2.10.6 3 weeks**								
Sakai 2011	71.7	6	19	71	5.4	20	18.1%	0.70 [-2.89 , 4.29]
**Subtotal**			**19**			**20**	**18.1%**	**0.70 [-2.89 , 4.29]**
Test for overall effect: Z = 0.38 (P = 0.70)								
Heterogeneity: Not applicable								
**2.10.7 12 weeks**								
**Subtotal**			**0**			**0**		**Not estimable**
Test for overall effect: Not applicable								
Heterogeneity: Not applicable								
**2.10.8 24 weeks**								
**Subtotal**			**0**			**0**		**Not estimable**
Test for overall effect: Not applicable								
Heterogeneity: Not applicable								
**Total (Wald[a])**			**112**			**113**	**100.0%**	**0.34 [-1.44 , 2.12]**
95% prediction interval								[-2.45 , 3.14]

Test for overall effect: Z = 0.38 (P = 0.71)
Test for subgroup differences: Chi² = 0.02, df = 1 (P = 0.88), I² = 0%
Heterogeneity: Tau² (REML[b], 95% CI) = 1.21 [0.00 , 64.46]; Chi² = 6.79, df = 4 (P = 0.15); I² = 30%

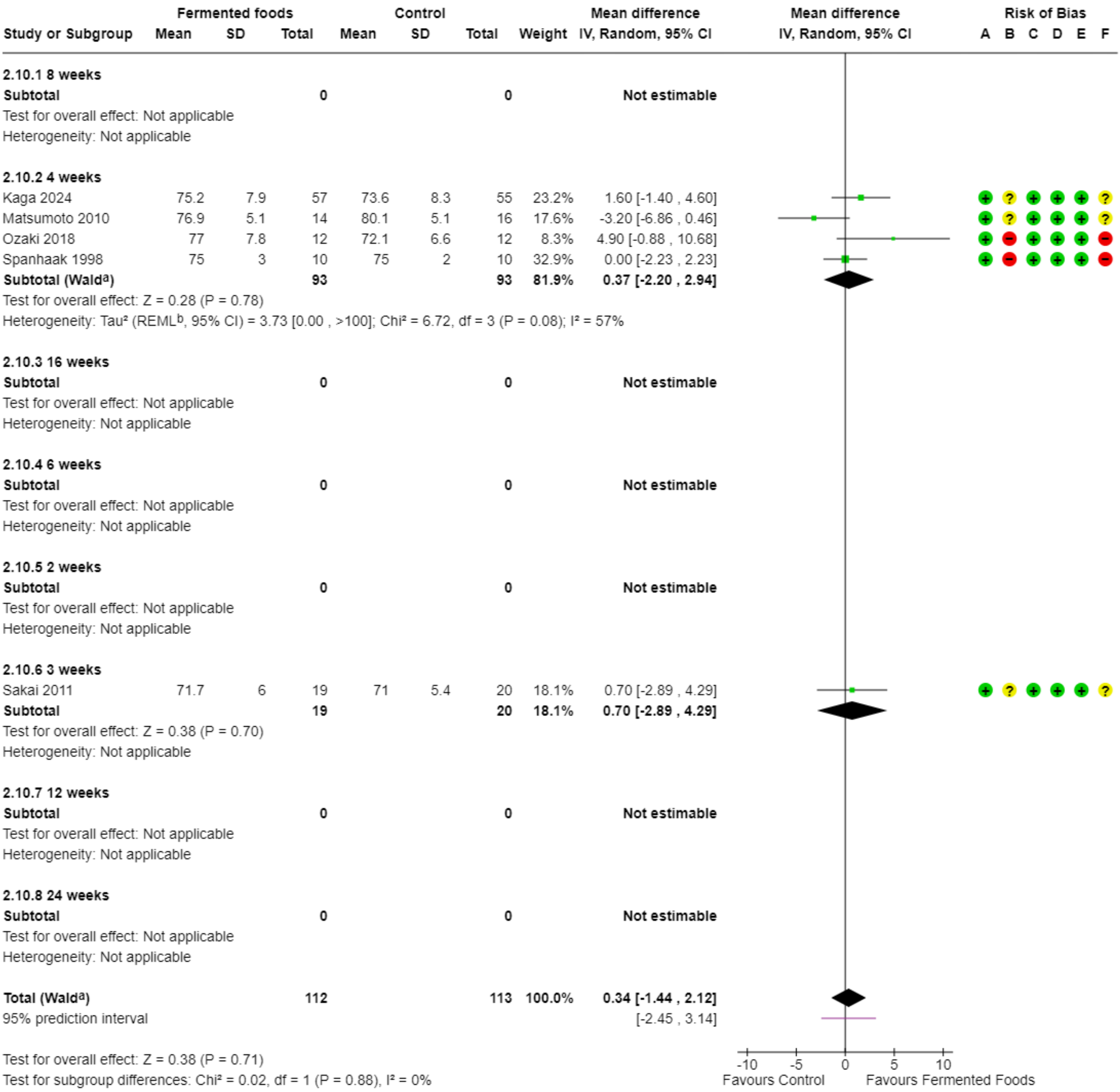

Supplementary Figure S7B. Subgroup analysis based on the intervention duration for stool water content. Forest plot of subgroup analysis based on the intervention duration for stool water content in randomised controlled trials comparing fermented foods with control in healthy adults (n=225). Values were calculated as mean difference (95% CIs) using a random-effects model. CI, confidence interval; IV, inverse variance; SD, standard deviation; [a]CI calculated by Wald-type method; [b]Tau^2 calculated using Restricted Maximum-Likelihood method; Risk of bias legend: (A) bias arising from the randomisation process, (B) bias due to deviations from intended interventions, (C) bias due to missing outcome data, (D) bias in the measurement of the outcome, (E) bias in the selection of the reported result, (F) overall bias.

Study or Subgroup	Fermented foods Mean	Fermented foods SD	Fermented foods Total	Control Mean	Control SD	Control Total	Weight	Mean difference IV, Random, 95% CI	A	B	C	D	E	F
**2.11.1 < 10^10 CFU/day**														
Sakai 2011	71.7	6	19	71	5.4	20	18.1%	0.70 [-2.89 , 4.29]	+	?	+	+	+	?
**Subtotal**			**19**			**20**	**18.1%**	**0.70 [-2.89 , 4.29]**						
Test for overall effect: Z = 0.38 (P = 0.70)														
Heterogeneity: Not applicable														
**2.11.2 >= 10^10 CFU/day**														
Kaga 2024	75.2	7.9	57	73.6	8.3	55	23.2%	1.60 [-1.40 , 4.60]	+	?	+	+	+	?
Matsumoto 2010	76.9	5.1	14	80.1	5.1	16	17.6%	-3.20 [-6.86 , 0.46]	+	?	+	+	+	?
Ozaki 2018	77	7.8	12	72.1	6.6	12	8.3%	4.90 [-0.88 , 10.68]	+	−	+	+	+	−
Spanhaak 1998	75	3	10	75	2	10	32.9%	0.00 [-2.23 , 2.23]	+	−	+	+	+	−
**Subtotal (Wald[a])**			**93**			**93**	**81.9%**	**0.37 [-2.20 , 2.94]**						
Test for overall effect: Z = 0.28 (P = 0.78)														
Heterogeneity: Tau² (REML[b], 95% CI) = 3.73 [0.00 , >100]; Chi² = 6.72, df = 3 (P = 0.08); I² = 57%														
**Total (Wald[a])**			**112**			**113**	**100.0%**	**0.34 [-1.44 , 2.12]**						
95% prediction interval								[-2.45 , 3.14]						
Test for overall effect: Z = 0.38 (P = 0.71)														
Test for subgroup differences: Chi² = 0.02, df = 1 (P = 0.88), I² = 0%														
Heterogeneity: Tau² (REML[b], 95% CI) = 1.21 [0.00 , 64.46]; Chi² = 6.79, df = 4 (P = 0.15); I² = 30%														

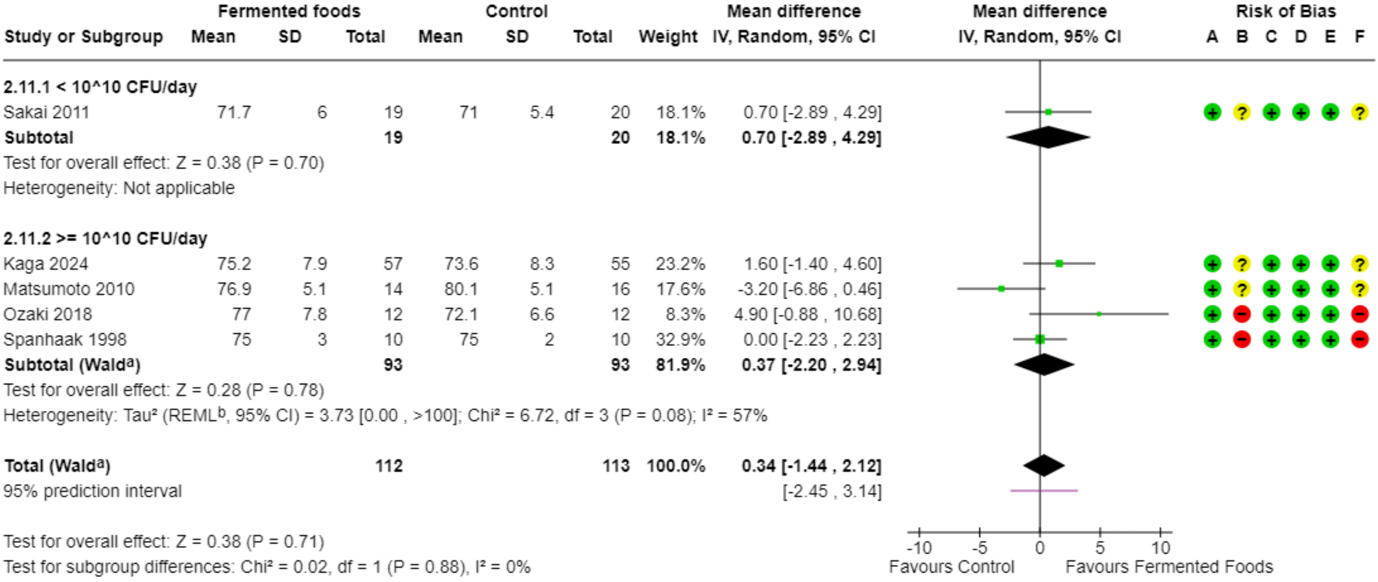

Supplementary Figure S7C. Subgroup analysis based on the microbial dosage for stool water content. Forest plot of subgroup analysis based on the microbial dosage for stool water content in randomised controlled trials comparing fermented foods with control in healthy adults (n=225). Values were calculated as mean difference (95% CIs) using a random-effects model. CI, confidence interval; IV, inverse variance; SD, standard deviation; [a]CI calculated by Wald-type method; [b]Tau^2 calculated using Restricted Maximum-Likelihood method; Risk of bias legend: (A) bias arising from the randomisation process, (B) bias due to deviations from intended interventions, (C) bias due to missing outcome data, (D) bias in the measurement of the outcome, (E) bias in the selection of the reported result, (F) overall bias.

Study or Subgroup	Fermented foods Mean	Fermented foods SD	Fermented foods Total	Control Mean	Control SD	Control Total	Weight	Mean difference IV, Random, 95% CI	Risk of Bias A	B	C	D	E	F
**2.12.1 Bif lactis, St thermo, Lb bulgar**														
**Subtotal**			**0**			**0**		**Not estimable**						
Test for overall effect: Not applicable														
Heterogeneity: Not applicable														
**2.12.2 Bif lactis, Lc cremoris, St thermo, Lb bulgar**														
**Subtotal**			**0**			**0**		**Not estimable**						
Test for overall effect: Not applicable														
Heterogeneity: Not applicable														
**2.12.3 Lactobacillus helveticus CP790**														
**Subtotal**			**0**			**0**		**Not estimable**						
Test for overall effect: Not applicable														
Heterogeneity: Not applicable														
**2.12.4 Aspergillus oryzae**														
**Subtotal**			**0**			**0**		**Not estimable**						
Test for overall effect: Not applicable														
Heterogeneity: Not applicable														
**2.12.5 Lacticaseibacillus casei strain Shirota**														
Kaga 2024	75.2	7.9	57	73.6	8.3	55	23.2%	1.60 [-1.40 , 4.60]	+	?	+	+	+	?
Matsumoto 2010	76.9	5.1	14	80.1	5.1	16	17.6%	-3.20 [-6.86 , 0.46]	+	?	+	+	+	?
Sakai 2011	71.7	6	19	71	5.4	20	18.1%	0.70 [-2.89 , 4.29]	+	?	+	+	+	?
Spanhaak 1998	75	3	10	75	2	10	32.9%	0.00 [-2.23 , 2.23]	+	−	+	+	+	−
**Subtotal (Wald[a])**			**100**			**101**	**91.7%**	**-0.04 [-1.65 , 1.57]**						
Test for overall effect: Z = 0.05 (P = 0.96)														
Heterogeneity: Tau^2 (REML[b], 95% CI) = 0.39 [0.00 , 57.47]; Chi^2 = 4.18, df = 3 (P = 0.24); I^2 = 14%														
**2.12.6 Lb bulgaricus OLL1073R-1, St thermophilus**														
**Subtotal**			**0**			**0**		**Not estimable**						
Test for overall effect: Not applicable														
Heterogeneity: Not applicable														
**2.12.7 COMPLEX**														
**Subtotal**			**0**			**0**		**Not estimable**						
Test for overall effect: Not applicable														
Heterogeneity: Not applicable														
**2.12.8 Lactococcus cremoris FC**														
Ozaki 2018	77	7.8	12	72.1	6.6	12	8.3%	4.90 [-0.88 , 10.68]	+	−	+	+	+	−
**Subtotal**			**12**			**12**	**8.3%**	**4.90 [-0.88 , 10.68]**						
Test for overall effect: Z = 1.66 (P = 0.10)														
Heterogeneity: Not applicable														
**2.12.9 Lactococcus lactis BM32-1**														
**Subtotal**			**0**			**0**		**Not estimable**						
Test for overall effect: Not applicable														
Heterogeneity: Not applicable														
**2.12.10 Levilactobacillus brevis subsp NSB2**														
**Subtotal**			**0**			**0**		**Not estimable**						
Test for overall effect: Not applicable														
Heterogeneity: Not applicable														
**2.12.11 Bif lactis, Lc lactis, St thermo, Lb bulgar**														
**Subtotal**			**0**			**0**		**Not estimable**						
Test for overall effect: Not applicable														
Heterogeneity: Not applicable														
**Total (Wald[a])**			**112**			**113**	**100.0%**	**0.34 [-1.44 , 2.12]**						
95% prediction interval								[-2.45 , 3.14]						

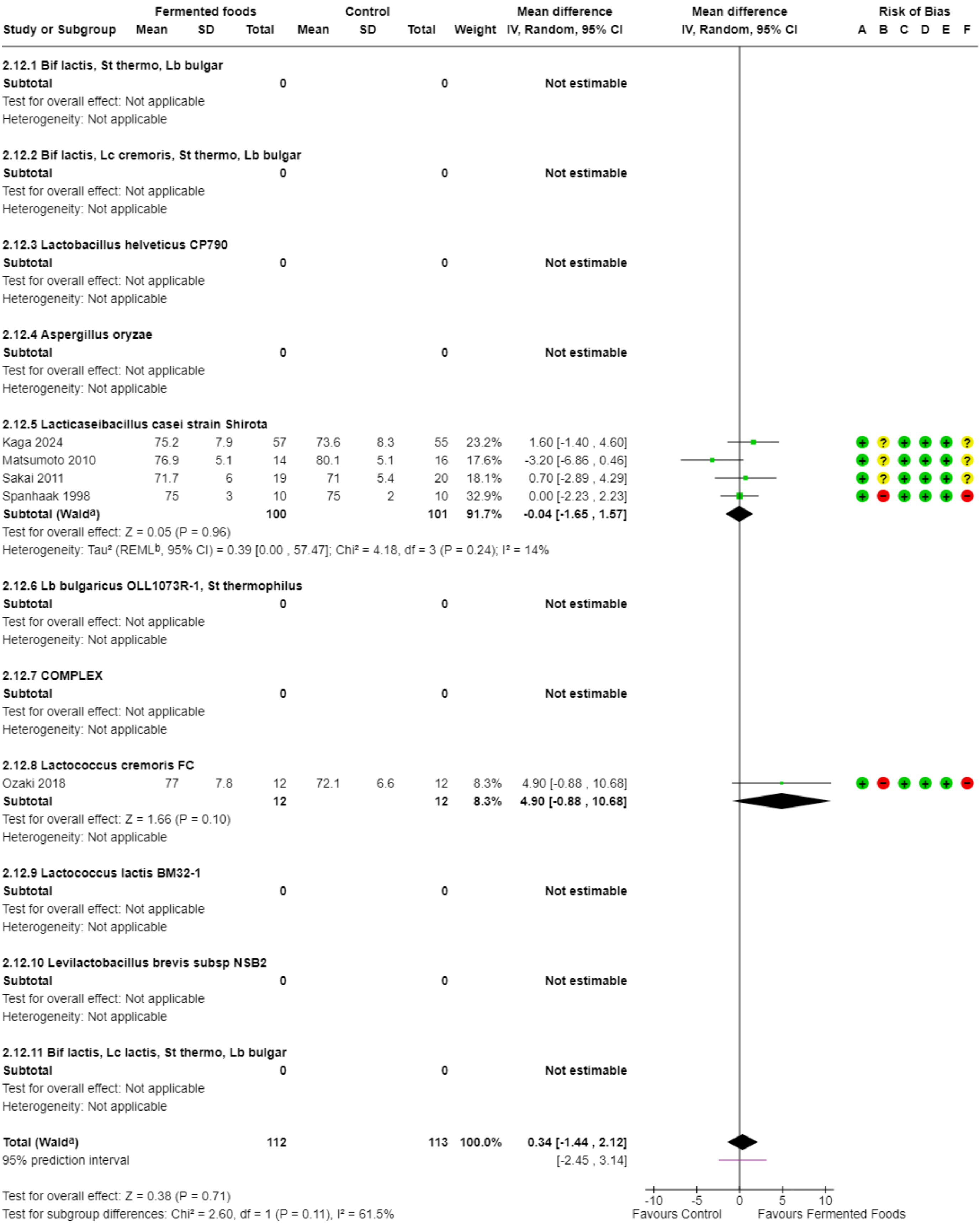

Test for overall effect: Z = 0.38 (P = 0.71)
Test for subgroup differences: Chi^2 = 2.60, df = 1 (P = 0.11), I^2 = 61.5%
Heterogeneity: Tau^2 (REML[b], 95% CI) = 1.21 [0.00 , 64.46]; Chi^2 = 6.79, df = 4 (P = 0.15); I^2 = 30%

Supplementary Figure S7D. Subgroup analysis based on the fermentation microorganisms for stool water content. Forest plot of subgroup analysis based on the fermentation microorganisms for stool water content in randomised controlled trials comparing fermented foods with control in healthy adults (n=225). Values were calculated as mean

difference (95% CIs) using a random-effects model. COMPLEX, complex fermenting microbial community of several microorganisms consisting primarily of lactic acid bacteria; CI, confidence interval; IV, inverse variance; SD, standard deviation; [a]CI calculated by Wald-type method; [b]Tau^2 calculated using Restricted Maximum-Likelihood method; Risk of bias legend: (A) bias arising from the randomisation process, (B) bias due to deviations from intended interventions, (C) bias due to missing outcome data, (D) bias in the measurement of the outcome, (E) bias in the selection of the reported result, (F) overall bias.

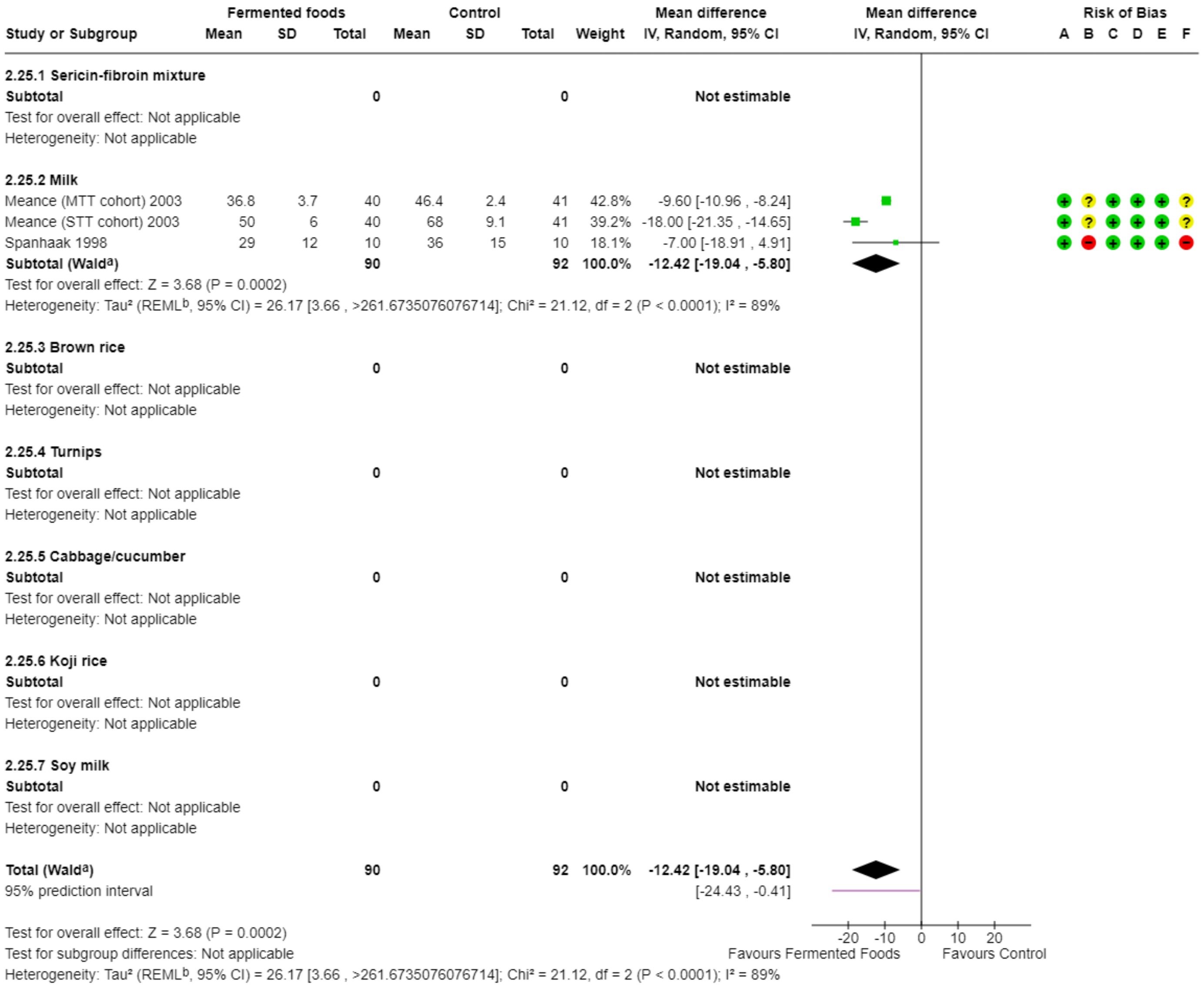

Supplementary Figure S8A. Subgroup analysis based on the fermentation matrix for intestinal transit time. Forest plot of subgroup analysis based on the fermentation matrix for intestinal transit time in randomised controlled trials comparing fermented foods with control in healthy adults (n=182). Values were calculated as mean difference (95% CIs) using a random-effects model. CI, confidence interval; IV, inverse variance; SD, standard deviation; [a]CI calculated by Wald-type method; [b]Tau^2 calculated using Restricted Maximum-Likelihood method; Risk of bias legend: (A) bias arising from the randomisation process, (B) bias due to deviations from intended interventions, (C) bias due to missing outcome data, (D) bias in the measurement of the outcome, (E) bias in the selection of the reported result, (F) overall bias.

Study or Subgroup	Fermented foods Mean	Fermented foods SD	Fermented foods Total	Control Mean	Control SD	Control Total	Weight	Mean difference IV, Random, 95% CI	A	B	C	D	E	F
**2.26.1 8 weeks**														
**Subtotal**			**0**			**0**		**Not estimable**						
Test for overall effect: Not applicable														
Heterogeneity: Not applicable														
**2.26.2 4 weeks**														
Spanhaak 1998	29	12	10	36	15	10	18.1%	-7.00 [-18.91 , 4.91]	+	-	+	+	+	-
**Subtotal**			**10**			**10**	**18.1%**	**-7.00 [-18.91 , 4.91]**						
Test for overall effect: Z = 1.15 (P = 0.25)														
Heterogeneity: Not applicable														
**2.26.3 16 weeks**														
**Subtotal**			**0**			**0**		**Not estimable**						
Test for overall effect: Not applicable														
Heterogeneity: Not applicable														
**2.26.4 6 weeks**														
**Subtotal**			**0**			**0**		**Not estimable**						
Test for overall effect: Not applicable														
Heterogeneity: Not applicable														
**2.26.5 2 weeks**														
Meance (MTT cohort) 2003	36.8	3.7	40	46.4	2.4	41	42.8%	-9.60 [-10.96 , -8.24]	+	?	+	+	+	?
Meance (STT cohort) 2003	50	6	40	68	9.1	41	39.2%	-18.00 [-21.35 , -14.65]	+	?	+	+	+	?
**Subtotal (Wald[a])**			**80**			**82**	**81.9%**	**-13.65 [-21.88 , -5.43]**						
Test for overall effect: Z = 3.25 (P = 0.001)														
Heterogeneity: Tau² (REML[b], 95% CI) = 33.58 [5.32 , >335.78753048780464]; Chi² = 20.74, df = 1 (P < 0.00001); I² = 95%														
**2.26.6 3 weeks**														
**Subtotal**			**0**			**0**		**Not estimable**						
Test for overall effect: Not applicable														
Heterogeneity: Not applicable														
**2.26.7 12 weeks**														
**Subtotal**			**0**			**0**		**Not estimable**						
Test for overall effect: Not applicable														
Heterogeneity: Not applicable														
**2.26.8 24 weeks**														
**Subtotal**			**0**			**0**		**Not estimable**						
Test for overall effect: Not applicable														
Heterogeneity: Not applicable														
**Total (Wald[a])**			**90**			**92**	**100.0%**	**-12.42 [-19.04 , -5.80]**						
95% prediction interval								[-24.43 , -0.41]						

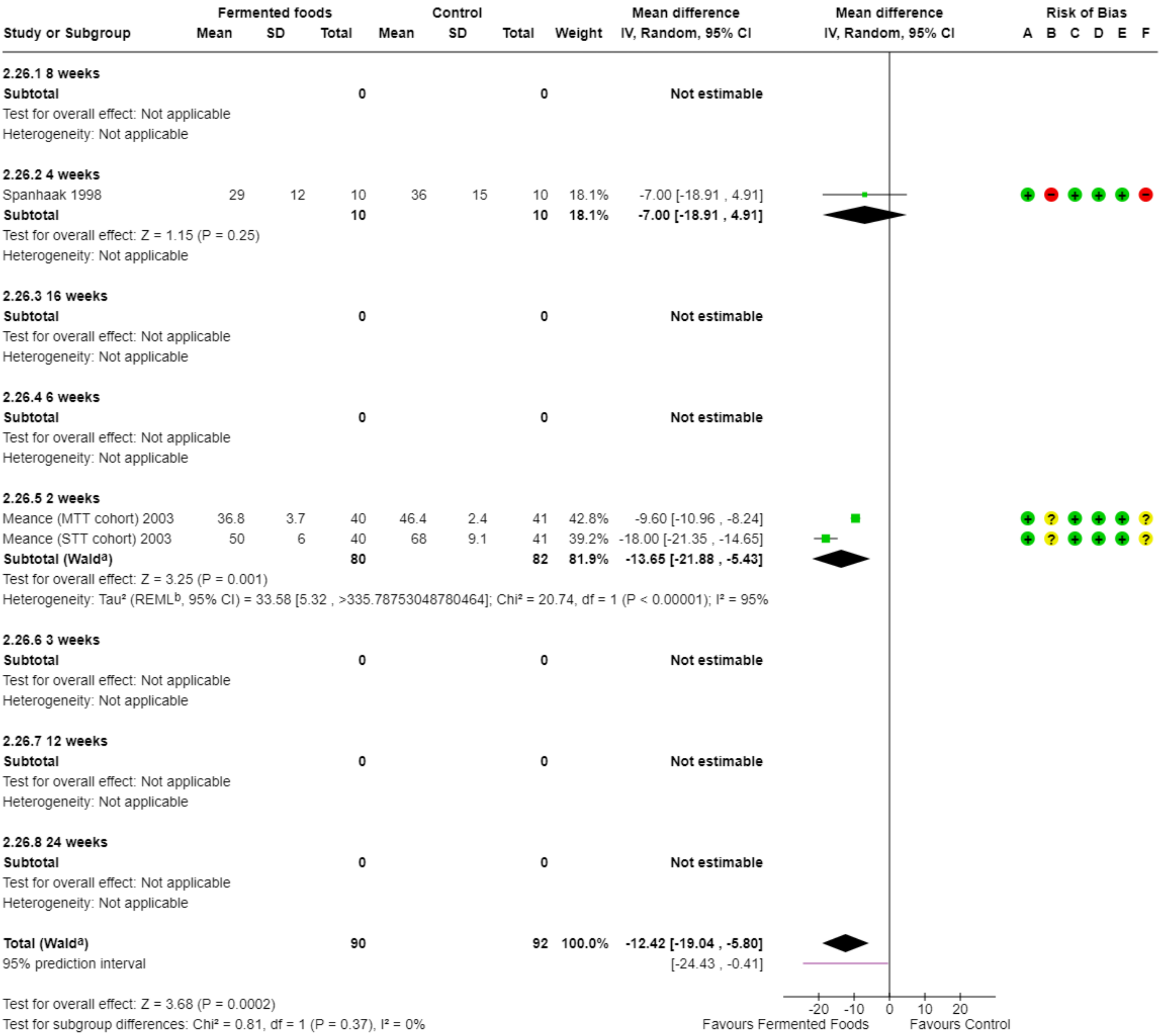

Test for overall effect: Z = 3.68 (P = 0.0002)
Test for subgroup differences: Chi² = 0.81, df = 1 (P = 0.37), I² = 0%
Heterogeneity: Tau² (REML[b], 95% CI) = 26.17 [3.66 , >261.6735076076714]; Chi² = 21.12, df = 2 (P < 0.0001); I² = 89%

Supplementary Figure S8B. Subgroup analysis based on the intervention duration for intestinal transit time. Forest plot of subgroup analysis based on the intervention duration for intestinal transit time in randomised controlled trials comparing fermented foods with control in healthy adults (n=182). Values were calculated as mean difference (95% CIs) using a random-effects model. CI, confidence interval; IV, inverse variance; SD, standard deviation; [a]CI calculated by Wald-type method; [b]Tau^2 calculated using Restricted Maximum-Likelihood method; Risk of bias legend: (A) bias arising from the randomisation process, (B) bias due to deviations from intended interventions, (C) bias due to missing outcome data, (D) bias in the measurement of the outcome, (E) bias in the selection of the reported result, (F) overall bias.

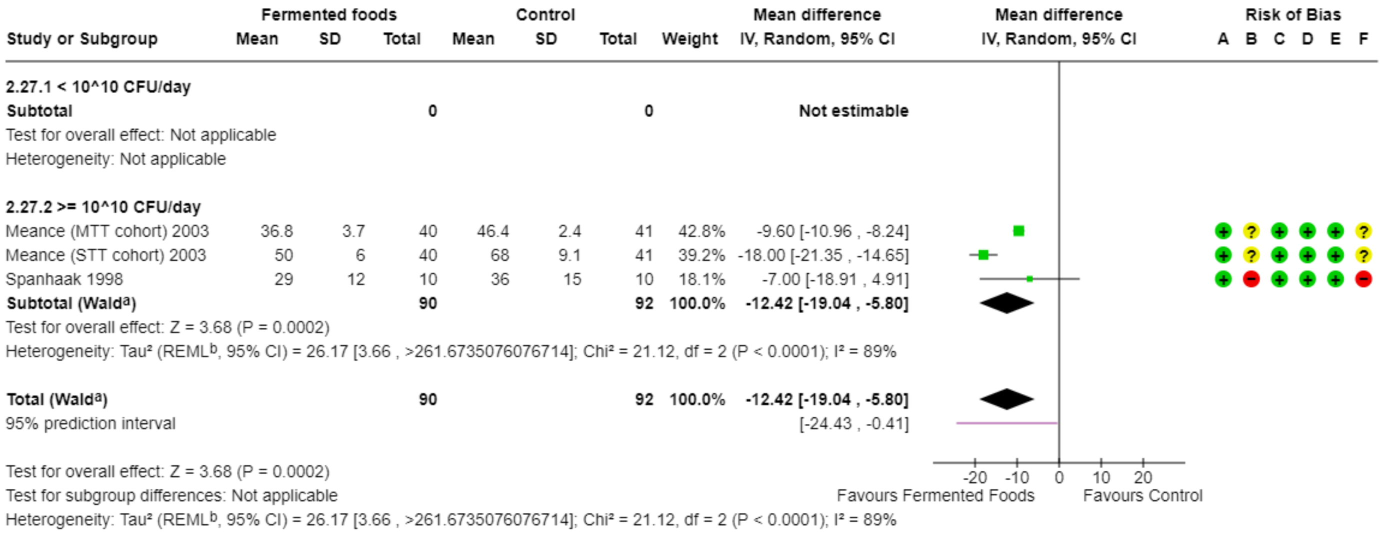

Supplementary Figure S8C. Subgroup analysis based on the microbial dosage for intestinal transit time. Forest plot of subgroup analysis based on the microbial dosage for intestinal transit time in randomised controlled trials comparing fermented foods with control in healthy adults (n=182). Values were calculated as mean difference (95% CIs) using a random-effects model. CI, confidence interval; IV, inverse variance; SD, standard deviation; [a]CI calculated by Wald-type method; [b]Tau^2 calculated using Restricted Maximum-Likelihood method; Risk of bias legend: (A) bias arising from the randomisation process, (B) bias due to deviations from intended interventions, (C) bias due to missing outcome data, (D) bias in the measurement of the outcome, (E) bias in the selection of the reported result, (F) overall bias.

Study or Subgroup	Fermented foods Mean	SD	Total	Control Mean	SD	Total	Weight	Mean difference IV, Random, 95% CI	Risk of Bias A	B	C	D	E	F
**2.28.1 Bif lactis, St thermo, Lb bulgar**														
Meance (MTT cohort) 2003	36.8	3.7	40	46.4	2.4	41	42.8%	-9.60 [-10.96 , -8.24]	+	?	+	+	+	?
Meance (STT cohort) 2003	50	6	40	68	9.1	41	39.2%	-18.00 [-21.35 , -14.65]	+	?	+	+	+	?
**Subtotal (Wald[a])**			**80**			**82**	**81.9%**	**-13.65 [-21.88 , -5.43]**						
Test for overall effect: Z = 3.25 (P = 0.001)														
Heterogeneity: Tau² (REML[b], 95% CI) = 33.58 [5.32 , >335.78753048780464]; Chi² = 20.74, df = 1 (P < 0.00001); I² = 95%														
**2.28.2 Bif lactis, Lc cremoris, St thermo, Lb bulgar**														
**Subtotal**			**0**			**0**		**Not estimable**						
Test for overall effect: Not applicable														
Heterogeneity: Not applicable														
**2.28.3 Lactobacillus helveticus CP790**														
**Subtotal**			**0**			**0**		**Not estimable**						
Test for overall effect: Not applicable														
Heterogeneity: Not applicable														
**2.28.4 Aspergillus oryzae**														
**Subtotal**			**0**			**0**		**Not estimable**						
Test for overall effect: Not applicable														
Heterogeneity: Not applicable														
**2.28.5 Lacticaseibacillus casei strain Shirota**														
Spanhaak 1998	29	12	10	36	15	10	18.1%	-7.00 [-18.91 , 4.91]	+	-	+	+	+	-
**Subtotal**			**10**			**10**	**18.1%**	**-7.00 [-18.91 , 4.91]**						
Test for overall effect: Z = 1.15 (P = 0.25)														
Heterogeneity: Not applicable														
**2.28.6 Lb bulgaricus OLL1073R-1, St thermophilus**														
**Subtotal**			**0**			**0**		**Not estimable**						
Test for overall effect: Not applicable														
Heterogeneity: Not applicable														
**2.28.7 COMPLEX**														
**Subtotal**			**0**			**0**		**Not estimable**						
Test for overall effect: Not applicable														
Heterogeneity: Not applicable														
**2.28.8 Lactococcus cremoris FC**														
**Subtotal**			**0**			**0**		**Not estimable**						
Test for overall effect: Not applicable														
Heterogeneity: Not applicable														
**2.28.9 Lactococcus lactis BM32-1**														
**Subtotal**			**0**			**0**		**Not estimable**						
Test for overall effect: Not applicable														
Heterogeneity: Not applicable														
**2.28.10 Levilactobacillus brevis subsp NSB2**														
**Subtotal**			**0**			**0**		**Not estimable**						
Test for overall effect: Not applicable														
Heterogeneity: Not applicable														
**2.28.11 Bif lactis, Lc lactis, St thermo, Lb bulgar**														
**Subtotal**			**0**			**0**		**Not estimable**						
Test for overall effect: Not applicable														
Heterogeneity: Not applicable														
**Total (Wald[a])**			**90**			**92**	**100.0%**	**-12.42 [-19.04 , -5.80]**						
95% prediction interval								[-24.43 , -0.41]						
Test for overall effect: Z = 3.68 (P = 0.0002)														
Test for subgroup differences: Chi² = 0.81, df = 1 (P = 0.37), I² = 0%														
Heterogeneity: Tau² (REML[b], 95% CI) = 26.17 [3.66 , >261.6735076076714]; Chi² = 21.12, df = 2 (P < 0.0001); I² = 89%														

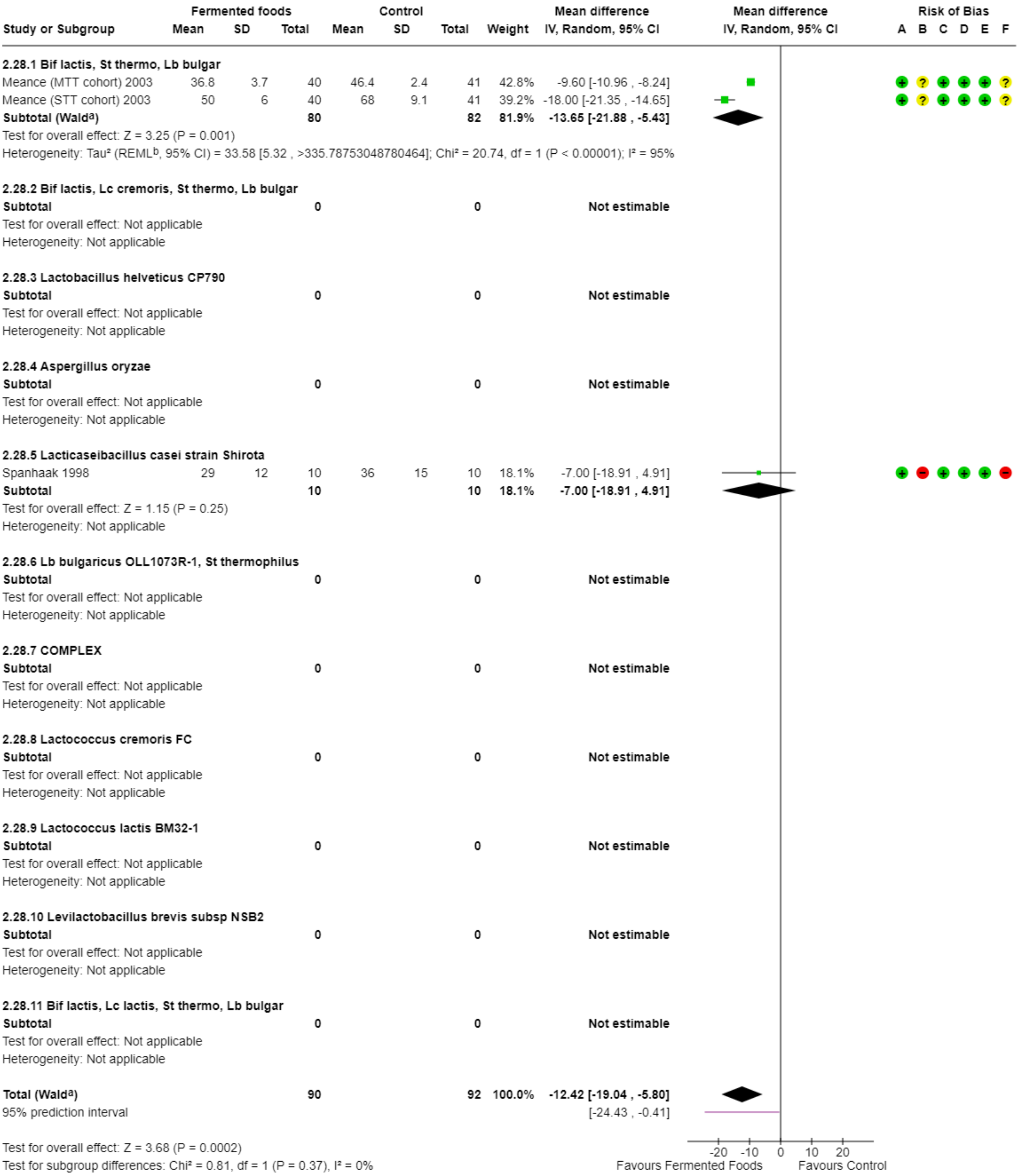

Supplementary Figure S8D. Subgroup analysis based on the fermentation microorganisms for intestinal transit time. Forest plot of subgroup analysis based on the fermentation microorganisms for intestinal transit time in randomised controlled trials comparing fermented foods with control in healthy adults (n=182). Values were calculated as mean difference (95% CIs) using a random-effects model. COMPLEX, complex fermenting microbial community of several microorganisms consisting primarily of lactic acid bacteria; CI, confidence interval; IV, inverse variance; SD, standard deviation; [a]CI calculated by Wald-type method; [b]Tau² calculated using Restricted Maximum-Likelihood method; Risk of bias legend: (A) bias arising from the randomisation process, (B) bias due to deviations from intended interventions,

(C) bias due to missing outcome data, (D) bias in the measurement of the outcome, (E) bias in the selection of the reported result, (F) overall bias.

Study or Subgroup	Fermented foods Mean	SD	Total	Control Mean	SD	Total	Weight	Std. mean difference IV, Random, 95% CI	A	B	C	D	E	F
**4.5.1 Sericin-fibroin mixture**														
**Subtotal**			**0**			**0**		**Not estimable**						
Test for overall effect: Not applicable														
Heterogeneity: Not applicable														
**4.5.2 Milk**														
Guyonnet (1-pot) 2009	2.07	0.77	144	2.61	0.75	35	16.9%	-0.70 [-1.08 , -0.33]	+	+	+	+	+	+
Guyonnet (2-pot) 2009	2.03	0.75	147	2.61	0.75	35	16.9%	-0.77 [-1.15 , -0.39]	+	+	+	+	+	+
Guyonnet 2009	4.52	2.99	100	5.36	2.95	97	17.8%	-0.28 [-0.56 , -0.00]	+	+	+	+	+	+
Kato-Kataoka 2016	2.35	0.67	22	3.54	0.59	24	13.0%	-1.86 [-2.56 , -1.16]	+	?	+	+	+	?
Marteau 2013	5.32	2.46	162	5.64	2.76	162	18.3%	-0.12 [-0.34 , 0.10]	+	+	+	+	+	+
Tanihiro 2024	0.16	0.38	57	0.24	0.39	60	17.0%	-0.21 [-0.57 , 0.16]	+	+	+	+	+	+
**Subtotal (Wald[a])**			**632**			**413**	**100.0%**	**-0.60 [-1.05 , -0.15]**						
Test for overall effect: Z = 2.62 (P = 0.009)														
Heterogeneity: Tau² (REML[b], 95% CI) = 0.27 [0.08 , 2.42]; Chi² = 30.68, df = 5 (P < 0.0001); I² = 90%														
**4.5.3 Brown rice**														
**Subtotal**			**0**			**0**		**Not estimable**						
Test for overall effect: Not applicable														
Heterogeneity: Not applicable														
**4.5.4 Turnips**														
**Subtotal**			**0**			**0**		**Not estimable**						
Test for overall effect: Not applicable														
Heterogeneity: Not applicable														
**4.5.5 Cabbage/cucumber**														
**Subtotal**			**0**			**0**		**Not estimable**						
Test for overall effect: Not applicable														
Heterogeneity: Not applicable														
**4.5.6 Koji rice**														
**Subtotal**			**0**			**0**		**Not estimable**						
Test for overall effect: Not applicable														
Heterogeneity: Not applicable														
**4.5.7 Soy milk**														
**Subtotal**			**0**			**0**		**Not estimable**						
Test for overall effect: Not applicable														
Heterogeneity: Not applicable														
**Total (Wald[a])**			**632**			**413**	**100.0%**	**-0.60 [-1.05 , -0.15]**						
95% prediction interval								[-1.71 , 0.52]						
Test for overall effect: Z = 2.62 (P = 0.009)														
Test for subgroup differences: Not applicable														
Heterogeneity: Tau² (REML[b], 95% CI) = 0.27 [0.08 , 2.42]; Chi² = 30.68, df = 5 (P < 0.0001); I² = 90%														

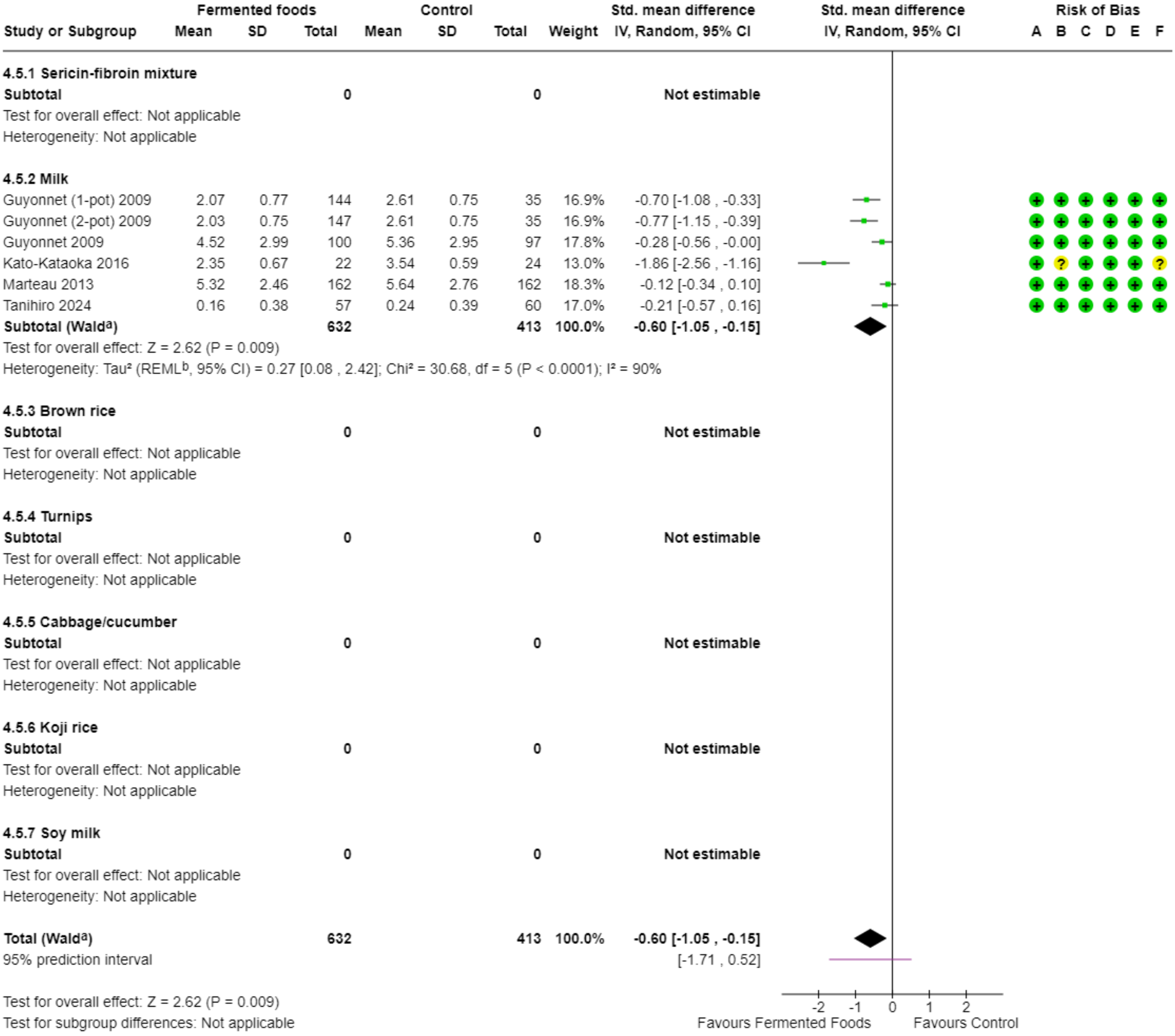

Supplementary Figure S9A. Subgroup analysis based on the fermentation matrix for abdominal symptoms. Forest plot of subgroup analysis based on the fermentation matrix for abdominal symptoms in randomised controlled trials comparing fermented foods with control in healthy adults (n=1,045). Values were calculated as standardised mean difference (95% CIs) using a random-effects model. CI, confidence interval; IV, inverse variance; SD, standard deviation; [a]CI calculated by Wald-type method; [b]Tau^2 calculated using Restricted Maximum-Likelihood method; Risk of bias legend: (A) bias arising from the randomisation process, (B) bias due to deviations from intended interventions, (C) bias due to missing outcome data, (D) bias in the measurement of the outcome, (E) bias in the selection of the reported result, (F) overall bias.

Study or Subgroup	Fermented foods Mean	SD	Total	Control Mean	SD	Total	Weight	Std. mean difference IV, Random, 95% CI
**4.6.1 8 weeks**								
Kato-Kataoka 2016	2.35	0.67	22	3.54	0.59	24	13.0%	-1.86 [-2.56 , -1.16]
**Subtotal**			**22**			**24**	**13.0%**	**-1.86 [-2.56 , -1.16]**
Test for overall effect: Z = 5.19 (P < 0.00001)								
Heterogeneity: Not applicable								
**4.6.2 4 weeks**								
Guyonnet 2009	4.52	2.99	100	5.36	2.95	97	17.8%	-0.28 [-0.56 , -0.00]
Marteau 2013	5.32	2.46	162	5.64	2.76	162	18.3%	-0.12 [-0.34 , 0.10]
Tanihiro 2024	0.16	0.38	57	0.24	0.39	60	17.0%	-0.21 [-0.57 , 0.16]
**Subtotal (Wald[a])**			**319**			**319**	**53.2%**	**-0.19 [-0.34 , -0.03]**
Test for overall effect: Z = 2.35 (P = 0.02)								
Heterogeneity: Tau² (REML[b], 95% CI) = 0.00 [0.00 , 0.24]; Chi² = 0.79, df = 2 (P = 0.67); I² = 0%								
**4.6.3 16 weeks**								
**Subtotal**			**0**			**0**		**Not estimable**
Test for overall effect: Not applicable								
Heterogeneity: Not applicable								
**4.6.4 6 weeks**								
**Subtotal**			**0**			**0**		**Not estimable**
Test for overall effect: Not applicable								
Heterogeneity: Not applicable								
**4.6.5 2 weeks**								
Guyonnet (1-pot) 2009	2.07	0.77	144	2.61	0.75	35	16.9%	-0.70 [-1.08 , -0.33]
Guyonnet (2-pot) 2009	2.03	0.75	147	2.61	0.75	35	16.9%	-0.77 [-1.15 , -0.39]
**Subtotal (Wald[a])**			**291**			**70**	**33.8%**	**-0.74 [-1.00 , -0.47]**
Test for overall effect: Z = 5.41 (P < 0.00001)								
Heterogeneity: Tau² (REML[b], 95% CI) = 0.00 [0.00 , 2.34]; Chi² = 0.06, df = 1 (P = 0.80); I² = 0%								
**4.6.6 3 weeks**								
**Subtotal**			**0**			**0**		**Not estimable**
Test for overall effect: Not applicable								
Heterogeneity: Not applicable								
**4.6.7 12 weeks**								
**Subtotal**			**0**			**0**		**Not estimable**
Test for overall effect: Not applicable								
Heterogeneity: Not applicable								
**4.6.8 24 weeks**								
**Subtotal**			**0**			**0**		**Not estimable**
Test for overall effect: Not applicable								
Heterogeneity: Not applicable								
**Total (Wald[a])**			**632**			**413**	**100.0%**	**-0.60 [-1.05 , -0.15]**
95% prediction interval								[-1.71 , 0.52]

Test for overall effect: Z = 2.62 (P = 0.009)
Test for subgroup differences: Chi² = 29.83, df = 2 (P < 0.00001), I² = 93.3%
Heterogeneity: Tau² (REML[b], 95% CI) = 0.27 [0.08 , 2.42]; Chi² = 30.68, df = 5 (P < 0.0001); I² = 90%

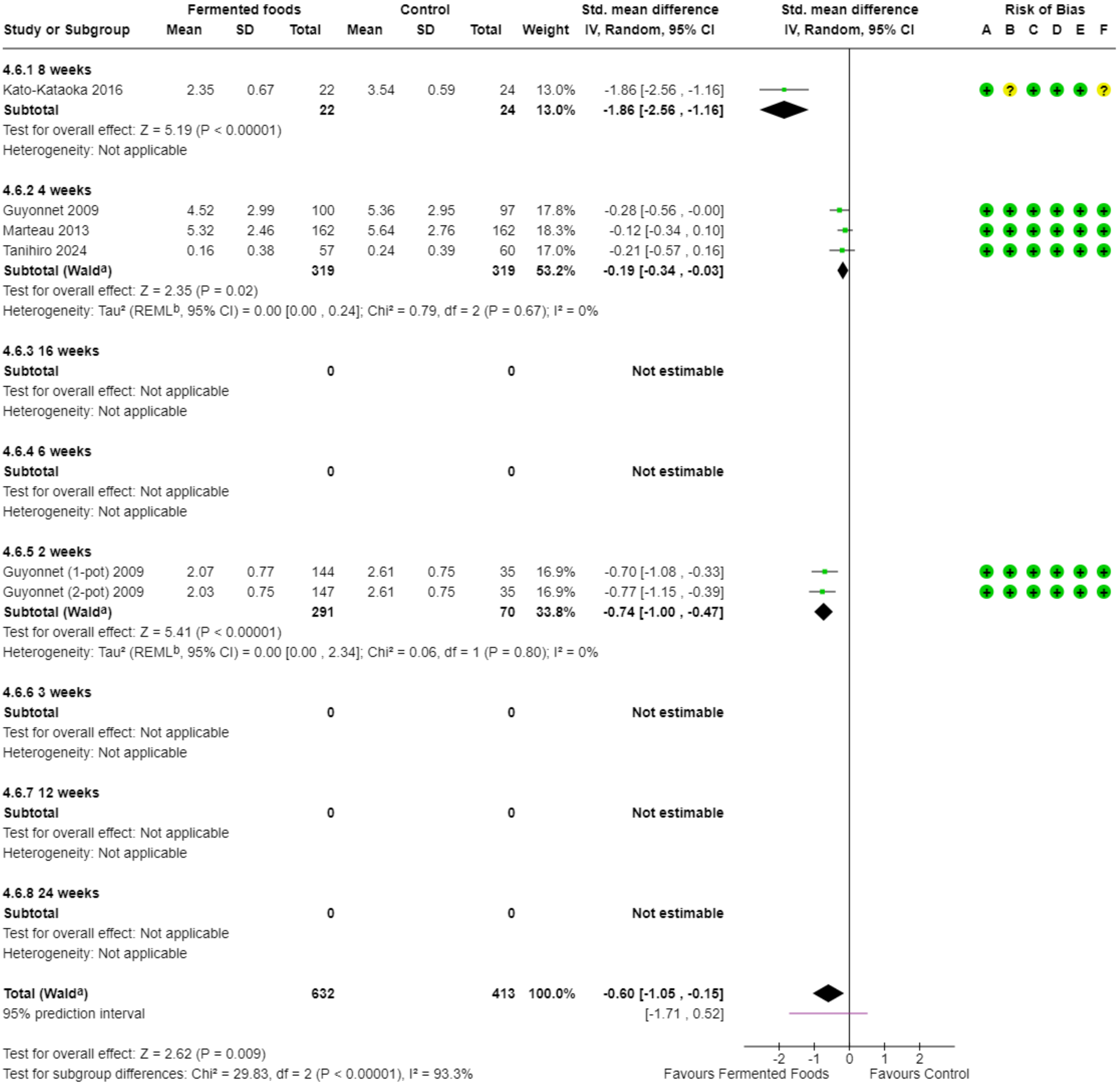

Supplementary Figure S9B. Subgroup analysis based on the intervention duration for abdominal symptoms. Forest plot of subgroup analysis based on the intervention duration for abdominal symptoms in randomised controlled trials comparing fermented foods with control in healthy adults (n=1,045). Values were calculated as standardised mean difference (95% CIs) using a random-effects model. CI, confidence interval; IV, inverse variance; SD, standard deviation; [a]CI calculated by Wald-type method; [b]Tau^2 calculated using Restricted Maximum-Likelihood method; Risk of bias legend: (A) bias arising from the randomisation process, (B) bias due to deviations from intended interventions, (C) bias due to missing outcome data, (D) bias in the measurement of the outcome, (E) bias in the selection of the reported result, (F) overall bias.

Study or Subgroup	Fermented foods Mean	Fermented foods SD	Fermented foods Total	Control Mean	Control SD	Control Total	Weight	Std. mean difference IV, Random, 95% CI	Risk of Bias A	B	C	D	E	F
**4.7.1 < 10^10 CFU/day**														
**Subtotal**			**0**			**0**		**Not estimable**						
Test for overall effect: Not applicable														
Heterogeneity: Not applicable														
**4.7.2 >= 10^10 CFU/day**														
Guyonnet (1-pot) 2009	2.07	0.77	144	2.61	0.75	35	16.9%	-0.70 [-1.08 , -0.33]	+	+	+	+	+	+
Guyonnet (2-pot) 2009	2.03	0.75	147	2.61	0.75	35	16.9%	-0.77 [-1.15 , -0.39]	+	+	+	+	+	+
Guyonnet 2009	4.52	2.99	100	5.36	2.95	97	17.8%	-0.28 [-0.56 , -0.00]	+	+	+	+	+	+
Kato-Kataoka 2016	2.35	0.67	22	3.54	0.59	24	13.0%	-1.86 [-2.56 , -1.16]	+	?	+	+	+	?
Marteau 2013	5.32	2.46	162	5.64	2.76	162	18.3%	-0.12 [-0.34 , 0.10]	+	+	+	+	+	+
Tanihiro 2024	0.16	0.38	57	0.24	0.39	60	17.0%	-0.21 [-0.57 , 0.16]	+	+	+	+	+	+
**Subtotal (Wald[a])**			**632**			**413**	**100.0%**	**-0.60 [-1.05 , -0.15]**						
Test for overall effect: Z = 2.62 (P = 0.009)														
Heterogeneity: Tau² (REML[b], 95% CI) = 0.27 [0.08 , 2.42]; Chi² = 30.68, df = 5 (P < 0.0001); I² = 90%														
**Total (Wald[a])**			**632**			**413**	**100.0%**	**-0.60 [-1.05 , -0.15]**						
95% prediction interval								[-1.71 , 0.52]						
Test for overall effect: Z = 2.62 (P = 0.009)														
Test for subgroup differences: Not applicable														
Heterogeneity: Tau² (REML[b], 95% CI) = 0.27 [0.08 , 2.42]; Chi² = 30.68, df = 5 (P < 0.0001); I² = 90%														

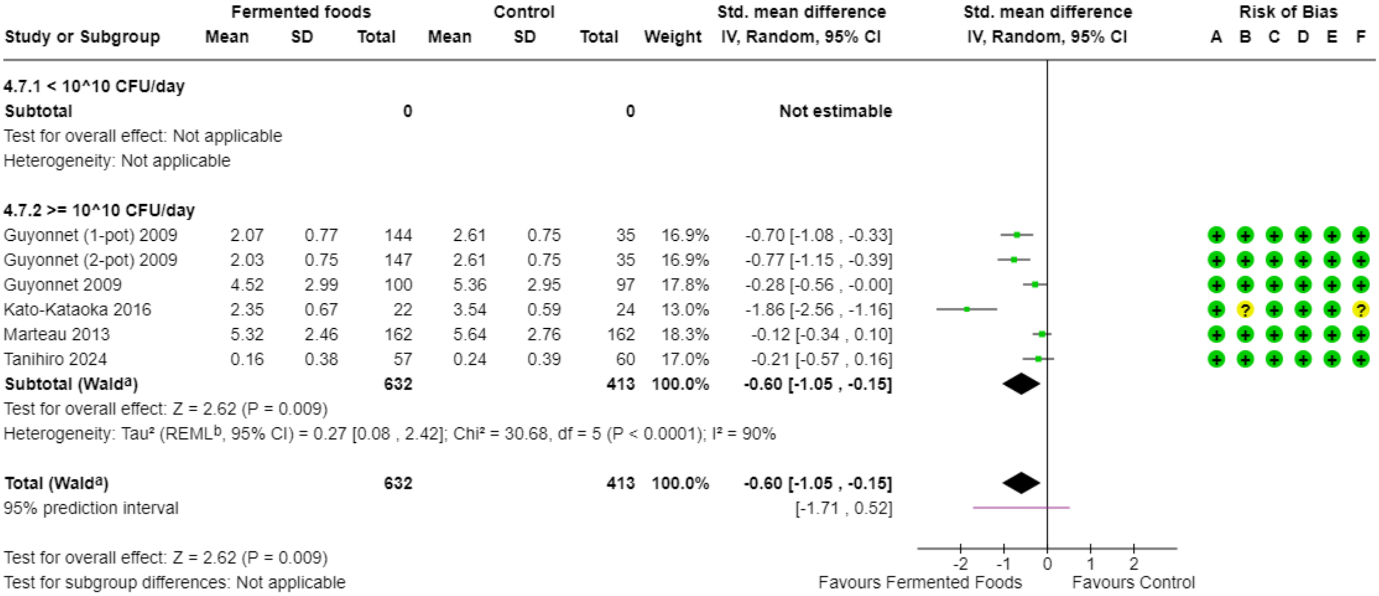

Supplementary Figure S9C. Subgroup analysis based on the microbial dosage for abdominal symptoms. Forest plot of subgroup analysis based on the microbial dosage for abdominal symptoms in randomised controlled trials comparing fermented foods with control in healthy adults (n=1,045). Values were calculated as standardised mean difference (95% CIs) using a random-effects model. CI, confidence interval; IV, inverse variance; SD, standard deviation; [a]CI calculated by Wald-type method; [b]Tau^2 calculated using Restricted Maximum-Likelihood method; Risk of bias legend: (A) bias arising from the randomisation process, (B) bias due to deviations from intended interventions, (C) bias due to missing outcome data, (D) bias in the measurement of the outcome, (E) bias in the selection of the reported result, (F) overall bias.

Study or Subgroup	Fermented foods Mean	Fermented foods SD	Fermented foods Total	Control Mean	Control SD	Control Total	Weight	Std. mean difference IV, Random, 95% CI	Risk of Bias A	B	C	D	E	F
**4.8.1 Bif lactis, St thermo, Lb bulgar**														
Guyonnet (1-pot) 2009	2.07	0.77	144	2.61	0.75	35	16.9%	-0.70 [-1.08 , -0.33]	+	+	+	+	+	+
Guyonnet (2-pot) 2009	2.03	0.75	147	2.61	0.75	35	16.9%	-0.77 [-1.15 , -0.39]	+	+	+	+	+	+
**Subtotal (Wald[a])**			**291**			**70**	**33.8%**	**-0.74 [-1.00 , -0.47]**						
Test for overall effect: Z = 5.41 (P < 0.00001)														
Heterogeneity: Tau² (REML[b], 95% CI) = 0.00 [0.00 , 2.34]; Chi² = 0.06, df = 1 (P = 0.80); I² = 0%														
**4.8.2 Bif lactis, Lc cremoris, St thermo, Lb bulgar**														
Guyonnet 2009	4.52	2.99	100	5.36	2.95	97	17.8%	-0.28 [-0.56 , -0.00]	+	+	+	+	+	+
**Subtotal**			**100**			**97**	**17.8%**	**-0.28 [-0.56 , -0.00]**						
Test for overall effect: Z = 1.97 (P = 0.05)														
Heterogeneity: Not applicable														
**4.8.3 Lactobacillus helveticus CP790**														
Tanihiro 2024	0.16	0.38	57	0.24	0.39	60	17.0%	-0.21 [-0.57 , 0.16]	+	+	+	+	+	+
**Subtotal**			**57**			**60**	**17.0%**	**-0.21 [-0.57 , 0.16]**						
Test for overall effect: Z = 1.11 (P = 0.27)														
Heterogeneity: Not applicable														
**4.8.4 Aspergillus oryzae**														
**Subtotal**			**0**			**0**		**Not estimable**						
Test for overall effect: Not applicable														
Heterogeneity: Not applicable														
**4.8.5 Lacticaseibacillus casei strain Shirota**														
Kato-Kataoka 2016	2.35	0.67	22	3.54	0.59	24	13.0%	-1.86 [-2.56 , -1.16]	+	?	+	+	+	?
**Subtotal**			**22**			**24**	**13.0%**	**-1.86 [-2.56 , -1.16]**						
Test for overall effect: Z = 5.19 (P < 0.00001)														
Heterogeneity: Not applicable														
**4.8.6 Lb bulgaricus OLL1073R-1, St thermophilus**														
**Subtotal**			**0**			**0**		**Not estimable**						
Test for overall effect: Not applicable														
Heterogeneity: Not applicable														
**4.8.7 COMPLEX**														
**Subtotal**			**0**			**0**		**Not estimable**						
Test for overall effect: Not applicable														
Heterogeneity: Not applicable														
**4.8.8 Lactococcus cremoris FC**														
**Subtotal**			**0**			**0**		**Not estimable**						
Test for overall effect: Not applicable														
Heterogeneity: Not applicable														
**4.8.9 Lactococcus lactis BM32-1**														
**Subtotal**			**0**			**0**		**Not estimable**						
Test for overall effect: Not applicable														
Heterogeneity: Not applicable														
**4.8.10 Levilactobacillus brevis subsp NSB2**														
**Subtotal**			**0**			**0**		**Not estimable**						
Test for overall effect: Not applicable														
Heterogeneity: Not applicable														
**4.8.11 Bif lactis, Lc lactis, St thermo, Lb bulgar**														
Marteau 2013	5.32	2.46	162	5.64	2.76	162	18.3%	-0.12 [-0.34 , 0.10]	+	+	+	+	+	+
**Subtotal**			**162**			**162**	**18.3%**	**-0.12 [-0.34 , 0.10]**						
Test for overall effect: Z = 1.10 (P = 0.27)														
Heterogeneity: Not applicable														
**Total (Wald[a])**			**632**			**413**	**100.0%**	**-0.60 [-1.05 , -0.15]**						
95% prediction interval								[-1.71 , 0.52]						

Test for overall effect: Z = 2.62 (P = 0.009)
Test for subgroup differences: Chi² = 30.62, df = 4 (P < 0.00001), I² = 86.9%
Heterogeneity: Tau² (REML[b], 95% CI) = 0.27 [0.08 , 2.42]; Chi² = 30.68, df = 5 (P < 0.0001); I² = 90%

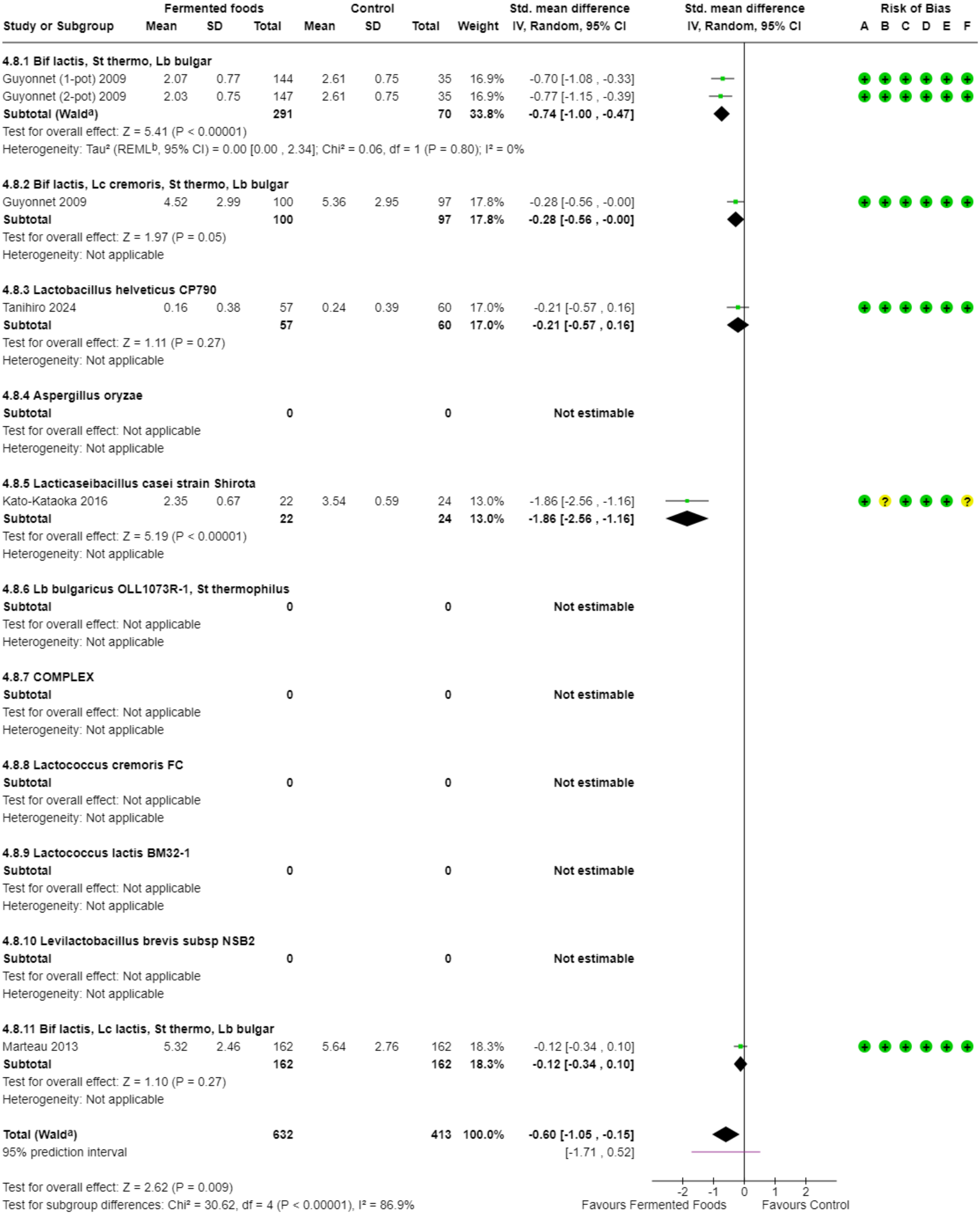

Supplementary Figure S9D. Subgroup analysis based on the fermentation microorganisms for abdominal symptoms. Forest plot of subgroup analysis based on the fermentation microorganisms for abdominal symptoms in randomised controlled trials comparing fermented foods with control in healthy adults (n=1,045). Values were calculated as

standardised mean difference (95% CIs) using a random-effects model. COMPLEX, complex fermenting microbial community of several microorganisms consisting primarily of lactic acid bacteria; CI, confidence interval; IV, inverse variance; SD, standard deviation; [a]CI calculated by Wald-type method; [b]Tau^2 calculated using Restricted Maximum-Likelihood method; Risk of bias legend: (A) bias arising from the randomisation process, (B) bias due to deviations from intended interventions, (C) bias due to missing outcome data, (D) bias in the measurement of the outcome, (E) bias in the selection of the reported result, (F) overall bias.

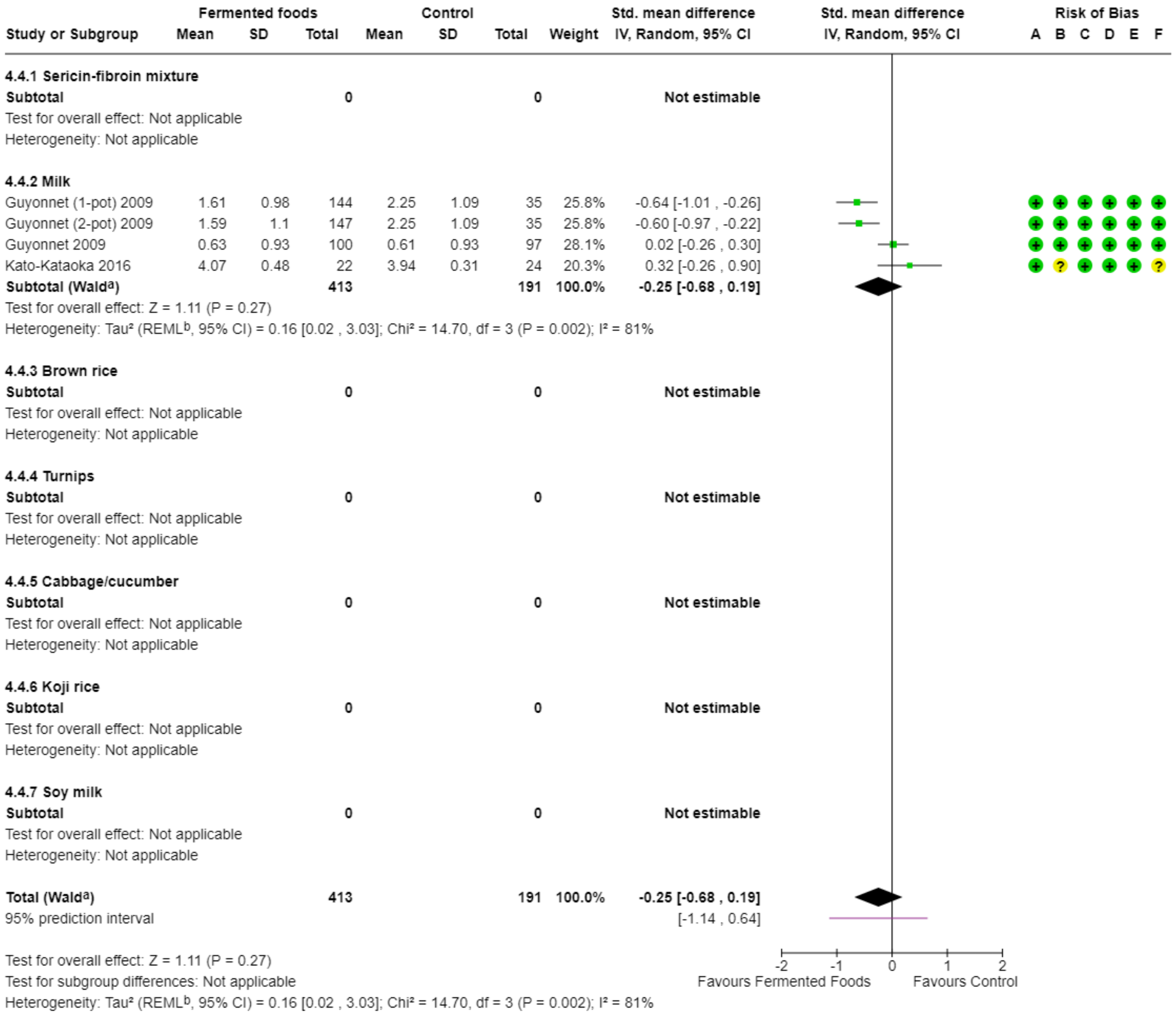

Supplementary Figure S10A. Subgroup analysis based on the fermentation matrix for abdominal pain. Forest plot of subgroup analysis based on the fermentation matrix for abdominal pain in randomised controlled trials comparing fermented foods with control in healthy adults (n=604). Values were calculated as standardised mean difference (95% CIs) using a random-effects model. CI, confidence interval; IV, inverse variance; SD, standard deviation; [a]CI calculated by Wald-type method; [b]Tau^2 calculated using Restricted Maximum-Likelihood method; Risk of bias legend: (A) bias arising from the randomisation process, (B) bias due to deviations from intended interventions, (C) bias due to missing outcome data, (D) bias in the measurement of the outcome, (E) bias in the selection of the reported result, (F) overall bias.

Study or Subgroup	Fermented foods Mean	SD	Total	Control Mean	SD	Total	Weight	Std. mean difference IV, Random, 95% CI	Risk of Bias A	B	C	D	E	F
**4.1.1 8 weeks**														
Kato-Kataoka 2016	4.07	0.48	22	3.94	0.31	24	20.3%	0.32 [-0.26 , 0.90]	+	?	+	+	+	?
**Subtotal**			**22**			**24**	**20.3%**	**0.32 [-0.26 , 0.90]**						
Test for overall effect: Z = 1.07 (P = 0.28)														
Heterogeneity: Not applicable														
**4.1.2 4 weeks**														
Guyonnet 2009	0.63	0.93	100	0.61	0.93	97	28.1%	0.02 [-0.26 , 0.30]	+	+	+	+	+	+
**Subtotal**			**100**			**97**	**28.1%**	**0.02 [-0.26 , 0.30]**						
Test for overall effect: Z = 0.15 (P = 0.88)														
Heterogeneity: Not applicable														
**4.1.3 16 weeks**														
**Subtotal**			**0**			**0**		**Not estimable**						
Test for overall effect: Not applicable														
Heterogeneity: Not applicable														
**4.1.4 6 weeks**														
**Subtotal**			**0**			**0**		**Not estimable**						
Test for overall effect: Not applicable														
Heterogeneity: Not applicable														
**4.1.5 2 weeks**														
Guyonnet (1-pot) 2009	1.61	0.98	144	2.25	1.09	35	25.8%	-0.64 [-1.01 , -0.26]	+	+	+	+	+	+
Guyonnet (2-pot) 2009	1.59	1.1	147	2.25	1.09	35	25.8%	-0.60 [-0.97 , -0.22]	+	+	+	+	+	+
**Subtotal (Wald[a])**			**291**			**70**	**51.5%**	**-0.62 [-0.88 , -0.35]**						
Test for overall effect: Z = 4.57 (P < 0.00001)														
Heterogeneity: Tau² (REML[b], 95% CI) = 0.00 [0.00 , 0.68]; Chi² = 0.02, df = 1 (P = 0.89); I² = 0%														
**4.1.6 3 weeks**														
**Subtotal**			**0**			**0**		**Not estimable**						
Test for overall effect: Not applicable														
Heterogeneity: Not applicable														
**4.1.7 12 weeks**														
**Subtotal**			**0**			**0**		**Not estimable**						
Test for overall effect: Not applicable														
Heterogeneity: Not applicable														
**4.1.8 24 weeks**														
**Subtotal**			**0**			**0**		**Not estimable**						
Test for overall effect: Not applicable														
Heterogeneity: Not applicable														
**Total (Wald[a])**			**413**			**191**	**100.0%**	**-0.25 [-0.68 , 0.19]**						
95% prediction interval								[-1.14 , 0.64]						

Test for overall effect: Z = 1.11 (P = 0.27)
Test for subgroup differences: Chi² = 14.68, df = 2 (P = 0.0007), I² = 86.4%
Heterogeneity: Tau² (REML[b], 95% CI) = 0.16 [0.02 , 3.03]; Chi² = 14.70, df = 3 (P = 0.002); I² = 81%

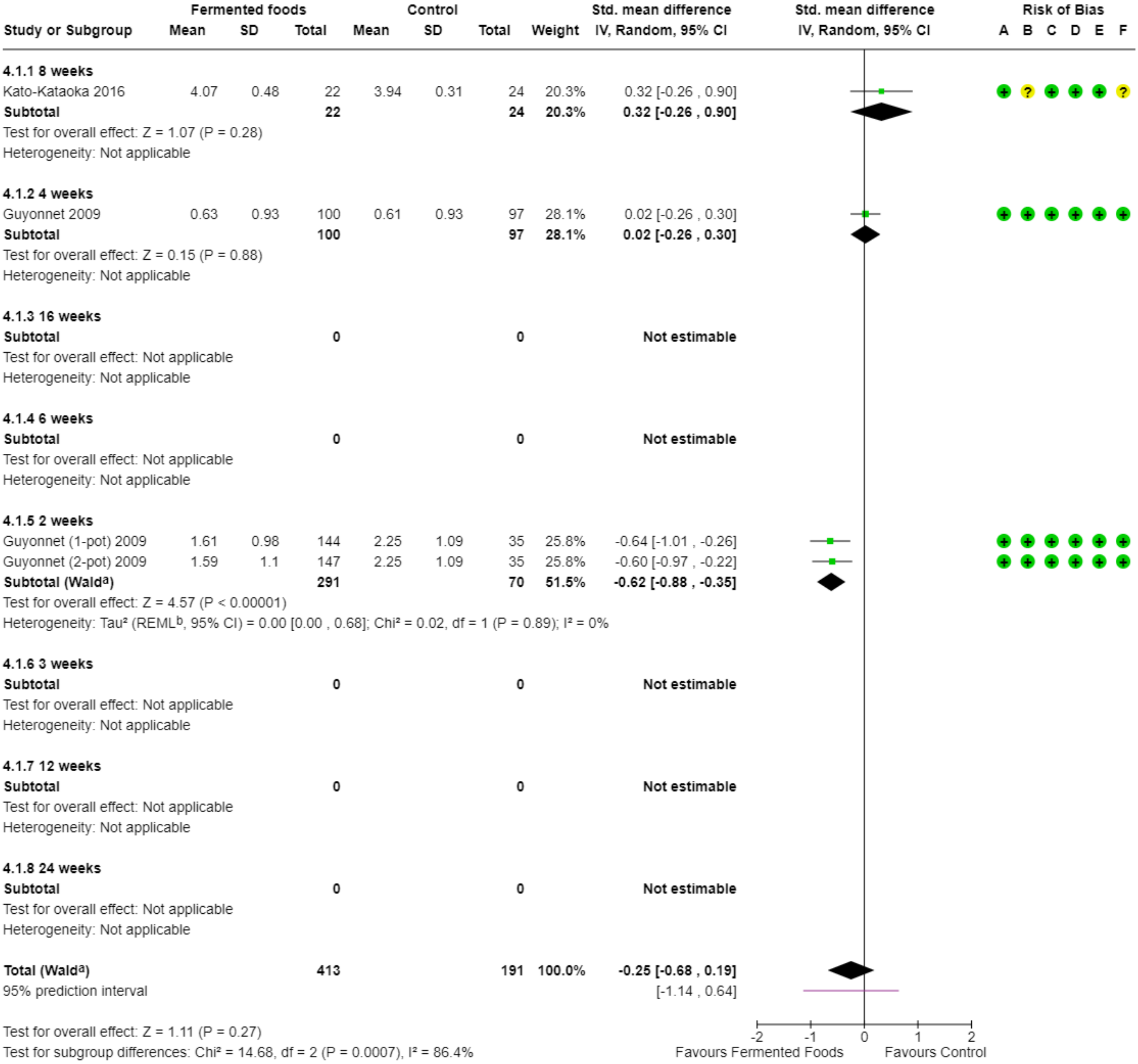

Supplementary Figure S10B. Subgroup analysis based on the intervention duration for abdominal pain. Forest plot of subgroup analysis based on the intervention duration for abdominal pain in randomised controlled trials comparing fermented foods with control in healthy adults (n=604). Values were calculated as standardised mean difference (95% CIs) using a random-effects model. CI, confidence interval; IV, inverse variance; SD, standard deviation; [a]CI calculated by Wald-type method; [b]Tau^2 calculated using Restricted Maximum-Likelihood method; Risk of bias legend: (A) bias arising from the randomisation process, (B) bias due to deviations from intended interventions, (C) bias due to missing outcome data, (D) bias in the measurement of the outcome, (E) bias in the selection of the reported result, (F) overall bias.

Study or Subgroup	Fermented foods Mean	SD	Total	Control Mean	SD	Total	Weight	Std. mean difference IV, Random, 95% CI	Risk of Bias A	B	C	D	E	F
**4.3.1 < 10^10 CFU/day**														
**Subtotal**			**0**			**0**		**Not estimable**						
Test for overall effect: Not applicable														
Heterogeneity: Not applicable														
**4.3.2 >= 10^10 CFU/day**														
Guyonnet (1-pot) 2009	1.61	0.98	144	2.25	1.09	35	25.8%	-0.64 [-1.01 , -0.26]	+	+	+	+	+	+
Guyonnet (2-pot) 2009	1.59	1.1	147	2.25	1.09	35	25.8%	-0.60 [-0.97 , -0.22]	+	+	+	+	+	+
Guyonnet 2009	0.63	0.93	100	0.61	0.93	97	28.1%	0.02 [-0.26 , 0.30]	+	+	+	+	+	+
Kato-Kataoka 2016	4.07	0.48	22	3.94	0.31	24	20.3%	0.32 [-0.26 , 0.90]	+	?	+	+	+	?
**Subtotal (Wald[a])**			**413**			**191**	**100.0%**	**-0.25 [-0.68 , 0.19]**						
Test for overall effect: Z = 1.11 (P = 0.27)														
Heterogeneity: Tau² (REML[b], 95% CI) = 0.16 [0.02 , 3.03]; Chi² = 14.70, df = 3 (P = 0.002); I² = 81%														
**Total (Wald[a])**			**413**			**191**	**100.0%**	**-0.25 [-0.68 , 0.19]**						
95% prediction interval								[-1.14 , 0.64]						

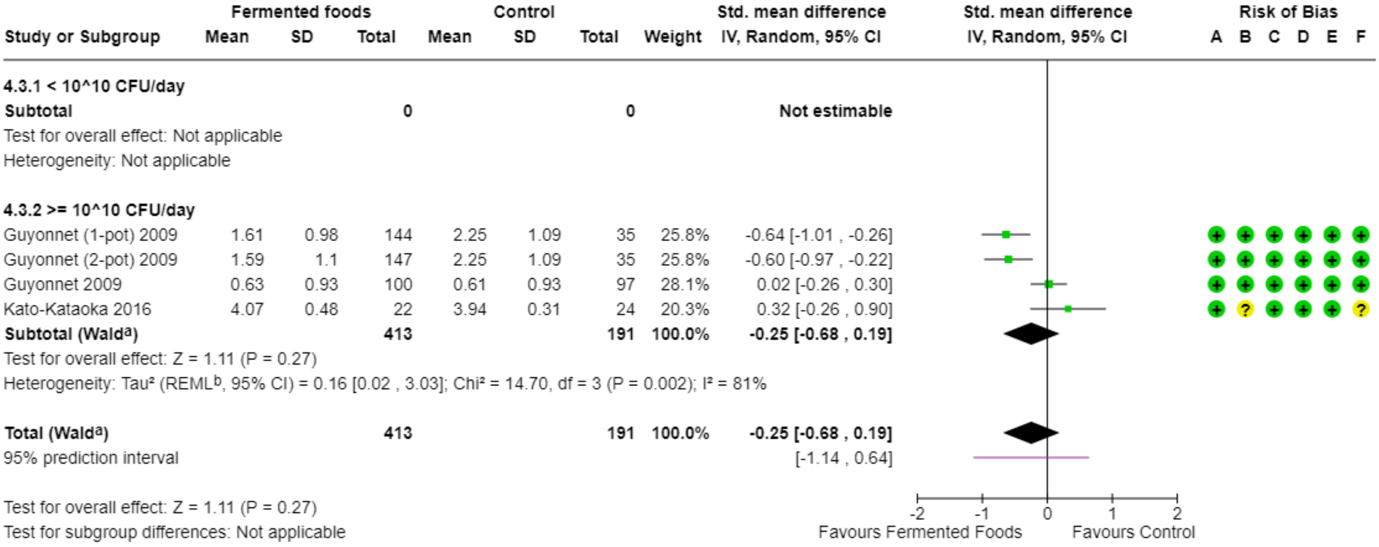

Test for overall effect: Z = 1.11 (P = 0.27)
Test for subgroup differences: Not applicable
Heterogeneity: Tau² (REML[b], 95% CI) = 0.16 [0.02 , 3.03]; Chi² = 14.70, df = 3 (P = 0.002); I² = 81%

Supplementary Figure S10C. Subgroup analysis based on the microbial dosage for abdominal pain. Forest plot of subgroup analysis based on the microbial dosage for abdominal pain in randomised controlled trials comparing fermented foods with control in healthy adults (n=604). Values were calculated as standardised mean difference (95% CIs) using a random-effects model. CI, confidence interval; IV, inverse variance; SD, standard deviation; [a]CI calculated by Wald-type method; [b]Tau^2 calculated using Restricted Maximum-Likelihood method; Risk of bias legend: (A) bias arising from the randomisation process, (B) bias due to deviations from intended interventions, (C) bias due to missing outcome data, (D) bias in the measurement of the outcome, (E) bias in the selection of the reported result, (F) overall bias.

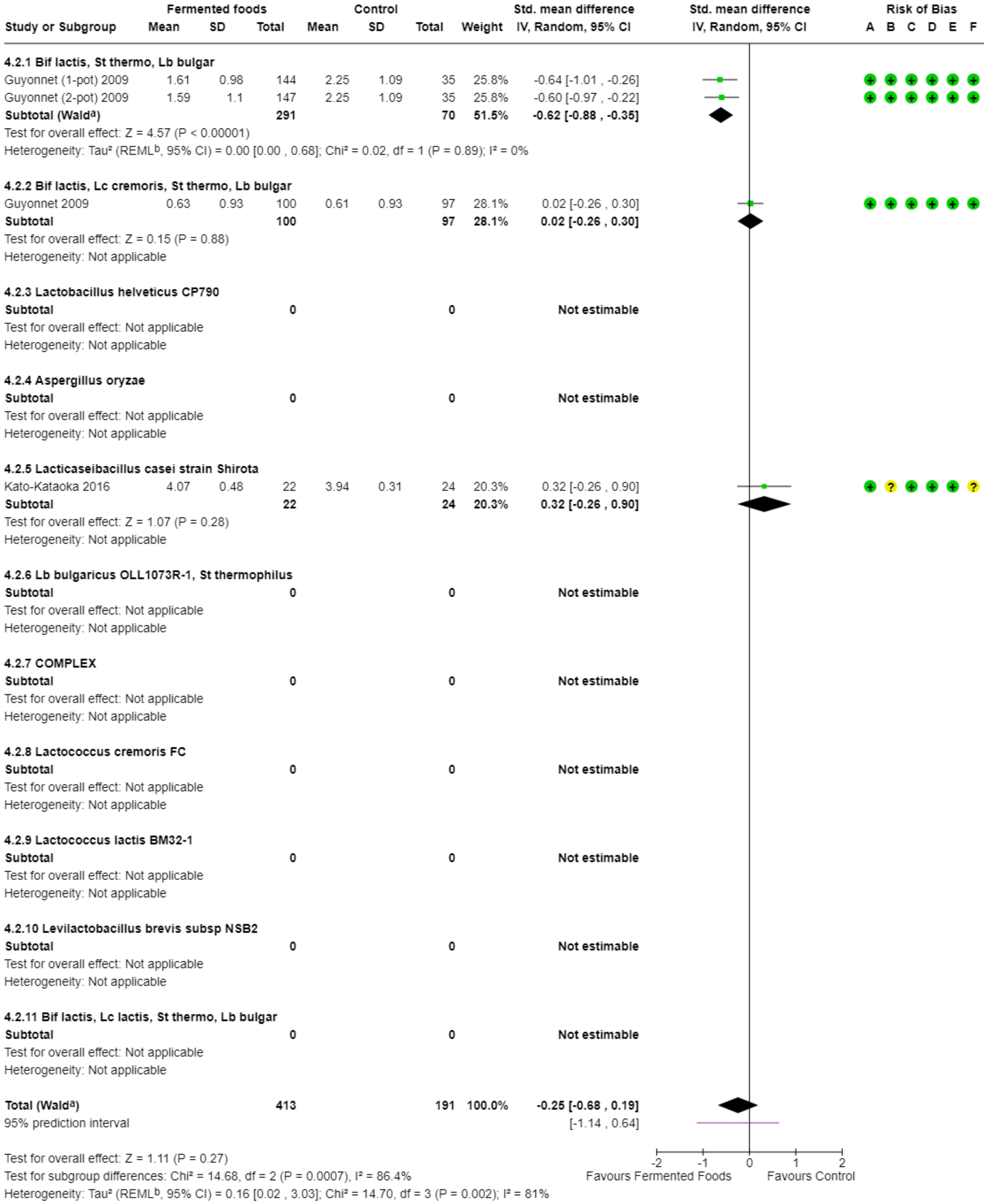

Supplementary Figure S10D. Subgroup analysis based on the fermentation microorganisms for abdominal pain. Forest plot of subgroup analysis based on the fermentation microorganisms for abdominal pain in randomised controlled trials comparing fermented foods with control in healthy adults (n=604). Values were calculated as standardised mean difference (95% CIs) using a random-effects model. COMPLEX, complex fermenting microbial community of several microorganisms consisting primarily of lactic acid bacteria; CI, confidence interval; IV, inverse

variance; SD, standard deviation; [a]CI calculated by Wald-type method; [b]Tau^2 calculated using Restricted Maximum-Likelihood method; Risk of bias legend: (A) bias arising from the randomisation process, (B) bias due to deviations from intended interventions, (C) bias due to missing outcome data, (D) bias in the measurement of the outcome, (E) bias in the selection of the reported result, (F) overall bias.

Study or Subgroup	Fermented foods Mean	SD	Total	Control Mean	SD	Total	Weight	Std. mean difference IV, Random, 95% CI
**4.9.1 Sericin-fibroin mixture**								
**Subtotal**			**0**			**0**		**Not estimable**
Test for overall effect: Not applicable								
Heterogeneity: Not applicable								
**4.9.2 Milk**								
Guyonnet (1-pot) 2009	1.66	0.68	144	2.8	1.07	35	25.6%	-1.47 [-1.87 , -1.07]
Guyonnet (2-pot) 2009	1.71	0.74	147	2.8	1.07	35	25.7%	-1.34 [-1.73 , -0.94]
Guyonnet 2009	1.26	0.997797575	100	1.45	0.971853898	97	26.7%	-0.19 [-0.47 , 0.09]
Kato-Kataoka 2016	0.64	0.18	22	0.98	0.17	24	22.0%	-1.91 [-2.62 , -1.20]
Koebnick 2003	1	0	35	1	0	35		Not estimable
**Subtotal (Wald[a])**			**448**			**226**	**100.0%**	**-1.19 [-1.92 , -0.47]**
Test for overall effect: Z = 3.22 (P = 0.001)								
Heterogeneity: Tau² (REML[b], 95% CI) = 0.49 [0.13 , 7.42]; Chi² = 44.92, df = 3 (P < 0.00001); I² = 92%								
**4.9.3 Brown rice**								
**Subtotal**			**0**			**0**		**Not estimable**
Test for overall effect: Not applicable								
Heterogeneity: Not applicable								
**4.9.4 Turnips**								
**Subtotal**			**0**			**0**		**Not estimable**
Test for overall effect: Not applicable								
Heterogeneity: Not applicable								
**4.9.5 Cabbage/cucumber**								
**Subtotal**			**0**			**0**		**Not estimable**
Test for overall effect: Not applicable								
Heterogeneity: Not applicable								
**4.9.6 Koji rice**								
**Subtotal**			**0**			**0**		**Not estimable**
Test for overall effect: Not applicable								
Heterogeneity: Not applicable								
**4.9.7 Soy milk**								
**Subtotal**			**0**			**0**		**Not estimable**
Test for overall effect: Not applicable								
Heterogeneity: Not applicable								
**Total (Wald[a])**			**448**			**226**	**100.0%**	**-1.19 [-1.92 , -0.47]**
95% prediction interval								[-2.75 , 0.36]
Test for overall effect: Z = 3.22 (P = 0.001)								
Test for subgroup differences: Not applicable								
Heterogeneity: Tau² (REML[b], 95% CI) = 0.49 [0.13 , 7.42]; Chi² = 44.92, df = 3 (P < 0.00001); I² = 92%								

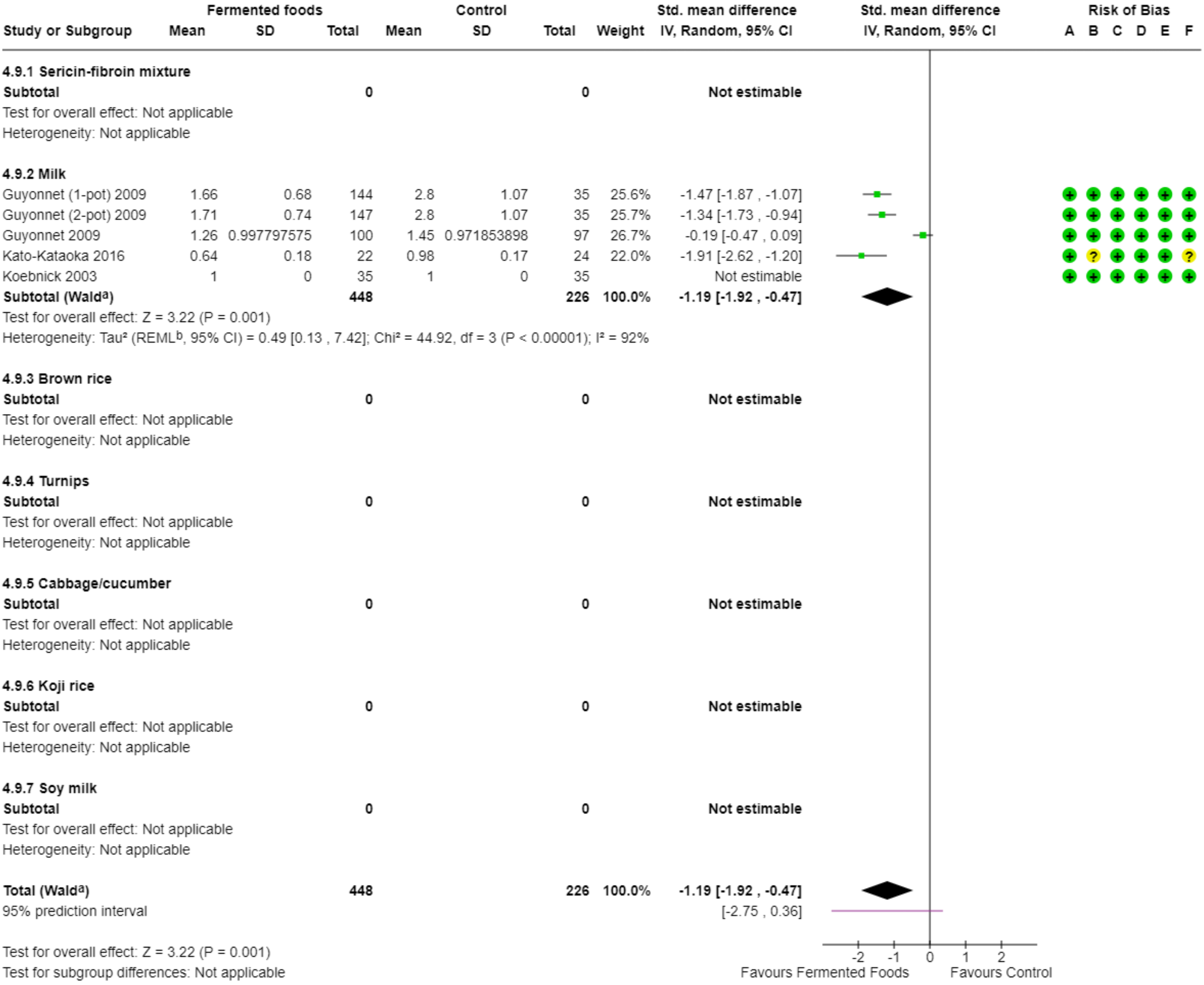

Supplementary Figure S11A. Subgroup analysis based on the fermentation matrix for bloating. Forest plot of subgroup analysis based on the fermentation matrix for bloating in randomised controlled trials comparing fermented foods with control in healthy adults (n=674). Values were calculated as standardised mean difference (95% CIs) using a random-effects model. CI, confidence interval; IV, inverse variance; SD, standard deviation; [a]CI calculated by Wald-type method; [b]Tau^2 calculated using Restricted Maximum-Likelihood method; Risk of bias legend: (A) bias arising from the randomisation process, (B) bias due to deviations from intended interventions, (C) bias due to missing outcome data, (D) bias in the measurement of the outcome, (E) bias in the selection of the reported result, (F) overall bias.

Study or Subgroup	Fermented foods Mean	SD	Total	Control Mean	SD	Total	Weight	Std. mean difference IV, Random, 95% CI	Risk of Bias A	B	C	D	E	F
**4.10.1 8 weeks**														
Kato-Kataoka 2016	0.64	0.18	22	0.98	0.17	24	22.0%	-1.91 [-2.62 , -1.20]	+	?	+	+	+	?
**Subtotal**			**22**			**24**	**22.0%**	**-1.91 [-2.62 , -1.20]**						
Test for overall effect: Z = 5.29 (P < 0.00001)														
Heterogeneity: Not applicable														
**4.10.2 4 weeks**														
Guyonnet 2009	1.26	0.997797575	100	1.45	0.971853898	97	26.7%	-0.19 [-0.47 , 0.09]	+	+	+	+	+	+
Koebnick 2003	1	0	35	1	0	35		Not estimable	+	+	+	+	+	+
**Subtotal**			**135**			**132**	**26.7%**	**-0.19 [-0.47 , 0.09]**						
Test for overall effect: Z = 1.35 (P = 0.18)														
Heterogeneity: Not applicable														
**4.10.3 16 weeks**														
**Subtotal**			**0**			**0**		**Not estimable**						
Test for overall effect: Not applicable														
Heterogeneity: Not applicable														
**4.10.4 6 weeks**														
**Subtotal**			**0**			**0**		**Not estimable**						
Test for overall effect: Not applicable														
Heterogeneity: Not applicable														
**4.10.5 2 weeks**														
Guyonnet (1-pot) 2009	1.66	0.68	144	2.8	1.07	35	25.6%	-1.47 [-1.87 , -1.07]	+	+	+	+	+	+
Guyonnet (2-pot) 2009	1.71	0.74	147	2.8	1.07	35	25.7%	-1.34 [-1.73 , -0.94]	+	+	+	+	+	+
**Subtotal (Wald[a])**			**291**			**70**	**51.3%**	**-1.40 [-1.68 , -1.12]**						
Test for overall effect: Z = 9.80 (P < 0.00001)														
Heterogeneity: Tau² (REML[b], 95% CI) = 0.00 [0.00 , 9.63]; Chi² = 0.23, df = 1 (P = 0.63); I² = 0%														
**4.10.6 3 weeks**														
**Subtotal**			**0**			**0**		**Not estimable**						
Test for overall effect: Not applicable														
Heterogeneity: Not applicable														
**4.10.7 12 weeks**														
**Subtotal**			**0**			**0**		**Not estimable**						
Test for overall effect: Not applicable														
Heterogeneity: Not applicable														
**4.10.8 24 weeks**														
**Subtotal**			**0**			**0**		**Not estimable**						
Test for overall effect: Not applicable														
Heterogeneity: Not applicable														
**Total (Wald[a])**			**448**			**226**	**100.0%**	**-1.19 [-1.92 , -0.47]**						
95% prediction interval								[-2.75 , 0.36]						

Test for overall effect: Z = 3.22 (P = 0.001)
Test for subgroup differences: Chi² = 44.69, df = 2 (P < 0.00001), I² = 95.5%
Heterogeneity: Tau² (REML[b], 95% CI) = 0.49 [0.13 , 7.42]; Chi² = 44.92, df = 3 (P < 0.00001); I² = 92%

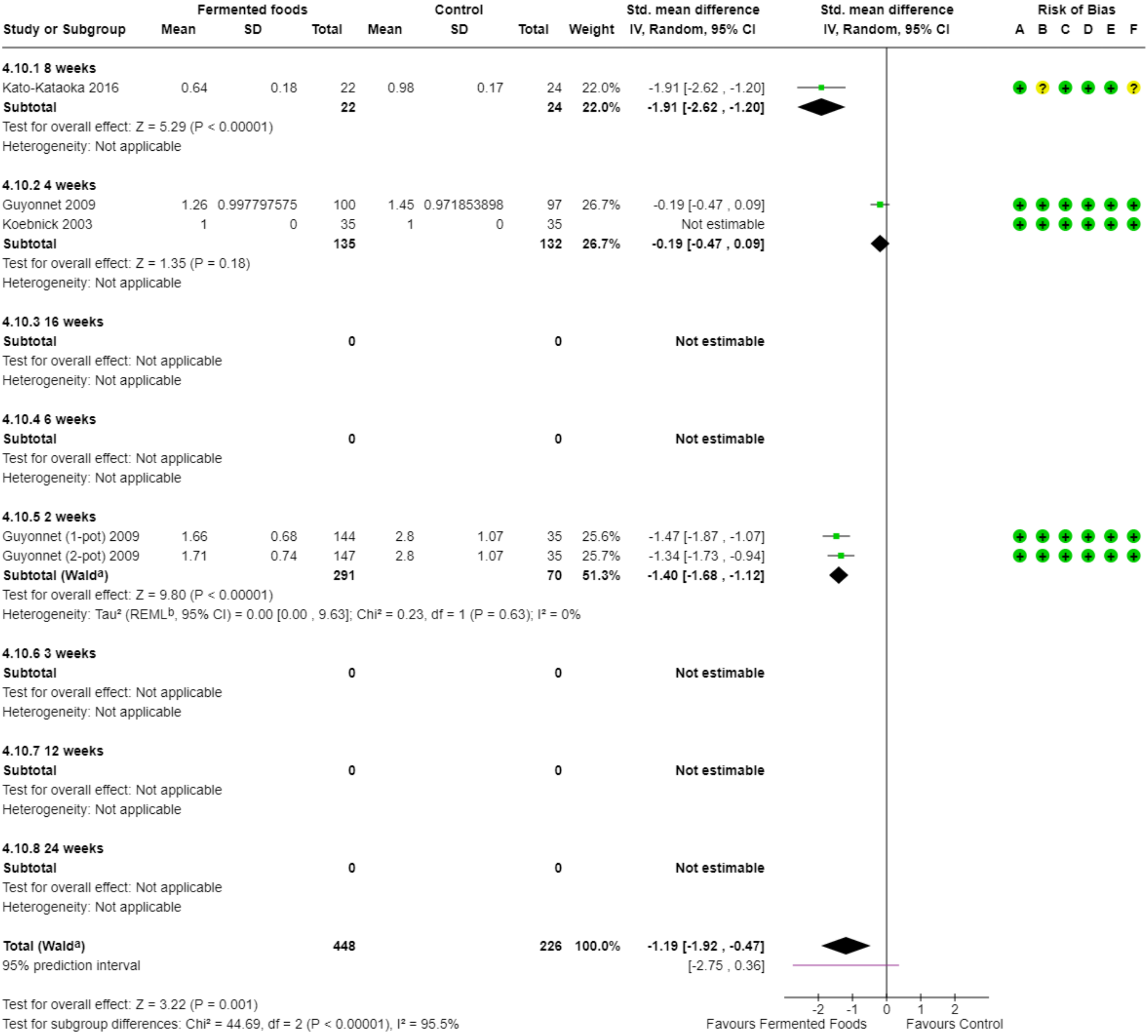

Supplementary Figure S11B. Subgroup analysis based on the intervention duration for bloating. Forest plot of subgroup analysis based on the intervention duration for bloating in randomised controlled trials comparing fermented foods with control in healthy adults (n=674). Values were calculated as standardised mean difference (95% CIs) using a random-effects model. CI, confidence interval; IV, inverse variance; SD, standard deviation; [a]CI calculated by Wald-type method; [b]Tau^2 calculated using Restricted Maximum-Likelihood method; Risk of bias legend: (A) bias arising from the randomisation process, (B) bias due to deviations from intended interventions, (C) bias due to missing outcome data, (D) bias in the measurement of the outcome, (E) bias in the selection of the reported result, (F) overall bias.

Study or Subgroup	Fermented foods Mean	Fermented foods SD	Fermented foods Total	Control Mean	Control SD	Control Total	Weight	Std. mean difference IV, Random, 95% CI	Risk of Bias A	B	C	D	E	F
**4.11.1 < 10^10 CFU/day**														
Koebnick 2003	1	0	35	1	0	35		Not estimable	+	+	+	+	+	+
**Subtotal**			**35**			**35**		**Not estimable**						
Test for overall effect: Not applicable														
Heterogeneity: Not applicable														
**4.11.2 >= 10^10 CFU/day**														
Guyonnet (1-pot) 2009	1.66	0.68	144	2.8	1.07	35	25.6%	-1.47 [-1.87 , -1.07]	+	+	+	+	+	+
Guyonnet (2-pot) 2009	1.71	0.74	147	2.8	1.07	35	25.7%	-1.34 [-1.73 , -0.94]	+	+	+	+	+	+
Guyonnet 2009	1.26	0.997797575	100	1.45	0.971853898	97	26.7%	-0.19 [-0.47 , 0.09]	+	+	+	+	+	+
Kato-Kataoka 2016	0.64	0.18	22	0.98	0.17	24	22.0%	-1.91 [-2.62 , -1.20]	+	?	+	+	+	?
**Subtotal (Wald[a])**			**413**			**191**	**100.0%**	**-1.19 [-1.92 , -0.47]**						
Test for overall effect: Z = 3.22 (P = 0.001)														
Heterogeneity: Tau² (REML[b], 95% CI) = 0.49 [0.13 , 7.42]; Chi² = 44.92, df = 3 (P < 0.00001); I² = 92%														
**Total (Wald[a])**			**448**			**226**	**100.0%**	**-1.19 [-1.92 , -0.47]**						
95% prediction interval								[-2.75 , 0.36]						

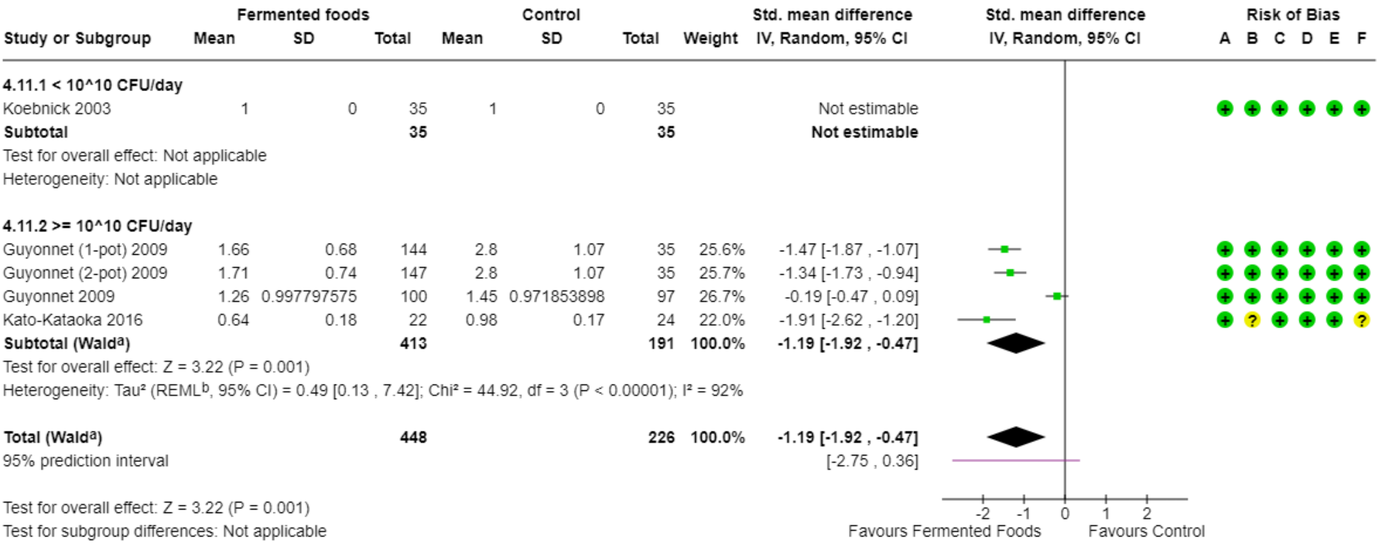

Test for overall effect: Z = 3.22 (P = 0.001)
Test for subgroup differences: Not applicable
Heterogeneity: Tau² (REML[b], 95% CI) = 0.49 [0.13 , 7.42]; Chi² = 44.92, df = 3 (P < 0.00001); I² = 92%

Supplementary Figure S11C. Subgroup analysis based on the microbial dosage for bloating. Forest plot of subgroup analysis based on the microbial dosage for bloating in randomised controlled trials comparing fermented foods with control in healthy adults (n=674). Values were calculated as standardised mean difference (95% CIs) using a random-effects model. CI, confidence interval; IV, inverse variance; SD, standard deviation; [a]CI calculated by Wald-type method; [b]Tau^2 calculated using Restricted Maximum-Likelihood method; Risk of bias legend: (A) bias arising from the randomisation process, (B) bias due to deviations from intended interventions, (C) bias due to missing outcome data, (D) bias in the measurement of the outcome, (E) bias in the selection of the reported result, (F) overall bias.

Study or Subgroup	Fermented foods Mean	Fermented foods SD	Fermented foods Total	Control Mean	Control SD	Control Total	Weight	Std. mean difference IV, Random, 95% CI
**4.12.1 Bif lactis, St thermo, Lb bulgar**								
Guyonnet (1-pot) 2009	1.66	0.68	144	2.8	1.07	35	25.6%	-1.47 [-1.87 , -1.07]
Guyonnet (2-pot) 2009	1.71	0.74	147	2.8	1.07	35	25.7%	-1.34 [-1.73 , -0.94]
**Subtotal (Wald[a])**			**291**			**70**	**51.3%**	**-1.40 [-1.68 , -1.12]**
Test for overall effect: Z = 9.80 (P < 0.00001)								
Heterogeneity: Tau² (REML[b], 95% CI) = 0.00 [0.00 , 9.63]; Chi² = 0.23, df = 1 (P = 0.63); I² = 0%								
**4.12.2 Bif lactis, Lc cremoris, St thermo, Lb bulgar**								
Guyonnet 2009	1.26	0.997797575	100	1.45	0.971853898	97	26.7%	-0.19 [-0.47 , 0.09]
**Subtotal**			**100**			**97**	**26.7%**	**-0.19 [-0.47 , 0.09]**
Test for overall effect: Z = 1.35 (P = 0.18)								
Heterogeneity: Not applicable								
**4.12.3 Lactobacillus helveticus CP790**								
**Subtotal**			**0**			**0**		**Not estimable**
Test for overall effect: Not applicable								
Heterogeneity: Not applicable								
**4.12.4 Aspergillus oryzae**								
**Subtotal**			**0**			**0**		**Not estimable**
Test for overall effect: Not applicable								
Heterogeneity: Not applicable								
**4.12.5 Lacticaseibacillus casei strain Shirota**								
Kato-Kataoka 2016	0.64	0.18	22	0.98	0.17	24	22.0%	-1.91 [-2.62 , -1.20]
Koebnick 2003	1	0	35	1	0	35		Not estimable
**Subtotal**			**57**			**59**	**22.0%**	**-1.91 [-2.62 , -1.20]**
Test for overall effect: Z = 5.29 (P < 0.00001)								
Heterogeneity: Not applicable								
**4.12.6 Lb bulgaricus OLL1073R-1, St thermophilus**								
**Subtotal**			**0**			**0**		**Not estimable**
Test for overall effect: Not applicable								
Heterogeneity: Not applicable								
**4.12.7 COMPLEX**								
**Subtotal**			**0**			**0**		**Not estimable**
Test for overall effect: Not applicable								
Heterogeneity: Not applicable								
**4.12.8 Lactococcus cremoris FC**								
**Subtotal**			**0**			**0**		**Not estimable**
Test for overall effect: Not applicable								
Heterogeneity: Not applicable								
**4.12.9 Lactococcus lactis BM32-1**								
**Subtotal**			**0**			**0**		**Not estimable**
Test for overall effect: Not applicable								
Heterogeneity: Not applicable								
**4.12.10 Levilactobacillus brevis subsp NSB2**								
**Subtotal**			**0**			**0**		**Not estimable**
Test for overall effect: Not applicable								
Heterogeneity: Not applicable								
**4.12.11 Bif lactis, Lc lactis, St thermo, Lb bulgar**								
**Subtotal**			**0**			**0**		**Not estimable**
Test for overall effect: Not applicable								
Heterogeneity: Not applicable								
**Total (Wald[a])**			**448**			**226**	**100.0%**	**-1.19 [-1.92 , -0.47]**
95% prediction interval								[-2.75 , 0.36]

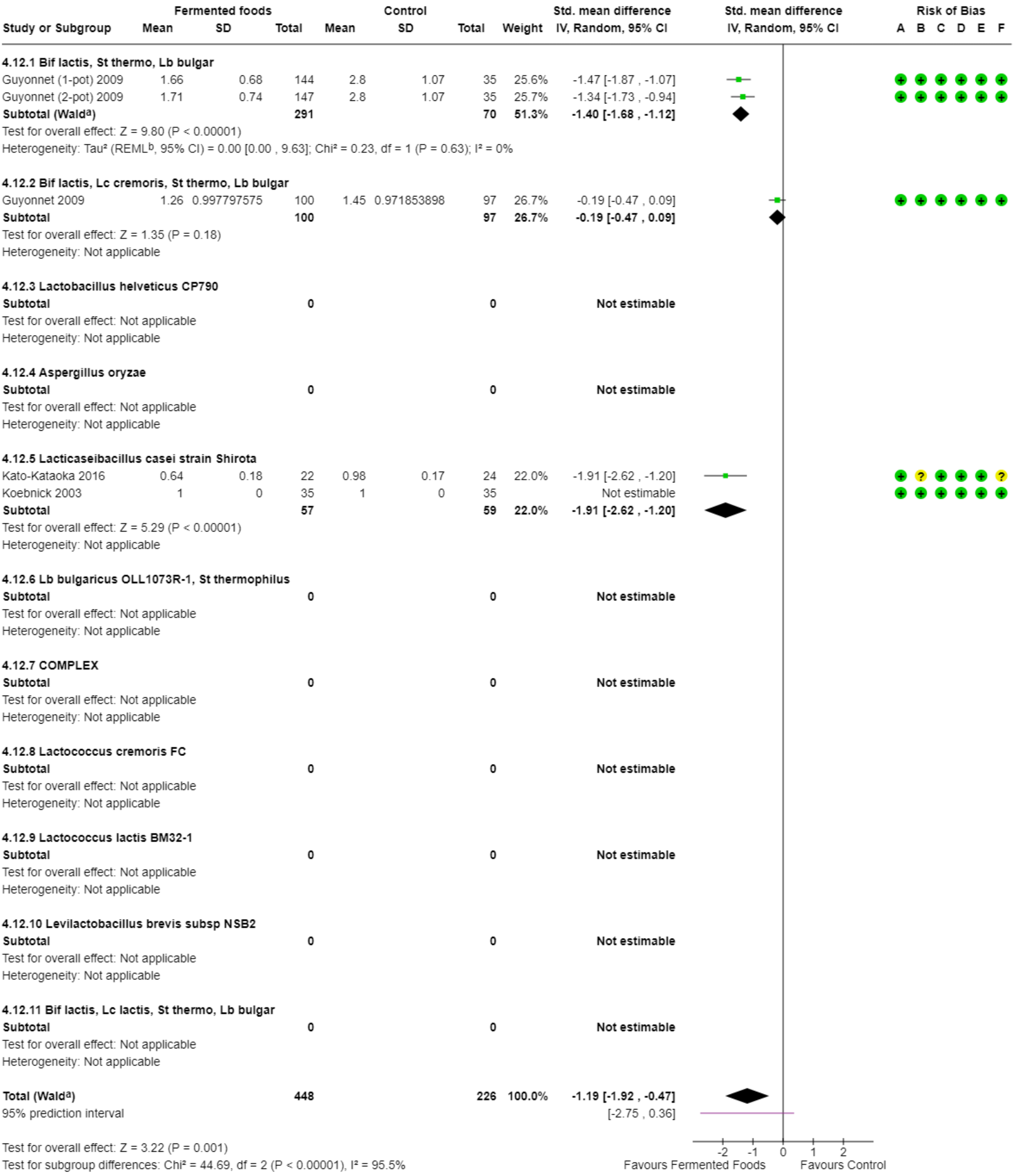

Test for overall effect: Z = 3.22 (P = 0.001)
Test for subgroup differences: Chi² = 44.69, df = 2 (P < 0.00001), I² = 95.5%
Heterogeneity: Tau² (REML[b], 95% CI) = 0.49 [0.13 , 7.42]; Chi² = 44.92, df = 3 (P < 0.00001); I² = 92%

Supplementary Figure S11D. Subgroup analysis based on the fermentation microorganisms for bloating. Forest plot of subgroup analysis based on the fermentation microorganisms for bloating in randomised controlled trials comparing fermented foods with control in healthy adults (n=674). Values were calculated as standardised mean difference (95% CIs) using a random-effects model. COMPLEX, complex fermenting microbial community of several microorganisms consisting primarily of lactic acid bacteria; CI, confidence interval; IV, inverse variance; SD, standard deviation; [a]CI calculated by Wald-type method; [b]Tau^2 calculated using Restricted Maximum-Likelihood method; Risk of bias legend:

(A) bias arising from the randomisation process, (B) bias due to deviations from intended interventions, (C) bias due to missing outcome data, (D) bias in the measurement of the outcome, (E) bias in the selection of the reported result, (F) overall bias.

Study or Subgroup	Fermented foods Mean	Fermented foods SD	Fermented foods Total	Control Mean	Control SD	Control Total	Weight	Std. mean difference IV, Random, 95% CI	Risk of Bias A	B	C	D	E	F
**4.13.1 Sericin-fibroin mixture**														
**Subtotal**			**0**			**0**		**Not estimable**						
Test for overall effect: Not applicable														
Heterogeneity: Not applicable														
**4.13.2 Milk**														
Guyonnet (1-pot) 2009	1.6	0.69	144	2.28	0.89	35	30.9%	-0.92 [-1.31 , -0.54]	+	+	+	+	+	+
Guyonnet (2-pot) 2009	1.71	0.81	147	2.28	0.89	35	31.4%	-0.69 [-1.06 , -0.31]	+	+	+	+	+	+
Guyonnet 2009	0.9	0.97	100	1.24	0.97	97	37.7%	-0.35 [-0.63 , -0.07]	+	+	+	+	+	+
**Subtotal (Wald[a])**			**391**			**167**	**100.0%**	**-0.63 [-0.97 , -0.29]**						
Test for overall effect: Z = 3.66 (P = 0.0003)														
Heterogeneity: Tau^2 (REML[b], 95% CI) = 0.06 [0.00 , 3.27]; Chi^2 = 6.01, df = 2 (P = 0.05); I^2 = 66%														
**4.13.3 Brown rice**														
**Subtotal**			**0**			**0**		**Not estimable**						
Test for overall effect: Not applicable														
Heterogeneity: Not applicable														
**4.13.4 Turnips**														
**Subtotal**			**0**			**0**		**Not estimable**						
Test for overall effect: Not applicable														
Heterogeneity: Not applicable														
**4.13.5 Cabbage/cucumber**														
**Subtotal**			**0**			**0**		**Not estimable**						
Test for overall effect: Not applicable														
Heterogeneity: Not applicable														
**4.13.6 Koji rice**														
**Subtotal**			**0**			**0**		**Not estimable**						
Test for overall effect: Not applicable														
Heterogeneity: Not applicable														
**4.13.7 Soy milk**														
**Subtotal**			**0**			**0**		**Not estimable**						
Test for overall effect: Not applicable														
Heterogeneity: Not applicable														
**Total (Wald[a])**			**391**			**167**	**100.0%**	**-0.63 [-0.97 , -0.29]**						
95% prediction interval								[-1.22 , -0.05]						

Test for overall effect: Z = 3.66 (P = 0.0003)
Test for subgroup differences: Not applicable
Heterogeneity: Tau^2 (REML[b], 95% CI) = 0.06 [0.00 , 3.27]; Chi^2 = 6.01, df = 2 (P = 0.05); I^2 = 66%

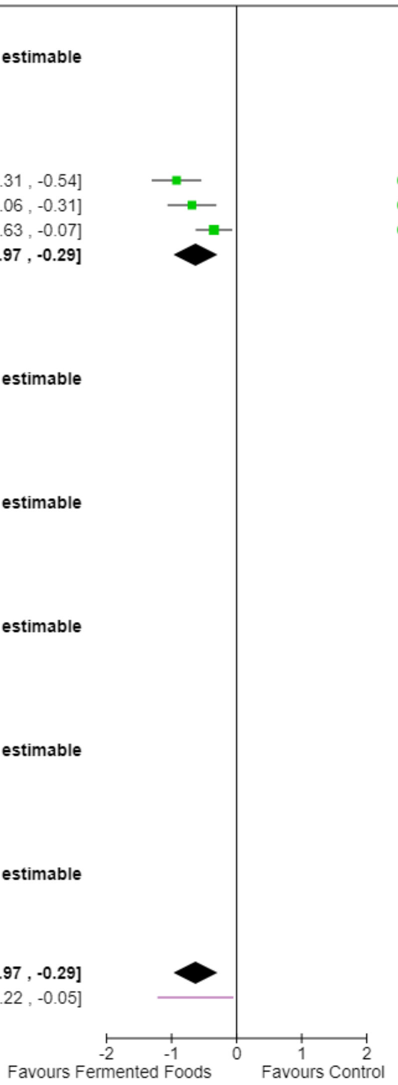

Supplementary Figure S12A. Subgroup analysis based on the fermentation matrix for borborygmi. Forest plot of subgroup analysis based on the fermentation matrix for borborygmi in randomised controlled trials comparing fermented foods with control in healthy adults (n=558). Values were calculated as standardised mean difference (95% CIs) using a random-effects model. CI, confidence interval; IV, inverse variance; SD, standard deviation; [a]CI calculated by Wald-type method; [b]Tau^2 calculated using Restricted Maximum-Likelihood method; Risk of bias legend: (A) bias arising from the randomisation process, (B) bias due to deviations from intended interventions, (C) bias due to missing outcome data, (D) bias in the measurement of the outcome, (E) bias in the selection of the reported result, (F) overall bias.

Study or Subgroup	Fermented foods Mean	Fermented foods SD	Fermented foods Total	Control Mean	Control SD	Control Total	Weight	Std. mean difference IV, Random, 95% CI	Risk of Bias A	B	C	D	E	F
**4.14.1 8 weeks**														
**Subtotal**			**0**			**0**		**Not estimable**						
Test for overall effect: Not applicable														
Heterogeneity: Not applicable														
**4.14.2 4 weeks**														
Guyonnet 2009	0.9	0.97	100	1.24	0.97	97	37.7%	-0.35 [-0.63 , -0.07]	+	+	+	+	+	+
**Subtotal**			**100**			**97**	**37.7%**	**-0.35 [-0.63 , -0.07]**						
Test for overall effect: Z = 2.43 (P = 0.02)														
Heterogeneity: Not applicable														
**4.14.3 16 weeks**														
**Subtotal**			**0**			**0**		**Not estimable**						
Test for overall effect: Not applicable														
Heterogeneity: Not applicable														
**4.14.4 6 weeks**														
**Subtotal**			**0**			**0**		**Not estimable**						
Test for overall effect: Not applicable														
Heterogeneity: Not applicable														
**4.14.5 2 weeks**														
Guyonnet (1-pot) 2009	1.6	0.69	144	2.28	0.89	35	30.9%	-0.92 [-1.31 , -0.54]	+	+	+	+	+	+
Guyonnet (2-pot) 2009	1.71	0.81	147	2.28	0.89	35	31.4%	-0.69 [-1.06 , -0.31]	+	+	+	+	+	+
**Subtotal (Wald[a])**			**291**			**70**	**62.3%**	**-0.80 [-1.07 , -0.54]**						
Test for overall effect: Z = 5.88 (P < 0.00001)														
Heterogeneity: Tau² (REML[b], 95% CI) = 0.00 [0.00 , 28.50]; Chi² = 0.75, df = 1 (P = 0.39); I² = 0%														
**4.14.6 3 weeks**														
**Subtotal**			**0**			**0**		**Not estimable**						
Test for overall effect: Not applicable														
Heterogeneity: Not applicable														
**4.14.7 12 weeks**														
**Subtotal**			**0**			**0**		**Not estimable**						
Test for overall effect: Not applicable														
Heterogeneity: Not applicable														
**4.14.8 24 weeks**														
**Subtotal**			**0**			**0**		**Not estimable**						
Test for overall effect: Not applicable														
Heterogeneity: Not applicable														
**Total (Wald[a])**			**391**			**167**	**100.0%**	**-0.63 [-0.97 , -0.29]**						
95% prediction interval								[-1.22 , -0.05]						
Test for overall effect: Z = 3.66 (P = 0.0003)														
Test for subgroup differences: Chi² = 5.26, df = 1 (P = 0.02), I² = 81.0%														
Heterogeneity: Tau² (REML[b], 95% CI) = 0.06 [0.00 , 3.27]; Chi² = 6.01, df = 2 (P = 0.05); I² = 66%														

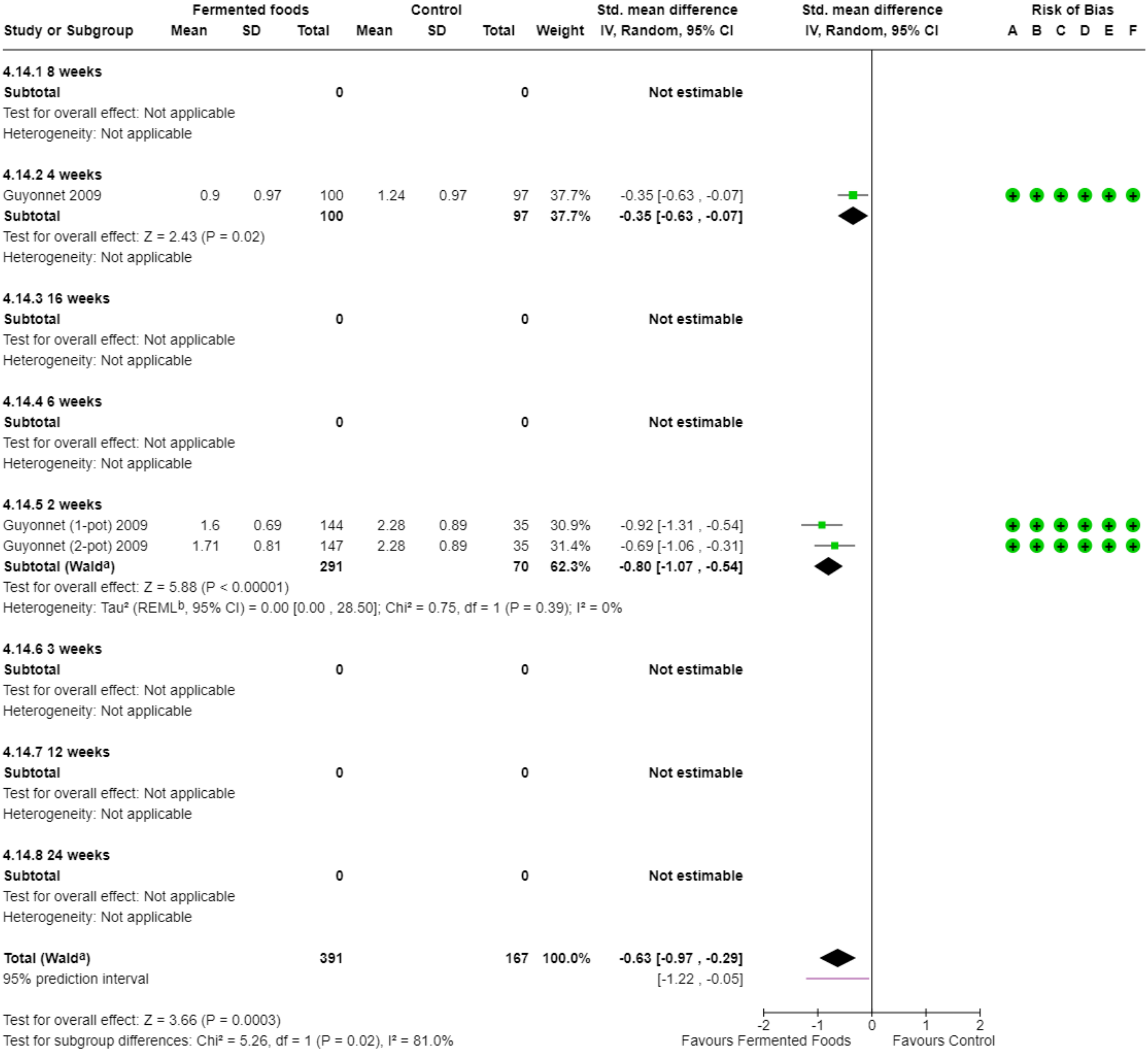

Supplementary Figure S12B. Subgroup analysis based on the intervention duration for borborygmi. Forest plot of subgroup analysis based on the intervention duration for borborygmi in randomised controlled trials comparing fermented foods with control in healthy adults (n=558). Values were calculated as standardised mean difference (95% CIs) using a random-effects model. CI, confidence interval; IV, inverse variance; SD, standard deviation; [a]CI calculated by Wald-type method; [b]Tau^2 calculated using Restricted Maximum-Likelihood method; Risk of bias legend: (A) bias arising from the randomisation process, (B) bias due to deviations from intended interventions, (C) bias due to missing outcome data, (D) bias in the measurement of the outcome, (E) bias in the selection of the reported result, (F) overall bias.

Study or Subgroup	Fermented foods Mean	SD	Total	Control Mean	SD	Total	Weight	Std. mean difference IV, Random, 95% CI
**4.15.1 < 10^10 CFU/day**								
**Subtotal**			**0**			**0**		**Not estimable**
Test for overall effect: Not applicable								
Heterogeneity: Not applicable								
**4.15.2 >= 10^10 CFU/day**								
Guyonnet (1-pot) 2009	1.6	0.69	144	2.28	0.89	35	30.9%	-0.92 [-1.31 , -0.54]
Guyonnet (2-pot) 2009	1.71	0.81	147	2.28	0.89	35	31.4%	-0.69 [-1.06 , -0.31]
Guyonnet 2009	0.9	0.97	100	1.24	0.97	97	37.7%	-0.35 [-0.63 , -0.07]
**Subtotal (Wald[a])**			**391**			**167**	**100.0%**	**-0.63 [-0.97 , -0.29]**
Test for overall effect: Z = 3.66 (P = 0.0003)								
Heterogeneity: Tau² (REML[b], 95% CI) = 0.06 [0.00 , 3.27]; Chi² = 6.01, df = 2 (P = 0.05); I² = 66%								
**Total (Wald[a])**			**391**			**167**	**100.0%**	**-0.63 [-0.97 , -0.29]**
95% prediction interval								[-1.22 , -0.05]
Test for overall effect: Z = 3.66 (P = 0.0003)								
Test for subgroup differences: Not applicable								
Heterogeneity: Tau² (REML[b], 95% CI) = 0.06 [0.00 , 3.27]; Chi² = 6.01, df = 2 (P = 0.05); I² = 66%								

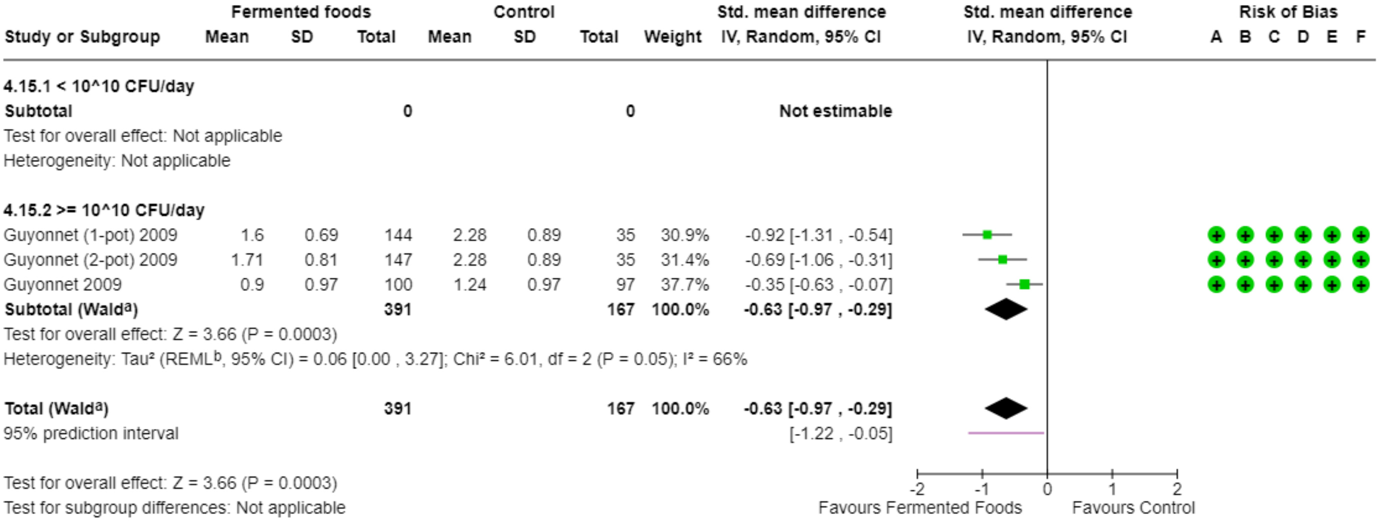

Supplementary Figure S12C. Subgroup analysis based on the microbial dosage for borborygmi. Forest plot of subgroup analysis based on the microbial dosage for borborygmi in randomised controlled trials comparing fermented foods with control in healthy adults (n=558). Values were calculated as standardised mean difference (95% CIs) using a random-effects model. CI, confidence interval; IV, inverse variance; SD, standard deviation; [a]CI calculated by Wald-type method; [b]Tau^2 calculated using Restricted Maximum-Likelihood method; Risk of bias legend: (A) bias arising from the randomisation process, (B) bias due to deviations from intended interventions, (C) bias due to missing outcome data, (D) bias in the measurement of the outcome, (E) bias in the selection of the reported result, (F) overall bias.

Study or Subgroup	Fermented foods Mean	Fermented foods SD	Fermented foods Total	Control Mean	Control SD	Control Total	Weight	Std. mean difference IV, Random, 95% CI	Risk of Bias A	B	C	D	E	F
**4.16.1 Bif lactis, St thermo, Lb bulgar**														
Guyonnet (1-pot) 2009	1.6	0.69	144	2.28	0.89	35	30.9%	-0.92 [-1.31 , -0.54]	+	+	+	+	+	+
Guyonnet (2-pot) 2009	1.71	0.81	147	2.28	0.89	35	31.4%	-0.69 [-1.06 , -0.31]	+	+	+	+	+	+
**Subtotal (Wald[a])**			**291**			**70**	**62.3%**	**-0.80 [-1.07 , -0.54]**						
Test for overall effect: Z = 5.88 (P < 0.00001)														
Heterogeneity: Tau^2 (REML[b], 95% CI) = 0.00 [0.00 , 28.50]; Chi^2 = 0.75, df = 1 (P = 0.39); I^2 = 0%														
**4.16.2 Bif lactis, Lc cremoris, St thermo, Lb bulgar**														
Guyonnet 2009	0.9	0.97	100	1.24	0.97	97	37.7%	-0.35 [-0.63 , -0.07]	+	+	+	+	+	+
**Subtotal**			**100**			**97**	**37.7%**	**-0.35 [-0.63 , -0.07]**						
Test for overall effect: Z = 2.43 (P = 0.02)														
Heterogeneity: Not applicable														
**4.16.3 Lactobacillus helveticus CP790**														
**Subtotal**			**0**			**0**		**Not estimable**						
Test for overall effect: Not applicable														
Heterogeneity: Not applicable														
**4.16.4 Aspergillus oryzae**														
**Subtotal**			**0**			**0**		**Not estimable**						
Test for overall effect: Not applicable														
Heterogeneity: Not applicable														
**4.16.5 Lacticaseibacillus casei strain Shirota**														
**Subtotal**			**0**			**0**		**Not estimable**						
Test for overall effect: Not applicable														
Heterogeneity: Not applicable														
**4.16.6 Lb bulgaricus OLL1073R-1, St thermophilus**														
**Subtotal**			**0**			**0**		**Not estimable**						
Test for overall effect: Not applicable														
Heterogeneity: Not applicable														
**4.16.7 COMPLEX**														
**Subtotal**			**0**			**0**		**Not estimable**						
Test for overall effect: Not applicable														
Heterogeneity: Not applicable														
**4.16.8 Lactococcus cremoris FC**														
**Subtotal**			**0**			**0**		**Not estimable**						
Test for overall effect: Not applicable														
Heterogeneity: Not applicable														
**4.16.9 Lactococcus lactis BM32-1**														
**Subtotal**			**0**			**0**		**Not estimable**						
Test for overall effect: Not applicable														
Heterogeneity: Not applicable														
**4.16.10 Levilactobacillus brevis subsp NSB2**														
**Subtotal**			**0**			**0**		**Not estimable**						
Test for overall effect: Not applicable														
Heterogeneity: Not applicable														
**4.16.11 Bif lactis, Lc lactis, St thermo, Lb bulgar**														
**Subtotal**			**0**			**0**		**Not estimable**						
Test for overall effect: Not applicable														
Heterogeneity: Not applicable														
**Total (Wald[a])**			**391**			**167**	**100.0%**	**-0.63 [-0.97 , -0.29]**						
95% prediction interval								[-1.22 , -0.05]						

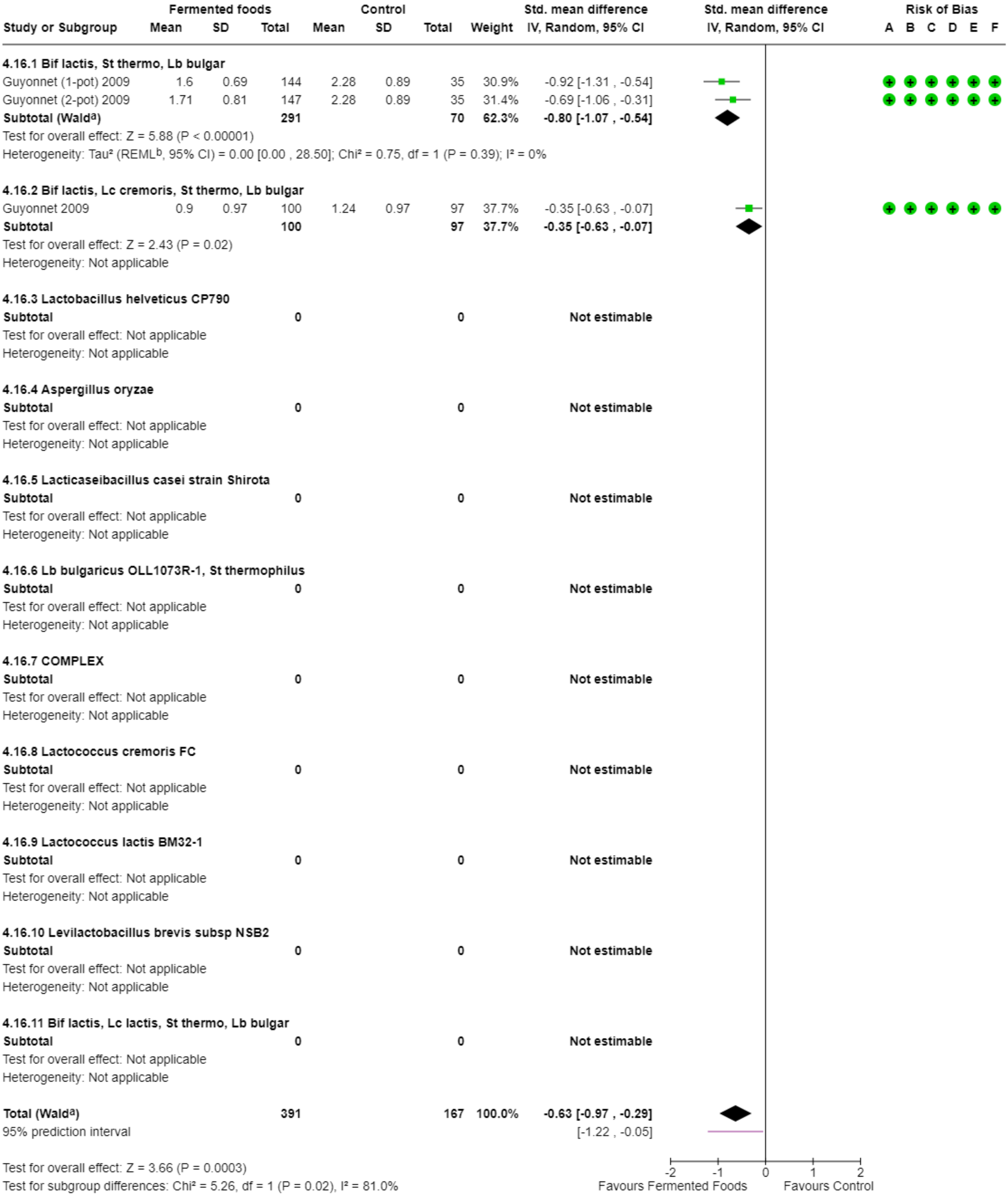

Test for overall effect: Z = 3.66 (P = 0.0003)
Test for subgroup differences: Chi^2 = 5.26, df = 1 (P = 0.02), I^2 = 81.0%
Heterogeneity: Tau^2 (REML[b], 95% CI) = 0.06 [0.00 , 3.27]; Chi^2 = 6.01, df = 2 (P = 0.05); I^2 = 66%

Supplementary Figure S12D. Subgroup analysis based on the fermentation microorganisms for borborygmi. Forest plot of subgroup analysis based on the fermentation microorganisms for borborygmi in randomised controlled trials comparing fermented foods with control in healthy adults (n=558). Values were calculated as standardised mean difference (95% CIs) using a random-effects model. COMPLEX, complex fermenting microbial community of several microorganisms consisting primarily of lactic acid bacteria; CI, confidence interval; IV, inverse variance; SD, standard

deviation; [a]CI calculated by Wald-type method; [b]Tau^2 calculated using Restricted Maximum-Likelihood method; Risk of bias legend: (A) bias arising from the randomisation process, (B) bias due to deviations from intended interventions, (C) bias due to missing outcome data, (D) bias in the measurement of the outcome, (E) bias in the selection of the reported result, (F) overall bias.

Study or Subgroup	Fermented foods Mean	SD	Total	Control Mean	SD	Total	Weight	Std. mean difference IV, Random, 95% CI	A	B	C	D	E	F
**4.17.1 Sericin-fibroin mixture**														
**Subtotal**			**0**			**0**		**Not estimable**						
Test for overall effect: Not applicable														
Heterogeneity: Not applicable														
**4.17.2 Milk**														
Guyonnet (1-pot) 2009	1.97	0.95	144	2.62	0.93	35	23.7%	-0.68 [-1.06 , -0.31]	+	+	+	+	+	+
Guyonnet (2-pot) 2009	2.24	1.14	147	2.62	0.93	35	24.3%	-0.34 [-0.71 , 0.03]	+	+	+	+	+	+
Guyonnet 2009	1.74	1.100227249	100	2.06	1.014642794	97	36.4%	-0.30 [-0.58 , -0.02]	+	+	+	+	+	+
Koebnick 2003	0.75	0.23	35	1	0.47	35	15.7%	-0.67 [-1.15 , -0.19]	+	+	+	+	+	+
**Subtotal (Wald[a])**			**426**			**202**	**100.0%**	**-0.46 [-0.67 , -0.25]**						
Test for overall effect: Z = 4.38 (P < 0.0001)														
Heterogeneity: Tau² (REML[b], 95% CI) = 0.01 [0.00 , 0.55]; Chi² = 3.67, df = 3 (P = 0.30); I² = 22%														
**4.17.3 Brown rice**														
**Subtotal**			**0**			**0**		**Not estimable**						
Test for overall effect: Not applicable														
Heterogeneity: Not applicable														
**4.17.4 Turnips**														
**Subtotal**			**0**			**0**		**Not estimable**						
Test for overall effect: Not applicable														
Heterogeneity: Not applicable														
**4.17.5 Cabbage/cucumber**														
**Subtotal**			**0**			**0**		**Not estimable**						
Test for overall effect: Not applicable														
Heterogeneity: Not applicable														
**4.17.6 Koji rice**														
**Subtotal**			**0**			**0**		**Not estimable**						
Test for overall effect: Not applicable														
Heterogeneity: Not applicable														
**4.17.7 Soy milk**														
**Subtotal**			**0**			**0**		**Not estimable**						
Test for overall effect: Not applicable														
Heterogeneity: Not applicable														
**Total (Wald[a])**			**426**			**202**	**100.0%**	**-0.46 [-0.67 , -0.25]**						
95% prediction interval								[-0.74 , -0.18]						

Test for overall effect: Z = 4.38 (P < 0.0001)
Test for subgroup differences: Not applicable
Heterogeneity: Tau² (REML[b], 95% CI) = 0.01 [0.00 , 0.55]; Chi² = 3.67, df = 3 (P = 0.30); I² = 22%

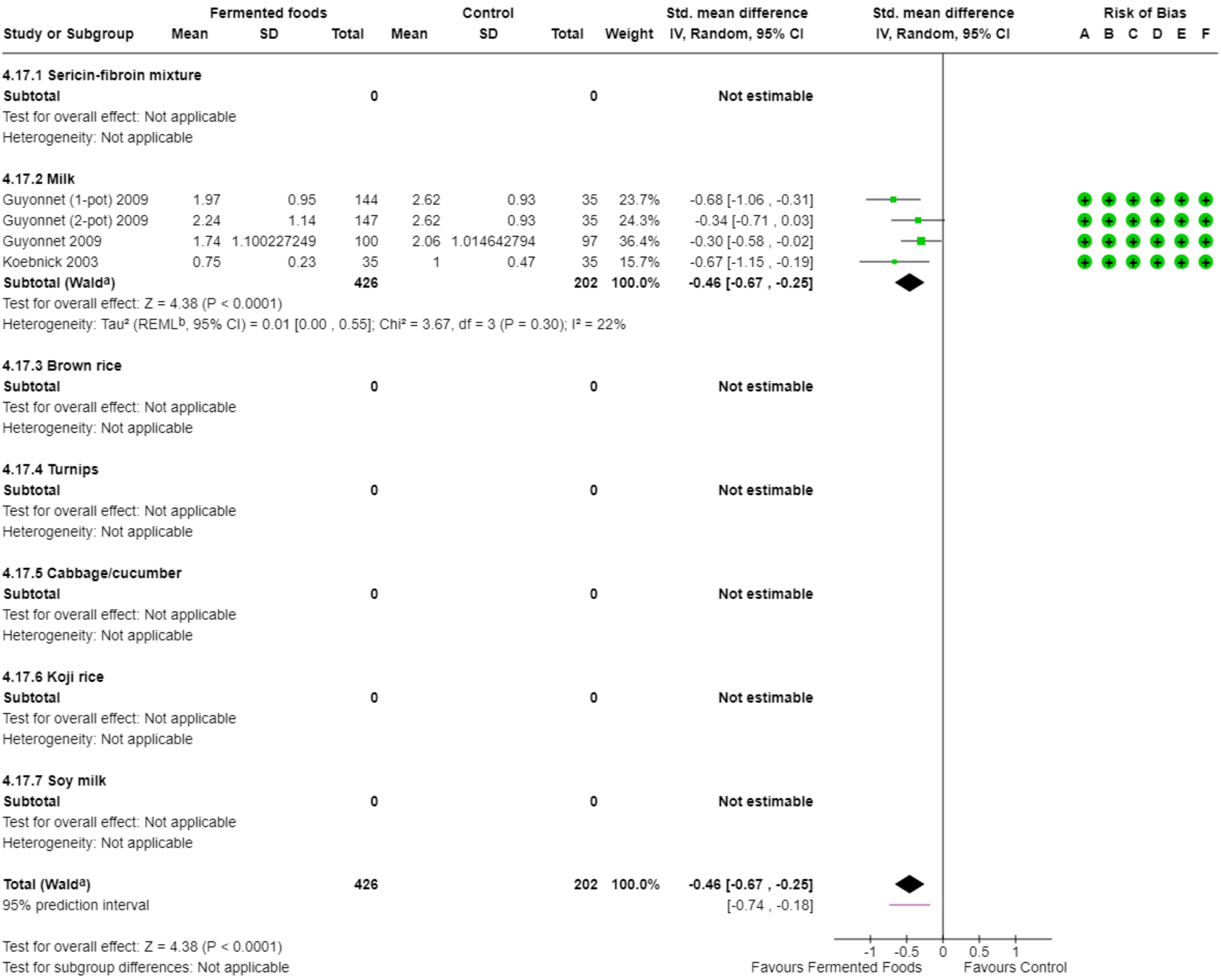

Supplementary Figure S13A. Subgroup analysis based on the fermentation matrix for flatulence. Forest plot of subgroup analysis based on the fermentation matrix for flatulence in randomised controlled trials comparing fermented foods with control in healthy adults (n=628). Values were calculated as standardised mean difference (95% CIs) using a random-effects model. CI, confidence interval; IV, inverse variance; SD, standard deviation; [a]CI calculated by Wald-type method; [b]Tau^2 calculated using Restricted Maximum-Likelihood method; Risk of bias legend: (A) bias arising from the randomisation process, (B) bias due to deviations from intended interventions, (C) bias due to missing outcome data, (D) bias in the measurement of the outcome, (E) bias in the selection of the reported result, (F) overall bias.

	Fermented foods			Control				Std. mean difference
Study or Subgroup	Mean	SD	Total	Mean	SD	Total	Weight	IV, Random, 95% CI
**4.18.1 8 weeks**								
**Subtotal**			**0**			**0**		**Not estimable**
Test for overall effect: Not applicable								
Heterogeneity: Not applicable								
**4.18.2 4 weeks**								
Guyonnet 2009	1.74	1.100227249	100	2.06	1.014642794	97	36.4%	-0.30 [-0.58 , -0.02]
Koebnick 2003	0.75	0.23	35	1	0.47	35	15.7%	-0.67 [-1.15 , -0.19]
**Subtotal (Wald[a])**			**135**			**132**	**52.1%**	**-0.43 [-0.77 , -0.09]**
Test for overall effect: Z = 2.45 (P = 0.01)								
Heterogeneity: Tau² (REML[b], 95% CI) = 0.03 [0.00 , 68.60]; Chi² = 1.66, df = 1 (P = 0.20); I² = 40%								
**4.18.3 16 weeks**								
**Subtotal**			**0**			**0**		**Not estimable**
Test for overall effect: Not applicable								
Heterogeneity: Not applicable								
**4.18.4 6 weeks**								
**Subtotal**			**0**			**0**		**Not estimable**
Test for overall effect: Not applicable								
Heterogeneity: Not applicable								
**4.18.5 2 weeks**								
Guyonnet (1-pot) 2009	1.97	0.95	144	2.62	0.93	35	23.7%	-0.68 [-1.06 , -0.31]
Guyonnet (2-pot) 2009	2.24	1.14	147	2.62	0.93	35	24.3%	-0.34 [-0.71 , 0.03]
**Subtotal (Wald[a])**			**291**			**70**	**47.9%**	**-0.51 [-0.85 , -0.18]**
Test for overall effect: Z = 3.00 (P = 0.003)								
Heterogeneity: Tau² (REML[b], 95% CI) = 0.02 [0.00 , 59.20]; Chi² = 1.60, df = 1 (P = 0.21); I² = 38%								
**4.18.6 3 weeks**								
**Subtotal**			**0**			**0**		**Not estimable**
Test for overall effect: Not applicable								
Heterogeneity: Not applicable								
**4.18.7 12 weeks**								
**Subtotal**			**0**			**0**		**Not estimable**
Test for overall effect: Not applicable								
Heterogeneity: Not applicable								
**4.18.8 24 weeks**								
**Subtotal**			**0**			**0**		**Not estimable**
Test for overall effect: Not applicable								
Heterogeneity: Not applicable								
**Total (Wald[a])**			**426**			**202**	**100.0%**	**-0.46 [-0.67 , -0.25]**
95% prediction interval								[-0.74 , -0.18]

Test for overall effect: Z = 4.38 (P < 0.0001)
Test for subgroup differences: Chi² = 0.11, df = 1 (P = 0.74), I² = 0%
Heterogeneity: Tau² (REML[b], 95% CI) = 0.01 [0.00 , 0.55]; Chi² = 3.67, df = 3 (P = 0.30); I² = 22%

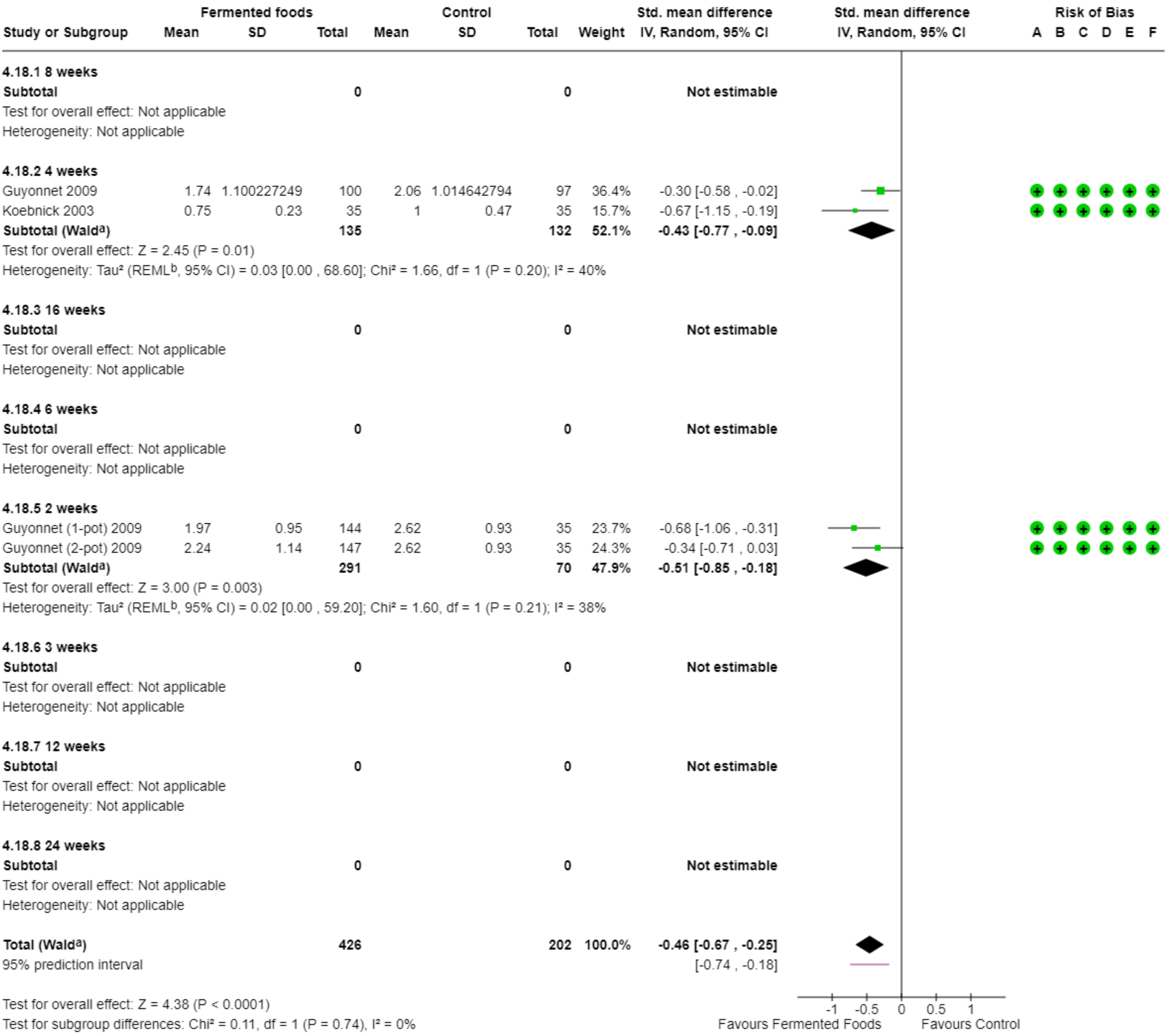

Supplementary Figure S13B. Subgroup analysis based on the intervention duration for flatulence. Forest plot of subgroup analysis based on the intervention duration for flatulence in randomised controlled trials comparing fermented foods with control in healthy adults (n=628). Values were calculated as standardised mean difference (95% CIs) using a random-effects model. CI, confidence interval; IV, inverse variance; SD, standard deviation; [a]CI calculated by Wald-type method; [b]Tau^2 calculated using Restricted Maximum-Likelihood method; Risk of bias legend: (A) bias arising from the randomisation process, (B) bias due to deviations from intended interventions, (C) bias due to missing outcome data, (D) bias in the measurement of the outcome, (E) bias in the selection of the reported result, (F) overall bias.

Study or Subgroup	Fermented foods Mean	Fermented foods SD	Fermented foods Total	Control Mean	Control SD	Control Total	Weight	Std. mean difference IV, Random, 95% CI	A	B	C	D	E	F
**4.19.1 < 10^10 CFU/day**														
Koebnick 2003	0.75	0.23	35	1	0.47	35	15.7%	-0.67 [-1.15 , -0.19]	+	+	+	+	+	+
**Subtotal**			**35**			**35**	**15.7%**	**-0.67 [-1.15 , -0.19]**						
Test for overall effect: Z = 2.72 (P = 0.007)														
Heterogeneity: Not applicable														
**4.19.2 >= 10^10 CFU/day**														
Guyonnet (1-pot) 2009	1.97	0.95	144	2.62	0.93	35	23.7%	-0.68 [-1.06 , -0.31]	+	+	+	+	+	+
Guyonnet (2-pot) 2009	2.24	1.14	147	2.62	0.93	35	24.3%	-0.34 [-0.71 , 0.03]	+	+	+	+	+	+
Guyonnet 2009	1.74	1.100227249	100	2.06	1.014642794	97	36.4%	-0.30 [-0.58 , -0.02]	+	+	+	+	+	+
**Subtotal (Wald[a])**			**391**			**167**	**84.3%**	**-0.42 [-0.65 , -0.20]**						
Test for overall effect: Z = 3.66 (P = 0.0003)														
Heterogeneity: Tau² (REML[b], 95% CI) = 0.01 [0.00 , 1.71]; Chi² = 2.74, df = 2 (P = 0.25); I² = 25%														
**Total (Wald[a])**			**426**			**202**	**100.0%**	**-0.46 [-0.67 , -0.25]**						
95% prediction interval								[-0.74 , -0.18]						

Test for overall effect: Z = 4.38 (P < 0.0001)
Test for subgroup differences: Chi² = 0.83, df = 1 (P = 0.36), I² = 0%
Heterogeneity: Tau² (REML[b], 95% CI) = 0.01 [0.00 , 0.55]; Chi² = 3.67, df = 3 (P = 0.30); I² = 22%

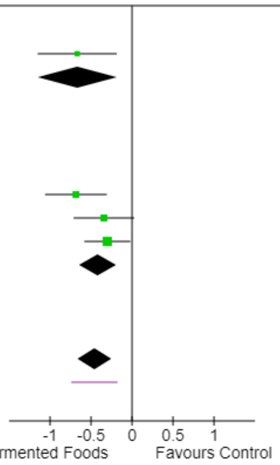

Supplementary Figure S13C. Subgroup analysis based on the microbial dosage for flatulence. Forest plot of subgroup analysis based on the microbial dosage for flatulence in randomised controlled trials comparing fermented foods with control in healthy adults (n=628). Values were calculated as standardised mean difference (95% CIs) using a random-effects model. CI, confidence interval; IV, inverse variance; SD, standard deviation; [a]CI calculated by Wald-type method; [b]Tau^2 calculated using Restricted Maximum-Likelihood method; Risk of bias legend: (A) bias arising from the randomisation process, (B) bias due to deviations from intended interventions, (C) bias due to missing outcome data, (D) bias in the measurement of the outcome, (E) bias in the selection of the reported result, (F) overall bias.

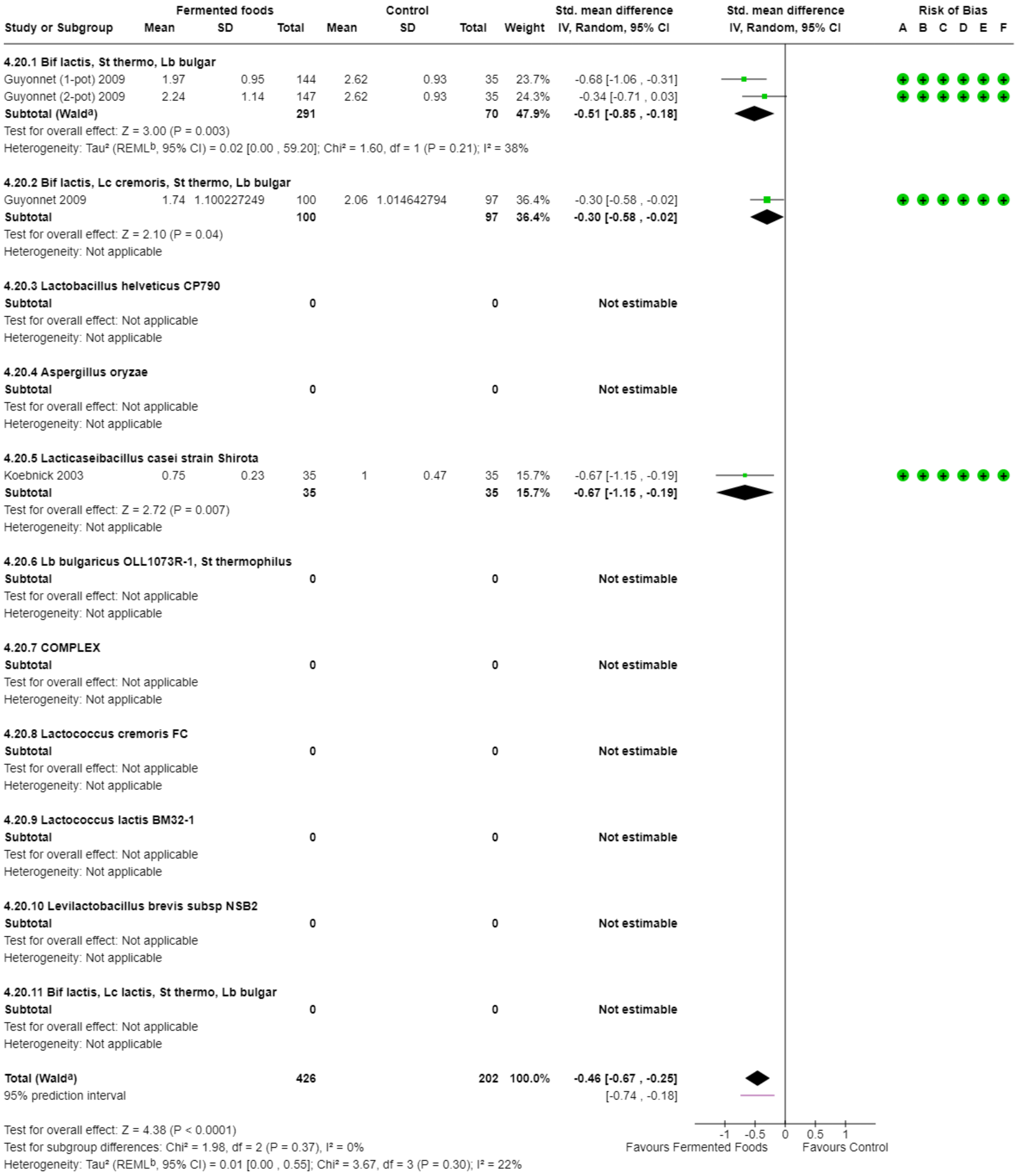

Supplementary Figure S13D. Subgroup analysis based on the fermentation microorganisms for flatulence. Forest plot of subgroup analysis based on the fermentation microorganisms for flatulence in randomised controlled trials comparing fermented foods with control in healthy adults (n=628). Values were calculated as standardised mean difference (95% CIs) using a random-effects model. COMPLEX, complex fermenting microbial community of several microorganisms consisting primarily of lactic acid bacteria; CI, confidence interval; IV, inverse variance; SD, standard deviation; [a]CI calculated by Wald-type method; [b]Tau^2 calculated using Restricted Maximum-Likelihood method; Risk of bias legend: (A) bias arising from the randomisation process, (B) bias due to deviations from intended interventions,

(C) bias due to missing outcome data, (D) bias in the measurement of the outcome, (E) bias in the selection of the reported result, (F) overall bias.

Study or Subgroup	Fermented foods Mean	Fermented foods SD	Fermented foods Total	Control Mean	Control SD	Control Total	Weight	Std. mean difference IV, Random, 95% CI
**3.5.1 Sericin-fibroin mixture**								
**Subtotal**			**0**			**0**		**Not estimable**
Test for overall effect: Not applicable								
Heterogeneity: Not applicable								
**3.5.2 Milk**								
Guyonnet (1-pot) 2009	1.49	0.72	144	1.97	1.04	35	26.4%	-0.60 [-0.98 , -0.23]
Guyonnet (2-pot) 2009	1.48	0.73	147	1.97	1.04	35	26.4%	-0.61 [-0.99 , -0.24]
Kato-Kataoka 2016	4.2	0.34	22	5.08	0.49	24	22.7%	-2.03 [-2.76 , -1.31]
Koebnick 2003	1.25	0.24	35	2	0.48	35	24.4%	-1.95 [-2.53 , -1.38]
**Subtotal (Wald[a])**			**348**			**129**	**100.0%**	**-1.26 [-2.04 , -0.48]**
Test for overall effect: Z = 3.16 (P = 0.002)								
Heterogeneity: Tau² (REML[b], 95% CI) = 0.57 [0.13 , 8.85]; Chi² = 26.77, df = 3 (P < 0.00001); I² = 91%								
**3.5.3 Brown rice**								
**Subtotal**			**0**			**0**		**Not estimable**
Test for overall effect: Not applicable								
Heterogeneity: Not applicable								
**3.5.4 Turnips**								
**Subtotal**			**0**			**0**		**Not estimable**
Test for overall effect: Not applicable								
Heterogeneity: Not applicable								
**3.5.5 Cabbage/cucumber**								
**Subtotal**			**0**			**0**		**Not estimable**
Test for overall effect: Not applicable								
Heterogeneity: Not applicable								
**3.5.6 Koji rice**								
**Subtotal**			**0**			**0**		**Not estimable**
Test for overall effect: Not applicable								
Heterogeneity: Not applicable								
**3.5.7 Soy milk**								
**Subtotal**			**0**			**0**		**Not estimable**
Test for overall effect: Not applicable								
Heterogeneity: Not applicable								
**Total (Wald[a])**			**348**			**129**	**100.0%**	**-1.26 [-2.04 , -0.48]**
95% prediction interval								[-2.93 , 0.41]

Test for overall effect: Z = 3.16 (P = 0.002)
Test for subgroup differences: Not applicable
Heterogeneity: Tau² (REML[b], 95% CI) = 0.57 [0.13 , 8.85]; Chi² = 26.77, df = 3 (P < 0.00001); I² = 91%

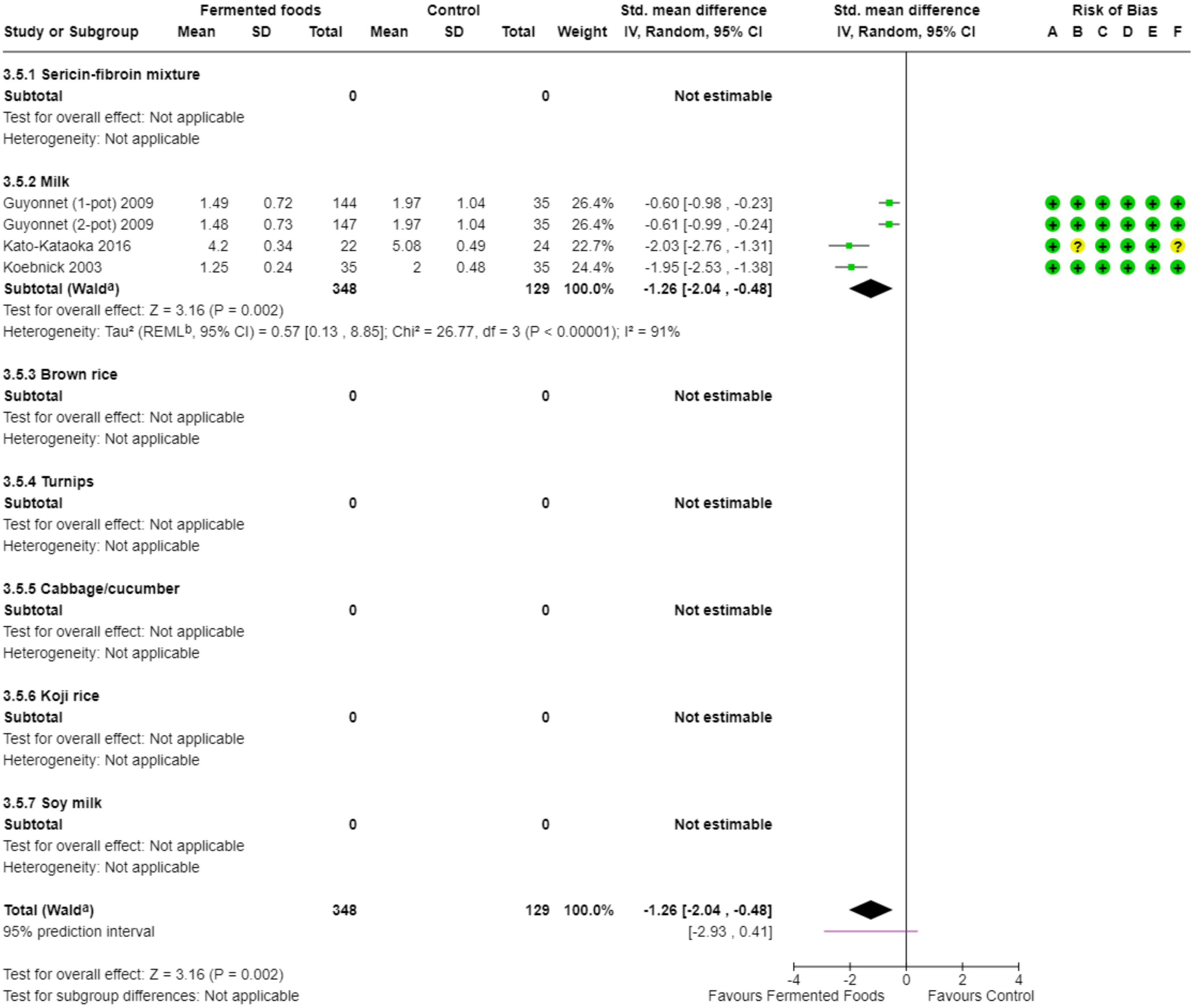

Supplementary Figure S14A. Subgroup analysis based on the type of fermentation matrix for degree of constipation. Forest plot of subgroup analysis based on the type of fermentation matrix for degree of constipation in randomised controlled trials comparing fermented foods with control in healthy adults (n=477). Values were calculated as standardized mean difference (95% CIs) using a random-effects model. CI, confidence interval; IV, inverse variance; SD, standard deviation; [a]CI calculated by Wald-type method; [b]Tau^2 calculated using Restricted Maximum-Likelihood method; Risk of bias legend: (A) bias arising from the randomisation process, (B) bias due to deviations from intended interventions, (C) bias due to missing outcome data, (D) bias in the measurement of the outcome, (E) bias in the selection of the reported result, (F) overall bias.

Study or Subgroup	Fermented foods Mean	SD	Total	Control Mean	SD	Total	Weight	Std. mean difference IV, Random, 95% CI	Risk of Bias A	B	C	D	E	F
**3.6.1 8 weeks**														
Kato-Kataoka 2016	4.2	0.34	22	5.08	0.49	24	22.7%	-2.03 [-2.76 , -1.31]	+	?	+	+	+	?
**Subtotal**			**22**			**24**	**22.7%**	**-2.03 [-2.76 , -1.31]**						
Test for overall effect: Z = 5.51 (P < 0.00001)														
Heterogeneity: Not applicable														
**3.6.2 4 weeks**														
Koebnick 2003	1.25	0.24	35	2	0.48	35	24.4%	-1.95 [-2.53 , -1.38]	+	+	+	+	+	+
**Subtotal**			**35**			**35**	**24.4%**	**-1.95 [-2.53 , -1.38]**						
Test for overall effect: Z = 6.66 (P < 0.00001)														
Heterogeneity: Not applicable														
**3.6.3 16 weeks**														
**Subtotal**			**0**			**0**		**Not estimable**						
Test for overall effect: Not applicable														
Heterogeneity: Not applicable														
**3.6.4 6 weeks**														
**Subtotal**			**0**			**0**		**Not estimable**						
Test for overall effect: Not applicable														
Heterogeneity: Not applicable														
**3.6.5 2 weeks**														
Guyonnet (1-pot) 2009	1.49	0.72	144	1.97	1.04	35	26.4%	-0.60 [-0.98 , -0.23]	+	+	+	+	+	+
Guyonnet (2-pot) 2009	1.48	0.73	147	1.97	1.04	35	26.4%	-0.61 [-0.99 , -0.24]	+	+	+	+	+	+
**Subtotal (Wald[a])**			**291**			**70**	**52.9%**	**-0.61 [-0.87 , -0.34]**						
Test for overall effect: Z = 4.50 (P < 0.00001)														
Heterogeneity: Tau² (REML[b], 95% CI) = 0.00 [not estimable]; Chi² = 0.00, df = 1 (P = 0.98); I² = 0%														
**3.6.6 3 weeks**														
**Subtotal**			**0**			**0**		**Not estimable**						
Test for overall effect: Not applicable														
Heterogeneity: Not applicable														
**3.6.7 12 weeks**														
**Subtotal**			**0**			**0**		**Not estimable**						
Test for overall effect: Not applicable														
Heterogeneity: Not applicable														
**3.6.8 24 weeks**														
**Subtotal**			**0**			**0**		**Not estimable**						
Test for overall effect: Not applicable														
Heterogeneity: Not applicable														
**Total (Wald[a])**			**348**			**129**	**100.0%**	**-1.26 [-2.04 , -0.48]**						
95% prediction interval								[-2.93 , 0.41]						

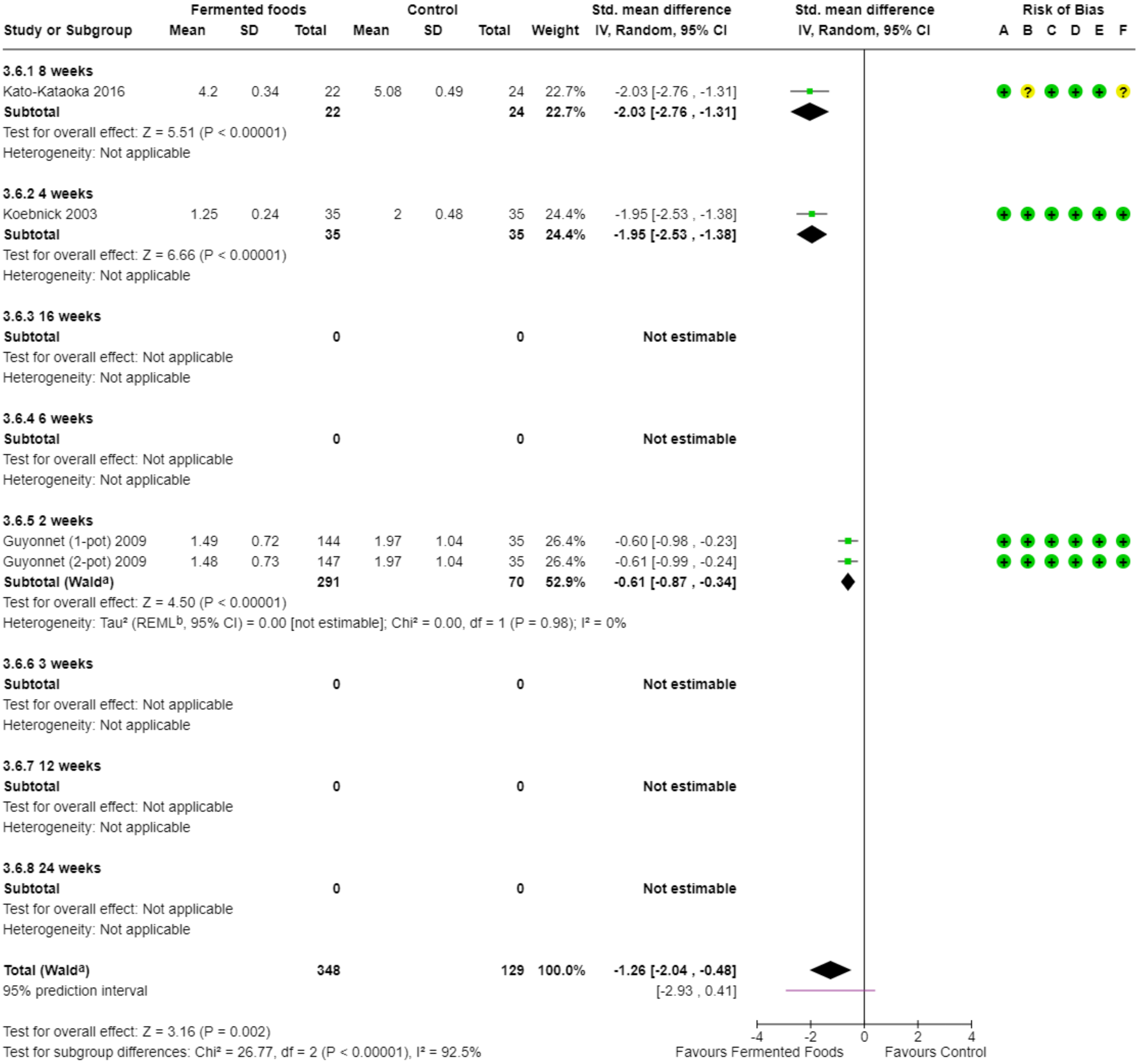

Test for overall effect: Z = 3.16 (P = 0.002)
Test for subgroup differences: Chi² = 26.77, df = 2 (P < 0.00001), I² = 92.5%
Heterogeneity: Tau² (REML[b], 95% CI) = 0.57 [0.13 , 8.85]; Chi² = 26.77, df = 3 (P < 0.00001); I² = 91%

Supplementary Figure S14B. Subgroup analysis based on the intervention duration for degree of constipation. Forest plot of subgroup analysis based on the intervention duration for degree of constipation in randomised controlled trials comparing fermented foods with control in healthy adults (n=477). Values were calculated as standardised mean difference (95% CIs) using a random-effects model. CI, confidence interval; IV, inverse variance; SD, standard deviation; [a]CI calculated by Wald-type method; [b]Tau^2 calculated using Restricted Maximum-Likelihood method; Risk of bias legend: (A) bias arising from the randomisation process, (B) bias due to deviations from intended interventions, (C) bias due to missing outcome data, (D) bias in the measurement of the outcome, (E) bias in the selection of the reported result, (F) overall bias.

Study or Subgroup	Fermented foods Mean	SD	Total	Control Mean	SD	Total	Weight	Std. mean difference IV, Random, 95% CI	Risk of Bias A	B	C	D	E	F
**3.7.1 < 10^10 CFU/day**														
Koebnick 2003	1.25	0.24	35	2	0.48	35	24.4%	-1.95 [-2.53 , -1.38]	+	+	+	+	+	+
**Subtotal**			**35**			**35**	**24.4%**	**-1.95 [-2.53 , -1.38]**						
Test for overall effect: Z = 6.66 (P < 0.00001)														
Heterogeneity: Not applicable														
**3.7.2 >= 10^10 CFU/day**														
Guyonnet (1-pot) 2009	1.49	0.72	144	1.97	1.04	35	26.4%	-0.60 [-0.98 , -0.23]	+	+	+	+	+	+
Guyonnet (2-pot) 2009	1.48	0.73	147	1.97	1.04	35	26.4%	-0.61 [-0.99 , -0.24]	+	+	+	+	+	+
Kato-Kataoka 2016	4.2	0.34	22	5.08	0.49	24	22.7%	-2.03 [-2.76 , -1.31]	+	?	+	+	+	?
**Subtotal (Wald[a])**			**313**			**94**	**75.6%**	**-1.03 [-1.91 , -0.15]**						
Test for overall effect: Z = 2.30 (P = 0.02)														
Heterogeneity: Tau² (REML[b], 95% CI) = 0.54 [0.08 , 26.71]; Chi² = 13.17, df = 2 (P = 0.001); I² = 91%														
**Total (Wald[a])**			**348**			**129**	**100.0%**	**-1.26 [-2.04 , -0.48]**						
95% prediction interval								[-2.93 , 0.41]						

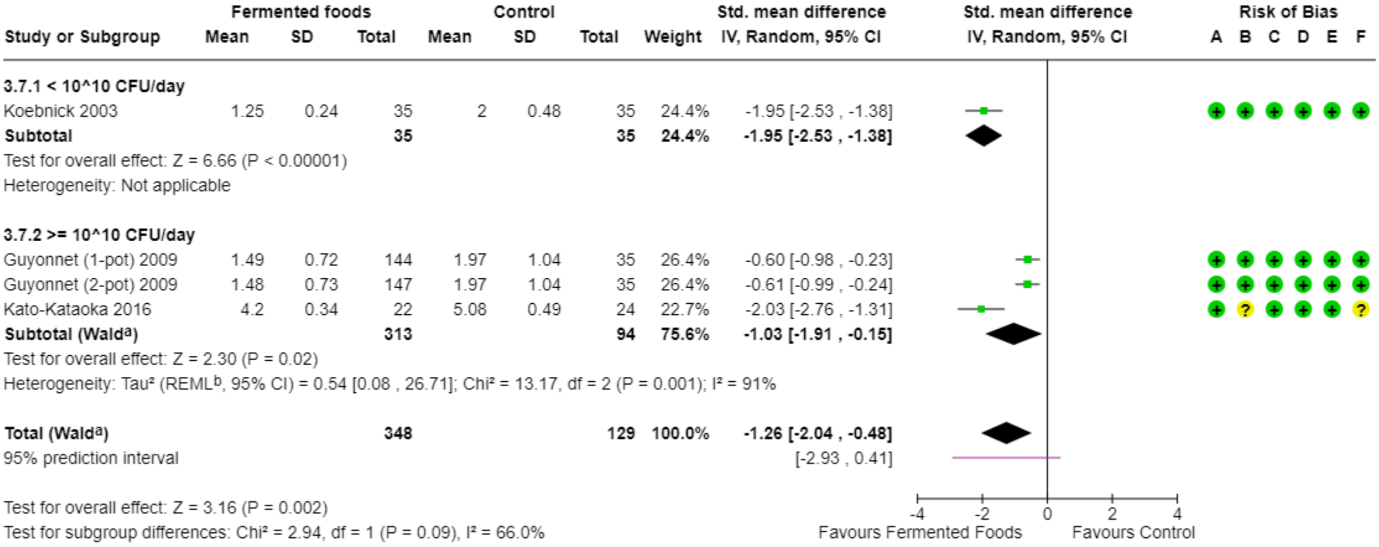

Test for overall effect: Z = 3.16 (P = 0.002)
Test for subgroup differences: Chi² = 2.94, df = 1 (P = 0.09), I² = 66.0%
Heterogeneity: Tau² (REML[b], 95% CI) = 0.57 [0.13 , 8.85]; Chi² = 26.77, df = 3 (P < 0.00001); I² = 91%

Supplementary Figure S14C. Subgroup analysis based on the microbial dosage for degree of constipation. Forest plot of subgroup analysis based on the microbial dosage for degree of constipation in randomised controlled trials comparing fermented foods with control in healthy adults (n=477). Values were calculated as standardised mean difference (95% CIs) using a random-effects model. CI, confidence interval; IV, inverse variance; SD, standard deviation; [a]CI calculated by Wald-type method; [b]Tau^2 calculated using Restricted Maximum-Likelihood method; Risk of bias legend: (A) bias arising from the randomisation process, (B) bias due to deviations from intended interventions, (C) bias due to missing outcome data, (D) bias in the measurement of the outcome, (E) bias in the selection of the reported result, (F) overall bias.

Study or Subgroup	Fermented foods Mean	Fermented foods SD	Fermented foods Total	Control Mean	Control SD	Control Total	Weight	Std. mean difference IV, Random, 95% CI	Risk of Bias A	B	C	D	E	F
**3.8.1 Bif lactis, St thermo, Lb bulgar**														
Guyonnet (1-pot) 2009	1.49	0.72	144	1.97	1.04	35	26.4%	-0.60 [-0.98 , -0.23]	+	+	+	+	+	+
Guyonnet (2-pot) 2009	1.48	0.73	147	1.97	1.04	35	26.4%	-0.61 [-0.99 , -0.24]	+	+	+	+	+	+
**Subtotal (Wald[a])**			**291**			**70**	**52.9%**	**-0.61 [-0.87 , -0.34]**						
Test for overall effect: Z = 4.50 (P < 0.00001)														
Heterogeneity: Tau^2 (REML[b], 95% CI) = 0.00 [not estimable]; Chi^2 = 0.00, df = 1 (P = 0.98); I^2 = 0%														
**3.8.2 Bif lactis, Lc cremoris, St thermo, Lb bulgar**														
**Subtotal**			**0**			**0**		**Not estimable**						
Test for overall effect: Not applicable														
Heterogeneity: Not applicable														
**3.8.3 Lactobacillus helveticus CP790**														
**Subtotal**			**0**			**0**		**Not estimable**						
Test for overall effect: Not applicable														
Heterogeneity: Not applicable														
**3.8.4 Aspergillus oryzae**														
**Subtotal**			**0**			**0**		**Not estimable**						
Test for overall effect: Not applicable														
Heterogeneity: Not applicable														
**3.8.5 Lacticaseibacillus casei strain Shirota**														
Kato-Kataoka 2016	4.2	0.34	22	5.08	0.49	24	22.7%	-2.03 [-2.76 , -1.31]	+	?	+	+	+	?
Koebnick 2003	1.25	0.24	35	2	0.48	35	24.4%	-1.95 [-2.53 , -1.38]	+	+	+	+	+	+
**Subtotal (Wald[a])**			**57**			**59**	**47.1%**	**-1.99 [-2.44 , -1.54]**						
Test for overall effect: Z = 8.64 (P < 0.00001)														
Heterogeneity: Tau^2 (REML[b], 95% CI) = 0.00 [0.00 , 3.17]; Chi^2 = 0.03, df = 1 (P = 0.86); I^2 = 0%														
**3.8.6 Lb bulgaricus OLL1073R-1, St thermophilus**														
**Subtotal**			**0**			**0**		**Not estimable**						
Test for overall effect: Not applicable														
Heterogeneity: Not applicable														
**3.8.7 COMPLEX**														
**Subtotal**			**0**			**0**		**Not estimable**						
Test for overall effect: Not applicable														
Heterogeneity: Not applicable														
**3.8.8 Lactococcus cremoris FC**														
**Subtotal**			**0**			**0**		**Not estimable**						
Test for overall effect: Not applicable														
Heterogeneity: Not applicable														
**3.8.9 Lactococcus lactis BM32-1**														
**Subtotal**			**0**			**0**		**Not estimable**						
Test for overall effect: Not applicable														
Heterogeneity: Not applicable														
**3.8.10 Levilactobacillus brevis subsp NSB2**														
**Subtotal**			**0**			**0**		**Not estimable**						
Test for overall effect: Not applicable														
Heterogeneity: Not applicable														
**3.8.11 Bif lactis, Lc lactis, St thermo, Lb bulgar**														
**Subtotal**			**0**			**0**		**Not estimable**						
Test for overall effect: Not applicable														
Heterogeneity: Not applicable														
**Total (Wald[a])**			**348**			**129**	**100.0%**	**-1.26 [-2.04 , -0.48]**						
95% prediction interval								[-2.93 , 0.41]						

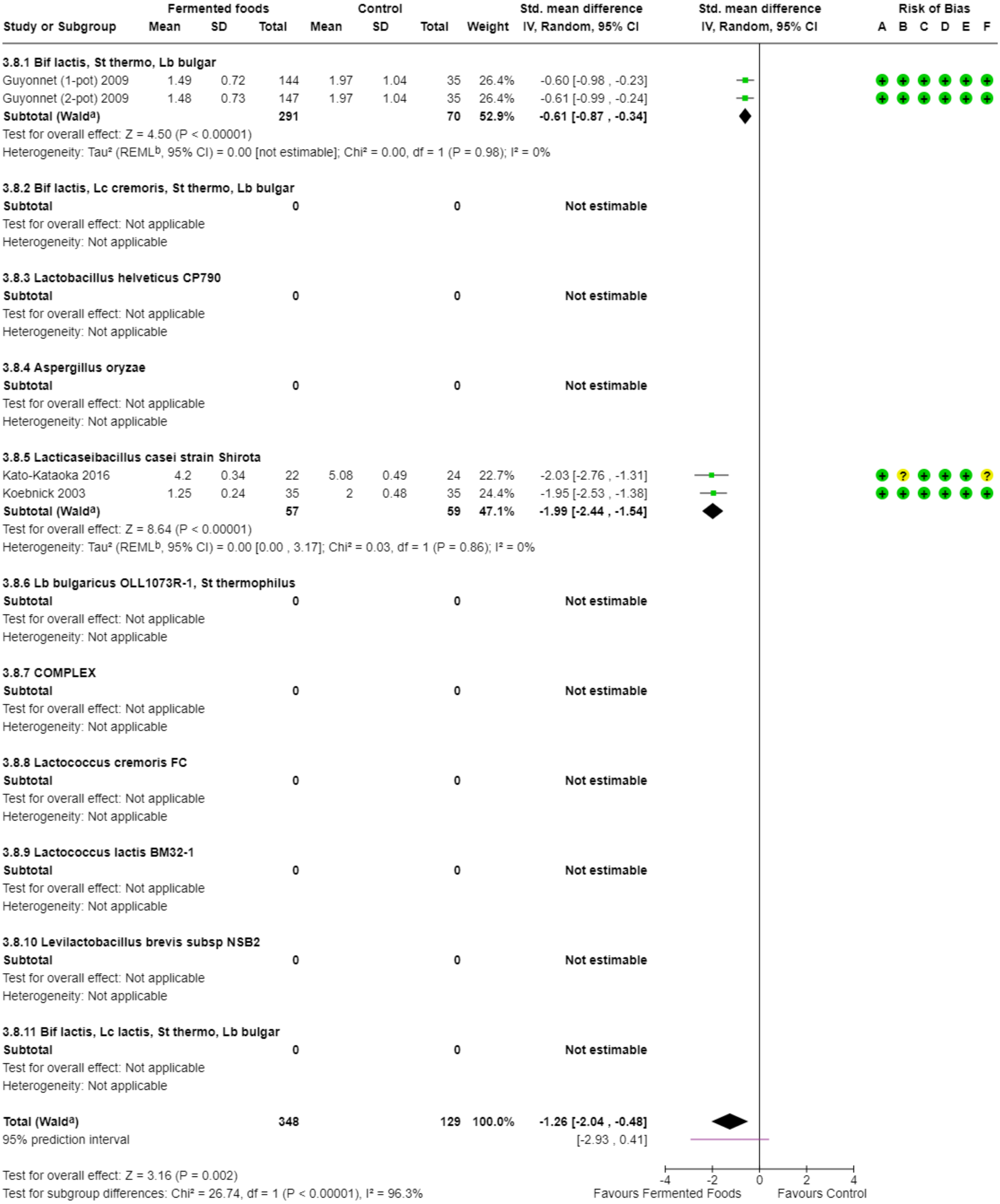

Test for overall effect: Z = 3.16 (P = 0.002)
Test for subgroup differences: Chi^2 = 26.74, df = 1 (P < 0.00001), I^2 = 96.3%
Heterogeneity: Tau^2 (REML[b], 95% CI) = 0.57 [0.13 , 8.85]; Chi^2 = 26.77, df = 3 (P < 0.00001); I^2 = 91%

Supplementary Figure S14D. Subgroup analysis based on the fermentation microorganisms for degree of constipation. Forest plot of subgroup analysis based on the fermentation microorganisms for degree of constipation in randomised controlled trials comparing fermented foods with control in healthy adults (n=477). Values were calculated as standardised mean difference (95% CIs) using a random-effects model. COMPLEX, complex fermenting microbial community of several microorganisms consisting primarily of lactic acid bacteria; CI, confidence interval; IV,

inverse variance; SD, standard deviation; [a]CI calculated by Wald-type method; [b]Tau^2 calculated using Restricted Maximum-Likelihood method; Risk of bias legend: (A) bias arising from the randomisation process, (B) bias due to deviations from intended interventions, (C) bias due to missing outcome data, (D) bias in the measurement of the outcome, (E) bias in the selection of the reported result, (F) overall bias.

Study or Subgroup	Fermented foods Mean	Fermented foods SD	Fermented foods Total	Control Mean	Control SD	Control Total	Weight	Std. mean difference IV, Random, 95% CI	Risk of Bias A	B	C	D	E	F
**3.9.1 Sericin-fibroin mixture**														
**Subtotal**			**0**			**0**		**Not estimable**						
Test for overall effect: Not applicable														
Heterogeneity: Not applicable														
**3.9.2 Milk**														
Kato-Kataoka 2016	0.41	0.13	24	0.71	0.17	22	32.2%	-1.96 [-2.67 , -1.25]	+	?	+	+	+	?
Sakai 2011	1.47	0.55	19	1.57	0.6	20	33.0%	-0.17 [-0.80 , 0.46]	+	?	+	+	+	?
Tanihiro 2024	1.79	0.6	57	1.7	0.69	60	34.8%	0.14 [-0.22 , 0.50]	+	+	+	+	+	+
**Subtotal (Wald[a])**			**100**			**102**	**100.0%**	**-0.64 [-1.91 , 0.63]**						
Test for overall effect: Z = 0.99 (P = 0.32)														
Heterogeneity: Tau² (REML[b], 95% CI) = 1.16 [0.24 , 50.60]; Chi² = 26.46, df = 2 (P < 0.00001); I² = 94%														
**3.9.3 Brown rice**														
**Subtotal**			**0**			**0**		**Not estimable**						
Test for overall effect: Not applicable														
Heterogeneity: Not applicable														
**3.9.4 Turnips**														
**Subtotal**			**0**			**0**		**Not estimable**						
Test for overall effect: Not applicable														
Heterogeneity: Not applicable														
**3.9.5 Cabbage/cucumber**														
**Subtotal**			**0**			**0**		**Not estimable**						
Test for overall effect: Not applicable														
Heterogeneity: Not applicable														
**3.9.6 Koji rice**														
**Subtotal**			**0**			**0**		**Not estimable**						
Test for overall effect: Not applicable														
Heterogeneity: Not applicable														
**3.9.7 Soy milk**														
**Subtotal**			**0**			**0**		**Not estimable**						
Test for overall effect: Not applicable														
Heterogeneity: Not applicable														
**Total (Wald[a])**			**100**			**102**	**100.0%**	**-0.64 [-1.91 , 0.63]**						
95% prediction interval								[-3.10 , 1.83]						

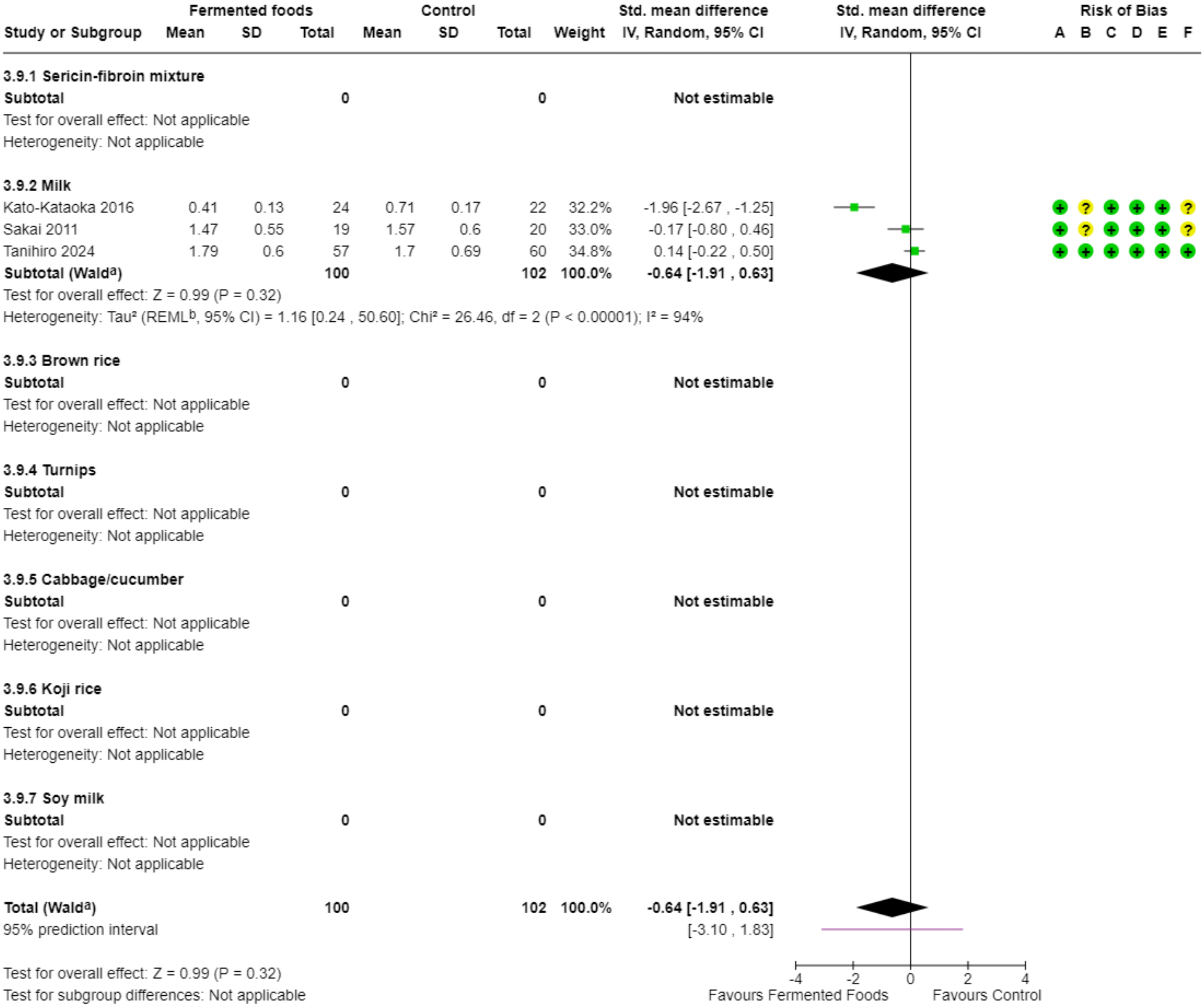

Test for overall effect: Z = 0.99 (P = 0.32)
Test for subgroup differences: Not applicable
Heterogeneity: Tau² (REML[b], 95% CI) = 1.16 [0.24 , 50.60]; Chi² = 26.46, df = 2 (P < 0.00001); I² = 94%

Supplementary Figure S15A. Subgroup analysis based on the fermentation matrix for feeling of incomplete evacuation. Forest plot of subgroup analysis based on the fermentation matrix for feeling of incomplete evacuation in randomised controlled trials comparing fermented foods with control in healthy adults (n=202). Values were calculated as standardised mean difference (95% CIs) using a random-effects model. CI, confidence interval; IV, inverse variance; SD, standard deviation; [a]CI calculated by Wald-type method; [b]Tau^2 calculated using Restricted Maximum-Likelihood method; Risk of bias legend: (A) bias arising from the randomisation process, (B) bias due to deviations from intended interventions, (C) bias due to missing outcome data, (D) bias in the measurement of the outcome, (E) bias in the selection of the reported result, (F) overall bias.

Study or Subgroup	Fermented foods Mean	Fermented foods SD	Fermented foods Total	Control Mean	Control SD	Control Total	Weight	Std. mean difference IV, Random, 95% CI	Risk of Bias A	B	C	D	E	F
**3.10.1 8 weeks**														
Kato-Kataoka 2016	0.41	0.13	24	0.71	0.17	22	32.2%	-1.96 [-2.67 , -1.25]	+	?	+	+	+	?
**Subtotal**			**24**			**22**	**32.2%**	**-1.96 [-2.67 , -1.25]**						
Test for overall effect: Z = 5.38 (P < 0.00001)														
Heterogeneity: Not applicable														
**3.10.2 4 weeks**														
Tanihiro 2024	1.79	0.6	57	1.7	0.69	60	34.8%	0.14 [-0.22 , 0.50]	+	+	+	+	+	+
**Subtotal**			**57**			**60**	**34.8%**	**0.14 [-0.22 , 0.50]**						
Test for overall effect: Z = 0.75 (P = 0.46)														
Heterogeneity: Not applicable														
**3.10.3 16 weeks**														
**Subtotal**			**0**			**0**		**Not estimable**						
Test for overall effect: Not applicable														
Heterogeneity: Not applicable														
**3.10.4 6 weeks**														
**Subtotal**			**0**			**0**		**Not estimable**						
Test for overall effect: Not applicable														
Heterogeneity: Not applicable														
**3.10.5 2 weeks**														
**Subtotal**			**0**			**0**		**Not estimable**						
Test for overall effect: Not applicable														
Heterogeneity: Not applicable														
**3.10.6 3 weeks**														
Sakai 2011	1.47	0.55	19	1.57	0.6	20	33.0%	-0.17 [-0.80 , 0.46]	+	?	+	+	+	?
**Subtotal**			**19**			**20**	**33.0%**	**-0.17 [-0.80 , 0.46]**						
Test for overall effect: Z = 0.53 (P = 0.60)														
Heterogeneity: Not applicable														
**3.10.7 12 weeks**														
**Subtotal**			**0**			**0**		**Not estimable**						
Test for overall effect: Not applicable														
Heterogeneity: Not applicable														
**3.10.8 24 weeks**														
**Subtotal**			**0**			**0**		**Not estimable**						
Test for overall effect: Not applicable														
Heterogeneity: Not applicable														
**Total (Wald[a])**			**100**			**102**	**100.0%**	**-0.64 [-1.91 , 0.63]**						
95% prediction interval								[-3.10 , 1.83]						

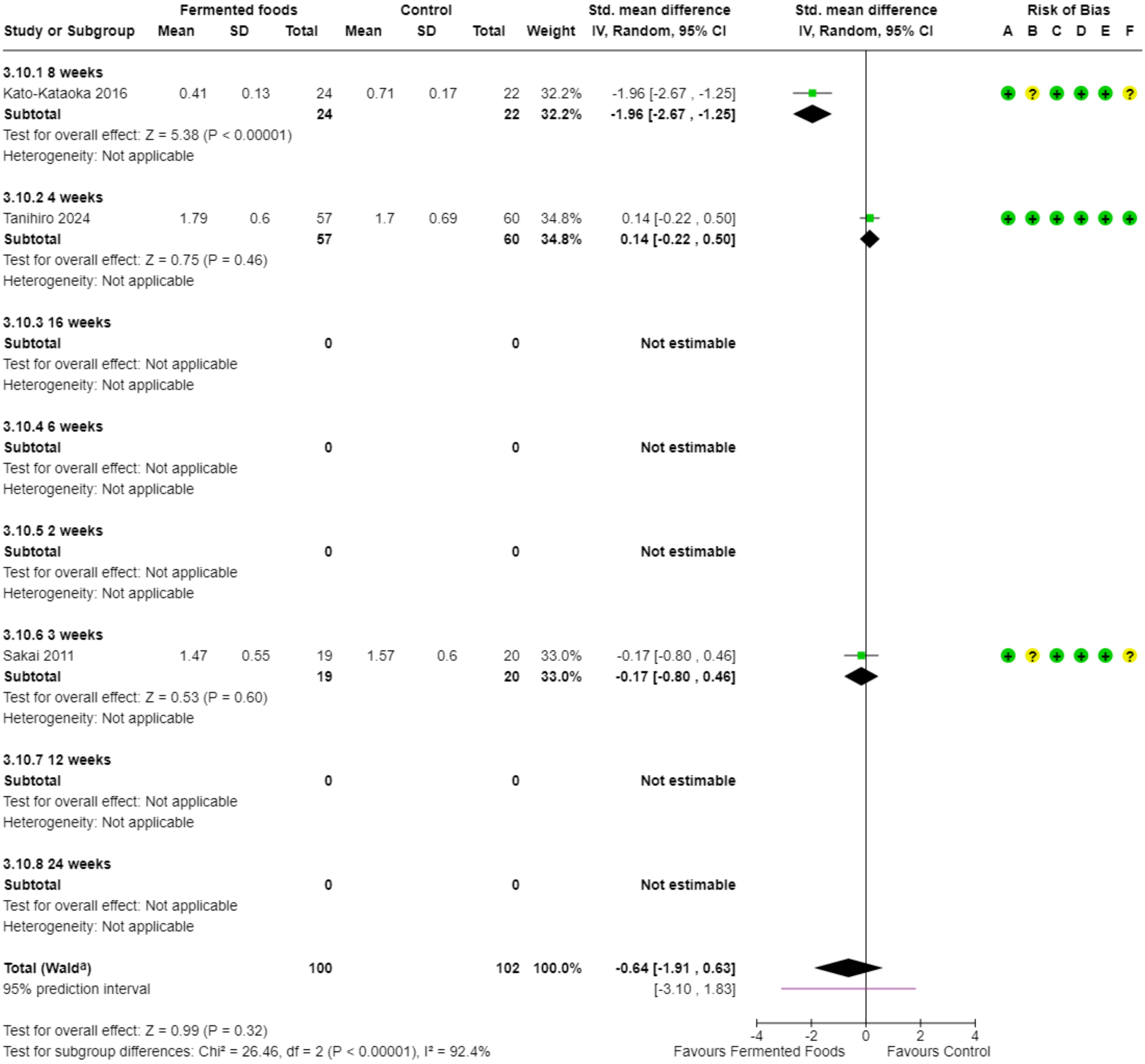

Test for overall effect: Z = 0.99 (P = 0.32)
Test for subgroup differences: Chi² = 26.46, df = 2 (P < 0.00001), I² = 92.4%
Heterogeneity: Tau² (REML[b], 95% CI) = 1.16 [0.24 , 50.60]; Chi² = 26.46, df = 2 (P < 0.00001); I² = 94%

Supplementary Figure S15B. Subgroup analysis based on the intervention duration for feeling of incomplete evacuation. Forest plot of subgroup analysis based on the intervention duration for feeling of incomplete evacuation in randomised controlled trials comparing fermented foods with control in healthy adults (n=202). Values were calculated as standardised mean difference (95% CIs) using a random-effects model. CI, confidence interval; IV, inverse variance; SD, standard deviation; [a]CI calculated by Wald-type method; [b]Tau^2 calculated using Restricted Maximum-Likelihood method; Risk of bias legend: (A) bias arising from the randomisation process, (B) bias due to deviations from intended interventions, (C) bias due to missing outcome data, (D) bias in the measurement of the outcome, (E) bias in the selection of the reported result, (F) overall bias.

Study or Subgroup	Fermented foods Mean	SD	Total	Control Mean	SD	Total	Weight	Std. mean difference IV, Random, 95% CI	Risk of Bias A	B	C	D	E	F
**3.11.1 < 10^10 CFU/day**														
Sakai 2011	1.47	0.55	19	1.57	0.6	20	33.0%	-0.17 [-0.80 , 0.46]	+	?	+	+	+	?
**Subtotal**			**19**			**20**	**33.0%**	**-0.17 [-0.80 , 0.46]**						
Test for overall effect: Z = 0.53 (P = 0.60)														
Heterogeneity: Not applicable														
**3.11.2 >= 10^10 CFU/day**														
Kato-Kataoka 2016	0.41	0.13	24	0.71	0.17	22	32.2%	-1.96 [-2.67 , -1.25]	+	?	+	+	+	?
Tanihiro 2024	1.79	0.6	57	1.7	0.69	60	34.8%	0.14 [-0.22 , 0.50]	+	+	+	+	+	+
**Subtotal (Wald[a])**			**81**			**82**	**67.0%**	**-0.89 [-2.94 , 1.17]**						
Test for overall effect: Z = 0.85 (P = 0.40)														
Heterogeneity: Tau² (REML[b], 95% CI) = 2.12 [0.35 , >100]; Chi² = 26.35, df = 1 (P < 0.00001); I² = 96%														
**Total (Wald[a])**			**100**			**102**	**100.0%**	**-0.64 [-1.91 , 0.63]**						
95% prediction interval								[-3.10 , 1.83]						

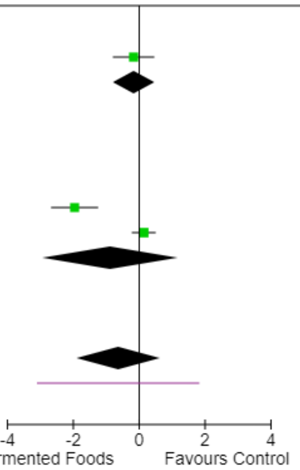

Test for overall effect: Z = 0.99 (P = 0.32)
Test for subgroup differences: Chi² = 0.43, df = 1 (P = 0.51), I² = 0%
Heterogeneity: Tau² (REML[b], 95% CI) = 1.16 [0.24 , 50.60]; Chi² = 26.46, df = 2 (P < 0.00001); I² = 94%

Supplementary Figure S15C. Subgroup analysis based on the microbial dosage for feeling of incomplete evacuation. Forest plot of subgroup analysis based on the microbial dosage for feeling of incomplete evacuation in randomised controlled trials comparing fermented foods with control in healthy adults (n=202). Values were calculated as standardised mean difference (95% CIs) using a random-effects model. CI, confidence interval; IV, inverse variance; SD, standard deviation; [a]CI calculated by Wald-type method; [b]Tau^2 calculated using Restricted Maximum-Likelihood method; Risk of bias legend: (A) bias arising from the randomisation process, (B) bias due to deviations from intended interventions, (C) bias due to missing outcome data, (D) bias in the measurement of the outcome, (E) bias in the selection of the reported result, (F) overall bias.

Study or Subgroup	Fermented foods Mean	SD	Total	Control Mean	SD	Total	Weight	Std. mean difference IV, Random, 95% CI	Risk of Bias A	B	C	D	E	F
**3.12.1 Bif lactis, St thermo, Lb bulgar**														
**Subtotal**			**0**			**0**		**Not estimable**						
Test for overall effect: Not applicable														
Heterogeneity: Not applicable														
**3.12.2 Bif lactis, Lc cremoris, St thermo, Lb bulgar**														
**Subtotal**			**0**			**0**		**Not estimable**						
Test for overall effect: Not applicable														
Heterogeneity: Not applicable														
**3.12.3 Lactobacillus helveticus CP790**														
Tanihiro 2024	1.79	0.6	57	1.7	0.69	60	34.8%	0.14 [-0.22 , 0.50]	+	+	+	+	+	+
**Subtotal**			**57**			**60**	**34.8%**	**0.14 [-0.22 , 0.50]**						
Test for overall effect: Z = 0.75 (P = 0.46)														
Heterogeneity: Not applicable														
**3.12.4 Aspergillus oryzae**														
**Subtotal**			**0**			**0**		**Not estimable**						
Test for overall effect: Not applicable														
Heterogeneity: Not applicable														
**3.12.5 Lacticaseibacillus casei strain Shirota**														
Kato-Kataoka 2016	0.41	0.13	24	0.71	0.17	22	32.2%	-1.96 [-2.67 , -1.25]	+	?	+	+	+	?
Sakai 2011	1.47	0.55	19	1.57	0.6	20	33.0%	-0.17 [-0.80 , 0.46]	+	?	+	+	+	?
**Subtotal (Wald[a])**			**43**			**42**	**65.2%**	**-1.06 [-2.81 , 0.70]**						
Test for overall effect: Z = 1.18 (P = 0.24)														
Heterogeneity: Tau^2 (REML[b], 95% CI) = 1.48 [0.20 , >100]; Chi^2 = 13.59, df = 1 (P = 0.0002); I^2 = 93%														
**3.12.6 Lb bulgaricus OLL1073R-1, St thermophilus**														
**Subtotal**			**0**			**0**		**Not estimable**						
Test for overall effect: Not applicable														
Heterogeneity: Not applicable														
**3.12.7 COMPLEX**														
**Subtotal**			**0**			**0**		**Not estimable**						
Test for overall effect: Not applicable														
Heterogeneity: Not applicable														
**3.12.8 Lactococcus cremoris FC**														
**Subtotal**			**0**			**0**		**Not estimable**						
Test for overall effect: Not applicable														
Heterogeneity: Not applicable														
**3.12.9 Lactococcus lactis BM32-1**														
**Subtotal**			**0**			**0**		**Not estimable**						
Test for overall effect: Not applicable														
Heterogeneity: Not applicable														
**3.12.10 Levilactobacillus brevis subsp NSB2**														
**Subtotal**			**0**			**0**		**Not estimable**						
Test for overall effect: Not applicable														
Heterogeneity: Not applicable														
**3.12.11 Bif lactis, Lc lactis, St thermo, Lb bulgar**														
**Subtotal**			**0**			**0**		**Not estimable**						
Test for overall effect: Not applicable														
Heterogeneity: Not applicable														
**Total (Wald[a])**			**100**			**102**	**100.0%**	**-0.64 [-1.91 , 0.63]**						
95% prediction interval								[-3.10 , 1.83]						

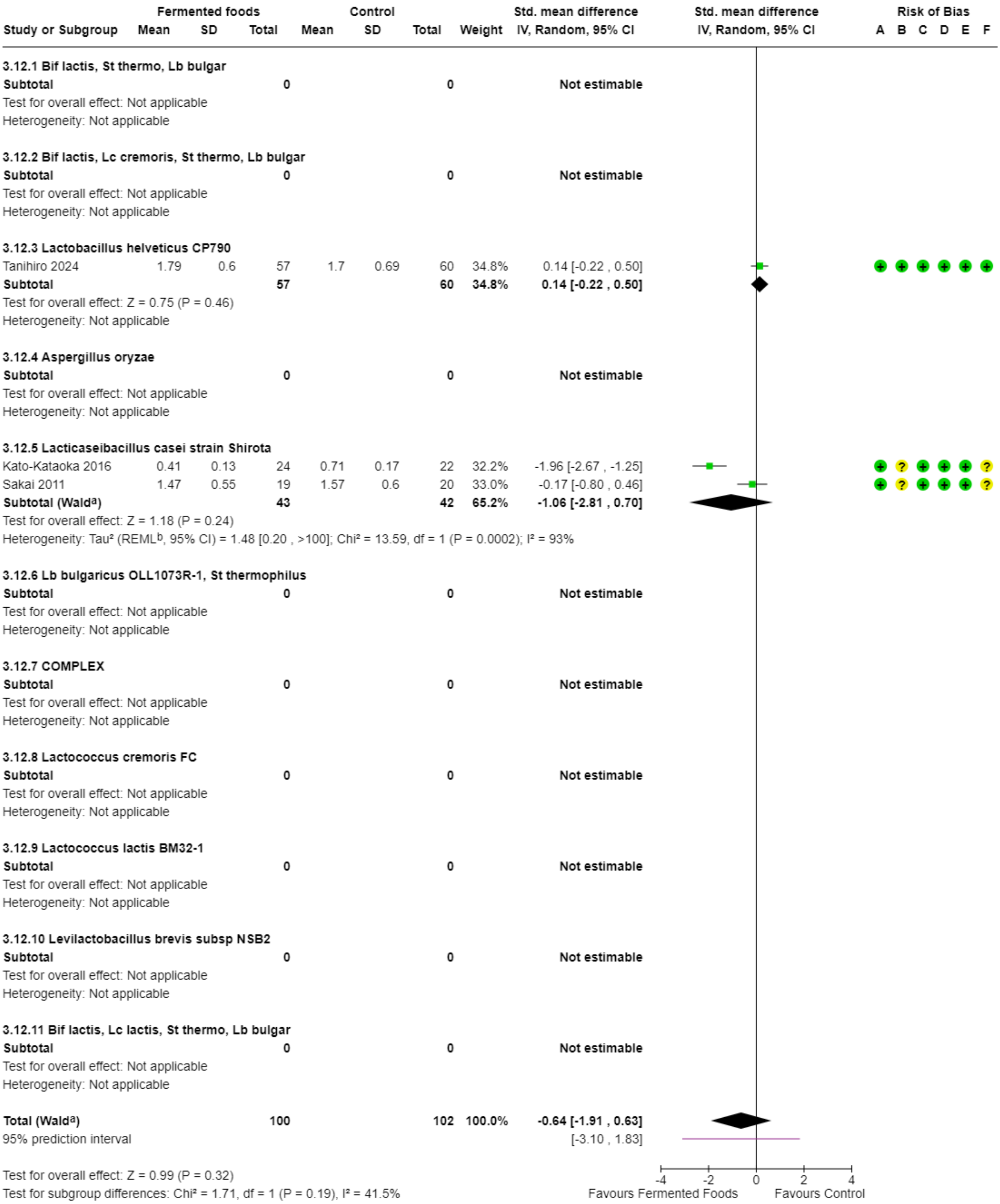

Test for overall effect: Z = 0.99 (P = 0.32)
Test for subgroup differences: Chi^2 = 1.71, df = 1 (P = 0.19), I^2 = 41.5%
Heterogeneity: Tau^2 (REML[b], 95% CI) = 1.16 [0.24 , 50.60]; Chi^2 = 26.46, df = 2 (P < 0.00001); I^2 = 94%

Supplementary Figure S15D. Subgroup analysis based on the fermentation microorganisms for feeling of incomplete evacuation. Forest plot of subgroup analysis based on the fermentation microorganisms for feeling of incomplete evacuation in randomised controlled trials comparing fermented foods with control in healthy adults (n=202). Values were calculated as standardised mean difference (95% CIs) using a random-effects model. COMPLEX, complex fermenting microbial community of several microorganisms consisting primarily of lactic acid bacteria; CI, confidence

interval; IV, inverse variance; SD, standard deviation; [a]CI calculated by Wald-type method; [b]Tau^2 calculated using Restricted Maximum-Likelihood method; Risk of bias legend: (A) bias arising from the randomisation process, (B) bias due to deviations from intended interventions, (C) bias due to missing outcome data, (D) bias in the measurement of the outcome, (E) bias in the selection of the reported result, (F) overall bias.

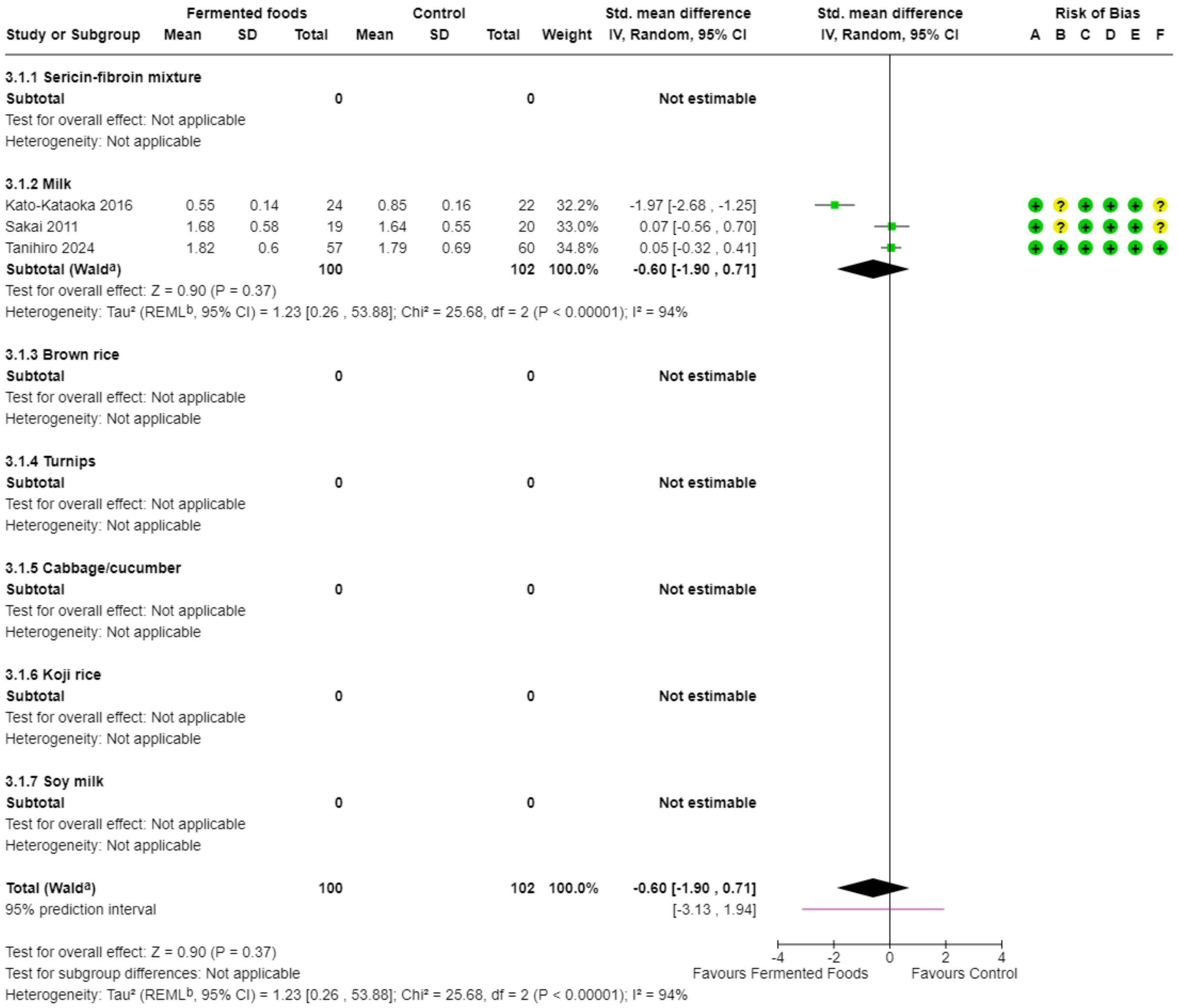

Supplementary Figure S16A. Subgroup analysis based on the fermentation matrix for straining during defaecation. Forest plot of subgroup analysis based on the fermentation matrix for straining during defaecation in randomised controlled trials comparing fermented foods with control in healthy adults (n=202). Values were calculated as standardised mean difference (95% CIs) using a random-effects model. CI, confidence interval; IV, inverse variance; SD, standard deviation; [a]CI calculated by Wald-type method; [b]Tau^2 calculated using Restricted Maximum-Likelihood method; Risk of bias legend: (A) bias arising from the randomisation process, (B) bias due to deviations from intended interventions, (C) bias due to missing outcome data, (D) bias in the measurement of the outcome, (E) bias in the selection of the reported result, (F) overall bias.

Study or Subgroup	Fermented foods Mean	Fermented foods SD	Fermented foods Total	Control Mean	Control SD	Control Total	Weight	Std. mean difference IV, Random, 95% CI	Risk of Bias A	B	C	D	E	F
**3.2.1 8 weeks**														
Kato-Kataoka 2016	0.55	0.14	24	0.85	0.16	22	32.2%	-1.97 [-2.68 , -1.25]	+	?	+	+	+	?
**Subtotal**			**24**			**22**	**32.2%**	**-1.97 [-2.68 , -1.25]**						
Test for overall effect: Z = 5.39 (P < 0.00001)														
Heterogeneity: Not applicable														
**3.2.2 4 weeks**														
Tanihiro 2024	1.82	0.6	57	1.79	0.69	60	34.8%	0.05 [-0.32 , 0.41]	+	+	+	+	+	+
**Subtotal**			**57**			**60**	**34.8%**	**0.05 [-0.32 , 0.41]**						
Test for overall effect: Z = 0.25 (P = 0.80)														
Heterogeneity: Not applicable														
**3.2.3 16 weeks**														
**Subtotal**			**0**			**0**		**Not estimable**						
Test for overall effect: Not applicable														
Heterogeneity: Not applicable														
**3.2.4 6 weeks**														
**Subtotal**			**0**			**0**		**Not estimable**						
Test for overall effect: Not applicable														
Heterogeneity: Not applicable														
**3.2.5 2 weeks**														
**Subtotal**			**0**			**0**		**Not estimable**						
Test for overall effect: Not applicable														
Heterogeneity: Not applicable														
**3.2.6 3 weeks**														
Sakai 2011	1.68	0.58	19	1.64	0.55	20	33.0%	0.07 [-0.56 , 0.70]	+	?	+	+	+	?
**Subtotal**			**19**			**20**	**33.0%**	**0.07 [-0.56 , 0.70]**						
Test for overall effect: Z = 0.22 (P = 0.83)														
Heterogeneity: Not applicable														
**3.2.7 12 weeks**														
**Subtotal**			**0**			**0**		**Not estimable**						
Test for overall effect: Not applicable														
Heterogeneity: Not applicable														
**3.2.8 24 weeks**														
**Subtotal**			**0**			**0**		**Not estimable**						
Test for overall effect: Not applicable														
Heterogeneity: Not applicable														
**Total (Wald[a])**			**100**			**102**	**100.0%**	**-0.60 [-1.90 , 0.71]**						
95% prediction interval								[-3.13 , 1.94]						

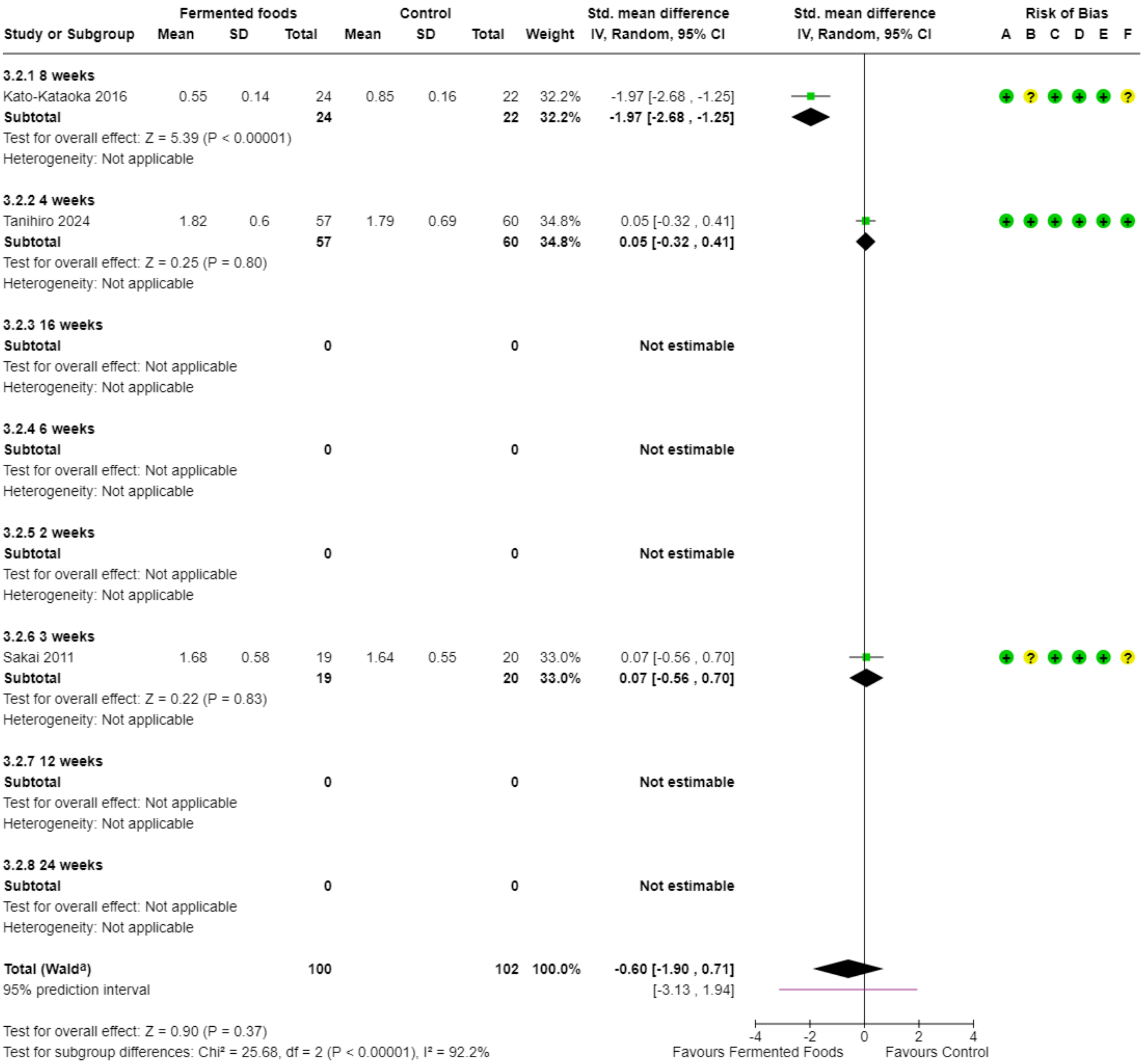

Test for overall effect: Z = 0.90 (P = 0.37)
Test for subgroup differences: Chi² = 25.68, df = 2 (P < 0.00001), I² = 92.2%
Heterogeneity: Tau² (REML[b], 95% CI) = 1.23 [0.26 , 53.88]; Chi² = 25.68, df = 2 (P < 0.00001); I² = 94%

Supplementary Figure S16B. Subgroup analysis based on the intervention duration for straining during defaecation. Forest plot of subgroup analysis based on the intervention duration for straining during defaecation in randomised controlled trials comparing fermented foods with control in healthy adults (n=202). Values were calculated as standardised mean difference (95% CIs) using a random-effects model. CI, confidence interval; IV, inverse variance; SD, standard deviation; [a]CI calculated by Wald-type method; [b]Tau^2 calculated using Restricted Maximum-Likelihood method; Risk of bias legend: (A) bias arising from the randomisation process, (B) bias due to deviations from intended interventions, (C) bias due to missing outcome data, (D) bias in the measurement of the outcome, (E) bias in the selection of the reported result, (F) overall bias.

Study or Subgroup	Fermented foods Mean	SD	Total	Control Mean	SD	Total	Weight	Std. mean difference IV, Random, 95% CI
**3.3.1 < 10^10 CFU/day**								
Sakai 2011	1.68	0.58	19	1.64	0.55	20	33.0%	0.07 [-0.56 , 0.70]
**Subtotal**			**19**			**20**	**33.0%**	**0.07 [-0.56 , 0.70]**
Test for overall effect: Z = 0.22 (P = 0.83)								
Heterogeneity: Not applicable								
**3.3.2 >= 10^10 CFU/day**								
Kato-Kataoka 2016	0.55	0.14	24	0.85	0.16	22	32.2%	-1.97 [-2.68 , -1.25]
Tanihiro 2024	1.82	0.6	57	1.79	0.69	60	34.8%	0.05 [-0.32 , 0.41]
**Subtotal (Wald[a])**			**81**			**82**	**67.0%**	**-0.94 [-2.91 , 1.04]**
Test for overall effect: Z = 0.93 (P = 0.35)								
Heterogeneity: Tau² (REML[b], 95% CI) = 1.94 [0.32 , >100]; Chi² = 24.22, df = 1 (P < 0.00001); I² = 96%								
**Total (Wald[a])**			**100**			**102**	**100.0%**	**-0.60 [-1.90 , 0.71]**
95% prediction interval								[-3.13 , 1.94]

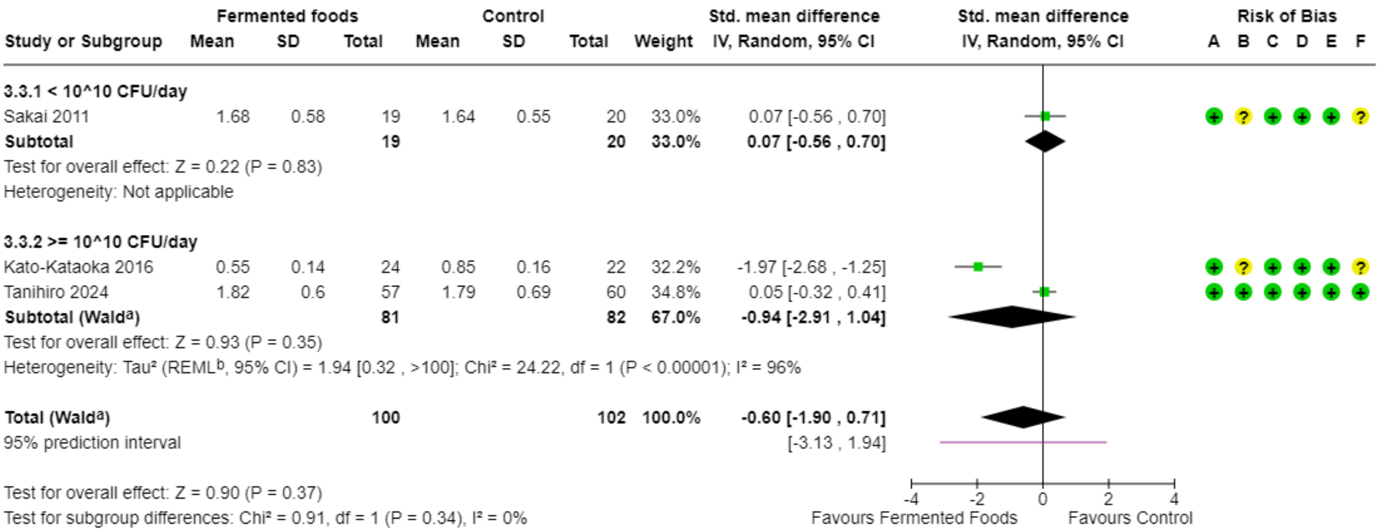

Test for overall effect: Z = 0.90 (P = 0.37)
Test for subgroup differences: Chi² = 0.91, df = 1 (P = 0.34), I² = 0%
Heterogeneity: Tau² (REML[b], 95% CI) = 1.23 [0.26 , 53.88]; Chi² = 25.68, df = 2 (P < 0.00001); I² = 94%

Supplementary Figure S16C. Subgroup analysis based on the microbial dosage for straining during defaecation. Forest plot of subgroup analysis based on the microbial dosage for straining during defaecation in randomised controlled trials comparing fermented foods with control in healthy adults (n=202). Values were calculated as standardised mean difference (95% CIs) using a random-effects model. CI, confidence interval; IV, inverse variance; SD, standard deviation; [a]CI calculated by Wald-type method; [b]Tau^2 calculated using Restricted Maximum-Likelihood method; Risk of bias legend: (A) bias arising from the randomisation process, (B) bias due to deviations from intended interventions, (C) bias due to missing outcome data, (D) bias in the measurement of the outcome, (E) bias in the selection of the reported result, (F) overall bias.

Study or Subgroup	Fermented foods Mean	SD	Total	Control Mean	SD	Total	Weight	Std. mean difference IV, Random, 95% CI	Risk of Bias A	B	C	D	E	F
**3.4.1 Bif lactis, St thermo, Lb bulgar**														
**Subtotal**			**0**			**0**		**Not estimable**						
Test for overall effect: Not applicable														
Heterogeneity: Not applicable														
**3.4.2 Bif lactis, Lc cremoris, St thermo, Lb bulgar**														
**Subtotal**			**0**			**0**		**Not estimable**						
Test for overall effect: Not applicable														
Heterogeneity: Not applicable														
**3.4.3 Lactobacillus helveticus CP790**														
Tanihiro 2024	1.82	0.6	57	1.79	0.69	60	34.8%	0.05 [-0.32 , 0.41]	+	+	+	+	+	+
**Subtotal**			**57**			**60**	**34.8%**	**0.05 [-0.32 , 0.41]**						
Test for overall effect: Z = 0.25 (P = 0.80)														
Heterogeneity: Not applicable														
**3.4.4 Aspergillus oryzae**														
**Subtotal**			**0**			**0**		**Not estimable**						
Test for overall effect: Not applicable														
Heterogeneity: Not applicable														
**3.4.5 Lacticaseibacillus casei strain Shirota**														
Kato-Kataoka 2016	0.55	0.14	24	0.85	0.16	22	32.2%	-1.97 [-2.68 , -1.25]	+	?	+	+	+	?
Sakai 2011	1.68	0.58	19	1.64	0.55	20	33.0%	0.07 [-0.56 , 0.70]	+	?	+	+	+	?
**Subtotal (Wald[a])**			**43**			**42**	**65.2%**	**-0.94 [-2.94 , 1.05]**						
Test for overall effect: Z = 0.92 (P = 0.36)														
Heterogeneity: Tau² (REML[b], 95% CI) = 1.96 [0.29 , >100]; Chi² = 17.59, df = 1 (P < 0.0001); I² = 94%														
**3.4.6 Lb bulgaricus OLL1073R-1, St thermophilus**														
**Subtotal**			**0**			**0**		**Not estimable**						
Test for overall effect: Not applicable														
Heterogeneity: Not applicable														
**3.4.7 COMPLEX**														
**Subtotal**			**0**			**0**		**Not estimable**						
Test for overall effect: Not applicable														
Heterogeneity: Not applicable														
**3.4.8 Lactococcus cremoris FC**														
**Subtotal**			**0**			**0**		**Not estimable**						
Test for overall effect: Not applicable														
Heterogeneity: Not applicable														
**3.4.9 Lactococcus lactis BM32-1**														
**Subtotal**			**0**			**0**		**Not estimable**						
Test for overall effect: Not applicable														
Heterogeneity: Not applicable														
**3.4.10 Levilactobacillus brevis subsp NSB2**														
**Subtotal**			**0**			**0**		**Not estimable**						
Test for overall effect: Not applicable														
Heterogeneity: Not applicable														
**3.4.11 Bif lactis, Lc lactis, St thermo, Lb bulgar**														
**Subtotal**			**0**			**0**		**Not estimable**						
Test for overall effect: Not applicable														
Heterogeneity: Not applicable														
**Total (Wald[a])**			**100**			**102**	**100.0%**	**-0.60 [-1.90 , 0.71]**						
95% prediction interval								[-3.13 , 1.94]						

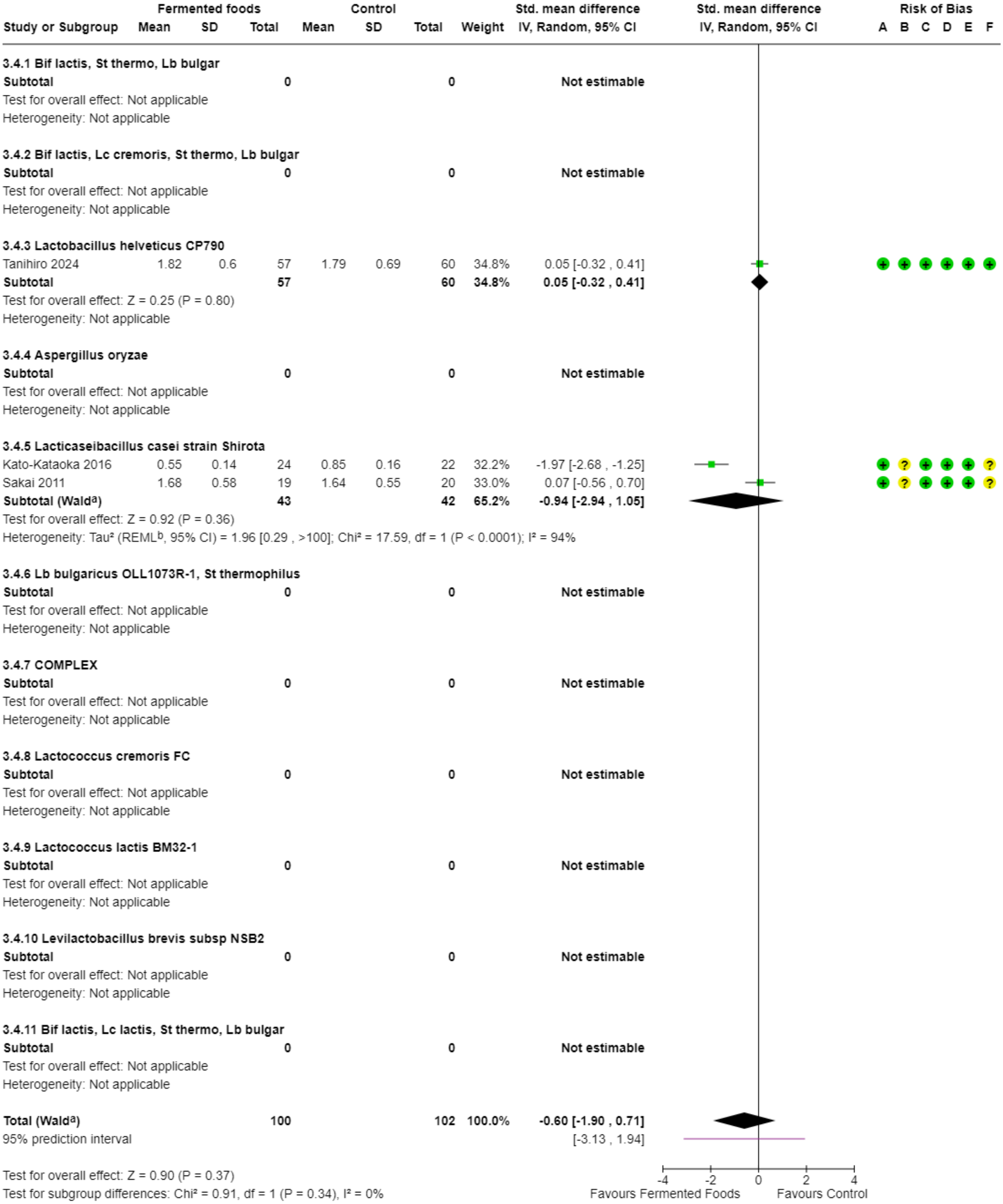

Test for overall effect: Z = 0.90 (P = 0.37)
Test for subgroup differences: Chi² = 0.91, df = 1 (P = 0.34), I² = 0%
Heterogeneity: Tau² (REML[b], 95% CI) = 1.23 [0.26 , 53.88]; Chi² = 25.68, df = 2 (P < 0.00001); I² = 94%

Supplementary Figure S16D. Subgroup analysis based on the fermentation microorganisms for straining during defaecation. Forest plot of subgroup analysis based on the fermentation microorganisms for straining during defaecation in randomised controlled trials comparing fermented foods with control in healthy adults (n=202). Values were calculated as standardised mean difference (95% CIs) using a random-effects model. COMPLEX, complex fermenting microbial community of several microorganisms consisting primarily of lactic acid bacteria; CI, confidence

interval; IV, inverse variance; SD, standard deviation; [a]CI calculated by Wald-type method; [b]Tau^2 calculated using Restricted Maximum-Likelihood method; Risk of bias legend: (A) bias arising from the randomisation process, (B) bias due to deviations from intended interventions, (C) bias due to missing outcome data, (D) bias in the measurement of the outcome, (E) bias in the selection of the reported result, (F) overall bias.

Study or Subgroup	Fermented foods Mean	SD	Total	Control Mean	SD	Total	Weight	Mean difference IV, Random, 95% CI	A	B	C	D	E	F
✔ Guyonnet 2009	8.72	3.734179964	100	8.18	2.16988479	97	15.1%	0.54 [-0.31 , 1.39]	+	+	+	+	+	+
✔ Kaga 2024	7.01	1.57	57	7.19	1.55	55	18.1%	-0.18 [-0.76 , 0.40]	+	?	+	+	+	?
✔ Koebnick 2003	5.75	0.24	35	5	0.47	35	21.3%	0.75 [0.58 , 0.92]	+	+	+	+	+	+
✔ Kurahashi 2021	6.73	2.76	22	5.09	9.18	22	2.0%	1.64 [-2.37 , 5.65]	+	+	+	+	+	+
✔ Matsumoto 2010	12.2	4.3	14	14.6	4.3	16	3.2%	-2.40 [-5.48 , 0.68]	+	?	+	+	+	?
✔ Noda 2024	5.7	2.18	25	3.97	0.88	25	14.3%	1.73 [0.81 , 2.65]	+	+	+	+	+	+
✖ Ozaki 2018	7.9	3.5	15	6.8	2.3	16	0.0%	1.10 [-1.00 , 3.20]	+	-	+	+	+	-
✔ Sakai 2011	7.9	3.1	19	6	2	20	8.2%	1.90 [0.25 , 3.55]	+	?	+	+	+	?
✔ Tanihiro 2024	5.39	1.74	57	5.37	1.63	60	17.7%	0.02 [-0.59 , 0.63]	+	+	+	+	+	+
**Total (Wald[a])**			**329**			**330**	**100.0%**	**0.57 [-0.03 , 1.17]**						
95% prediction interval								[-0.85 , 1.99]						

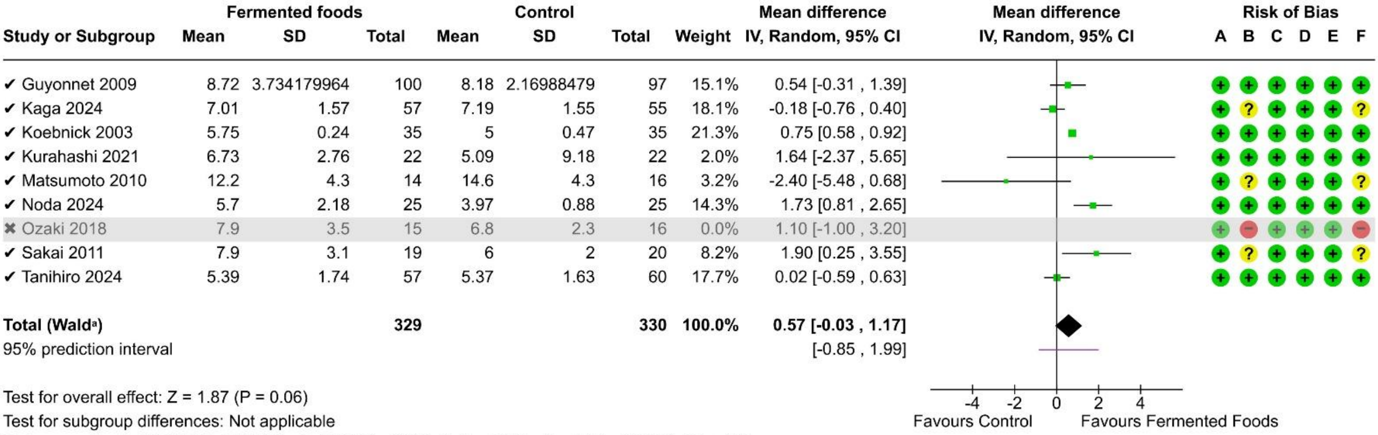

Test for overall effect: Z = 1.87 (P = 0.06)
Test for subgroup differences: Not applicable
Heterogeneity: Tau² (REML[b], 95% CI) = 0.43 [0.08 , 6.40]; Chi² = 24.79, df = 7 (P = 0.0008); I² = 78%

Supplementary Figure S17A. Forest plot of sensitivity analysis for stool frequency. A sensitivity analysis of the forest plot for stool frequency in randomised controlled trials comparing fermented foods with control in healthy adults (n=659) was carried out excluding the studies that were deemed at a high risk of bias. Values were calculated as mean difference (95% CIs) using a random-effects model. CI, confidence interval; IV, inverse variance; SD, standard deviation; [a]CI calculated by Wald-type method; [b]Tau^2 calculated using Restricted Maximum-Likelihood method; Risk of bias legend: (A) bias arising from the randomisation process, (B) bias due to deviations from intended interventions, (C) bias due to missing outcome data, (D) bias in the measurement of the outcome, (E) bias in the selection of the reported result, (F) overall bias.

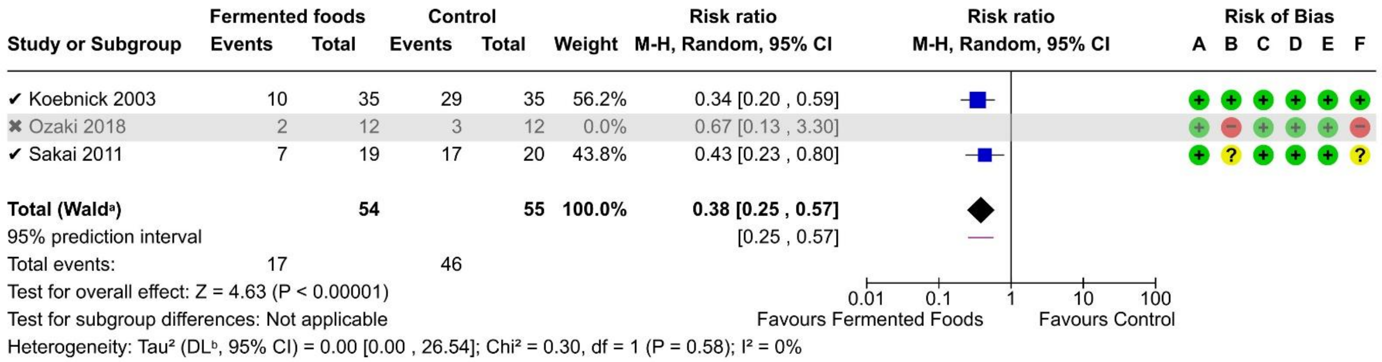

Supplementary Figure S17B. Forest plot of sensitivity analysis for hard stools. A sensitivity analysis of the forest plot for hard stools in randomised controlled trials comparing fermented foods with control in healthy adults (n=99) was carried out excluding the studies that were deemed at a high risk of bias. Values were calculated as risk ratio (95% CIs) using a random-effects model. CI, confidence interval; M-H, Mantel-Haenszel; RR, risk ratio; [a]CI calculated by Wald-type method; [b]Tau^2 calculated using DerSimonian and Laird method; Risk of bias legend: (A) bias arising from the randomisation process, (B) bias due to deviations from intended interventions, (C) bias due to missing outcome data, (D) bias in the measurement of the outcome, (E) bias in the selection of the reported result, (F) overall bias.

Study or Subgroup	Fermented foods Mean	SD	Total	Control Mean	SD	Total	Weight	Mean difference IV, Random, 95% CI	A	B	C	D	E	F
✔ Kaga 2024	6.8	0.64	57	6.64	0.54	55	49.0%	0.16 [-0.06 , 0.38]	+	?	+	+	+	?
✔ Kurahashi 2021	6.97	0.64	22	7	0.37	22	24.6%	-0.03 [-0.34 , 0.28]	+	+	+	+	+	+
✔ Matsumoto 2010	6.2	0.5	14	6.1	0.6	16	15.2%	0.10 [-0.29 , 0.49]	+	?	+	+	+	?
✖ Nagata (elder cohort) 2015	7.15	0.64	36	7.98	0.6	36	0.0%	-0.83 [-1.12 , -0.54]	+	−	+	+	+	−
✖ Nagata (nursing cohort) 2015	6.88	0.45	10	7.35	0.59	10	0.0%	-0.47 [-0.93 , -0.01]	+	−	+	+	+	−
✔ Nemoto 2011	7	0.41	18	7.1	0.9	18	11.3%	-0.10 [-0.56 , 0.36]	+	+	+	+	+	+
✖ Ozaki 2018	6.68	0.52	12	6.88	0.39	12	0.0%	-0.20 [-0.57 , 0.17]	+	−	+	+	+	−
✖ Spanhaak 1998	7	0.3	10	6.8	0.4	10	0.0%	0.20 [-0.11 , 0.51]	+	−	+	+	+	−
**Total (Wald[a])**			**111**			**111**	**100.0%**	**0.07 [-0.08 , 0.23]**						
95% prediction interval								[-0.08 , 0.23]						

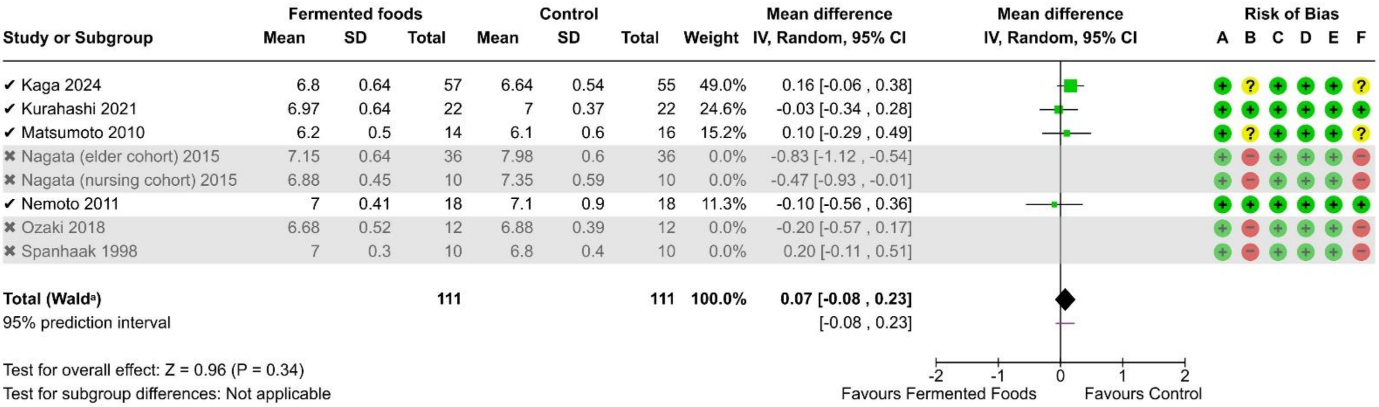

Test for overall effect: Z = 0.96 (P = 0.34)
Test for subgroup differences: Not applicable
Heterogeneity: Tau² (REML[b], 95% CI) = 0.00 [0.00 , 0.16]; Chi² = 1.60, df = 3 (P = 0.66); I² = 0%

Supplementary Figure S17C. Forest plot of sensitivity analysis for stool pH. A sensitivity analysis of the forest plot for stool pH in randomised controlled trials comparing fermented foods with control in healthy adults (n=222) was carried out excluding the studies that were deemed at a high risk of bias. Values were calculated as mean difference (95% CIs) using a random-effects model. CI, confidence interval; IV, inverse variance; SD, standard deviation; [a]CI calculated by Wald-type method; [b]Tau^2 calculated using Restricted Maximum-Likelihood method; Risk of bias legend: (A) bias arising from the randomisation process, (B) bias due to deviations from intended interventions, (C) bias due to missing outcome data, (D) bias in the measurement of the outcome, (E) bias in the selection of the reported result, (F) overall bias.

Study or Subgroup	Fermented foods Mean	SD	Total	Control Mean	SD	Total	Weight	Mean difference IV, Random, 95% CI	A	B	C	D	E	F
✔ Kaga 2024	75.2	7.9	57	73.6	8.3	55	37.3%	1.60 [-1.40 , 4.60]	+	?	+	+	+	?
✔ Matsumoto 2010	76.9	5.1	14	80.1	5.1	16	31.0%	-3.20 [-6.86 , 0.46]	+	?	+	+	+	?
✖ Ozaki 2018	77	7.8	12	72.1	6.6	12	0.0%	4.90 [-0.88 , 10.68]	+	−	+	+	+	−
✔ Sakai 2011	71.7	6	19	71	5.4	20	31.7%	0.70 [-2.89 , 4.29]	+	?	+	+	+	?
✖ Spanhaak 1998	75	3	10	75	2	10	0.0%	0.00 [-2.23 , 2.23]	+	−	+	+	+	−
**Total (Wald[a])**			**90**			**91**	**100.0%**	**-0.18 [-3.02 , 2.67]**						
95% prediction interval								[-4.73 , 4.38]						

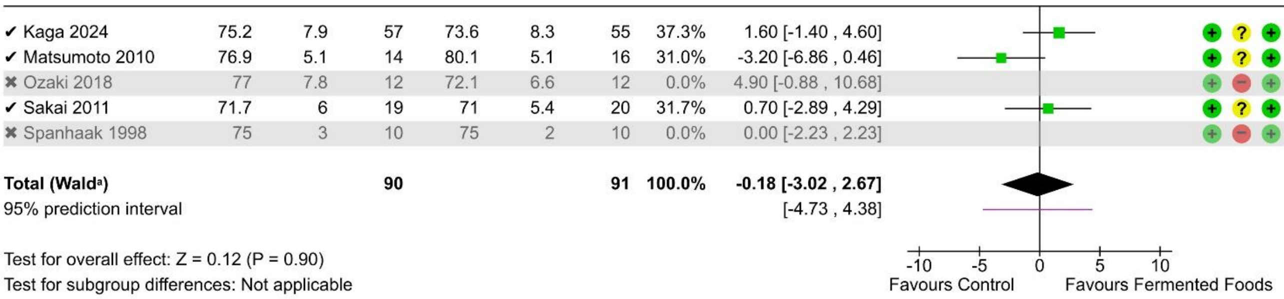

Test for overall effect: Z = 0.12 (P = 0.90)
Test for subgroup differences: Not applicable
Heterogeneity: Tau² (REML[b], 95% CI) = 3.29 [0.00 , >100]; Chi² = 4.18, df = 2 (P = 0.12); I² = 52%

Supplementary Figure S17D. Forest plot of sensitivity analysis for stool water content. A sensitivity analysis of the forest plot for stool water content in randomised controlled trials comparing fermented foods with control in healthy adults (n=181) was carried out excluding the studies that were deemed at a high risk of bias. Values were calculated as mean difference (95% CIs) using a random-effects model. CI, confidence interval; IV, inverse variance; SD, standard deviation; [a]CI calculated by Wald-type method; [b]Tau^2 calculated using Restricted Maximum-Likelihood method; Risk of bias legend: (A) bias arising from the randomisation process, (B) bias due to deviations from intended interventions, (C) bias due to missing outcome data, (D) bias in the measurement of the outcome, (E) bias in the selection of the reported result, (F) overall bias.

Study or Subgroup	Fermented foods Mean	SD	Total	Control Mean	SD	Total	Weight	Std. mean difference IV, Random, 95% CI	A	B	C	D	E	F
✔ Kurahashi 2021	752	360	22	647	386	22	35.3%	0.28 [-0.32 , 0.87]	+	+	+	+	+	+
✔ Matsumoto 2010	36	15.8	14	40.3	20.2	16	25.9%	-0.23 [-0.95 , 0.49]	+	?	+	+	+	?
✔ Noda 2024	3.41	0.57	25	3.61	0.59	25	38.8%	-0.34 [-0.90 , 0.22]	+	+	+	+	+	+
✖ Ozaki 2018	5.9	2.7	15	4.5	2.2	16	0.0%	0.56 [-0.16 , 1.28]	+	−	+	+	+	−
**Total (Wald[a])**			**61**			**63**	**100.0%**	**-0.09 [-0.49 , 0.31]**						
95% prediction interval								[-0.60 , 0.42]						

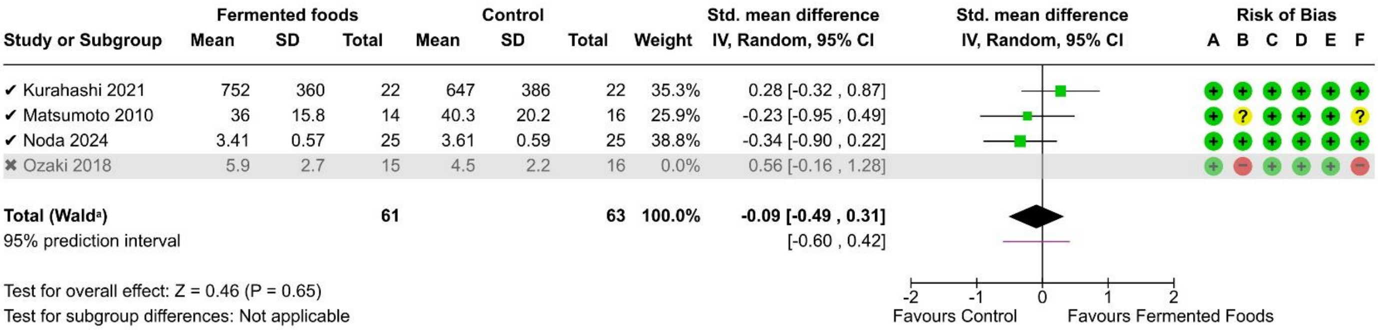

Test for overall effect: Z = 0.46 (P = 0.65)
Test for subgroup differences: Not applicable
Heterogeneity: Tau² (REML[b], 95% CI) = 0.03 [0.00 , 4.16]; Chi² = 2.37, df = 2 (P = 0.31); I² = 21%

Supplementary Figure S17E. Forest plot of sensitivity analysis for stool bulk. A sensitivity analysis of the forest plot for stool bulk in randomised controlled trials comparing fermented foods with control in healthy adults (n=124) was carried out excluding the studies that were deemed at a high risk of bias. Values were calculated as standardised mean difference (95% CIs) using a random-effects model. CI, confidence interval; IV, inverse variance; SD, standard deviation; [a]CI calculated by Wald-type method; [b]Tau^2 calculated using Restricted Maximum-Likelihood method; Risk of bias legend: (A) bias arising from the randomisation process, (B) bias due to deviations from intended interventions, (C) bias due to missing outcome data, (D) bias in the measurement of the outcome, (E) bias in the selection of the reported result, (F) overall bias.

Study or Subgroup	Fermented foods Mean	SD	Total	Control Mean	SD	Total	Weight	Mean difference IV, Random, 95% CI	Risk of Bias A	B	C	D	E	F
✔ Meance (MTT cohort) 2003	36.8	3.7	40	46.4	2.4	41	51.7%	-9.60 [-10.96 , -8.24]	+	?	+	+	+	?
✔ Meance (STT cohort) 2003	50	6	40	68	9.1	41	48.3%	-18.00 [-21.35 , -14.65]	+	?	+	+	+	?
✖ Spanhaak 1998	29	12	10	36	15	10	0.0%	-7.00 [-18.91 , 4.91]	+	−	+	+	+	−
**Total (Wald[a])**			**80**			**82**	**100.0%**	**-13.65 [-21.88 , -5.43]**						
95% prediction interval								[-27.68 , 0.37]						

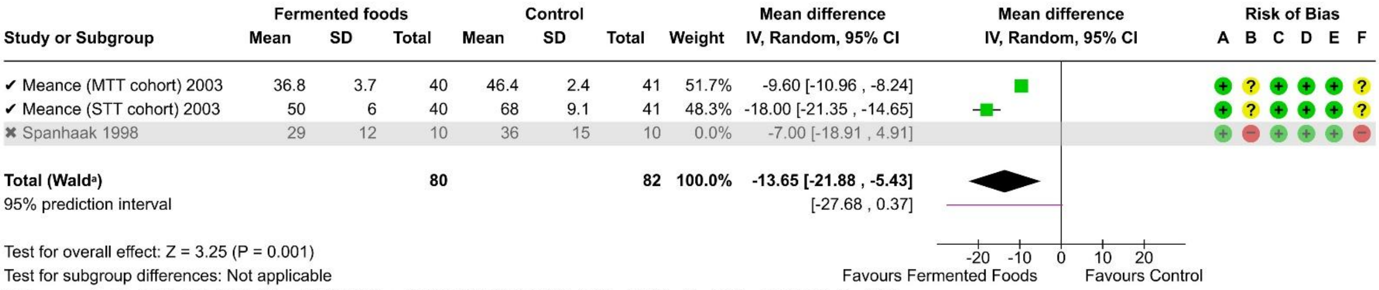

Test for overall effect: Z = 3.25 (P = 0.001)
Test for subgroup differences: Not applicable
Heterogeneity: Tau² (REML[b], 95% CI) = 33.58 [5.32 , >335.78753048780464]; Chi² = 20.74, df = 1 (P < 0.00001); I² = 95%

Supplementary Figure S17F. Forest plot of sensitivity analysis for intestinal transit time. A sensitivity analysis of the forest plot for intestinal transit time in randomised controlled trials comparing fermented foods with control in healthy adults (n=162) was carried out excluding the studies that were deemed at a high risk of bias. Values were calculated as mean difference (95% CIs) using a random-effects model. CI, confidence interval; IV, inverse variance; SD, standard deviation; [a]CI calculated by Wald-type method; [b]Tau^2 calculated using Restricted Maximum-Likelihood method; Risk of bias legend: (A) bias arising from the randomisation process, (B) bias due to deviations from intended interventions, (C) bias due to missing outcome data, (D) bias in the measurement of the outcome, (E) bias in the selection of the reported result, (F) overall bias.

References

Alves, E., Gregório, J., Rijo, P., Rosado, C., and Monteiro Rodrigues, L. (2022). Kefir and the Gut-Skin Axis. *Int J Environ Res Public Health* 19.

Aslam, H., Mohebbi, M., Ruusunen, A., Dawson, S.L., Williams, L.J., Berk, M., Holloway-Kew, K.L., Collier, F., Loughman, A., Pasco, J.A., and Jacka, F.N. (2022). Associations between dairy consumption and constipation in adults: A cross-sectional study. *Nutr Health* 28**,** 31-39.

Galena, A.E., Chai, J., Zhang, J., Bednarzyk, M., Perez, D., Ochrietor, J.D., Jahan-Mihan, A., and Arikawa, A.Y. (2022). The effects of fermented vegetable consumption on the composition of the intestinal microbiota and levels of inflammatory markers in women: A pilot and feasibility study. *PLoS One* 17**,** e0275275.

Guyonnet, D., Naliboff, B., Rondeau, P., Mayer, E., and Chassany, O. (2013). Gastrointestinal well-being in subjects reporting mild gastrointestinal discomfort: characteristics and properties of a global assessment measure. *Br J Nutr* 110**,** 1263-1271.

Guyonnet, D., Schlumberger, A., Mhamdi, L., Jakob, S., and Chassany, O. (2009a). Fermented milk containing Bifidobacterium lactis DN-173 010 improves gastrointestinal well-being and digestive symptoms in women reporting minor digestive symptoms: a randomised, double-blind, parallel, controlled study. *Br J Nutr* 102**,** 1654-1662.

Guyonnet, D., Woodcock, A., Stefani, B., Trevisan, C., and Hall, C. (2009b). Fermented milk containing Bifidobacterium lactis DN-173 010 improved self-reported digestive comfort amongst a general population of adults. A randomized, open-label, controlled, pilot study. *J Dig Dis* 10**,** 61-70.

Kaga, C., Nagino, T., Gomi, A., Takagi, A., Miyazaki, K., Yoshida, Y., and Shida, K. (2024). Effects of fermented soymilk with Lacticaseibacillus paracasei YIT 9029 on gut microbiota and defecation habits: a randomised, double-blind, placebo-controlled study. *Benef Microbes* 15**,** 127-143.

Kato-Kataoka, A., Nishida, K., Takada, M., Kawai, M., Kikuchi-Hayakawa, H., Suda, K., Ishikawa, H., Gondo, Y., Shimizu, K., Matsuki, T., Kushiro, A., Hoshi, R., Watanabe, O., Igarashi, T., Miyazaki, K., Kuwano, Y., and Rokutan, K. (2016). Fermented Milk Containing Lactobacillus casei Strain Shirota Preserves the Diversity of the Gut Microbiota and Relieves Abdominal Dysfunction in Healthy Medical Students Exposed to Academic Stress. *Appl Environ Microbiol* 82**,** 3649-3658.

Kinoshita, T., Maruyama, K., Suyama, K., Nishijima, M., Akamatsu, K., Jogamoto, A., Katakami, K., and Saito, I. (2021). Consumption of OLL1073R-1 yogurt improves psychological quality of life in women healthcare workers: secondary analysis of a randomized controlled trial. *BMC Gastroenterology* 21**,** 237.

Koebnick, C., Wagner, I., Leitzmann, P., Stern, U., and Zunft, H.J.F. (2003). Probiotic Beverage Containing Lactobacillus casei Shirota Improves Gastrointestinal Symptoms in Patients with Chronic Constipation. *Canadian Journal of Gastroenterology and Hepatology* 17**,** 654907.

Kurahashi, A., Enomoto, T., Oguro, Y., Kojima-Nakamura, A., Kodaira, K., Watanabe, K., Ozaki, N., Goto, H., and Hirayama, M. (2021). Intake of Koji Amazake Improves Defecation Frequency in Healthy Adults. *J Fungi (Basel)* 7.

Ling, W.H., Hanninen, O., Mykkanen, H., Heikura, M., Salminen, S., and Von Wright, A. (1992). Colonization and Fecal Enzyme Activities after Oral Lactobacillus GG Administration in Elderly Nursing Home Residents. *Annals of Nutrition and Metabolism* 36**,** 162-166.

Marteau, P., Guyonnet, D., Lafaye De Micheaux, P., and Gelu, S. (2013). A randomized, double-blind, controlled study and pooled analysis of two identical trials of fermented milk containing probiotic Bifidobacterium lactis CNCM I-2494 in healthy women reporting minor digestive symptoms. *Neurogastroenterol Motil* 25**,** 331-e252.

Matsumoto, K., Takada, T., Shimizu, K., Moriyama, K., Kawakami, K., Hirano, K., Kajimoto, O., and Nomoto, K. (2010). Effects of a probiotic fermented milk beverage containing Lactobacillus casei strain Shirota on defecation frequency, intestinal microbiota, and the intestinal environment of healthy individuals with soft stools. *J Biosci Bioeng* 110**,** 547-552.

Meance, S., Chantal, C., Aldo, R., Pietro, T., Claudio, L., and And Antoine, J.-M. (2003). Recent Advances in the Use of Functional Foods: Effects of the Commercial Fermented Milk with Bifidobacterium Animalis Strain DN-173 010 and Yoghurt Strains on Gut Transit Time in the Elderly. *Microbial Ecology in Health and Disease* 15**,** 15-22.

Nagata, S., Asahara, T., Wang, C., Suyama, Y., Chonan, O., Takano, K., Daibou, M., Takahashi, T., Nomoto, K., and Yamashiro, Y. (2016). The Effectiveness of Lactobacillus Beverages in Controlling Infections among the

Residents of an Aged Care Facility: A Randomized Placebo-Controlled Double-Blind Trial. *Ann Nutr Metab* 68**,** 51-59.

Nemoto, H., Ikata, K., Arimochi, H., Iwasaki, T., Ohnishi, Y., Kuwahara, T., and Kataoka, K. (2011). Effects of fermented brown rice on the intestinal environments in healthy adult. *J Med Invest* 58**,** 235-245.

Noda, M., Danshiitsoodol, N., Kanno, K., and Sugiyama, M. (2024). Silk-derived sericin/fibroin mixture drink fermented with plant-derived Lactococcus lactis BM32-1 improves constipation and related microbiota: a randomized, double-blind, and placebo-controlled clinical trial. *Biosci Microbiota Food Health* 43**,** 282-292.

Ozaki, K., Maruo, T., Kosaka, H., Mori, M., Mori, H., Yamori, Y., and Toda, T. (2018). The effects of fermented milk containing Lactococcus lactis subsp. cremoris FC on defaecation in healthy young Japanese women: a double-blind, placebo-controlled study. *Int J Food Sci Nutr* 69**,** 762-769.

Sakai, T., Makino, H., Ishikawa, E., Oishi, K., and Kushiro, A. (2011). Fermented milk containing Lactobacillus casei strain Shirota reduces incidence of hard or lumpy stools in healthy population. *Int J Food Sci Nutr* 62**,** 423-430.

Spanhaak, S., Havenaar, R., and Schaafsma, G. (1998). The effect of consumption of milk fermented by Lactobacillus casei strain Shirota on the intestinal microflora and immune parameters in humans. *European Journal of Clinical Nutrition* 52**,** 899-907.

Sterne, J.A., Savović, J., Page, M.J., Elbers, R.G., Blencowe, N.S., Boutron, I., Cates, C.J., Cheng, H.-Y., Corbett, M.S., and Eldridge, S.M. (2019). RoB 2: a revised tool for assessing risk of bias in randomised trials. *bmj* 366.

Takada, M., Nishida, K., Kataoka-Kato, A., Gondo, Y., Ishikawa, H., Suda, K., Kawai, M., Hoshi, R., Watanabe, O., Igarashi, T., Kuwano, Y., Miyazaki, K., and Rokutan, K. (2016). Probiotic Lactobacillus casei strain Shirota relieves stress-associated symptoms by modulating the gut-brain interaction in human and animal models. *Neurogastroenterol Motil* 28**,** 1027-1036.

Takii, Y., Nishimura, S., Yoshida-Yamamoto, S., Kobayashi, Y., and Nagayoshi, E. (2013). Effects of intake of pickles containing Lactobacillus brevis on immune activity and bowel symptoms in female students. *J Nutr Sci Vitaminol (Tokyo)* 59**,** 402-411.

Tanaka, S., Yamamoto, K., Hamajima, C., Takahashi, F., Endo, K., and Uyeno, Y. (2021). Dietary Supplementation with Fermented Brassica rapa L. Stimulates Defecation Accompanying Change in Colonic Bacterial Community Structure. *Nutrients* 13.

Tanihiro, R., Yuki, M., Sakano, K., Sasai, M., Sawada, D., Ebihara, S., and Hirota, T. 2024. Effects of Heat-Treated Lactobacillus helveticus CP790-Fermented Milk on Gastrointestinal Health in Healthy Adults: A Randomized Double-Blind Placebo-Controlled Trial. *Nutrients* [Online], 16.

Tilley, L., Keppens, K., Kushiro, A., Takada, T., Sakai, T., Vaneechoutte, M., and Degeest, B. (2014). A probiotic fermented milk drink containing lactobacillus casei strain shirota improves stool consistency of subjects with hard stools. *International Journal of Probiotics and Prebiotics* 9**,** 23-30.

Wells, G.A., Wells, G., Shea, B., Shea, B., O'connell, D., Peterson, J., Welch, Losos, M., Tugwell, P., Ga, S.W., Zello, G.A., and Petersen, J.A. (Year). "The Newcastle-Ottawa Scale (NOS) for Assessing the Quality of Nonrandomised Studies in Meta-Analyses").
